# The burden of informal family caregiving in Europe, 2000–2050: a microsimulation modelling study

**DOI:** 10.1016/j.lanepe.2025.101295

**Published:** 2025-04-09

**Authors:** Andrea Cattaneo, Andrea Vitali, Daniele Regazzoni, Caterina Rizzi

**Affiliations:** Department of Management, Information and Production Engineering, University of Bergamo, Bergamo, Italy

**Keywords:** Informal caregivers, Population ageing, Long-term care, Caregiver burden, Computational modelling

## Abstract

**Background:**

The demand for informal care in Europe is increasing, with family members often providing essential support. However, the future burden of informal family caregiving remains unclear. This study estimates and projects trends in the burden of family caregiving across 31 European countries from 2000 to 2050.

**Methods:**

We developed a demographic microsimulation model to estimate and project family care burden. The model produces virtual populations with realistic kinship networks and health trajectories over time. From these kinship structures, we defined a novel metric – Years Lived Caregiving for older relatives (YLCs). It accounts for care recipients’ disability severity, care dynamics within kinship networks, and national institutional care capacity. Model inputs included demographic data from UN World Population Prospects, marital statistics from Eurostat, disease prevalence from the Global Burden of Disease study, and microdata from the SHARE survey.

**Findings:**

From 2000 to 2050, in Europe the overall burden for informal care is projected to increase by +49.7% (95% UI 45–53.6), from 7.98 (7.65–8.28) to 11.9 (11.4–12.5) million YLC. Musculoskeletal disorders are the largest cause of care burden, contributing to 2.3 (2.18–2.42) million YLC in 2050. Burden of informal care is disproportionally higher for women, although the gender gap is in a descending trend.

**Interpretation:**

Projections indicate a substantial rise in the informal family care burden across Europe by 2050, with only limited offsetting from institutional care. These findings underscore the need for comprehensive strategies to support both caregivers and care recipients, ensuring sustainable healthcare systems across Europe.

**Funding:**

Italian government and the 10.13039/501100000780European Union, National Plan for NRRP Complementary Investments (PNC).

**Research in context**

**Evidence before this study**

We conducted a systematic search of PubMed on July 12, 2024, for original research articles using the query “(informal OR family OR potential) AND caregiver AND (projection OR forecast)”. We restricted our search to studies published since 2010, without language restrictions. We screened results for original research articles providing quantitative projections of family caregiving efforts. There is broad consensus that the demand for care in developed countries will generally increase, although the magnitude varies geographically and by the source of impairment. However, while previous works investigated caregiving for functional impairments related to various causes, notably no study conducted a comprehensive assessment of caregiving burden across multiple conditions. Furthermore, all studies assumed static family structures, with none modeling changes in kinship structure over time.

**Added value of this study**

To the best of our knowledge, this is the first comprehensive assessment of informal family caregiving burden across multiple conditions in Europe. Our study introduces a novel metric, Years Lived Caregiving for older adults (YLCs), which accounts for the severity of the disability of care receivers, the likelihood of family members responding to care demands, the number of relatives requiring care, and institutional care capacity. To compute the metric, we designed and implemented a demographic microsimulation model that incorporates changing kinship structures over time, providing more realistic projections of future caregiving demands. Our findings reveal that the informal family care burden in Europe is projected to increase by 50% from 2000 to 2050.

**Implications of all the available evidence**

Our study, combined with existing evidence, underscores the growing challenge of informal caregiving in Europe. The projected increase in the burden of informal care demands highlights the urgent need for comprehensive strategies to support both caregivers and care recipients. Policymakers and healthcare systems must develop interventions that address the increasing care needs to ensure sustainable and equitable healthcare across Europe. Importantly, these strategies should consider the significant gender and age disparities in caregiving. Future research should focus on evaluating the effectiveness of various support strategies and interventions for family caregivers.

## Introduction

The rapidly aging population presents unprecedented challenges for European society. Despite significant efforts, public healthcare systems struggle to keep pace with the increasing demand for care, resulting in a heightened reliance on informal care.^1^ While caregiving has historically been an intrinsic role for family members, the past three decades have seen dramatic increases in the number of caregivers, the duration and intensity of care provided, and the complexity of care delivered.^2^ This trend is driven not only by increasing care demands but also by changing family structures in Europe, characterized by higher proportions of older individuals due to increased life expectancy and fewer young people due to declining fertility rates.^3^

Informal family caregiving encompasses a wide range of care activities, from managing chronic illnesses and disabilities to supporting individuals recovering from acute health events or living with mental health challenges.^2^ Caregivers often provide emotional support, assist with activities of daily living (e.g., bathing, dressing, and eating), manage complex medical regimens, and navigate healthcare systems on behalf of care recipients.^4^ This broad scope highlights the critical role caregivers play not only in filling gaps left by formal care systems but also in sustaining the well-being of individuals across the life course.

However, caregiving comes at a considerable personal cost. Physical health impacts are pervasive, with caregivers reporting higher rates of chronic conditions, compromised immune function, and poorer self-rated health.^5^ Mental health is also frequently affected, with caregivers experiencing elevated levels of stress, anxiety, and depression. The prolonged stress associated with caregiving can lead to burnout, characterized by emotional exhaustion, depersonalization, and a reduced sense of personal accomplishment. Socially, caregivers often face isolation and loneliness as caregiving responsibilities limit their ability to maintain social connections and engage in leisure activities.^6^ The cumulative effect of these stressors can create a cycle where the caregiver’s diminished well-being negatively impacts the quality of care they can provide, potentially compromising the health outcomes of the care recipient.^7^

Sociocultural factors play a significant role in shaping caregiving dynamics. In many European societies, caregiving responsibilities are influenced by cultural norms that emphasize family duty and intergenerational solidarity.^8^ These expectations, often reinforced by gendered social norms, disproportionately burden women, who are more likely to take on intensive caregiving roles at the expense of their own health, financial stability, and professional aspirations.^9^ Gender imbalances in caregiving not only exacerbate inequalities but also reflect deeply rooted biases in how care is valued and distributed within society.

While several studies have addressed the prevalence of informal caregivers in various countries, accurate estimates remain elusive due to varying definitions and sampling methods.^2^ Moreover, there is a lack of understanding of how the rising long term care (LTC) needs translates to care burden among family members, particularly in the context of Europe’s diverse healthcare systems and socioeconomic landscapes.

To address this gap, our study employs a demographic microsimulation model that incorporates virtual populations with realistic kinship structures. This advanced tool allows for the assessment of health trajectories and consequences across population subgroups within and across countries. By integrating demographic data, health trends, and sociocultural factors, we aim to estimate and project trends of informal family care burden across 31 European countries from 2000 to 2050. By elucidating the projected demands and potential impacts on caregivers, our findings will contribute to the evidence base needed for creating sustainable and equitable healthcare strategies across Europe.

## Methods

### Overview

**Fig. 1:**
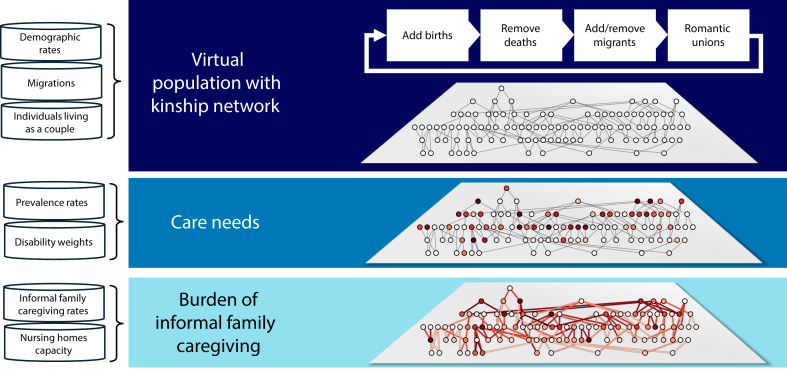
Model diagram.

We developed a stochastic demographic microsimulation model to analyze informal caregiving patterns within family networks. The model incorporates historical demographic data, projected population rates, disease prevalence statistics, and institutional care capacity as primary inputs (Fig. 1). It generates a synthetic population that mirrors each country’s demographic composition and simulates temporal population dynamics—including births, deaths, and partnership formations—to construct realistic kinship networks. For individuals aged ≥ 65 years, the model evaluates care requirements across 359 distinct health conditions. A logistic regression model then identifies likely informal caregivers among relatives, by accounting for demographic characteristics, kinship relationships, and sociocultural factors. The model incorporates bed availability in nursing homes and long-term care facilities to assess the influence of institutional care capacity. To standardize caregiving burden assessment across temporal and geographical contexts, we introduced a novel metric named Years Lived Caregiving for older relatives (YLCs).

A detailed technical description of the model, adhering to the ODD (Overview, Design concepts, Details) protocol (version 2),^10^ is available in the Supplementary appendix (pp. 3–13). The model and data analyses were implemented using Python 3.10. Ethical approval was not required since all data used in this study are de-identified and publicly available.

### Model inputs

The study encompasses 31 European countries. We sourced fertility rates, life tables, demographic counts, and migration data from the UN World Population Prospects 2024. For the period 1950–2023, the data are consolidated, while for 2024–2050, we used UN projections under the medium scenario. Partnership dynamics incorporate both married couples and cohabiting partners. We derived baseline marriage and divorce rates from Eurostat, adjusting them to include consensual unions and cohabitations (Supplementary appendix pp 7–8). These data were projected to 2050 using logistic growth curves. We accounted for both heterosexual and homosexual relationships, with breakdowns based on rates from the OECD Family Database. Prevalence rates of diseases and injuries were obtained from the Global Burden of Disease (GBD) Foresight,^11^ incorporating both historical data and projections. Prevalence rates were modeled as random variables using beta distributions to reflect uncertainty. Institutional care capacity was assessed using Eurostat data on LTC facilities, nursing homes, and LTC beds in hospitals. Projections for institutional care assumed a constant growth rate, accounting for the existing trends in formal care provision. Additionally, we used microdata from Wave 9 of the Survey of Health, Aging, and Retirement in Europe (SHARE) to estimate the likelihood of providing informal care.^12^ To address uncertainties inherent in projected data, sensitivity analyses were conducted. These analyses included variations in population growth dynamics, rates of couple formation and dissolution, alternative health trajectories, and differing growth rates of institutional care capacity.

### Simulation

The simulation was run between the period 1900–2050. The first 100 years of the simulation are needed to articulate the complete kinship structure, hence we considered outputs from 2000 onwards.

Individuals dynamically enter and leave the synthetic population according to births and deaths events, and migrations. During their life cycle, individuals can engage and dissolute romantic relationships, based on marriage and divorce rates adjusted to include consensual unions. As the simulation unrolls, the model keeps track of parents-offsprings links, as well as romantic relationships, resulting in the kinship network of the individuals. Model individuals are representative of inhabitants, with a resolution target of 15,000 agents for the year 2000.

Health status was simulated using data on 359 conditions from the GBD Study. The presence of each condition was determined through Bernoulli trials, with probabilities derived from age-, sex-, time-, and location-specific prevalence rates. Disability weights, reflecting the severity of each condition, were similarly modeled as age-, sex-, time-, and location-specific values, providing a nuanced and accurate representation of health impacts across diverse populations. For individuals aged 65 years or older, the probability of admission to long-term care institutions was modeled probabilistically, with higher disability levels increasing the likelihood of admission. Admissions were constrained by local LTC bed capacities, ensuring alignment with institutional care resources.

We performed 250 model runs for each country included. We expressed results as the average value of all model runs, and uncertainty as 95% percentile intervals.

### Identification of family caregivers

We calibrated and validated a logistic regression model to estimate the probability of individuals becoming informal caregivers for relatives requiring care. The model incorporated eleven regressor variables capturing multidimensional factors influencing caregiving likelihood. These variables included demographic characteristics of care recipients and caregivers, type of kinship, structural family attributes (e.g., number of children), and geographical subregion (e.g., Northern Europe) to systematically account for sociocultural variations in caregiving patterns.^13^

To enhance analytical granularity, we modeled caregiving probabilities using a disaggregated approach that distinguished between gender-specific familial relationships. Rather than aggregating broad kinship categories (e.g., “children”), we separately analyzed probabilities for distinct familial dyads such as sons versus daughters and brothers versus sisters. This methodological approach enabled a nuanced examination of gendered differences in caregiving responsibilities and potential disparities in care provision between men and women.

Model development and validation relied on comprehensive microdata from the Survey of Health, Aging and Retirement in Europe (SHARE), a cross-national panel study encompassing more than 140,000 individuals over Europe.^12^ The analysis incorporated data of caregiving contributions from both in-household and out-of-household family members, ensuring a complete representation of caregiving dynamics. The logistic regression model demonstrated robust predictive performance, with caregiving probability estimates closely aligning with empirical observations (Brier score 0.02; log-loss 0.08). Comprehensive methodological specifications, including detailed model parameters, variable selection rationale, and validation metrics, are reported in the Supplementary appendix (pp. 10–12).

### Years lived caregiving (YLCs)

To quantify the burden of informal care provision for older relatives, we developed the Years Lived Caregiving (YLCs) metric. This novel measure builds upon the established Years of Life lost due to Disability (YLDs) framework, which quantifies time spent in suboptimal health states due to disease or disability.^14^

The YLC metric incorporates three key components: (1) care receiver disability severity, which primarily determines caregivers’ workload^15^; (2) the number of available caregivers, as multiple caregivers can distribute responsibilities^16^; (3) the total number of relatives requiring care, since caregiving burden increases with multiple care recipients.^6^ Disability severity (D) is quantified using standardized disability weights, where 0 represents perfect health and 1 represents a state equivalent to death.^14^

Mathematically, we define the YLCs as the sum of the care burden (CB) experienced by all individuals within a given population:

$$YLC=\sum_{i}CB_{i}$$

where the individual care burden is calculated as the sum of each care receiver’s disability (D) divided by the number of caregivers (N) the care receiver has.

$$CB_{i}=\sum_{j}\frac{D_{j}}{N_{j}}$$

The analysis considers potential care receivers as individuals aged 65 years or older who reside outside institutional care facilities. Caregivers are selected within the kinship network using the logistic model previously described, excluding people in institutionalized care facilities and individuals under the age of 15. Some assumptions underlie this metric: caregiving responsibilities are distributed equally among informal caregivers; the burden increases linearly with disability severity; and there are no interaction effects between caring for multiple relatives. While these simplifications may not fully reflect real-world complexities, they enable systematic quantification of population-level caregiving burden.

### Validation

For each country included in the study, we validated the model outputs against demographic counts, cohabitation shares, and health metrics. This involved comparing the total population count, numbers of newborns, and deaths from 1950 to 2050 against reference data from the UN World Population Prospects. We also validated the population structure for the years 2000, 2021, and 2050. Additionally, we checked the shares of people living in a couple by age group against Eurostat census data. Further details are available in the Supplementary appendix (pp. 19–51).

### Role of the funding source

Study founders had no role in the study design, data collection, data analysis, data interpretation, or writing of the report.

## Results

As of 2021, the overall informal caregiving burden was 10.3 million YLCs (95% UI 9.9–10.7), increasing from 7.98 million (7.65–8.28) in 2000. By 2050, this burden is projected to reach 11.9 million (11.4–12.5), representing a 49.7% (45.0–53.6) increase from 2000. The informal care burden per capita is projected to rise from 21.3 (20.5–21.9) YLCs per 1000 people in 2000 to 31.0 (29.9–32.0) in 2050.

**Fig. 2:**
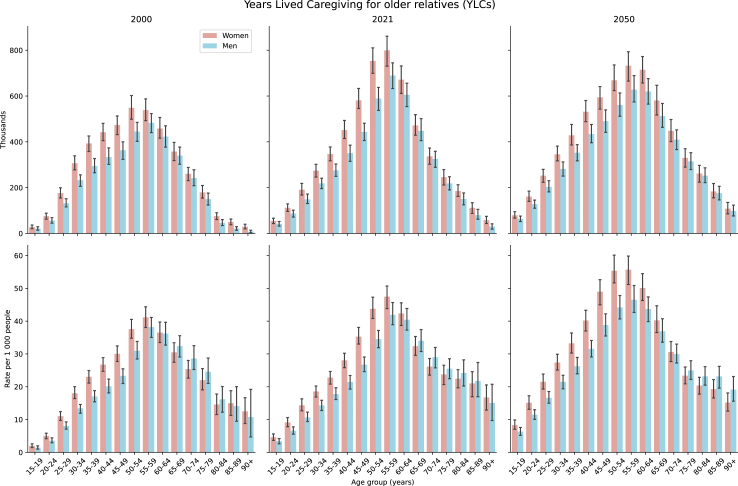
Years lived caregiving (YLCs) in Europe, by age group and sex assigned at birth. Error bars indicate 95% UI. Data are cumulative for selected European countries; plots by individual location are available in the Supplementary appendix (pp.68–83).

The burden of informal familiy caregiving demonstrates significant age-related variation (Fig. 2). The peak caregiving burden occurs in the 50–59 age group, with total YLCs increasing from 2.01 million (95% CI 1.90–2.11) in 2000 to 2.83 million (95% CI 2.70–2.97) in 2021. Gender analysis reveals that women consistently bear a higher caregiving burden, although the gender gap is narrowing over time. In 2000, women accounted for 22.3% (95% CI 17.4–28.1) more total YLCs than men; this disparity decreased to 20.1% (95% CI 15.6–24.4) in 2021 and is projected to decline further to 16.2% (95% CI 12.1–20.7) by 2050. The gender disparity is most pronounced between the ages of 35–54, reaching 28.6% (95% CI 20.5–36.7) in 2021. However, after age 60, men experience a slightly higher per-person caregiving burden compared to women, with substantial caregiving responsibilities extending into older age groups (70+ years). These trends are consistent across European countries, despite regional differences in absolute caregiving levels (Supplementary appendix pp. 68–83).

Musculoskeletal disorders constitute the primary source of informal care burden, accounting for 573 (554–592) YLCs per 100,000 people in 2021, with projections showing an increase to 647 (626–666) in 2050. Among detailed causes, low back pain is the first cause of informal care burden, accounting for 3.0 (2.88–3.12) YLCs per 100,000 people in 2021 (Table 1). Projections indicate it will remain the predominant cause in 2050, rising to 3.26 (3.13–3.38) YLCs per 100,000 people—a 24.4% (19.1–29.9) increase from 2000. The YLC rate for diabetes mellitus is projected to rise by +150% (138–164) by 2050, ranking as the second leading cause of informal care burden. Other conditions with more than a twofold increase include chronic kidney disease (+144%, 127–165), prostate cancer (+125%, 84.6–181), Alzheimer’s disease and other dementias (+125%, 100–150), and Parkinson’s disease (+110%, 61.8–175). Detailed projections by individual cause and condition aggregates are provided in the Supplementary appendix (pp. 58–67).

**Table 1:** Top 30 level-3 causes of years lived caregiving for older relatives (YLCs) in Europe.

	Years lived caregiving (YLC) rate per 100,000				
	2000	2021	2050	Change	
				2021 versus 2000	2050 versus 2000
1. Low back pain	262 (251–274)	300 (288–312)	326 (313–338)	14.6% (10–19.7)	24.4% (19.1–29.9)
2. Diabetes mellitus	101 (95.2–106)	162 (155–169)	252 (242–261)	61% (52.5–70.1)	150% (138–164)
3. Falls	150 (144–156)	182 (174–191)	227 (215–239)	21.4% (16.5–26.1)	51.2% (45–57.9)
4. Age-related and other hearing loss	130 (125–137)	167 (160–173)	220 (212–229)	27.9% (23.6–32.7)	69% (63.1–75.4)
5. Alzheimer’s disease and other dementias	82.1 (74.5–90.4)	121 (112–132)	184 (170–201)	47.6% (32.5–62.4)	125% (100–150)
6. Osteoarthritis	107 (103–112)	135 (129–140)	161 (155–167)	25.7% (21–30.6)	50.4% (43.4–56.6)
7. Oral disorders	89.6 (86.3–93)	99.7 (95.9–104)	119 (115–124)	11.4% (7.52–15.3)	33.3% (28.3–38.3)
8. Depressive disorders	90.6 (83.6–98)	110 (103–119)	113 (105–122)	21.9% (10.1–33)	25.3% (13.8–38)
9. Blindness and vision loss	64.1 (59.8–68.3)	76.9 (72.5–81.7)	96.2 (90.3–102)	19.9% (12.2–28.5)	50.1% (40.7–61.8)
10. Stroke	63.3 (57.6–68.6)	68.4 (61.9–74.4)	91.1 (83.9–98.9)	8.16% (−4.76 to 22)	44% (30.1–61.4)
11. Chronic obstructive pulmonary disease	46.2 (43.5–49.1)	59.5 (55.9–63.1)	83.7 (78.9–88.3)	28.8% (20.4–38.1)	81.3% (70.4–94.4)
12. Anxiety disorders	64.9 (59.3–70.4)	85.2 (78.8–92.1)	81.6 (75.8–87.8)	31.5% (19.5–45.2)	26.1% (14.1–41)
13. Other musculoskeletal disorders	47.9 (43.5–51.9)	61.6 (57.5–65.4)	74.2 (69.9–79.6)	28.8% (16.8–40.5)	55.3% (40.4–75.2)
14. Chronic kidney disease	29.7 (27.7–31.9)	40.8 (38.2–43.4)	72.4 (67.2–77.2)	37.6% (27.4–48.5)	144% (127–165)
15. Headache disorders	66.1 (62.8–69.1)	73.1 (69.7–76.3)	71.8 (68.6–74.8)	10.6% (4.9–17.2)	8.55% (2.19–13.8)
16. Atrial fibrillation and flutter	33.6 (30.5–36.4)	42 (38.9–45.4)	61.5 (57.2–66)	25.4% (12.7–39.6)	83.7% (64.1–106)
17. Neck pain	50.8 (46.6–55.1)	57.7 (53.1–62.3)	61 (56.8–65.5)	13.7% (3.62–26.4)	20.2% (8.48–32.3)
18. Other cardiovascular and circulatory diseases	36.4 (33.3–39.7)	47.1 (43.3–50.7)	58.8 (54.5–63.2)	29.5% (16.1–41)	61.8% (45.3–77.8)
19. Ischemic heart disease	28.2 (26.7–30)	31.1 (29–33)	41.8 (39.5–44.2)	10.1% (2.08–18.4)	48% (38.2–58)
20. Gynecological diseases	40 (37.9–42.4)	42.8 (40.4–45.3)	37.8 (34.7–41.3)	7.11% (−0.225 to 14.9)	−5.26% (−14.1 to 3.74)
21. Gallbladder and biliary diseases	29.3 (27.7–30.8)	30.7 (29.2–32.5)	34.3 (32.4–36.3)	5.14% (−1.36 to 12.8)	17.4% (9.24–26.4)
22. Exposure to mechanical forces	28.6 (27.1–30.1)	28.1 (26.8–29.4)	28.8 (27.4–30.1)	−1.84% (−7.47 to 3.67)	0.698% (−4.37 to 6.93)
23. Alcohol use disorders	22.6 (20.3–25.3)	24.2 (21.6–26.7)	25.4 (23.1–28.2)	7.06% (−6.18 to 22.1)	12.7% (−1.36 to 30.8)
24. Other mental disorders	18.9 (16.9–21)	21.9 (20–24.2)	24.3 (22.3–26.7)	16.5% (1.69–33.3)	29.3% (13.6–45.4)
25. Road injuries	34.3 (31.8–36.9)	21.1 (19.4–23)	22.3 (20.7–24.3)	−38.5% (−44.2 to −32.2)	−35% (−41.1 to −27.5)
26. Other sense organ diseases	15.1 (13.9–16.6)	18.4 (17.2–19.8)	22 (20.3–23.7)	22.1% (11–35.3)	46.1% (30.3–62.3)
27. Bipolar disorder	18.9 (15.6–22.7)	21.6 (18.4–25.5)	21.9 (18.3–25.1)	15.5% (−10.9 to 45.2)	17% (−6.53 to 44.6)
28. Schizophrenia	19.4 (14.1–25.1)	21.3 (15.6–27.5)	21 (15.8–27.2)	12% (−25.5 to 61.7)	10.6% (−27.1 to 65.8)
29. Parkinson’s disease	9.96 (7.67–12)	14.4 (12.2–16.8)	20.7 (17.6–23.6)	46.5% (14.2–94)	110% (61.8–175)
30. Prostate cancer	9.16 (7.49–10.7)	12 (10.4–13.9)	20.5 (17.9–22.8)	32% (6.75–62.7)	125% (84.6–181)

Health conditions are sorted by 2050 rates. The numbers between brackets are the 95% UIs. The full list of causes is provided in the Supplementary appendix (pp. 58–67).

**Fig. 3:**
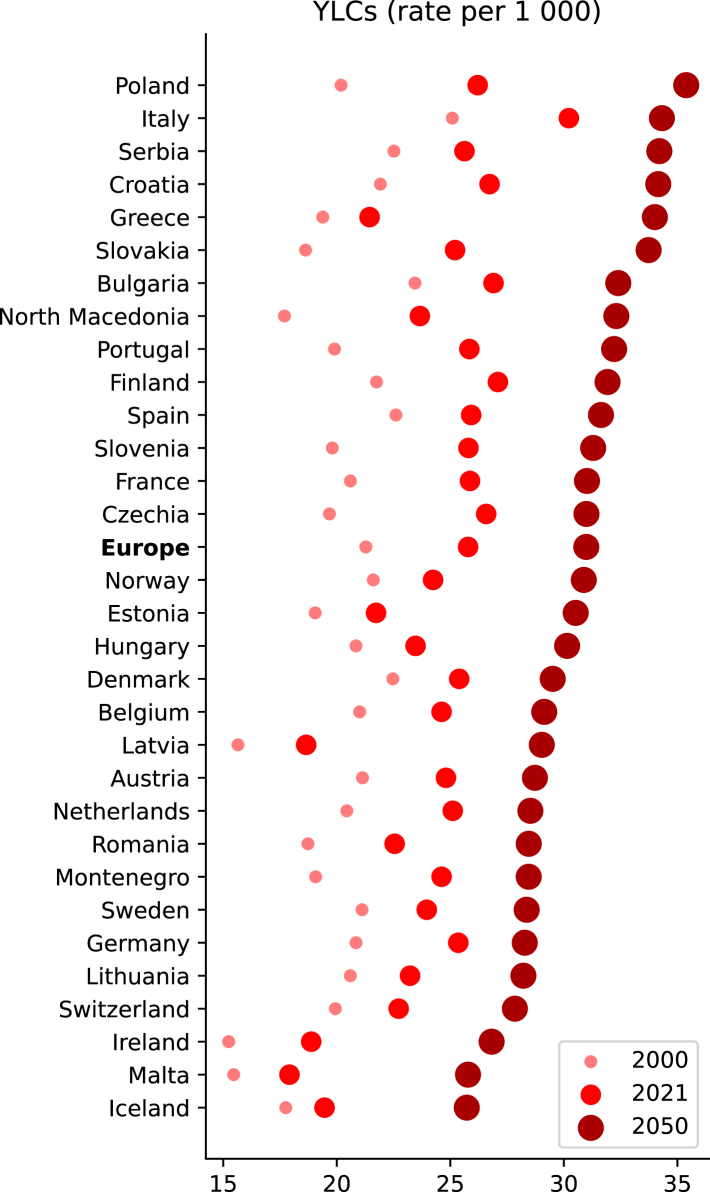
Observed and projected changes in years lived caregiving (YLCs) rate between 2000 and 2050. The size of the dots does not convey specific information; it is utilized to illustrate the direction of the time series and to enhance clarity in cases of overlap. Countries are arranged in descending order according to the 2050 projections.

**Fig. 4:**
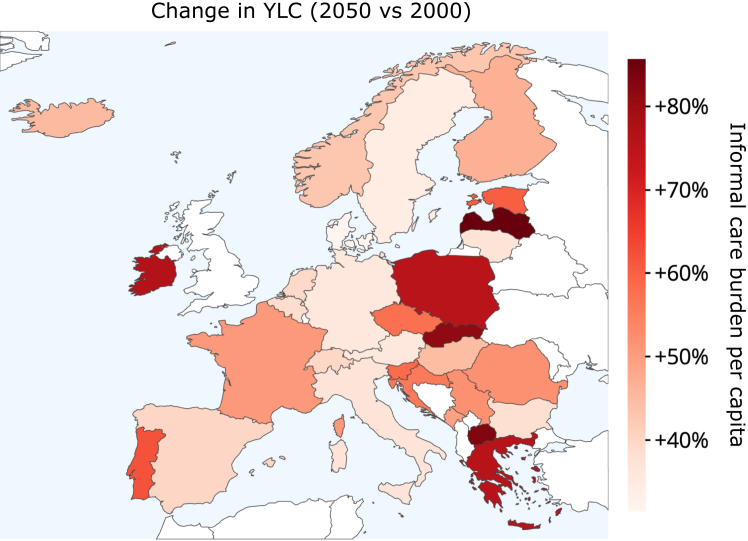
Change in years lived caregiving for older relatives (YLCs), 2000 to 2050.

National analyses reveal substantial local variations (Fig. 3, Fig. 4). By 2050, Poland is projected to have the highest per-person burden of care, with 35.4 (32.8–38.1) YLCs per 1000 people, while Iceland shows the lowest projected burden at 25.7 (24.1–27.2). We identified a correlation between informal care burden per capita and population median age (R^2^ = 0.711, appendix Supplementary Fig. S5). Countries with faster projected population aging show larger increases in per-capita informal care burden. The largest increases from 2000 to 2050 are projected in Latvia (85%, 67–104), North Macedonia (83%, 68–100), and Slovakia (81%, 65–98), with Denmark showing the smallest increase (31%, 22–42). Clear regional patterns emerge in the projected increases, with Eastern European countries showing the highest projected growth in informal care burden. In contrast, Western and Northern European countries generally show more moderate increases, with Denmark and neighboring countries exhibiting the lowest projected growth rates. Southern European countries display intermediate levels of increase, though with notable variation between countries.

Sensitivity analyses show that mortality rates most strongly influence YLC outcomes. A 20% reduction in projected death counts increase YLC rates by 17.3% by 2050, while a 20% mortality increase reduce YLC rates by 16.5% per person. Birth counts have more modest effects: variations of +20% and −20% in fertility rates changed YLC per person by 4.1% and −4.9% respectively. Uncertainty in couple formation and dissolution rates impact YLC rates between −2.2% and +1.2%. Alternative health trajectories assuming reduced risk factor exposure projected YLC rates 12.5% lower (95% CI −13.9 to −10.8) compared with our main analysis. Scenarios modeling accelerated institutional care capacity expansion (threefold faster growth) show a 4.1% (2.6–5.7) decrease in YLC rates by 2050. Further details are reported in the Supplementary appendix (pp. 52–56).

## Discussion

This study provides comprehensive estimates and projections of informal care burden for family members across Europe. Our findings suggest that the burden for informal care per person is expected to increase by 50% (45–54) from 2000 to 2050.

We quantified family care demands using a novel metric, YLCs, which considers the number of individuals with family care responsibilities and the extent of such demands. This metric accounts for the disability level of the care receiver, the likelihood of family members providing care, and the number of relatives requiring care. YLCs are computed using realistic kinship networks obtained through a demographic microsimulation model that we developed and validated. The aim was to comprehensively quantify the informal care burden to enable trend detection across different health conditions, diverse populations, and points in time. Built upon the mathematical framework of Years of healthy Life lost due to Disability (YLDs), YLCs provided an effective quantitative assessment of the burden of informal family caregiving.

Our analysis reveals that the burden of informal caregiving in Europe remains significantly gendered, with women shouldering the majority of caregiving responsibilities. This finding aligns with existing literature and survey data, which consistently highlight the disproportionate caregiving role played by women.^2^ This uneven distribution has substantial implications for women’s participation in the labor market, career progression, and overall economic independence. Women often face greater difficulties balancing paid employment with caregiving duties, leading to higher rates of part-time work, career interruptions, and lower lifetime earnings compared to men. These challenges not only exacerbate gender inequalities in the workplace but also contribute to wider economic disparities, including reduced pension benefits for women in older age.^17^ Encouragingly, our projections indicate that the gender caregiving gap will narrow over time. However, despite these improvements, women are still expected to bear 16% more caregiving responsibilities than men by 2050. This overall trend toward narrowing can be partially attributed to caregiving dynamics among the elderly, paired with extended life expectancies. In older age, caregiving responsibilities are more balanced, with men equaling or even surpassing women in caregiving efforts. These patterns, observed in our simulations, are consistent with findings from the EU-SILC cross-sectional survey.^18^

The present study identifies musculoskeletal disorders as the primary cause of informal caregiving burden. This aligns with previous research highlighting the high prevalence and impact of musculoskeletal conditions in Europe.^19^ In particular, low back pain, recognized as the leading cause of disability globally,^20^ emerges as the predominant contributor to informal care burden. Providing care for individuals with musculoskeletal disabilities often requires significant physical effort, including tasks such as assisting with transfers and mobility. These activities can be particularly strenuous for older caregivers, who often experience physical limitations themselves.^21^ Beyond musculoskeletal conditions, other health challenges pose growing demands on informal caregivers. The rise of more than two fold of informal care burden for people with diabetes carries significant implications. Diabetes often requires extensive and continuous management, including monitoring blood glucose levels, administering medications, meal planning, and managing complications. These caregiving tasks can be particularly challenging for people who lack medical training or sufficient resources to provide optimal care. Of particular concern is the projected doubling of care burden for neurodegenerative conditions such as Alzheimer’s disease and Parkinson’s disease. For many people with dementia, family caregivers are the most important source of support, yet there is growing concern that relatives do not receive adequate support for their caregiving roles.^22^ Relatives of people with dementia experience high levels of physical and emotional strain, which can lead to health issues such as chronic stress and injuries due to the demanding nature of caregiving tasks.^5^

Unsurprisingly, a key factor strongly associated with informal care burden is population aging. Our analysis reveals a robust correlation between median age and YLC per person, a relationship that persists across different countries and time periods. In contrast, while the number of nursing home and LTC beds varies widely across European countries, no clear pattern emerges linking these factors with overall care burden. Indeed, sensitivity analysis revealed that even in optimistic scenarios where institutional care capacity grows, institutional LTC care is unlikely to keep pace with the increasing demands for care. This suggests that demographic shifts—regardless of formal care availability—are the primary drivers of the growing need for informal caregiving. However, it is important to note that in countries with stronger LTC systems, the caregiving burden tends to be more evenly distributed, meaning fewer individuals carry high levels of burden despite comparable overall care demands. These findings are consistent with previous research suggesting a lower prevalence of intensive caregiving in countries with stronger welfare systems, though not a lower number of total informal caregivers.^23^

As a consequence, resources should also be directed to support informal cares. At present, several European countries have introduced policies aimed at providing enhanced support for informal caregivers. These include financial assistance, access to respite care, and the provision of training programs designed to equip caregivers with the requisite skills. However, there is significant national variation in design, regulation, implementation, and outcomes.^24^ For example, in countries like the Netherlands and Denmark, comprehensive caregiving support systems have been developed, including state-funded respite services and caregiver training programs, with positive short-term outcomes for caregiver well-being.^25^ In contrast, southern European countries such as Italy and Spain have more fragmented systems that can result in inconsistent access to services, which may impact long-term caregiver well-being and sustainability.^26^ The increasing demand for family care should urge policymakers to prioritize such interventions. Policies should target reducing the disproportionate impact on women, by promoting gender-equitable caregiving practices and offering targeted financial and social support. Additionally, interventions must account for the unique challenges faced by older caregivers, who may themselves require assistance or have health limitations, to ensure sustainable caregiving arrangements across all age groups.

From a research perspective, there is a pressing need for high-quality studies on interventions for caregivers. A recent meta-analysis found limited convincing evidence of robust effectiveness of interventions, partially attributable to literature that too often overlooks the heterogeneity among caregivers and care receivers.^27^ Interventions designed for caregivers have been reported to improve a wide range of practical, emotional, and relational outcomes for both caregivers and care receivers.^27^ However, some evidence indicates that interventions are most effective when caregivers find flexible, person-centered, and needs-based interventions rather than ‘off-the-rack’ support services.^28^ Furthermore, multi-component interventions may deliver more consistent positive effects, especially when they include both educational and therapeutic components.^27^ Additionally, promising novel opportunities supporting both caregivers and care receivers may come from the enhanced use of digital technologies and telemedicine. For instance, in Sweden, a nationwide program offering telemedicine-based support has demonstrated significant improvements in caregiver satisfaction and health outcomes.^29^ However, access to such technologies is not uniform across Europe, with rural areas and lower-income countries often facing barriers such as limited internet access or lack of funding for digital infrastructure.^29^ Therefore, while these technological solutions hold promise, ensuring equitable access across regions is crucial for their widespread effectiveness.

Given the social and economic implications of informal care, several studies have projected informal care availability for various diseases and locations using a variety of methodologies.^30^, ^31^, ^32^, ^33^, ^34^ However, to the best of our knowledge, no study has adopted a comprehensive approach that stratifies projections by both demographics and conditions, nor have they incorporated changes in family structure into their modeling strategies. As a result, direct comparisons with other studies are challenging. Nevertheless, there is some evidence that our findings point in the same direction as the current literature. Among European studies, Colombo et al. assessed family care for all causes, finding that 20%–50% more family caregivers would be required to maintain the current dependency ratio (caregiver per care receiver) by 2050.^30^ Similarly, Pickard estimated a need for 21% more caregivers in England from 2007 to 2032.^31^ A more recent study by Hu et al., combining a Markov model with a macrosimulation model, found that in England the total hours of informal care will need to increase from 34% to 40% in the following two decades to keep pace with demographic and epidemiological trends.^32^ Furthermore, consistent with our findings, other works suggest that family care for people with dementia is rising faster than the demands for other causes. Using macro- and micro-simulation models, Wittenberg et al. estimated a 52% increase in unpaid care costs per capita for dementia in England from 2015 to 2040,^33^ while Brück et al. projected an increase of 72% in QALYs lost by caregivers of people with dementia in the Netherlands from 2020 to 2050.^34^

While this study offers significant insights, it is essential to recognize several limitations. First, although our model incorporated multiple sociodemographic variables to estimate caregiving probability, some potentially influential factors were not included, such as employment status and geographic proximity between relatives.^5^ Furthermore, the model assumes that the distribution of intra-family caregiving responsibilities remains stable over time. While this approach is grounded in current patterns, future caregiving dynamics may vary. Next, our analysis focused exclusively on care provision within kinship networks, thereby excluding informal care from non-family sources such as friends and neighbors. The primary emphasis of this study is to quantify trends of caregiving among family members, and results should be interpreted accordingly. Our analysis included only a selection of family members, whereas informal care can be provided by people with other roles. However, our analysis covers a wide spectrum of common informal caregivers.^6^ Furthermore, the caregiver pool included individuals aged 15 or more, although some younger individuals engage in caregiving activities. We recognize that younger caregivers may face unique challenges, such as balancing caregiving responsibilities with educational attainment and personal development. Given that data on young carers across Europe is currently limited, future studies could explore the role and impact of caregiving responsibilities on children and adolescents. We opted for this threshold as national surveys and studies suggest that individuals aged 15 or above are capable of providing effective caregiving.^35^ We acknowledge that the use of standardized disability weights, while enabling consistent comparisons across populations and health conditions, may not fully capture the caregiving burden associated with cognitive, emotional, and behavioral dimensions. Next, we should note that model inputs rely on a diverse set of data sources. While our data sources consist of established international organizations and research institutions, we acknowledge that input data may vary in their underlying assumptions and uncertainty levels. However, model validations showed a reliable fit across a broad range of socio-demographic variables. Model projections rely on a set of time series forecasts. While we have propagated uncertainty adopting a stochastic forecasting approach and by running sensitivity analyses, the reliability of future projections is not guaranteed.

In conclusion, a steady increase in the burden of informal care is expected in Europe for at least the next three decades. We hope that these findings will prompt policymakers to devise proactive, sustainable strategies that support both caregivers and care recipients in the context of Europe’s evolving caregiving landscape. As we progress, it will be essential to maintain ongoing monitoring of these trends and adapt our approaches to ensure the well-being of both caregivers and care receivers across Europe.

## Contributors

Conceptualization: Andrea Cattaneo. Data curation: Andrea Cattaneo. Formal analysis: Andrea Cattaneo. Funding acquisition: Daniele Regazzoni, Caterina Rizzi. Methodology: Andrea Cattaneo. Project administration: Andrea Vitali. Resources: Andrea Vitali, Daniele Regazzoni. Software: Andrea Cattaneo. Supervision: Andrea Vitali, Caterina Rizzi. Validation: Andrea Cattaneo. Visualization: Andrea Cattaneo. Writing – original draft: Andrea Cattaneo. Writing – review & editing: Andrea Cattaneo, Andrea Vitali, Daniele Regazzoni, Caterina Rizzi. All authors contributed to interpreting the results and critically revising the draft, and all agreed on the final version.

## Data sharing statement

Model inputs are publicly available from the providers (see Supplementary appendix). Code lists, analysis code, and model code can be provided on request to AC or AV. All data supporting the findings of this study are available within the article and its Supplementary materials.

## Editor note

The Lancet Group takes a neutral position with respect to territorial claims in published maps and institutional affiliations.

## Declaration of interests

All authors have no conflict of interest to declare.
