# Supplementary material for: The burden of informal family caregiving in Europe, 2000–2050: a microsimulation modelling study
Source: Lancet Reg Health Eur. 2025 Apr 9;53:101295. doi: 10.1016/j.lanepe.2025.101295 (PMC12008708; doi:10.1016/j.lanepe.2025.101295)
Supplement: Supplementary appendix [file mmc1.pdf]

Supplementary appendix

Supplement to: “Burden of Informal Family Caregiving in Europe from 2000 to 2050: A Microsimulation Modeling Study”

Andrea Cattaneo, Andrea Vitali, Daniele Regazzoni, and Caterina Rizzi.

Table of contents

MODEL DESCRIPTION (ODD PROTOCOL) 3

1. Purpose and patterns 3

2. Entities, state variables, and scales 3

3. Process overview and scheduling 4

4. Design concepts 5

5. Initialization 6

6. Input data 7

7. Submodels 8

COHORT-COMPONENT BACK-PROJECTION (CCBP) 14

Introduction 14

Method 14

Validation 17

MODEL VALIDATION 19

SENSITIVITY ANALYSES 52

Population Growth Dynamics 52

Couples formation and dissolution 52

Health trajectories 53

Institutional care capacity 54

YEARS LIVED CAREGIVING AND MEDIAN POPULATION AGE 57

YEARS LIVED CAREGIVING BY GBD CAUSE 58

YEARS LIVED CAREGIVING BY SEX ASSIGNED AT BIRTH, AGE GROUP, AND LOCATION 68

DATA SOURCES, LIMITATIONS, AND IMPUTATION METHODS 85

REFERENCES 87

Model description (ODD Protocol)

The model description follows the **ODD (Overview, Design concepts, Details) protocol** for describing individual- and agent-based models [1], as updated by Grimm et al. [2].

1. Purpose and patterns

The primary objective of this microsimulation model is to estimate and project the burden associated with informal care for family members. Currently, estimates of healthy life lost due to disability or ill health are based on prevalence rates of conditions weighted by the condition severity [3]. This model extends these estimates by incorporating kinship networks (i.e., family trees) of selected European countries at the individual level, allowing for an assessment of the relationships between individuals with care needs and their relatives.

To ensure that the model produces realistic patterns and is useful for its intended purpose, its outputs are compared to empirical data on the following aspects:

1. **Population Counts**: This includes overall population size, the number of newborns, and the number of deaths per year. Additionally, age and gender distributions (population pyramids) are considered.
2. **Shares of People Living in a Couple**: While the formation and dissolution of familial ties "by blood" can be tracked through birth and death events, validating the complete kinship structure requires considering romantic unions as well.
3. **Demand for Care**: This encompasses the number of individuals with disability or ill health and the Years Lived with Disabilities (YLDs), a metric that accounts for both the prevalence and severity of conditions.

2. Entities, state variables, and scales

Entities

There are two entities:

- **Person**: represents an inhabitant, characterized by age, sex, and kin relationships.
- **Environment**: represents the location people are living in.

Rationale. Since we're interested in modeling population dynamics and kinship structure, the only entities we need to explicitly model are people living within a location. Furthermore, to represent all the countries in Europe, we performed a separate model run. Hence, location name is part of the environment and not an attribute of people.

State variables

Person

Variable	Type	Range	Dynamic/Static	Description
ID	Integer	[0, +inf)	Static	Identifier of the agent.
Alive	Boolean	{ 0, 1 }	Dynamic	Indicate if the agent is alive or dead.
Age	Integer	[0, 100]	Dynamic	Age of the agent. An age of 100 indicates that the agent is 100 or more years old.
Sex	Categorical	{ M, F }	Static	Sex assigned at birth.
Mother	Integer	[-1, +inf)	Static	ID of the mother of the agent. Value -1 indicates no mother.
Father	Integer	[-1, +inf)	Static	ID of the father of the agent. Value -1 indicates no father.
Partner	Integer	[-1, +inf)	Dynamic	ID of the partner of the agent. Value -1 indicates the agent is not in a romantic relationship.
Partnership time	Integer	[-1, +inf)	Dynamic	It indicates the time elapsed since the start of the romantic relationship, expressed in years. Value -1 indicates the agent is not in a romantic relationship.

Environment

Variable	Type	Range	Dynamic/ Static	Description
Current year	Integer	[1900, 2040]	Dynamic	Current year within the simulation.
Location	Categorical	EU Countries	Static	The name of the country simulated.

Scales

The model run covered the time interval 1900-2050. The first 100 years are needed to fully build the kinship networks (see Section 3). For this reason, we considered model outputs from the year 2000 onwards. The temporal resolution is 1 year. We chose this time unit mainly because (a) model parameters relative to demographics rate and prevalence of conditions available use this time unit and (b) as we are interested in a relatively long timespan (2000-2050), a yearly resolution can effectively capture trends.

The model has no explicit definition of space, apart from the definition of location as a state variable of the environment.

A person entity represents several inhabitants. The resolutions depend on the population size of the country simulated. We aimed at having 15.000 active agents within the simulation as of the year 2000. Hence, we set the scale factor accounting for the country's population growth from 1900 to 2000. This scale allows us to effectively represent population and kinship dynamics in all countries.

3. Process overview and scheduling

The model is developed to cover the entire life cycle of individuals and is structured into nine processes. The first seven processes focus on population dynamics and kinship network building, while the final processes estimates the caregivers' burden.

The processes are scheduled as follows. Steps 1-7 are executed for each iteration, while steps 8-10 are executed only at selected timestamps from 2000 onward, as the full kinship network is required. Details are presented in the Submodels section.

1. **Environment Update**: Increment the current timestamp by one year.
2. **Age and Relationship Duration Update**: Agents increment their age and, eventually, the duration of their romantic relationships.
3. **Births**: Introduce newborns into the simulation.
4. **Deaths**: Remove agents who have died.
5. **Migrations**: Add or remove agents according to migration counts.
6. **New Couples Formation**: Create new romantic couples among agents.
7. **Couples Breakups**: Dissolve romantic unions.
8. **Care Needs**: Mark individuals as any having of the 359 health conditions included.
9. **Institutional Care**: Identify individuals who are in nursing homes or long-term care facilities.
10. **Caregiving Burden**:
    a. Compute the kinship network
    b. Identify informal family caregivers
    c. Estimate the burden for care among family members.

Rationale for processes included

The processes included in the model ensure a comprehensive representation of population changes by incorporating all sources of demographic variation, namely births, deaths, and cross-border migrations. Births, deaths, and migrations are modeled based on age-specific and sex-specific rates to accurately reflect population dynamics and align with reference population pyramids. Using Age-Specific Fertility Rates (ASFR) allows the model to track parentage, ensuring that the relationships between agents are accurately maintained. The inclusion of couple formation and dissolution processes is crucial, as partners often serve as primary caregivers, and thus their relationships significantly impact caregiving dynamics. A prevalence approach to conditions, rather than an incidence approach, is used due to data availability and to align with the methodology of the Global Burden of Disease (GBD) study [4]. These processes enable the construction of realistic kinship networks, which are essential for identifying family caregivers and accurately modeling care demand and burden.

Rationale for scheduling

Updating agents' ages at the start of each year ensures that all individuals are at least age 1 before adding newborns at age 0. Processing deaths after births allow for the possibility of newborn deaths within the same year. By handling deaths, couple formations, and breakups before calculating the caregiving burden, the model accounts for all population shifts, ensuring that the caregiving burden is computed based on the most accurate and current population structure.

The caregiving burden is calculated only after the year 2000, as the initial 100 iterations are needed to build a complete and stable kinship network. Agents are initialized without a specified mother and father, so it's essential to allow enough

time for these relationships to be established through births and deaths. Waiting for 100 years ensures that nearly all agents present at initialization are replaced by those generated during the simulation, with proper parentage and kinship links set, thereby ensuring an accurate and functional kinship network.

4. Design concepts

Basic principles

This model aligns with Mason's definition of demographic models [5]. At its core, the model constructs and evolves virtual populations with realistic structures and kinship networks, providing a comprehensive framework to understand caregiving dynamics within families. The populations and networks emerge from simulations of births, deaths, migrations, and the formation and dissolution of couples over time. These demographic events are randomly triggered based on age-specific and sex-specific rates.

Additionally, the model represents health conditions using a prevalence-based approach, consistent with the methodology of GBD studies [6]. This ensures that the assignment of health conditions to individuals is reflective of actual disease burdens within the population.

Emergence

In this model, the emergent results pertain to care demands and care burdens for rehabilitation needs, which are directly influenced by the size and structure of kinship networks.

Conversely, the prevalence of rehabilitation needs, population growth, and population structure are not emergent properties but are instead imposed based on the input rates. These elements are predetermined by the input data, ensuring that the model adheres to specified demographic and health condition parameters.

Adaptation

In this model, agents exhibit the following adaptive behaviors that influence demographic dynamics:

- **Births**: Female individuals make decisions regarding childbirth, drawn randomly from a Bernoulli distribution. The probability of giving birth is determined by age- and location-specific fertility rates.
- **Deaths**: Individuals may die based on age, sex, and location-specific mortality rates (qx).
- **Romantic Relationships**: Agents decide whether to enter into a romantic relationship based on adjusted marriage rates, which depend on sex, age, and year. These unions can be either heterosexual or homosexual, reflecting the proportion of such marriages within a given location.
- **Couple Dissolution**: Agents also exhibit adaptive behaviors related to couple dissolution through divorce rates. Individuals may choose to terminate their romantic relationships based on age, duration of the relationship, and location-specific divorce rates.
- **Caregiving**: Agents decide to provide informal care to relatives according to probability that depends on the demographic of the (potential) caregiver, the structure of the kinship network, and the type of kinship with the person with care needs.

Objectives

The decision-making process for each behavior is modeled as indirect objective seeking, in which agents follow rules that reproduce observed behaviors (fertility rates, mortality rates, marriage rates, …).

Learning

Agents do not change their behavior over time as a consequence of their experience.

Prediction

Agents do not use explicit or implicit prediction.

Sensing

In this model, agents utilize sensing mechanisms in two separate contexts:

- **Romantic relationship formation**. Agents employ a two-queue system to determine potential romantic relationships. This system matches candidates based on their desire to enter a relationship based on sex, age, and current romantic status. In this context, sensing is global, as all pairs of individuals can potentially be matched.
- **Caregiving needs**. Agents sense caregiving availability from relatives within their kinship network. Relatives taken into account include romantic partners, parents, children, and grandchildren. Agents sense the caregiving needs of these relatives to inform their decisions regarding caregiving responsibilities.

In both cases, sensing is assumed to be instantaneous and accurate.

Interaction

- **Interaction for the Selection of a Romantic Partner**: This interaction is direct and involves all individuals within the population, allowing any individual to potentially form a romantic relationship with any other individual.
- **Interaction for Care Demands and Caregiving**: This interaction is also direct but is limited to individuals interacting with agents within their kinship network. Here, individuals rely on their family members to meet care demands and provide caregiving support.

Stochasticity

We use stochasticity to make model events and behaviors occur with specified frequencies. The following processes are modeled as stochastic:

- **Births**: Women deliver newborns randomly based on age-specific fertility rates.
- **Deaths**: People are removed randomly based on mortality rates.
- **New Couples Formation**: Candidates for romantic unions are selected randomly using marriage rates adjusted by cohabitating ratios.
- **Couples Breakups**: Dissolutions are randomly drawn according to divorce rates.
- **Care Needs**: Conditions and comorbidities are drawn using prevalence rates.
- **Institutional Care**: Individuals staying in nursing homes or long-term care facilities are randomly drawn, with selection weighed by their disability level.
- **Caregiving Burden**: Caregivers are randomly selected, according to demographics, kinship type, and sociocultural factors.

Collectives

The model includes families. Family members share caregiving demands and provisioning. Within the model, we consider as relatives people that have one of the following links: romantic partner, mother, father, daughters, sons, brothers, sisters, and grandchildren. Family are modeled as emerging entirely from agent behaviors, as a result of romantic unions and descendances.

Observation

The principal outputs of interest are related to caregiving dynamics among kin, including the share of relatives requiring rehabilitation, the shares of kin by comorbidities, and the Years Lived Caregiving (YLCs). YLC is a novel metric introduced to quantify the caregiving burden, accounting for the number of relatives requiring care, the severity and comorbidities of their conditions, and the size of their family networks. YLCs are defined as the sum of the caregiving burden experienced by all individuals in the population. More details are provided in Section 7 - Caregiving Burden. These variables are collected at each iteration from 2000 onwards and grouped by sex assigned at birth and 5-year age groups.

Some outputs are observed to validate the model, including demographic counts (population count, newborns, deaths), shares of people living in a couple, and prevalence and YLDs associated with health conditions. These variables are also collected at each iteration and grouped by sex assigned at birth and 5-year age groups.

Lastly, data are collected for decomposition analysis, specifically the rate of relatives by age group and sex.

5. Initialization

Proper initialization of the kinship network in a demographic model typically requires at least 100 years of data to ensure accuracy [5]. However, the available data on fertility and mortality rates from the UN World Population Prospects (WPP) begins only from 1950 onwards [7]. Since our objective is to establish a reliable kinship structure as of the year 2000, the WPP data alone is insufficient for direct initialization. In such scenarios, models are often initialized under the stable growth assumption [5], [8]. This method involves initializing individuals with random ages and sexes, and then simulating population dynamics for over 100 years under the assumption of no migration, with mortality rates set to the most recent data available. However, this approach proved inadequate for our purposes, as it failed to produce a population pyramid that was strictly consistent with the reference population, leading to an inaccurate estimate of the number of individuals requiring rehabilitation.

To address this, we developed a new algorithm, termed Cohort-Component Back-Projection (CCBP), which back-projects the population using age-specific fertility rates and age- and sex-specific life tables. Although our application

of this methodology used constant demographic rates, the CCBP algorithm is equally applicable in scenarios with time-varying rates. For each location included in the analysis, we retrieved population counts, fertility rates, and mortality rates from the UN WPP as of 1950, the earliest available period. Assuming stable growth, we iteratively back-projected the population using these demographic rates until reaching the starting year of the model simulation. This process resulted in a population structure defined by sex at birth and age, with 1-year age-group resolution, which was used to initialize our model. Because this population initialization methodology was both developed and validated, and due to its applicability in other contexts, we have provided a comprehensive description of the procedure in a separate section (see**Error! Reference source not found.**).

6. Input data

We included 31 European countries: Austria, Belgium, Bulgaria, Croatia, Switzerland, Czechia, Denmark, Estonia, Finland, France, Germany, Greece, Hungary, Ireland, Iceland, Italy, Latvia, Lithuania, Montenegro, North Macedonia, Malta, Netherlands, Norway, Poland, Portugal, Romania, Serbia, Slovakia, Slovenia, Spain, and Sweden. The selection was based on data availability.

Demographics rates

We obtained **fertility and mortality rates** from the UN World Population Prospect 2024 [7]. Rates are expressed per location, age with 1-year resolution, and sex assigned at birth. For the period 1950-2023 data are consolidated, while for the period 2024-2040 we used UN projections under the "medium" scenario. For years before 1950 we used the earliest rates available assuming stable growth.

Migrations

While the UN World Population Prospect includes the overall net migration counts, fine-grained data by sex and age are not available. Hence, **migration counts by age and sex** have been reconstructed from population estimates, capturing the difference of populations unexplained by deaths and births. This approach is the same as the one used by the OECD Strategic Public Health Planning for NCDs [9]. In detail, to determine migrations we solved the following equations, where t is time, a is age and s is sex assigned at birth.

When $1 \leq a \leq 99$:

$$Pop(a,s,t) = Pop(a-1,s,t-1) - Deaths(a,s,t) + Migrations(a,s,t) \tag{1}$$

The equation (1) should be modified to handle edge cases. In particular, when computing migrations in the group of newborns ($a = 0$) the equation began:

$$Pop(0,s,t) = Births(s,t) - Deaths(0,s,t) + Migrations(0,s,t) \tag{2}$$

Lastly, since within our model $a = 100$ indicates all people aged 100 or more, for them the equation is:

$$Pop(100,s,t) = Pop(100,s,t-1) + Pop(99,s,t-1) - Deaths(100,s,t) + Migrations(100,s,t) \tag{3}$$

Romantic unions

We obtained **crude marriage rates (CMRs)** from Eurostat [10]. CMRs express the number of marriages per inhabitant over time, by country. As Eurostat provides data from 1960 to 2022, to encompass the full temporal scope of the simulation, we employed Prophet, an open-source time series forecasting tool developed at Meta [11], to generate forecasts for the entire period. In particular, we employed logistic growth curves to project rates over the time interval 1950-2040. Curves were fitted to data from before 2020 to avoid distortions in projections caused by the COVID-19 pandemic.

While CMRs are widely available, in this model we're interested not only in marriages but also in **consensual unions**[1], as they account for a significant proportion of all romantic unions. For this reason, we have adjusted the CMRs to include such relationships. Specifically, we obtained from Eurostat the number of persons in a couple and the number of persons in a married couple for each country. We calculated **adjusted-CMRs** as the product of and the ratio of all couples to married couples.

---

[1] We refer to "consensual union" as the situation when two persons belong to the same household, have a "marriage-like" relationship with each other, and are not married to or in a registered partnership with each other [33].

This model includes **both heterosexual and homosexual unions.** To achieve this, we obtained the proportions of different union types (M-M, F-F, M-F) by country from the OECD Family Database [12].

Since the **rates of engagements in new romantic unions** vary **by age and sex** assigned at birth, we obtained the number of marriages by age, sex, and location from Eurostat [13]. From these distributions, we also calculated the average age gap between couples by year and location.

Lastly, we included the **proportion of first marriages** by age, sex assigned at birth, location and year [14]. Logistic growth curves were used to project values over all simulated timespan.

Unions dissolutions

A non-negligible fraction of marriages and romantic unions broke up for reasons other than the death of the partner. For this reason, we included **crude divorce rates (CDRs)**, which express the number of divorces per marriage by year and location [15]. To cover the entire simulated time interval, we projected the rates over time using logistic growth curves. Furthermore, we included country-specific distribution of **divorces by marriage duration** [16].

Care needs

The Global Burden of Disease (GBD) study is the largest and most comprehensive effort to quantify health loss across places and over time [3]. From the GBD Foresight tool (https://vizhub.healthdata.org/gbd-foresight/) we obtained prevalence rates of health conditions and the associated Years Lived with Disability (YLDs), a metric that weights the prevalence and severity of conditions [17]. We included the 359 causes and conditions aggregates for which YLDs are available. We then calculated age-, sex- and location-specific disability weights (DWs) of the conditions as the ratio of YLDs to prevalence. For projections, in alignment with GBD methodology, we used disability weights as of 2019 [18].

Institutional care capacity

The institutional care capacity of each country was estimated from the availability of beds in long-term care (LTC) facilities, nursing homes, and LTC beds in hospitals. Data were collected from Eurostat [19], [20], except for Portugal where data was obtained from the Portuguese Public Finance Council [21].

For projections up to 2050, we assumed in the main analysis that the average annual increase in the number of beds observed during the period 2000–2021 would remain stable. To evaluate the robustness of this assumption, a sensitivity analysis was conducted to assess how alternative growth scenarios influence the simulation results (see Sensitivity analys).

Caregiving pattern

We analyzed caregiving patterns using microdata from Wave 9 of the Survey of Health, Ageing and Retirement in Europe (SHARE) [22], [23]. This dataset encompasses a cross-national panel of more than 140,000 individuals aged 50 and older, across 20 European countries and Israel. The dataset includes detailed individual-level information on demographics, health status, kinship structures, and whether care was received from relatives.

7. Submodels

Newborns

To incorporate newborns into the simulation, each woman is assigned a probability of giving birth based on the age-specific fertility rate for that particular year. This probability determines which women will have children. For each woman who gives birth, a new agent with age 0 is added to the simulation. The sex of the newborns is randomly assigned according to the sex ratios at birth. Each newborn is given the mother's ID. For the father's ID, two scenarios are considered: if the mother is in a romantic relationship with a man, her partner is assigned as the father; if the mother is single, the father is chosen randomly. For this selection, each potential father within the simulation is assigned a weight based on the age-specific fertility rates of women, adjusted by the average age gap within couples for the given year. This approach ensures that the ages of the fathers are consistent with observed real-world patterns.

Deaths

To simulate death events, life tables were utilized, specifically employing q_x, which represents the probability that an individual of a given age and sex will die within one year. This probability was assigned to each individual based on year- and location-specific data. Mortality was implemented by randomly selecting individuals for removal according to these q_x rates. Upon an individual's removal, their "alive" attribute was set to false. Additionally, if the individual was engaged in a romantic relationship, the "partner" attribute of their partner was updated to -1, indicating the termination of the relationship.

Migrations

At each iteration, agents are dynamically added and removed based on migration counts, which detail the number of individuals entering or exiting the population. These counts are categorized by sex assigned at birth and age, with a resolution of one year. During emigration, individuals are removed according to the specified counts. If an emigrating individual is in a romantic relationship, their partner's relationship status is updated to -1, indicating the dissolution of the relationship. Conversely, during immigration, new individuals are introduced to the population based on the immigration counts, with assignments made according to age and sex. This migration modeling is essential for maintaining a population pyramid that remains coherent with the expected demographic structure.

Formation of romantic couples

This submodel involves the following steps:

1. The expected total number of new couples is computed by multiplying the number of individuals alive by the crude marriage rates, adjusted to include all consensual relationships (see section 6, Input Data – Romantic Unions).
2. The total number of couples was then partitioned into F-F, M-M, and F-M couples according to contry-specific proportions (see section 6, Input Data – Romantic Unions).
3. Couples were created using a "two-queue" system [24]:
    a. Two sets, candidates A and candidates B, were created, each containing the number of individuals equal to the expected number of couples.
    b. Individuals were randomly selected for inclusion in candidates A and candidates B based on age- and sex-specific rates of engagement in romantic unions. Only single individuals (i.e., those not currently in a romantic relationship) were included in the selection.
    c. Candidates A and B were then matched by setting the partner ID attribute of candidate A_i to candidate B_i and vice versa. Additionally, the relationship duration attribute was set to 0.

Couples dissolution

This submodel encompasses the following steps:

1. The number of couple dissolutions (d) was computed by multiplying the crude divorce rates, which represent the number of divorces per marriage, with the total number of couples formed by the submodel "Formation of Romantic Couples." These rates depend on location and time.
2. Couples were selected for dissolution by randomly drawing d couples. The probability of selection was based on the duration of the partnership, weighted according to divorce rates by marriage duration.
3. For individuals involved in a couple dissolution, the attributes PARTNER_ID and PARTNERSHIP_TIME were reset to -1, indicating the termination of their partnership.

Health conditions and disability

For each living individual, we randomly drew whether they had one or more of the 359 conditions from the GBD study. This is done by drawing for each condition using Bernoulli random variables. To account for uncertainty, we modelled prevalence rates using beta distributions. The Beta distribution is a family of continuous probability distributions defined on the interval [0, 1] by two parameters denoted by alpha (α) and beta (β).

$$X \sim Bernoulli(Beta(\alpha, \beta))$$

The parameters alpha and beta were obtained from the mean prevalence and 95% confidence interval reported by the GBD. Wherever fitting a Beta distribution is infeasible (i.e. prevalence rates of zero), we used the mean prevalence rate as the parameter for the Bernoulli variable. Notably, prevalence rates are specific to the location, sex, and age of the individual, and change over time. This approach allowed us to effectively propagate projections uncertainty within model estimates.

Next, we compute the individual's disability level attributable to these conditions using disability weights. Disability weights are measured on a scale from 0 to 1, with 0 indicating full health and 1 indicating a state equivalent to death [25]. According to WHO estimates [26], for individuals with a single condition, the disability weight of that condition is used, while in the presence of comorbidities an additive model is applied:

$$D = \sum_i DW_i$$

where D is the disability level and DW_i are the disability weights of each condition the individual has.

The calculation of the disability level assumes independence of conditions. This means that the probability of having two comorbid conditions is the product of the individual probabilities of each condition. This assumption, currently used by WHO estimates, was tested using data from the UW Medical Expenditure Panel Survey and found to introduce minimal error in magnitude [26].

By implementing these steps, we ensure that our model accurately reflects the distribution and impact of rehabilitative conditions across different demographics over time.

Institutional care

This submodel determines which individuals access institutional care. Eligibility is restricted to agents aged 65 years or older. At each iteration, N individuals are randomly selected for institutionalization, where N represents the total capacity of the institutional care system in a given country at a specific point in time.

Selection probabilities are weighted by the disability levels of eligible individuals, with those experiencing more severe disabilities having a higher likelihood of being institutionalized. This probabilistic approach ensures alignment with real-world patterns of institutional care allocation, where resources are typically prioritized for individuals with the greatest need.

Kinship network

This submodel computes the kinship network of agents, considering the following relationships: romantic partners, parents, offspring, siblings, grandparents, and grandchildren.

The kinship network is represented as the matrix

$$K = \begin{bmatrix} k_{11} & k_{12} & \cdots & k_{1n} \\ k_{21} & k_{22} & \cdots & k_{2n} \\ \vdots & \vdots & \ddots & \vdots \\ k_{n1} & k_{n2} & \cdots & k_{nn} \end{bmatrix}$$

where k_{ij} is 1 if individual i is related to j, 0 otherwise.

To compute the matrix K, we first build the following three matrices:

- K_m for **motherhood** ties, where $k_{ij} = 1$ means agent j is the mother of the agent i, 0 otherwise.
- K_f for **fatherhood** ties, where $k_{ij} = 1$ means agent j is the father of the agent i, 0 otherwise.
- K_{so} for ties with **significant others**, where $k_{ij} = 1$ means agents i and j are in a romantic relationship, 0 otherwise.

The three matrices above are computed from state variables of person entities.

Next, we compute:

- $K_p = K_m + K_f$ for ties with **parents**
- $K_o = (K_p)^T$ for ties with **offspring**
- $K_s = K_p\, K_o - I$ for ties with **siblings**
- $K_{gc} = (K_{gp})^T$ for ties with **grandchildren**

Lastly, we compute the kinship matrix K as the sum of all ties:

$$K = K_{so} + K_p + K_o + K_s + K_{gc}$$

Note that to properly perform these computations, it is necessary to include deceased individuals. Furthermore, all components are sparse matrices, which allows for memory and processing optimization within computer models [27]. It enables a significantly faster and more memory-efficient computation than the linked-lists approach used by other demographic microsimulations such as Socsim and others [24].

Caregivers availability

This submodel aims at identifying the relatives who became caregivers of people with disabilities.

To assess the likelihood of individuals becoming informal caregivers for a relative in need, we utilized microdata from Wave 9 of the Survey of Health, Ageing and Retirement in Europe (SHARE) [22], [23]. This dataset encompasses a cross-national panel of more than 140,000 individuals aged 50 and older, across 20 European countries and Israel.

Data Preparation and Feature Selection

From the SHARE dataset, we selected key predictor variables (features) reported to influence the likelihood of becoming caregivers [28]: demographic factors, kinship relationships, and sociocultural factors. The **features** (regressors) used in the model were:

- Demographics:
  - Gender (DN042)
  - Age (AGE)
  - Marital status (MAR_STAT)
- Kinship:
  - Mother alive (DN026_1)
  - Father alive (DN026_2)
  - Number of daughters alive (CH005_1, CH005_2…, CH005_20)
  - Number of sons alive (CH005_1, CH005_2…, CH005_20)
  - Number of sisters alive (DN037)
  - Number of brothers alive (DN036)
  - Number of grandchildren alive (CH021)
- Geographic subregions (EuroVoc classification):
  - Central and Eastern Europe (COUNTRY)
  - Northern Europe (COUNTRY)
  - Southern Europe (COUNTRY)
  - Western Europe (COUNTRY)

The **outcomes** (targets) were binary variables representing whether the individual receive care from specific kin categories:

- Partner (SP019D1, SP003)
- Sons (SP031, SP027)
- Daughters (SP031, SP027)
- Mother (SP019D2, SP003)
- Father (SP019D3, SP003)
- Brother (SP019D8, SP003)
- Sister (SP019D9, SP003)
- Grandchildren (SP022D1, SP003)

Model Development

We employed a probabilistic framework using a multi-output logistic regression model. The model pipeline consisted of the following steps:

- Standardization: Features were standardized using StandardScaler.
- Classification: A MultiOutputClassifier was used with a logistic regression model (LogisticRegression) wrapped in a CalibratedClassifierCV for probability estimation. The calibration method used was sigmoid scaling, with 5-fold cross-validation. For the logistic regression, we used L2 regularization.

The model was implemented using the Python package *scikit-learn* version 1.5.2.

Postprocessing of Probabilities

For certain targets, such as caregiving by siblings or children, the SHARE survey data indicates whether any individual within the group provided care but does not specify the exact number. To address this limitation, we postprocessed the model's output to estimate per-individual probabilities.

Assuming that all individuals in a given category (e.g., brothers, sons) are equally likely to provide care and act independently, we used the following transformation:

$$p_{individual} = 1 - \left(1 - p_{group}\right)^{1/n}$$

Where p_{group} is the predicted probability that any member of the group provides care, $p_{individual}$ is the estimated probability for a single idividual in the group, and n is the number of individuals in the group (e.g., number of brothers).

This adjustment was applied to the following caregiving targets:

- Sons
- Daughters
- Brothers
- Sisters
- Grandchildren

Nuanced Understanding of Gender Effects

An important strength of our approach lies in the explicit disaggregation of caregiving targets by gender. Instead of aggregating caregiving roles (e.g., combining sons and daughters into "children"), we separately modeled the likelihood of caregiving by gender-specific relatives (e.g., sons vs. daughters, brothers vs. sisters). This approach provides a nuanced understanding of gender-specific dynamics and reveals the degree of skewness in caregiving responsibilities due to gender. For example, this methodology allows us to capture and compare the propensity for daughters versus sons to provide care, reflecting societal norms and gendered expectations within families. Similarly, distinctions between brothers and sisters as caregivers further enrich our understanding of the interplay between kinship and gender roles in caregiving.

Model Evaluation

Model performance was assessed using Brier Score Loss and Log Loss metrics. The results for caregiving outcomes are reported in Table S1.

Table S1. Brier Score and Log Loss for each caregiving outcome, with both metrics assessing the model's probabilistic predictions. The Brier Score ranges from 0 to 1, where lower values indicate better accuracy (0 being perfect prediction). The Log Loss evaluates the alignment of predicted probabilities with true outcomes, with lower scores reflecting better performance.

Help from	Brier Score	Log loss
Partner	0.044	0.168
Sons	0.047	0.174
Daughters	0.048	0.175
Mother	0.005	0.021
Fathers	0.001	0.007
Brothers	0.006	0.032
Sisters	0.006	0.034
Grandchildren	0.001	0.007
AVERAGE	0.020	0.077

Caregiving burden

This submodel computes care burden at the individual level. It considers three key elements. First, the severity of the disability, as higher disability levels result in higher care needs [25]. The second is the number of available caregivers, as multiple caregivers can distribute responsibilities. the total number of relatives requiring care, since caregiving burden increases with multiple care recipients. Disability severity is quantified using standardized disability weights, where 0 represents perfect health and 1 represents a state equivalent to death

We decided to include individuals aged 15 and above as potential caregivers, given that the majority of children and adolescents below this age are unlikely to be in a position to provide care for rehabilitation needs.

Mathematically, we define the YLCs as the sum of the care burden (CB) experienced by all individuals within a given population:

$$YLC = \sum_{i} CB_i$$

where the individual care burden is calculated as the sum of each care receiver's disability (D) divided by the number of caregivers (N) the care receiver has.

$$CB_i = \sum_j \frac{D_j}{N_j}$$

The analysis considers potential care receivers as individuals aged 65 years or older who reside outside institutional care facilities. Caregivers are selected within the kinship network using the logistic model previously described, excluding people in institutionalized care facilities and individuals under the age of 15. Some assumptions underlie this metric: caregiving responsibilities are distributed equally among informal caregivers; the burden increases linearly with disability severity; and there are no interaction effects between caring for multiple relatives. While these simplifications may not fully reflect real-world complexities, they enable systematic quantification of population-level caregiving burden.

Cohort-Component Back-Projection (CCBP)

Introduction

The Cohort-Component Projection (CCP) method is a demographic technique used to project future population numbers based on the age and sex structure of a given population. This method divides the population into cohorts, which are groups of individuals born in the same time period. These cohorts are then projected forward in time based on assumptions about birth rates, death rates, and migration patterns [29]. While the cohort-component projection (CCP) has been extensively used for population forecasting, it is just one among many analytic uses of the model. It is a general theoretical model of population dynamics [30].

Here, we introduce a method that performs the inverse of CCP, namely the cohort-component back-projection (CCBP) method. This method takes as input a population structure at time $t + n$ and age-specific fertility rates and life tables from time t to $t + n$. The result is the population structure at time t. It is based on solving a non-negative least square optimization problem on a recursive equation parametrized with demographic rates.

Method

Let's assume we have a population pyramid at time t represented by the vector

$$p(t) = \begin{bmatrix} p_0(t) \\ p_1(t) \\ \vdots \\ p_{100}(t) \end{bmatrix}$$

where $p_a(t)$ with $a = 0,1,\ldots,99$ is the number of people of age a within the population, and p_{100} represents all people having age 100 or more. For the moment, we ignore the sex of individuals.

First, we want to write the equation that allows us to compute $P(t + n)$ from $P(t)$ and known cohort fertility rates and life table. We need three elements: (i) the increase of people age within vector p, (ii) the removal of people who dies, (iii) the insertion of newborns.

Aging

To perform aging we need to "shift down" the cohorts in the population pyramid. To this aim, we multiply the population vector $p(t)$ by the lower shift matrix L.

$$\underbrace{\begin{bmatrix} 0 & 0 & 0 & \ldots & 0 \\ 1 & 0 & 0 & \ldots & 0 \\ 0 & 1 & 0 & \ldots & 0 \\ \vdots & & \ddots & & \vdots \\ 0 & 0 & \ldots & 1 & 0 \end{bmatrix}}_{L} \cdot \underbrace{\begin{bmatrix} p_0(t) \\ p_1(t) \\ \vdots \\ p_{99}(t) \\ p_{100}(t) \end{bmatrix}}_{p(t)} = \begin{bmatrix} 0 \\ p_0(t) \\ p_1(t) \\ \vdots \\ p_{99}(t) \end{bmatrix}$$

A caveat is that the last item of the population vector, p_{100}, contains all people aged 100 or older. As a consequence, we need to re-add the people shifted out of this group. We can do that using the *lower corner matrix* C.

$$\underbrace{\begin{bmatrix} 0 & \ldots & \ldots & 0 \\ \vdots & \vdots & \vdots & \vdots \\ \vdots & \vdots & \vdots & \vdots \\ 0 & \ldots & 0 & 0 \\ 0 & \ldots & 0 & 1 \end{bmatrix}}_{C} \cdot \underbrace{\begin{bmatrix} p_0(t) \\ p_1(t) \\ \vdots \\ p_{99}(t) \\ p_{100}(t) \end{bmatrix}}_{p(t)} = \begin{bmatrix} 0 \\ 0 \\ \vdots \\ 0 \\ p_{100}(t) \end{bmatrix}$$

Hence, by summing L and C we can increase the age of all individuals.

$$\left(\begin{bmatrix} 0 & 0 & 0 & \ldots & 0 \\ 1 & 0 & 0 & \ldots & 0 \\ 0 & 1 & 0 & \ldots & 0 \\ \vdots & & \ddots & & \vdots \\ 0 & 0 & \ldots & 1 & 0 \end{bmatrix} + \begin{bmatrix} 0 & \ldots & \ldots & 0 \\ \vdots & \vdots & \vdots & \vdots \\ \vdots & \vdots & \vdots & \vdots \\ 0 & \ldots & 0 & 0 \\ 0 & \ldots & 0 & 1 \end{bmatrix} \right) \cdot \begin{bmatrix} p_0(t) \\ p_1(t) \\ \vdots \\ p_{99}(t) \\ p_{100}(t) \end{bmatrix} = \begin{bmatrix} 0 \\ p_0(t) \\ p_1(t) \\ \vdots \\ p_{99}(t) + p_{100}(t) \end{bmatrix}$$

For convenience, we define the matrix S as the sum of L and C.

$$\underbrace{\begin{bmatrix} 0 & 0 & 0 & \dots & 0 \\ 1 & 0 & 0 & \dots & 0 \\ 0 & 1 & 0 & \dots & 0 \\ \vdots & & \ddots & & \vdots \\ 0 & 0 & \dots & 1 & 0 \end{bmatrix}}_{L} + \underbrace{\begin{bmatrix} 0 & \dots & \dots & 0 \\ \vdots & \vdots & \vdots & \vdots \\ \vdots & \vdots & \vdots & \vdots \\ 0 & \dots & 0 & 0 \\ 0 & \dots & 0 & 1 \end{bmatrix}}_{C} = \underbrace{\begin{bmatrix} 0 & 0 & 0 & \dots & 0 \\ 1 & 0 & 0 & \dots & 0 \\ 0 & 1 & 0 & \dots & 0 \\ \vdots & & \ddots & & \vdots \\ 0 & 0 & \dots & 1 & 1 \end{bmatrix}}_{S}$$

Deaths

The expected number of deaths in each age group, denoted as $d_a(t)$, can be computed using life tables. In this context, $q_x(t)$ represents the probability that a person of exact age x will die within one year at time t. To determine the expected number of deaths for each age group, we construct a diagonal matrix where each diagonal element corresponds to $q_x(t)$ for the respective age. By multiplying the population vector by this diagonal matrix, we obtain the expected number of deaths for each age group. This method provides a systematic calculation of the number of removals from the population due to mortality.

$$\underbrace{\begin{bmatrix} d_0(t) \\ d_1(t) \\ \vdots \\ \vdots \\ d_{100}(t) \end{bmatrix}}_{d(t)} = \underbrace{\begin{bmatrix} q_0(t) & 0 & 0 & \dots & 0 \\ 0 & q_1(t) & 0 & \dots & 0 \\ 0 & 0 & q_2(t) & \dots & 0 \\ \vdots & \vdots & \vdots & \ddots & \vdots \\ 0 & 0 & 0 & \dots & q_{100}(t) \end{bmatrix}}_{Q(t)} \underbrace{\begin{bmatrix} p_0(t) \\ p_1(t) \\ \vdots \\ \vdots \\ p_{100}(t) \end{bmatrix}}_{p(t)}$$

Newborns

The expected number of newborns at time $t-1$ who reach the following year t can be computed using age-specific fertility rates (ASFR) adjusted by infant mortality rates. To do so, we define $f_a(t)$ as follows:

$$f_a(t) = ASFR_a(t) \cdot \big(1 - q_0(t)\big)$$

Here, $f_a(t)$ represents the adjusted fertility rate for age group a at time t, taking into account the survival probability of infants. We can then compute the number of newborns with the following equation:

$$\underbrace{\begin{bmatrix} p_0(t) \\ 0 \\ \vdots \\ 0 \end{bmatrix}}_{b(t)} = \underbrace{\begin{bmatrix} f_0(t) & f_1(t) & \dots & f_{100}(t) \\ 0 & 0 & \dots & 0 \\ \vdots & \vdots & \dots & \vdots \\ 0 & 0 & \dots & 0 \end{bmatrix}}_{F(t)} \underbrace{\begin{bmatrix} p_0(t) \\ p_1(t) \\ \vdots \\ p_{100}(t) \end{bmatrix}}_{p(t)}$$

This matrix multiplication results in the vector $b(t)$ where the first element represents the number of people aged 0 at time t.

Recursive equation of population growth

Assuming no migrations, the population at time t can be computed as follows

$$p(t) = S\big(p(t-1) - d(t-1)\big) + b(t-1)$$

Which can be rewritten as

$$p(t) = S\big(p(t-1) - Q(t-1)p(t-1)\big) + F(t-1)p(t-1)$$

$$p(t) = \underbrace{[S - S \cdot Q(t-1) + F(t-1)]}_{A(t-1)} p(t-1) \tag{1}$$

$$p(t) = A(t-1) \cdot p(t-1)$$

The recursive equation can be propagated at n time steps

$$p(t) = \left(\prod_{i=1}^{n} A\,(i)\right) p(t-n) \tag{2}$$

Notably, under the assumption of constant demographic rates, the matrix A is time invariant. Hence, the equation can be simplified as

$$p(t) = A^{n} \cdot p(t-n)$$

Backward Projection

The matrix A is singular, preventing us from directly solving the equation. However, we can find a suitable solution using convex optimization techniques. Specifically, we aim to ensure that all elements of $p(t-n)$ are non-negative. This leads us to employ non-negative least squares (NNLS) algorithms. The optimization problem can be formulated as follows:

$$\underset{p(t-n)}{\arg\min} \left\| \left(\prod_{i=1}^{n} A\,(i)\right) p(t-n) - p(t) \right\|_2^2 \quad \text{subject to } p(t-n) \geq 0$$

Extension: age- and sex-cohorts

To introduce sex cohorts, we can extend the proposed methodology using block matrices. First, we define the vector $\mathcal{P}$ as the concatenation of female and male vectors. The subscript f stands for females and m males respectively.

$$\mathcal{P}(t) = \begin{bmatrix} p_f(t) \\ p_m(t) \end{bmatrix} = \begin{bmatrix} p_{f0}(t) \\ \vdots \\ p_{f100}(t) \\ p_{m0}(t) \\ \vdots \\ p_{m100}(t) \end{bmatrix}$$

Then, we define the following block matrices

$$\mathcal{L} = \begin{bmatrix} L & 0 \\ 0 & L \end{bmatrix} \qquad \mathcal{C} = \begin{bmatrix} C & 0 \\ 0 & C \end{bmatrix} \qquad \mathcal{S} = \begin{bmatrix} S & 0 \\ 0 & S \end{bmatrix}$$

For mortality matrices, we include both female and male rates as follows.

$$\mathcal{Q} = \begin{bmatrix} Q_f(t) & 0 \\ 0 & Q_m(t) \end{bmatrix}$$

As the probability of a newborn to be male or female is not the same, nor constant over time, we need to multiply fertility rates by the expected sex ratio at birth.

$$\mathcal{F} = \begin{bmatrix} r_f F_f(t) & 0 \\ \left(1 - r_{f(t)}\right) F_f(t) & 0 \end{bmatrix} \qquad \text{where } r_f = \frac{\text{female newborns}}{\text{total newborns}}$$

Hence, equation (1) can be rewritten as follows.

$$\mathcal{P}(t) = \underbrace{\left[\mathcal{S} - \mathcal{S} \cdot \mathcal{Q}(t-1) + \mathcal{F}(t-1)\right]}_{\mathcal{A}(t-1)} \mathcal{P}(t-1)$$

$$\mathcal{P}(t) = \mathcal{A}(t-1) \cdot \mathcal{P}(t-1)$$

Similarly as equation (2), the recursive equation can be propagated at n time steps.

$$\mathcal{P}(t) = \left(\prod_{i=1}^{n} \mathcal{A}\,(i) \right) \mathcal{P}(t-n)$$

Validation

To validate the proposed method, CCBP, we initialized several population structures from various European countries. The CCBP method was then applied with back-projection intervals of $n = \{1, 5, 10, 25, 50\}$ to estimate the population at time $t - n$. Subsequently, the CCP method was employed over n years to project the population forward. The accuracy of the re-projected population was assessed by calculating the mean absolute percentage error (MAPE) between the reference population and the re-projected population. The actual demographic rates used for the simulations were sourced from the UN World Population Prospects 2024 [7], ensuring the use of reliable and up-to-date data. To solve the optimization problem inherent in the CCBP method, we utilized the function *optimize.nnls()* from the open-source Python package *scipy* (version 1.13) [31].

Table S2 summarizes the results, highlighting the effectiveness of the CCBP method. Notably, the average MAPE across the selected countries was 0.01% for a one-year interval and 4.43% for a 50-year interval. These results demonstrate the method's precision in short-term projections and its reasonable accuracy over longer periods.

Table S2. Mean absolute percentage error (MAPE) of the CCBP method.

**Location**	**Back-projection timespan (years)**				
	**1**	**5**	**10**	**25**	**50**
Austria	0.00%	0.09%	0.29%	1.51%	3.56%
Belgium	0.00%	0.06%	0.24%	1.51%	3.80%
Bulgaria	0.01%	0.05%	0.10%	0.65%	7.00%
Croatia	0.01%	0.08%	0.14%	0.61%	4.56%
Czechia	0.00%	0.07%	0.26%	1.49%	3.15%
Denmark	0.01%	0.09%	0.25%	1.38%	3.50%
Estonia	0.02%	0.05%	0.06%	0.35%	5.15%
Finland	0.01%	0.06%	0.26%	1.59%	4.36%
France	0.00%	0.05%	0.18%	1.22%	3.70%
Germany	0.01%	0.10%	0.29%	1.41%	3.72%
Greece	0.01%	0.06%	0.10%	0.28%	2.15%
Hungary	0.00%	0.08%	0.25%	1.21%	4.15%
Iceland	0.01%	0.11%	0.30%	1.49%	3.24%
Ireland	0.00%	0.05%	0.19%	1.35%	3.14%
Italy	0.01%	0.05%	0.22%	1.57%	4.29%
Latvia	0.02%	0.10%	0.19%	1.12%	6.88%
Lithuania	0.02%	0.10%	0.22%	1.37%	6.42%
Malta	0.00%	0.05%	0.20%	1.31%	3.70%
Montenegro	0.01%	0.03%	0.06%	0.63%	8.31%
Netherlands	0.00%	0.08%	0.23%	1.42%	3.91%
North Macedonia	0.02%	0.08%	0.14%	0.69%	5.54%
Norway	0.00%	0.07%	0.24%	1.44%	3.47%
Poland	0.02%	0.09%	0.16%	0.85%	5.38%
Portugal	0.00%	0.06%	0.18%	1.16%	4.17%
Romania	0.01%	0.05%	0.12%	0.66%	5.78%
Serbia	0.01%	0.06%	0.12%	0.66%	4.53%

Location	Back-projection timespan (years)				
	**1**	**5**	**10**	**25**	**50**
Slovakia	0.01%	0.04%	0.07%	0.60%	4.47%
Slovenia	0.00%	0.09%	0.33%	1.79%	3.26%
Spain	0.01%	0.04%	0.19%	1.43%	4.12%
Sweden	0.00%	0.08%	0.28%	1.65%	4.14%
Switzerland	0.01%	0.10%	0.32%	1.62%	3.66%
**Average**	**0.01%**	**0.07%**	**0.20%**	**1.16%**	**4.43%**

Model validation

This section presents the results of the validation of the model output against reference values. The evaluation metric used was the Mean Absolute Percentage Error (MAPE). For each country included, we performed 250 model runs with different random seeds. Table S3 shows the metric used for the evaluation and the source of the reference values. Table S4 shows the validation results for each country included. Figure S1 presents plots of model output with 95% percentile intervals, as well as reference values.

Table S3. Description of parameters assessed for validation and sources of reference values.

Parameter	Description	Timestamps evaluated	Reference source
Population	Total population.	All years within the range 1950-2050.	United Nations' World Population Prospects (UN WPP 2024) [7].
Births	Number of newborns.		
Deaths	Number of deaths.		
Population structure	The distribution of people in a population according to sex assigned at birth and age.	2000, 2021, and 2050.	
People in a couple	Number of people living in a couple. Within the context of this work, the term "couple" includes married couples, couples in registered partnerships, and couples who live in a consensual union.	2011.	Eurostat Population and Housing Census [32].
Care needs	Prevalence and Years Lived with Disability (YLDs) attributable to 359 health conditions.	2000, 2010, 2021, 2030, 2040, and 2050	GBD Foresight [18].

Table S4. Summary of mean absolute percentage errors of model simulation. The last row indicates averages weighted by population counts. Errors for people in a couple in Montenegro, North Macedonia, amd Serbia are not computed due to the unavailability of reference data.

	Mean Absolute Percentage Error								
**Location**	**Population**	**Births**	**Deaths**	**Population structure**			**People in a couple**	**Rehabilitation needs**	
				**2000**	**2019**	**2040**		**Prevalence**	**YLDs**
Austria	0.84%	6.02%	5.86%	6.24%	4.66%	6.04%	1.78%	4.35%	3.61%
Belgium	0.85%	5.73%	5.94%	5.34%	4.46%	6.27%	2.52%	3.09%	2.75%
Bulgaria	1.38%	6.14%	5.78%	7.61%	8.16%	12.98%	3.23%	4.91%	7.44%
Croatia	1.45%	6.10%	5.84%	6.78%	6.41%	6.81%	2.98%	4.68%	3.36%
Czechia	0.92%	5.85%	5.89%	7.18%	6.14%	12.86%	25.10%	1.26%	1.94%
Denmark	0.89%	5.65%	6.17%	5.95%	4.78%	6.98%	4.19%	2.22%	1.89%
Estonia	0.89%	6.02%	5.77%	5.49%	5.54%	8.16%	5.54%	1.73%	2.03%
Finland	0.94%	5.90%	6.30%	7.35%	5.64%	6.87%	3.11%	1.12%	1.76%
France	1.06%	5.45%	6.27%	6.67%	6.23%	11.13%	5.02%	1.74%	2.22%
Germany	0.83%	6.26%	5.83%	6.94%	6.22%	7.74%	6.67%	4.09%	3.30%
Greece	1.37%	6.36%	6.47%	7.20%	5.69%	6.26%	5.06%	6.23%	7.20%
Hungary	0.91%	6.05%	5.60%	5.31%	5.10%	7.76%	7.66%	2.22%	2.27%
Iceland	0.80%	4.95%	7.56%	5.62%	4.24%	7.96%	10.53%	4.78%	3.57%
Ireland	0.76%	4.84%	6.50%	6.00%	4.54%	6.11%	14.12%	2.04%	2.05%
Italy	1.19%	6.40%	6.14%	5.73%	5.21%	6.89%	24.28%	2.09%	3.40%
Latvia	0.99%	6.54%	5.73%	6.49%	7.01%	10.41%	16.69%	5.10%	3.78%
Lithuania	1.08%	6.43%	6.29%	6.39%	6.37%	13.98%	9.15%	8.05%	6.11%
Malta	0.76%	5.66%	6.57%	8.93%	6.59%	8.67%	22.29%	7.44%	7.95%
Montenegro	1.04%	5.32%	6.63%	6.78%	6.87%	10.53%	--	1.75%	3.27%
Netherlands	0.95%	5.63%	6.85%	5.28%	4.93%	7.47%	2.26%	3.54%	4.52%
North Macedonia	1.86%	5.50%	6.67%	7.39%	13.04%	20.08%	--	10.42%	8.41%
Norway	0.72%	5.53%	6.44%	6.30%	4.36%	8.42%	1.44%	2.36%	1.86%
Poland	1.20%	5.99%	6.48%	6.28%	6.42%	8.96%	10.02%	1.41%	2.46%
Portugal	1.40%	5.79%	6.11%	5.26%	5.37%	10.76%	4.04%	2.82%	2.49%
Romania	1.25%	5.88%	6.11%	5.71%	6.24%	8.82%	5.77%	3.51%	3.36%
Serbia	1.55%	6.23%	6.01%	8.82%	8.80%	8.30%	--	20.99%	16.58%
Slovakia	0.96%	5.73%	6.44%	5.31%	4.98%	7.11%	28.01%	1.09%	2.21%
Slovenia	1.08%	5.90%	6.28%	5.09%	4.78%	7.07%	12.17%	2.41%	2.13%
Spain	1.27%	5.97%	6.62%	6.98%	5.27%	7.50%	1.54%	4.59%	3.73%
Sweden	0.79%	5.72%	6.12%	6.64%	4.85%	7.09%	5.30%	5.49%	4.26%
Switzerland	0.78%	5.82%	6.53%	5.74%	4.49%	6.56%	13.97%	3.81%	3.89%
**Europe**	**1.07%**	**5.97%**	**6.20%**	**6.42%**	**5.79%**	**8.40%**	**8.73%**	**3.41%**	**3.46%**

Figure S1. Model output and reference data of the parameters used for validation, for each location. Uncertainty is expressed as 95% percentile intervals and shown as shades. The Mean Absolute Percentage Error (MAPE) is displayed over each plot.

Austria

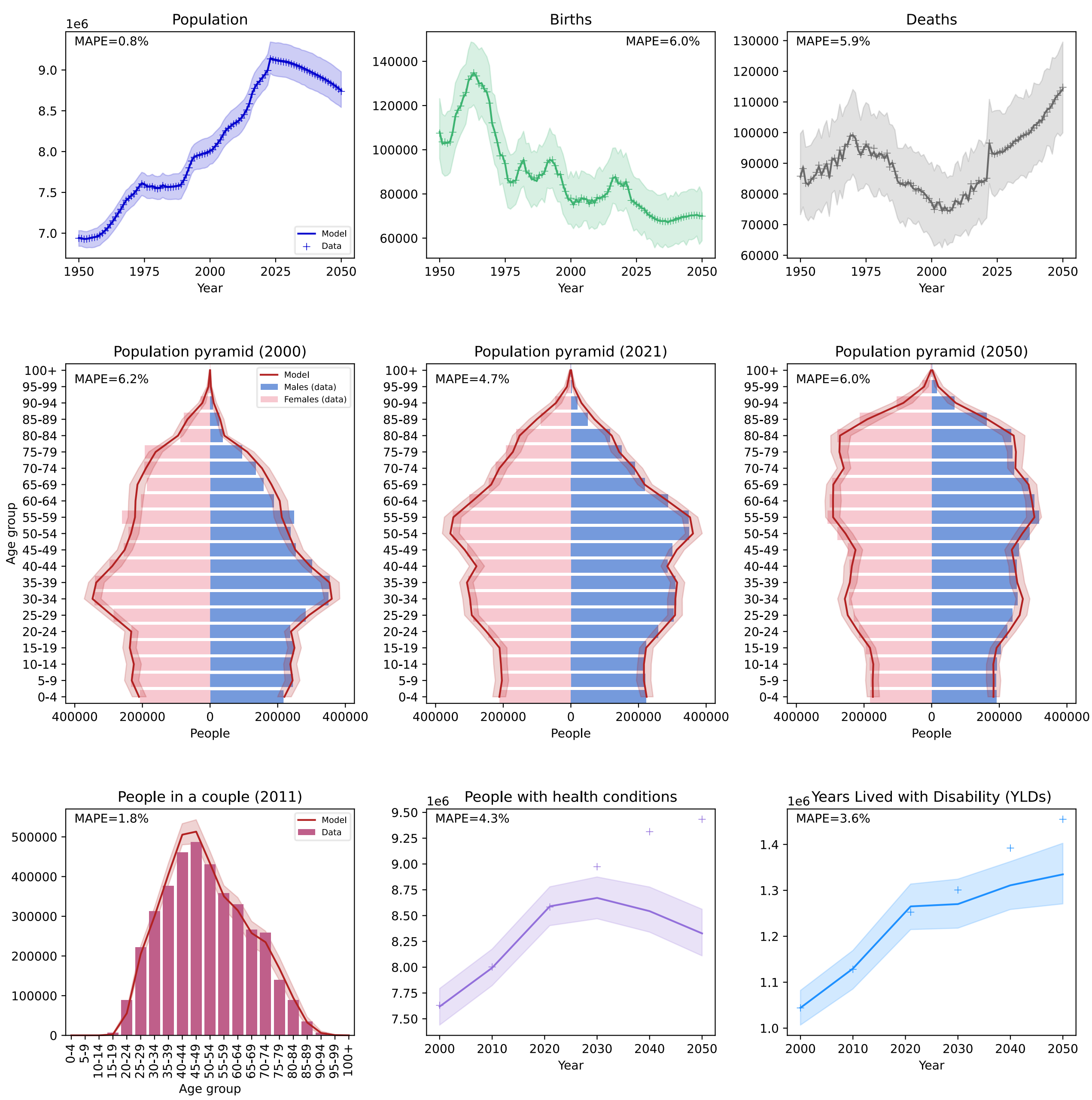

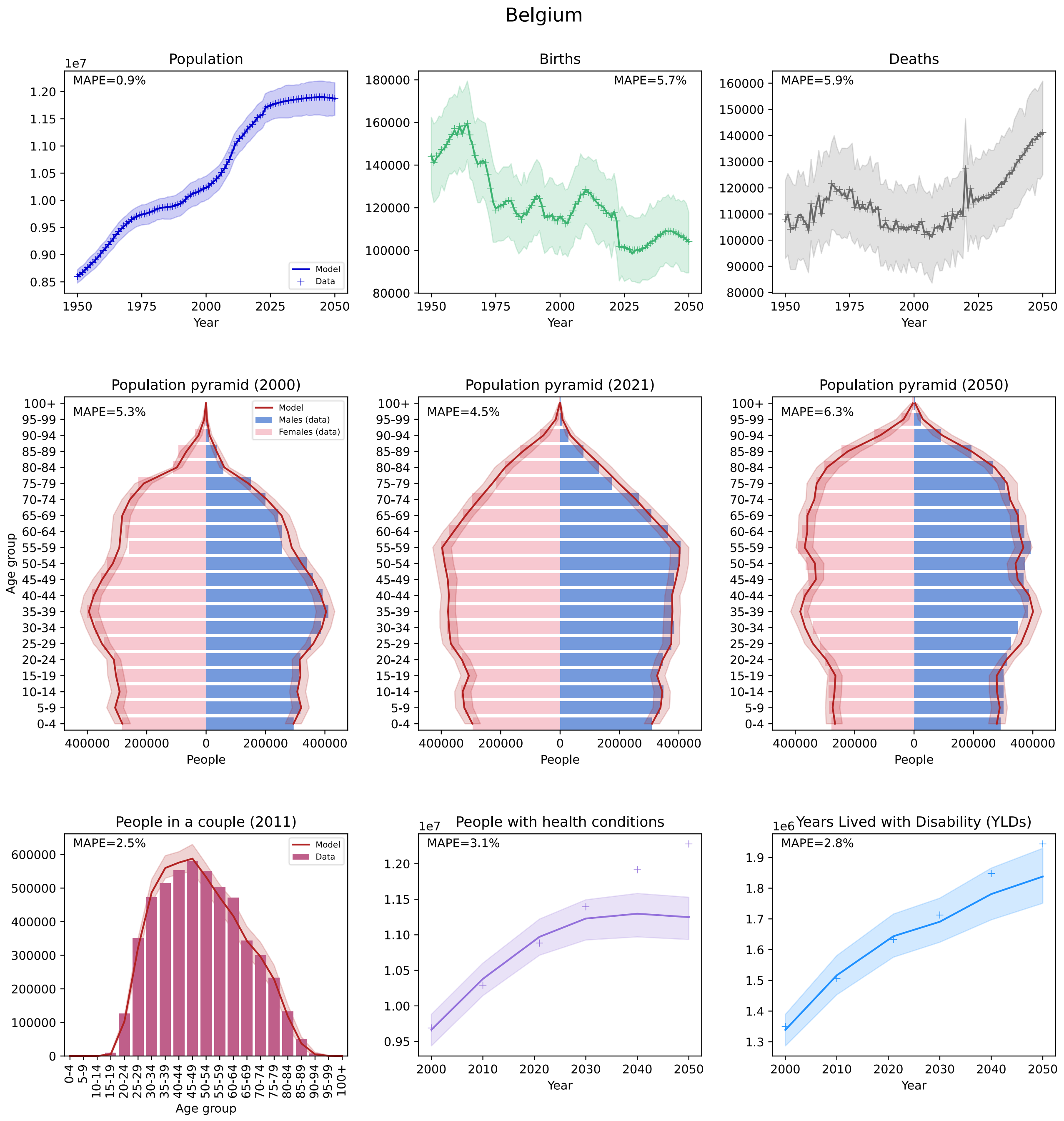
Belgium
Population
1e7
MAPE=0.9%
Model
Data
Year
Births
MAPE=5.7%
Year
Deaths
MAPE=5.9%
Year
Population pyramid (2000)
MAPE=5.3%
Model
Males (data)
Females (data)
Age group
People
Population pyramid (2021)
MAPE=4.5%
People
Population pyramid (2050)
MAPE=6.3%
People
People in a couple (2011)
MAPE=2.5%
Model
Data
Age group
People with health conditions
1e7
MAPE=3.1%
Year
Years Lived with Disability (YLDs)
1e6
MAPE=2.8%
Year

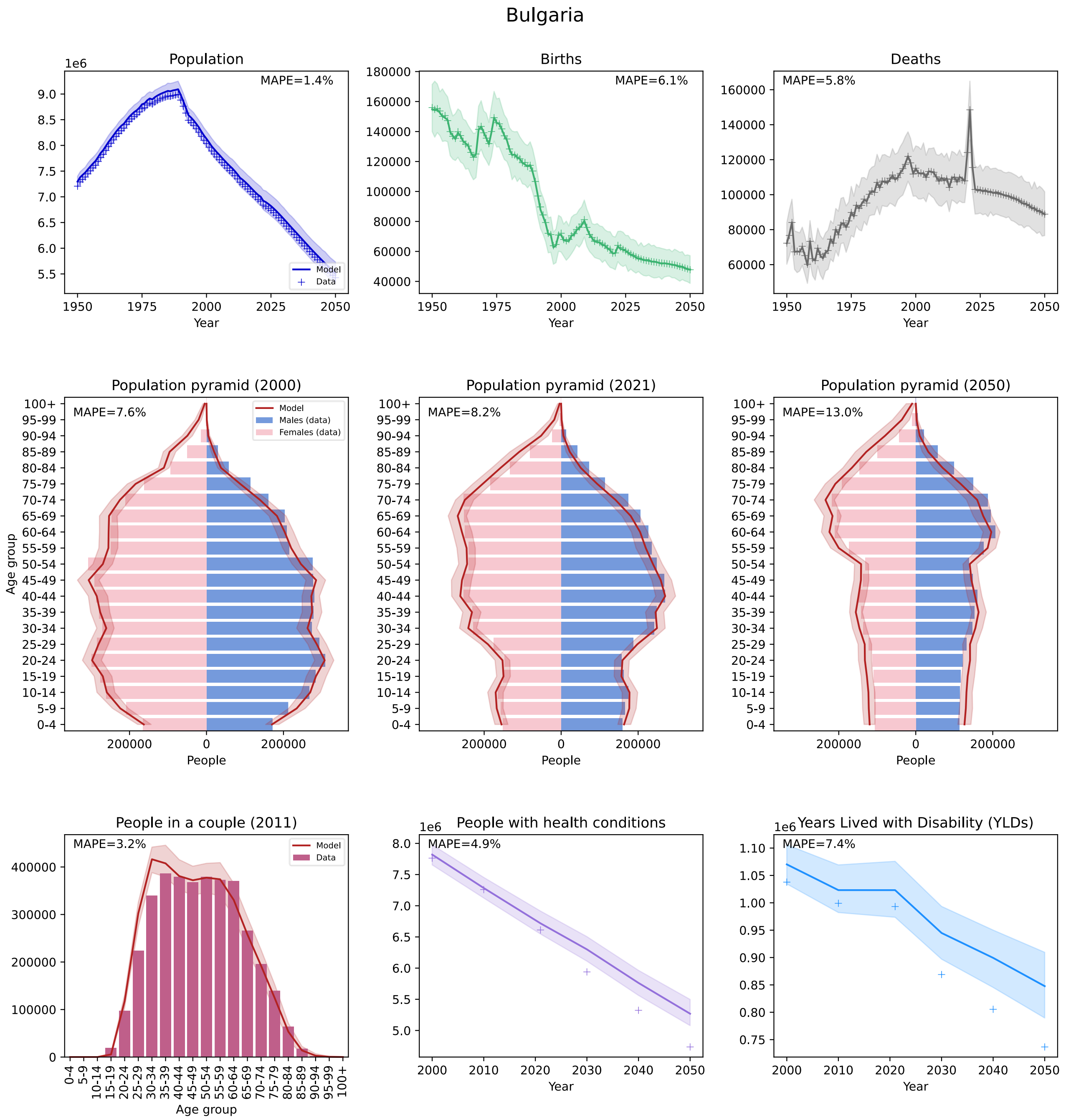
Bulgaria
Population
1e6
MAPE=1.4%
Model
Data
Year
Births
MAPE=6.1%
Year
Deaths
MAPE=5.8%
Year
Population pyramid (2000)
MAPE=7.6%
Model
Males (data)
Females (data)
Age group
People
Population pyramid (2021)
MAPE=8.2%
People
Population pyramid (2050)
MAPE=13.0%
People
People in a couple (2011)
MAPE=3.2%
Model
Data
Age group
People with health conditions
1e6
MAPE=4.9%
Year
Years Lived with Disability (YLDs)
1e6
MAPE=7.4%
Year

Croatia

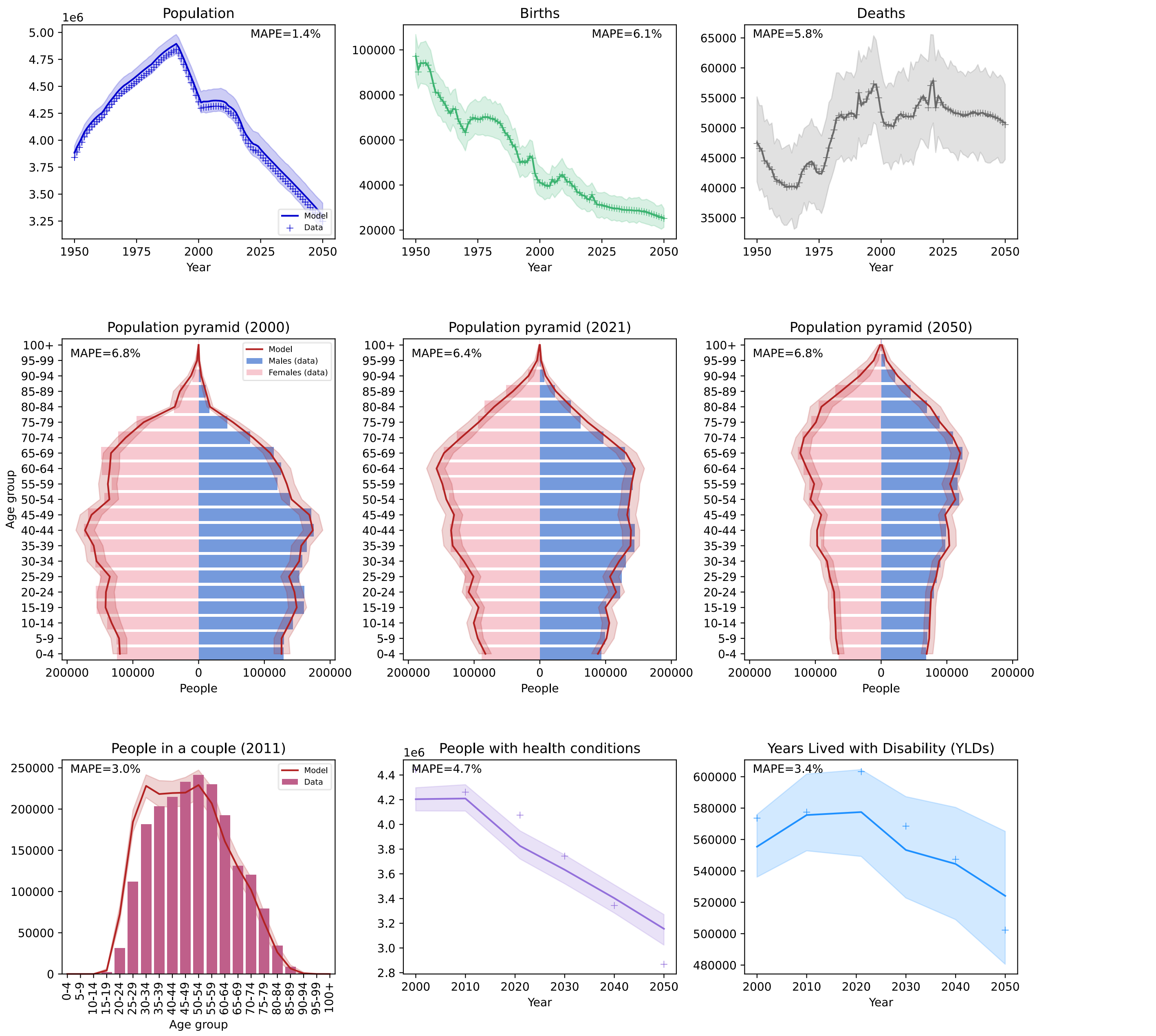
Population
1e6
MAPE=1.4%
Model
Data
Year
Births
MAPE=6.1%
Deaths
MAPE=5.8%
Population pyramid (2000)
MAPE=6.8%
Males (data)
Females (data)
Age group
People
Population pyramid (2021)
MAPE=6.4%
Population pyramid (2050)
MAPE=6.8%
People in a couple (2011)
MAPE=3.0%
People with health conditions
MAPE=4.7%
Years Lived with Disability (YLDs)
MAPE=3.4%

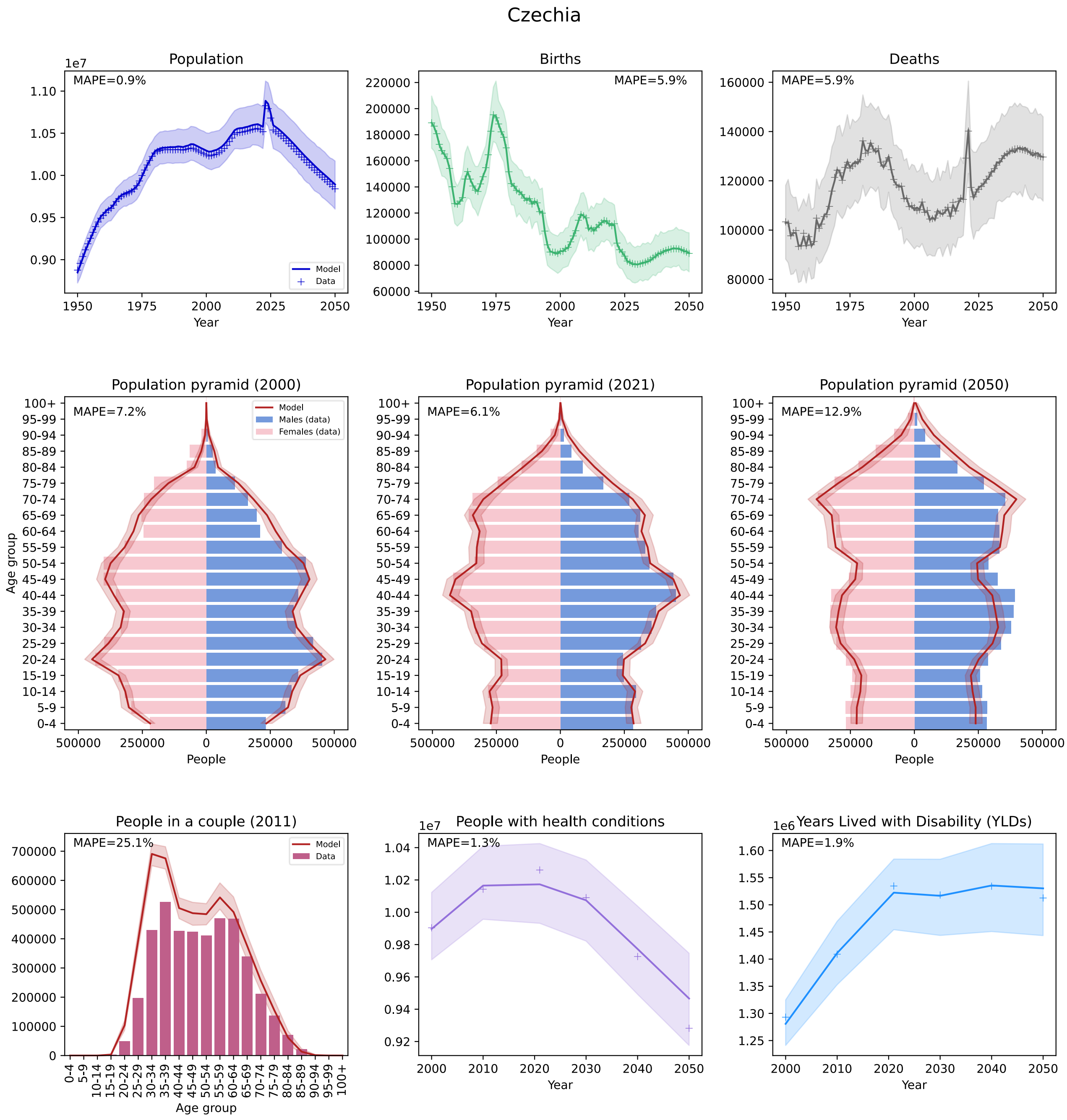
Czechia
Population
1e7
MAPE=0.9%
Model
Data
Year
Births
MAPE=5.9%
Year
Deaths
MAPE=5.9%
Year
Population pyramid (2000)
MAPE=7.2%
Model
Males (data)
Females (data)
Age group
People
Population pyramid (2021)
MAPE=6.1%
People
Population pyramid (2050)
MAPE=12.9%
People
People in a couple (2011)
MAPE=25.1%
Model
Data
Age group
People with health conditions
1e7
MAPE=1.3%
Year
Years Lived with Disability (YLDs)
1e6
MAPE=1.9%
Year

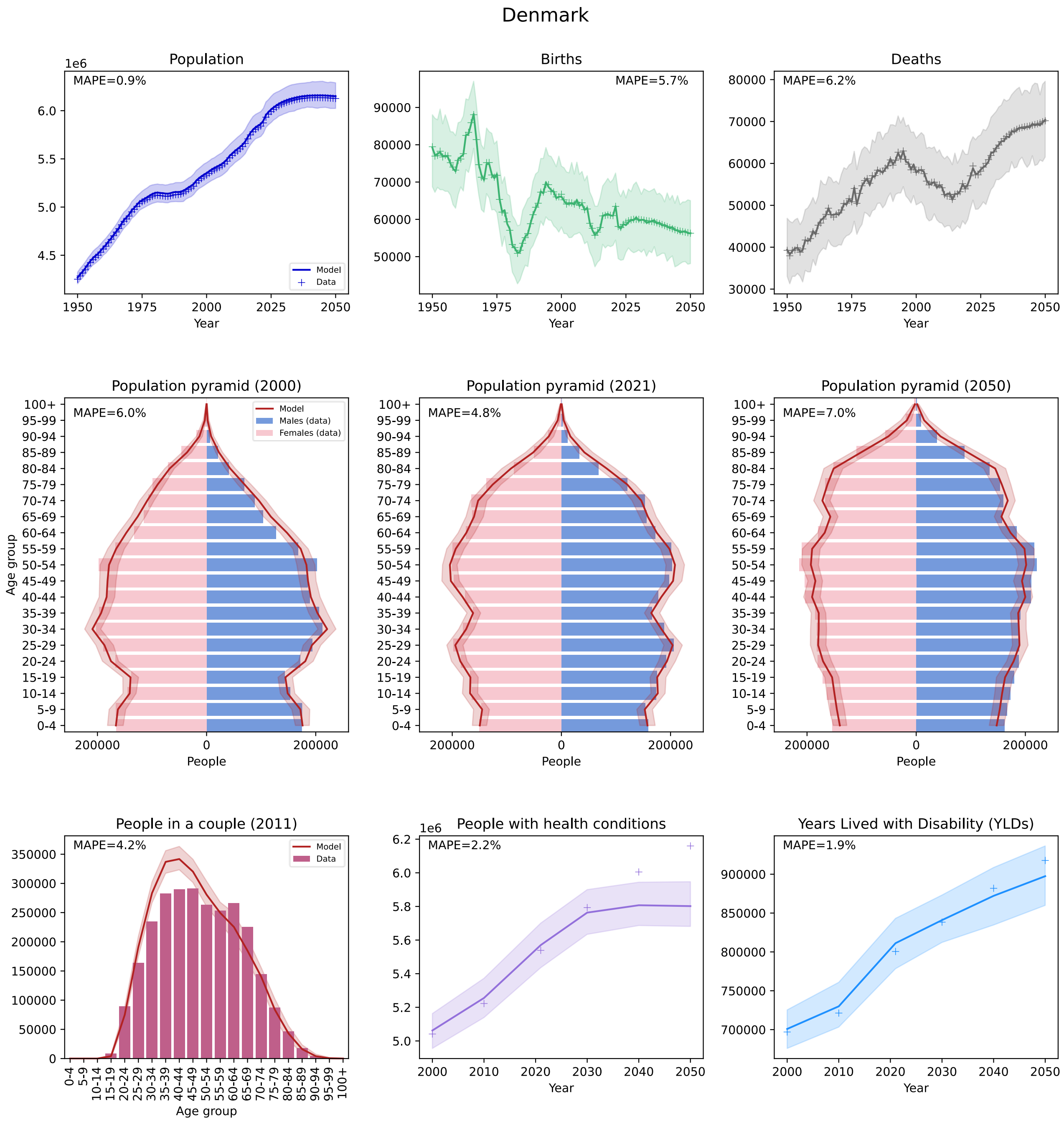

Denmark
Population
1e6
MAPE=0.9%
Model
Data
Year
Births
MAPE=5.7%
Deaths
MAPE=6.2%
Population pyramid (2000)
MAPE=6.0%
Model
Males (data)
Females (data)
Age group
People
Population pyramid (2021)
MAPE=4.8%
Population pyramid (2050)
MAPE=7.0%
People in a couple (2011)
MAPE=4.2%
Model
Data
Age group
People with health conditions
1e6
MAPE=2.2%
Years Lived with Disability (YLDs)
MAPE=1.9%

Estonia

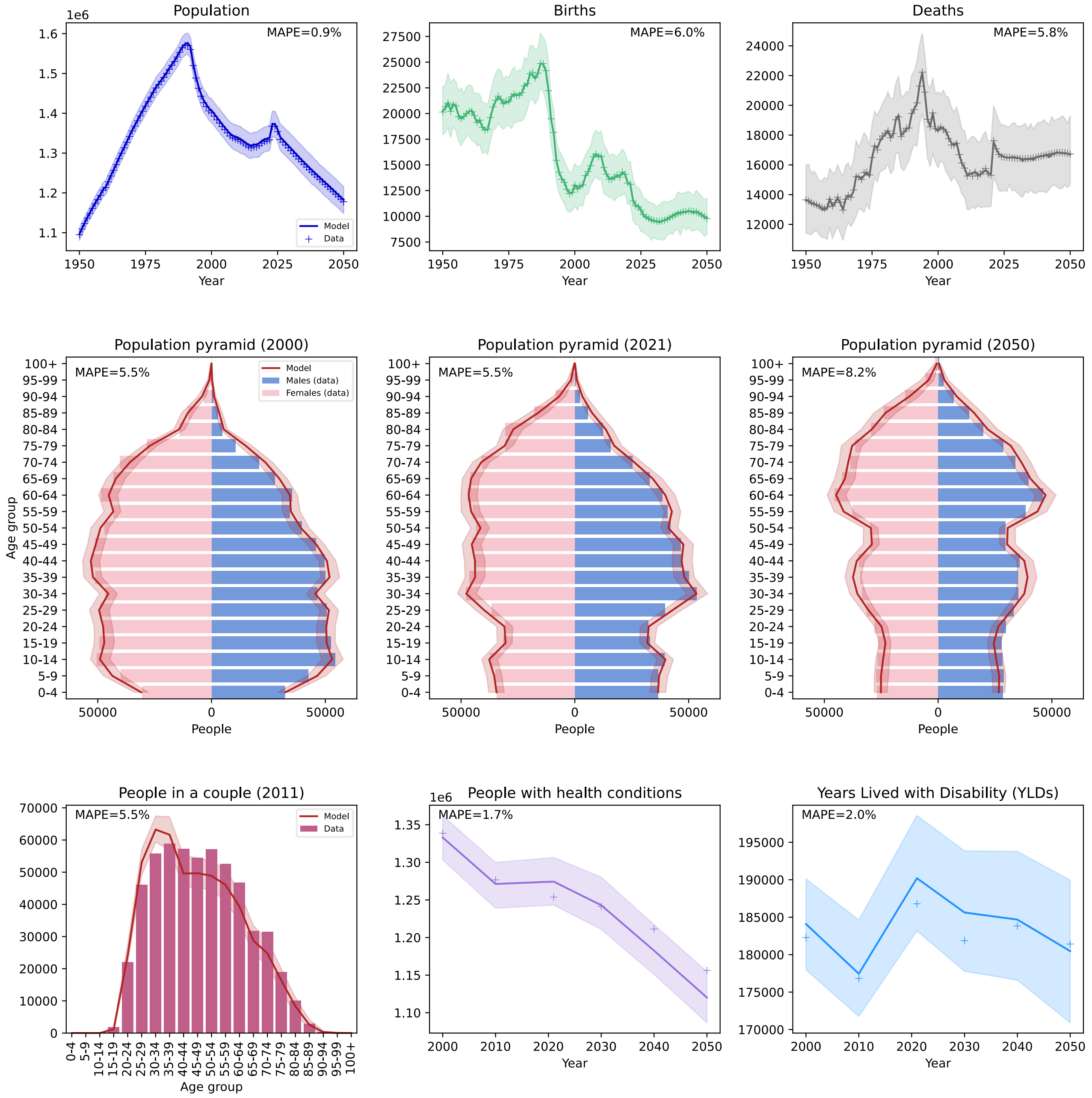
Population
1e6
MAPE=0.9%
Model
Data
Year
Births
MAPE=6.0%
Year
Deaths
MAPE=5.8%
Year
Population pyramid (2000)
MAPE=5.5%
Model
Males (data)
Females (data)
Age group
People
Population pyramid (2021)
MAPE=5.5%
People
Population pyramid (2050)
MAPE=8.2%
People
People in a couple (2011)
MAPE=5.5%
Model
Data
Age group
People with health conditions
1e6
MAPE=1.7%
Year
Years Lived with Disability (YLDs)
MAPE=2.0%
Year

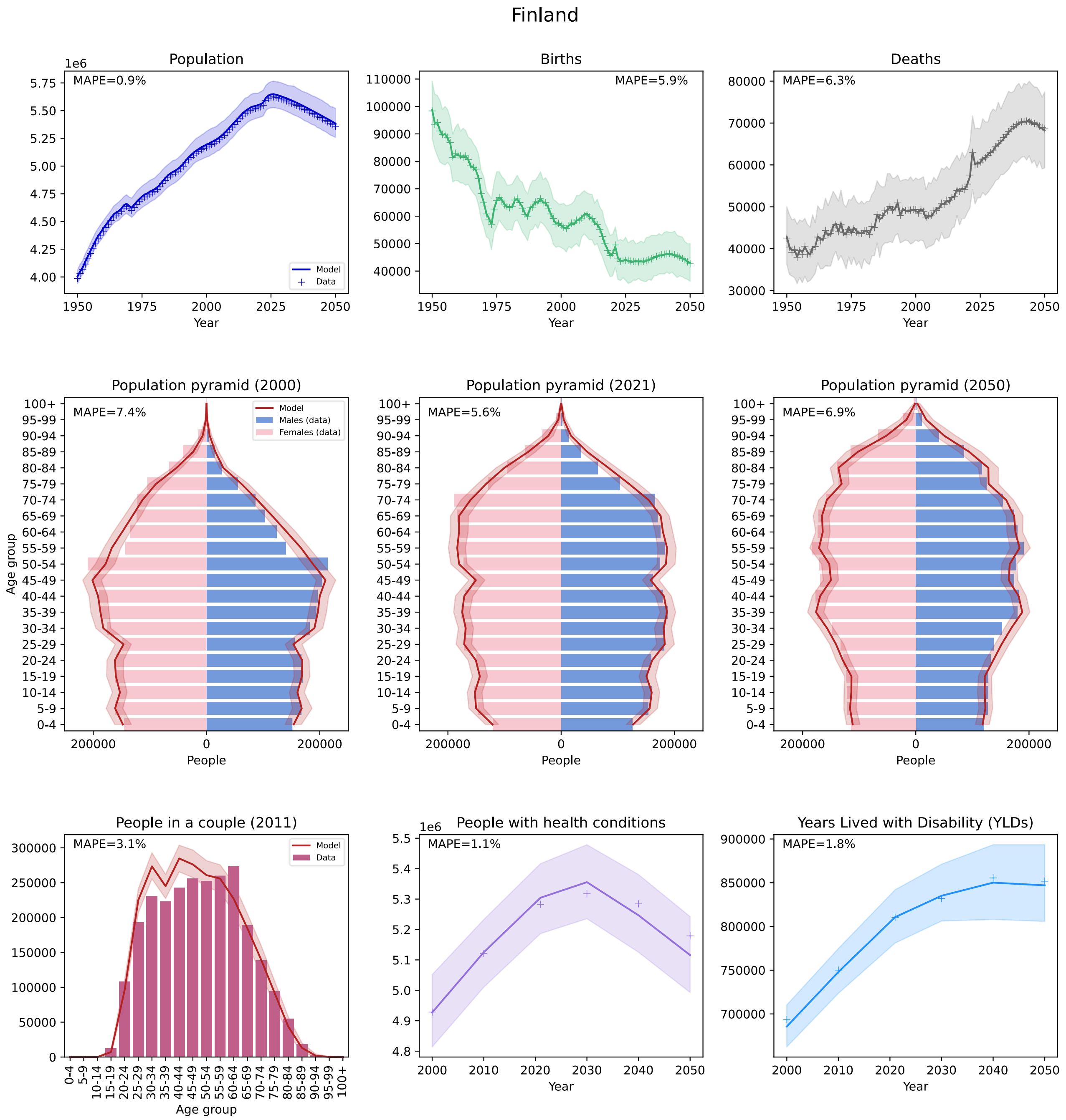
Finland
Population
1e6
MAPE=0.9%
Model
Data
Year
Births
MAPE=5.9%
Deaths
MAPE=6.3%
Population pyramid (2000)
MAPE=7.4%
Males (data)
Females (data)
Age group
People
Population pyramid (2021)
MAPE=5.6%
Population pyramid (2050)
MAPE=6.9%
People in a couple (2011)
MAPE=3.1%
Age group
People with health conditions
1e6
MAPE=1.1%
Years Lived with Disability (YLDs)
MAPE=1.8%

France

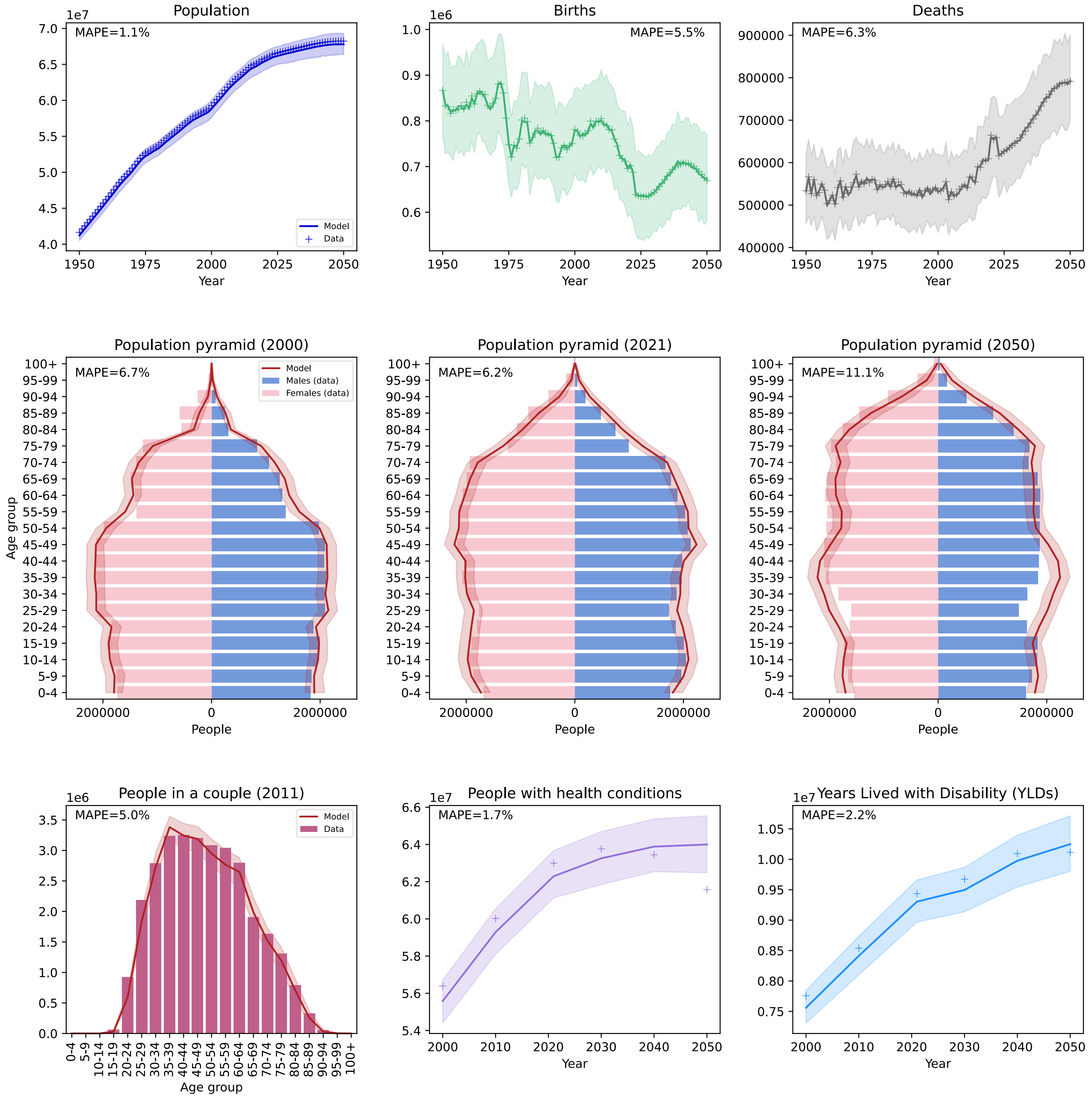

Population
MAPE=1.1%
Model
Data
Births
MAPE=5.5%
Deaths
MAPE=6.3%
Year
Population pyramid (2000)
MAPE=6.7%
Males (data)
Females (data)
Population pyramid (2021)
MAPE=6.2%
Population pyramid (2050)
MAPE=11.1%
Age group
People
People in a couple (2011)
MAPE=5.0%
People with health conditions
MAPE=1.7%
Years Lived with Disability (YLDs)
MAPE=2.2%

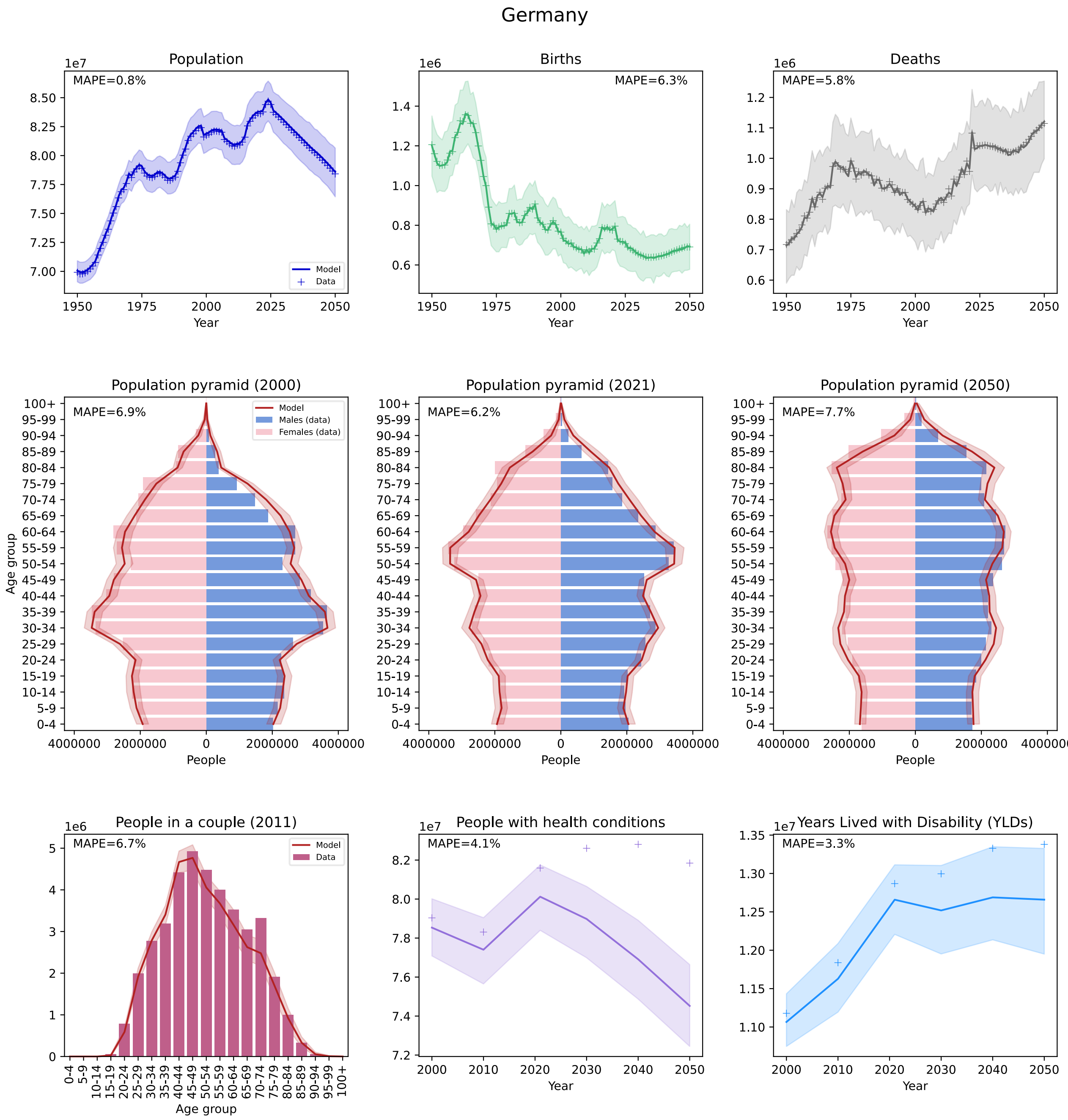
Germany
Population
MAPE=0.8%
Model
Data
Year
Births
MAPE=6.3%
Deaths
MAPE=5.8%
Population pyramid (2000)
MAPE=6.9%
Males (data)
Females (data)
Age group
People
Population pyramid (2021)
MAPE=6.2%
Population pyramid (2050)
MAPE=7.7%
People in a couple (2011)
MAPE=6.7%
People with health conditions
MAPE=4.1%
Years Lived with Disability (YLDs)
MAPE=3.3%

Greece

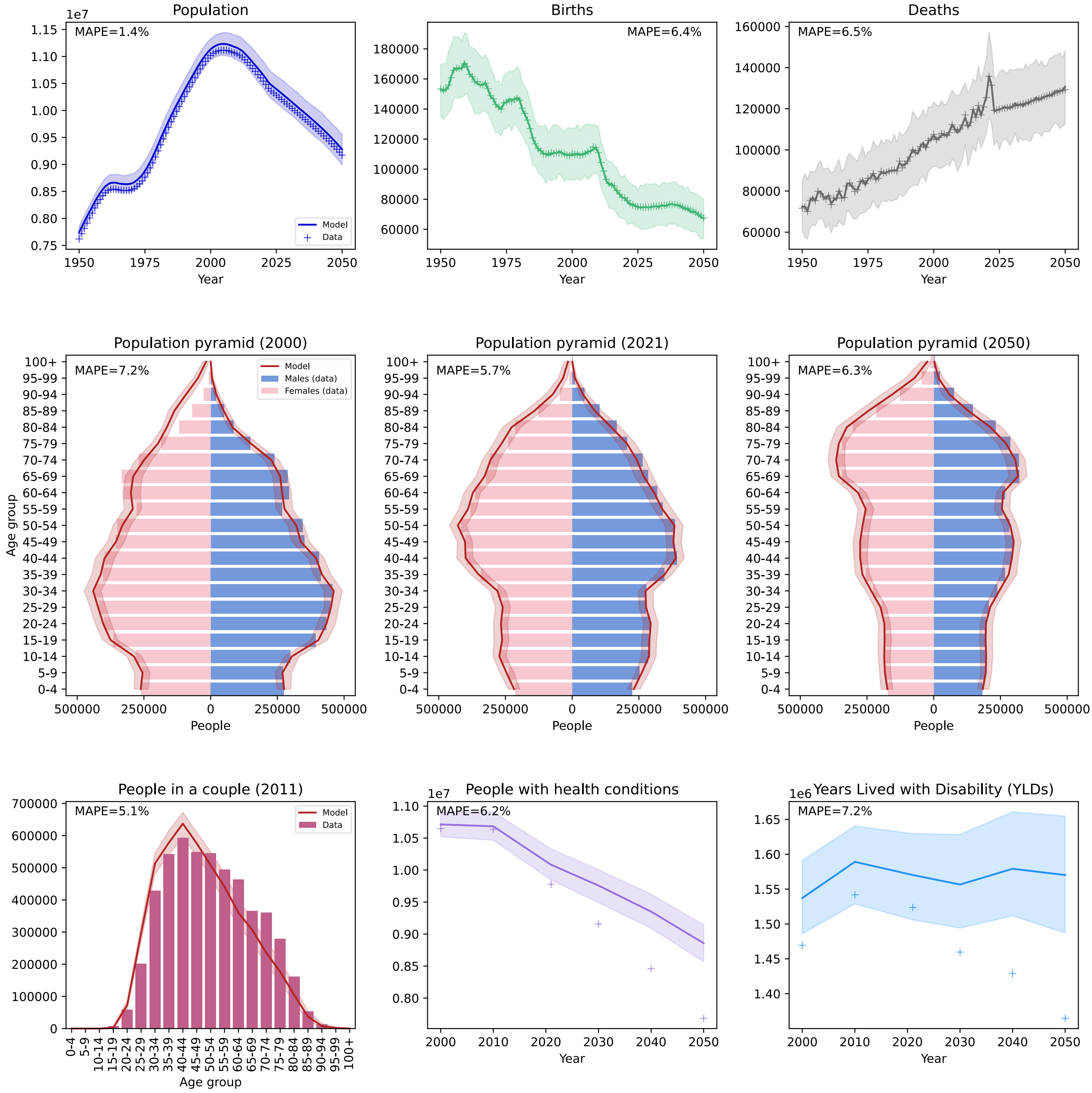
Population
MAPE=1.4%
1e7
Model
Data
Year
Births
MAPE=6.4%
Year
Deaths
MAPE=6.5%
Year
Population pyramid (2000)
MAPE=7.2%
Model
Males (data)
Females (data)
Age group
People
Population pyramid (2021)
MAPE=5.7%
People
Population pyramid (2050)
MAPE=6.3%
People
People in a couple (2011)
MAPE=5.1%
Model
Data
Age group
People with health conditions
1e7
MAPE=6.2%
Year
Years Lived with Disability (YLDs)
1e6
MAPE=7.2%
Year

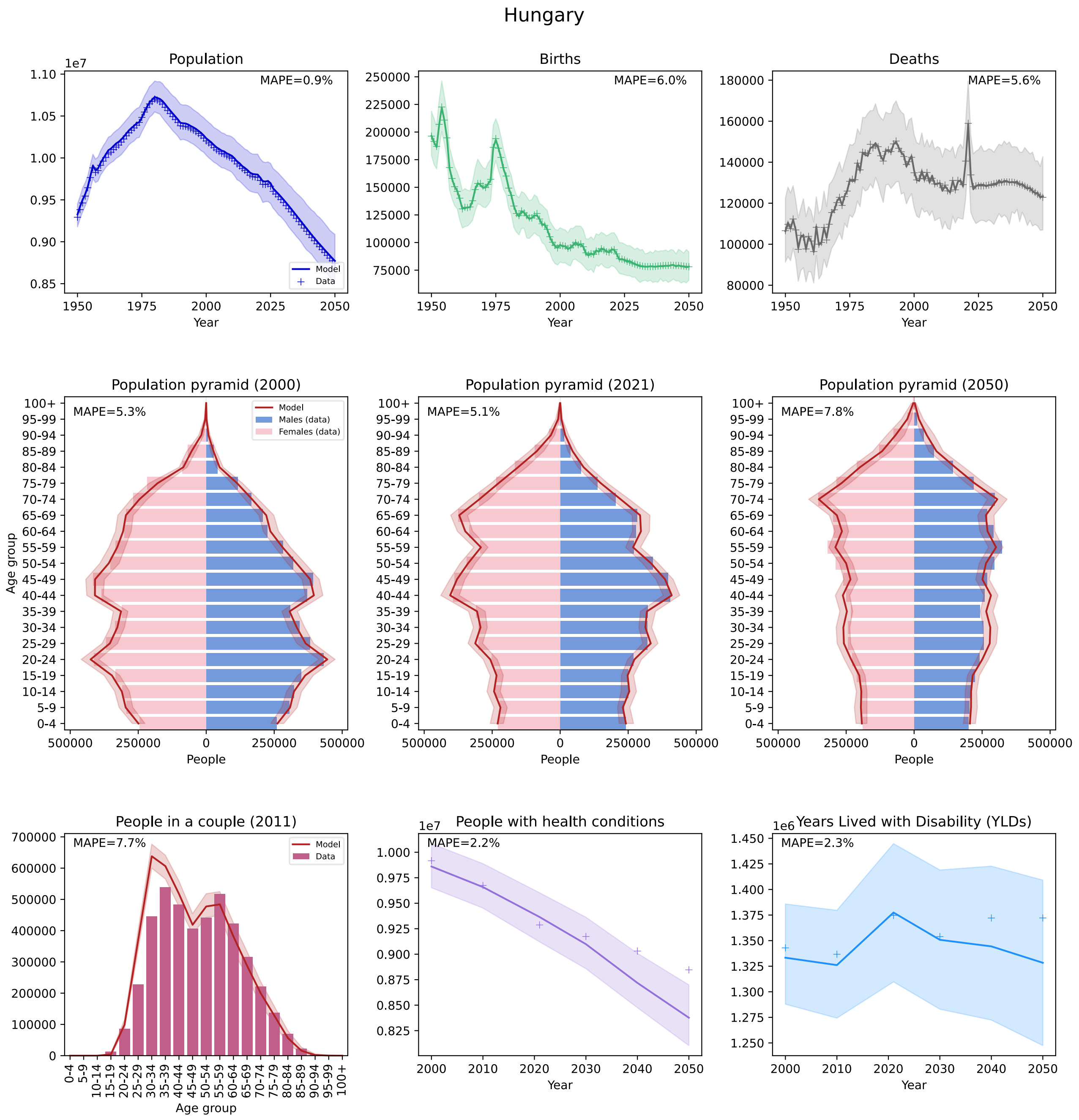
Hungary
Population
1e7
MAPE=0.9%
Model
Data
Year
Births
MAPE=6.0%
Year
Deaths
MAPE=5.6%
Year
Population pyramid (2000)
MAPE=5.3%
Model
Males (data)
Females (data)
Age group
People
Population pyramid (2021)
MAPE=5.1%
People
Population pyramid (2050)
MAPE=7.8%
People
People in a couple (2011)
MAPE=7.7%
Model
Data
Age group
People with health conditions
1e7
MAPE=2.2%
Year
Years Lived with Disability (YLDs)
1e6
MAPE=2.3%
Year

Iceland

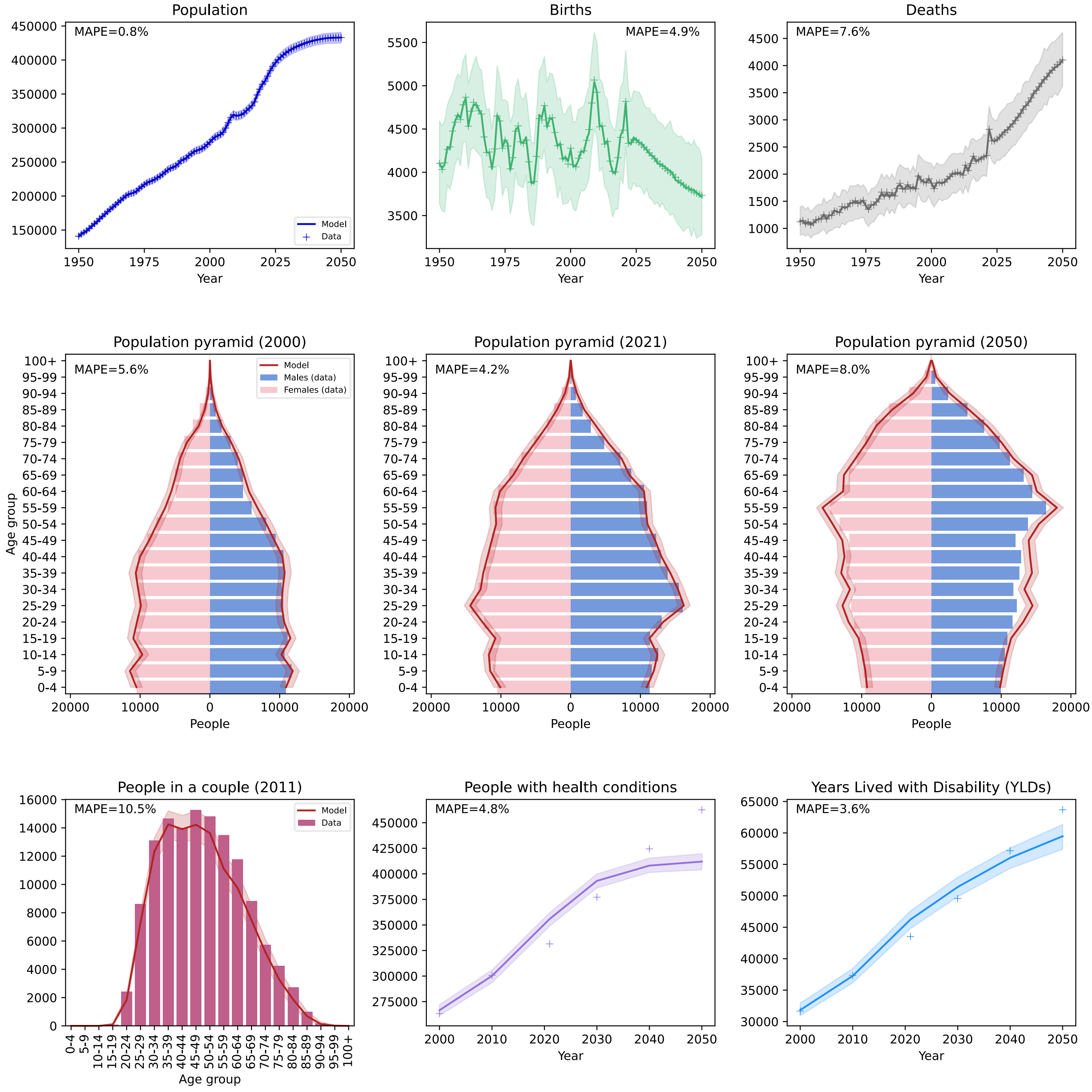
Population
MAPE=0.8%
Model
Data
Year
Births
MAPE=4.9%
Deaths
MAPE=7.6%
Population pyramid (2000)
MAPE=5.6%
Males (data)
Females (data)
Age group
People
Population pyramid (2021)
MAPE=4.2%
Population pyramid (2050)
MAPE=8.0%
People in a couple (2011)
MAPE=10.5%
People with health conditions
MAPE=4.8%
Years Lived with Disability (YLDs)
MAPE=3.6%

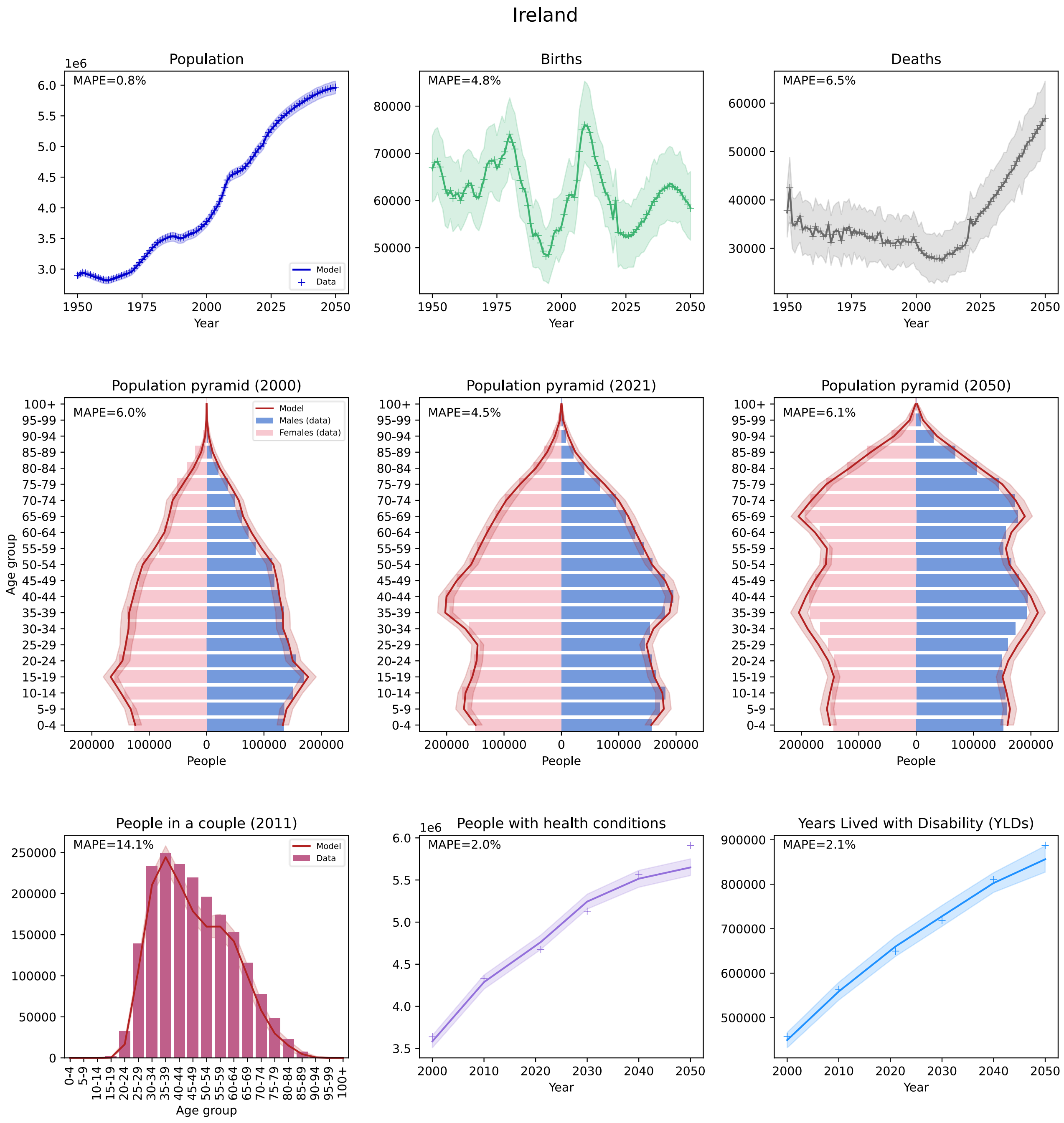
Ireland
Population
1e6
MAPE=0.8%
Model
Data
Year
Births
MAPE=4.8%
Year
Deaths
MAPE=6.5%
Year
Population pyramid (2000)
MAPE=6.0%
Model
Males (data)
Females (data)
Age group
People
Population pyramid (2021)
MAPE=4.5%
People
Population pyramid (2050)
MAPE=6.1%
People
People in a couple (2011)
MAPE=14.1%
Model
Data
Age group
People with health conditions
1e6
MAPE=2.0%
Year
Years Lived with Disability (YLDs)
MAPE=2.1%
Year

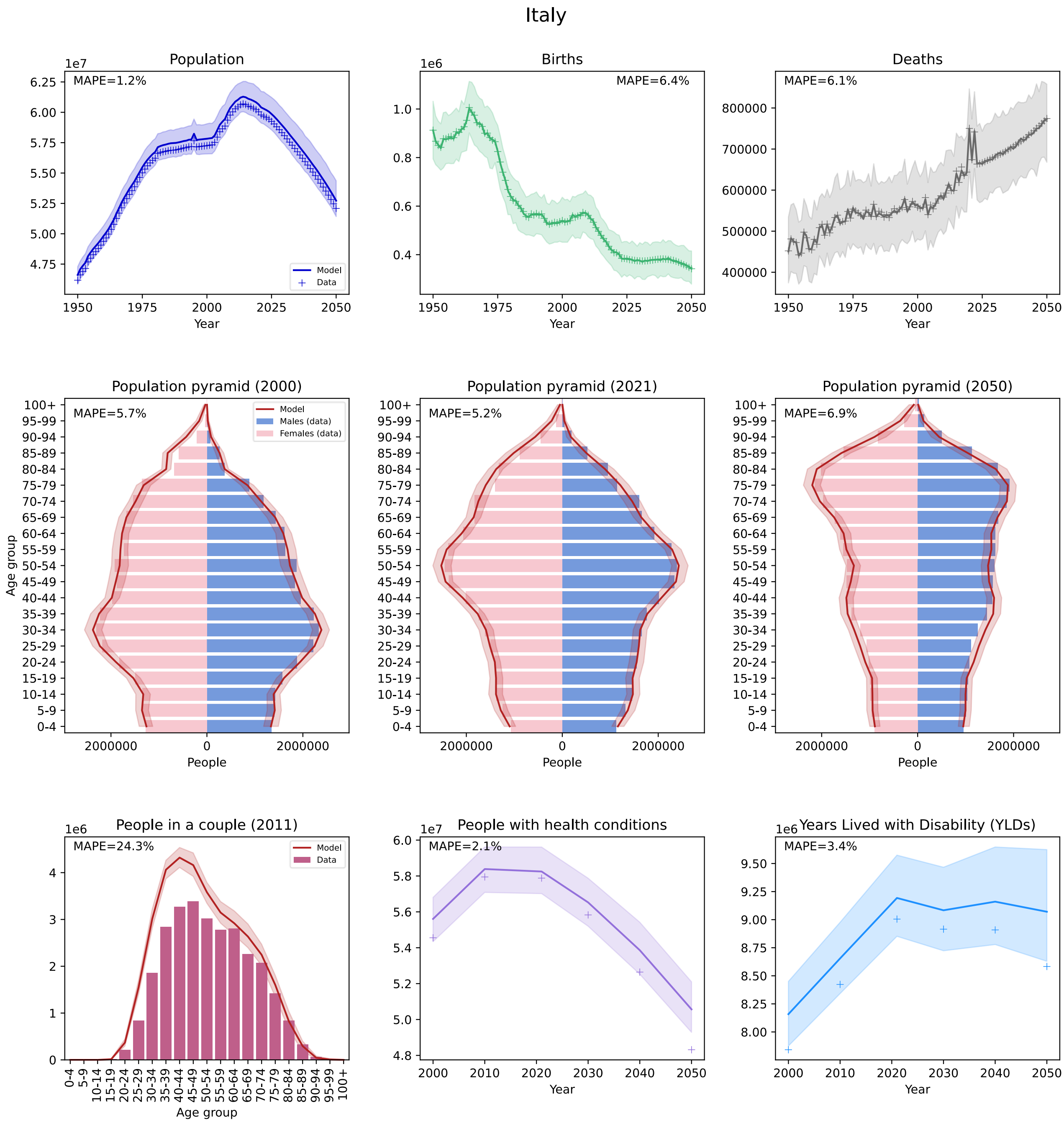
Italy
Population
1e7
MAPE=1.2%
Model
Data
Year
Births
1e6
MAPE=6.4%
Year
Deaths
MAPE=6.1%
Year
Population pyramid (2000)
MAPE=5.7%
Model
Males (data)
Females (data)
Age group
People
Population pyramid (2021)
MAPE=5.2%
People
Population pyramid (2050)
MAPE=6.9%
People
People in a couple (2011)
1e6
MAPE=24.3%
Model
Data
Age group
People with health conditions
1e7
MAPE=2.1%
Year
1e6
Years Lived with Disability (YLDs)
MAPE=3.4%
Year

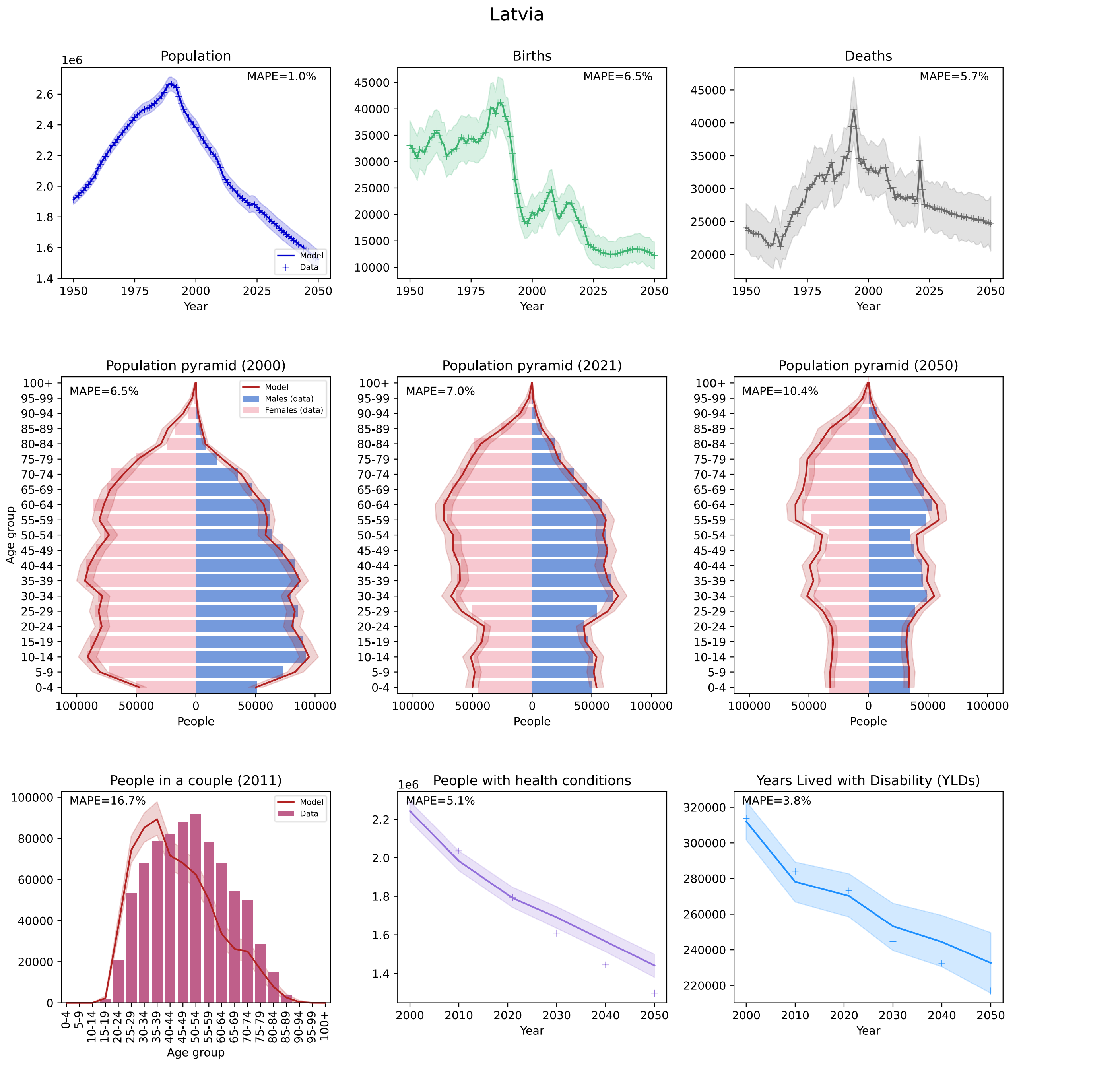
Latvia
Population
1e6
MAPE=1.0%
Model
Data
Year
Births
MAPE=6.5%
Year
Deaths
MAPE=5.7%
Year
Population pyramid (2000)
MAPE=6.5%
Model
Males (data)
Females (data)
Age group
People
Population pyramid (2021)
MAPE=7.0%
People
Population pyramid (2050)
MAPE=10.4%
People
People in a couple (2011)
MAPE=16.7%
Model
Data
Age group
People with health conditions
1e6
MAPE=5.1%
Year
Years Lived with Disability (YLDs)
MAPE=3.8%
Year

Lithuania

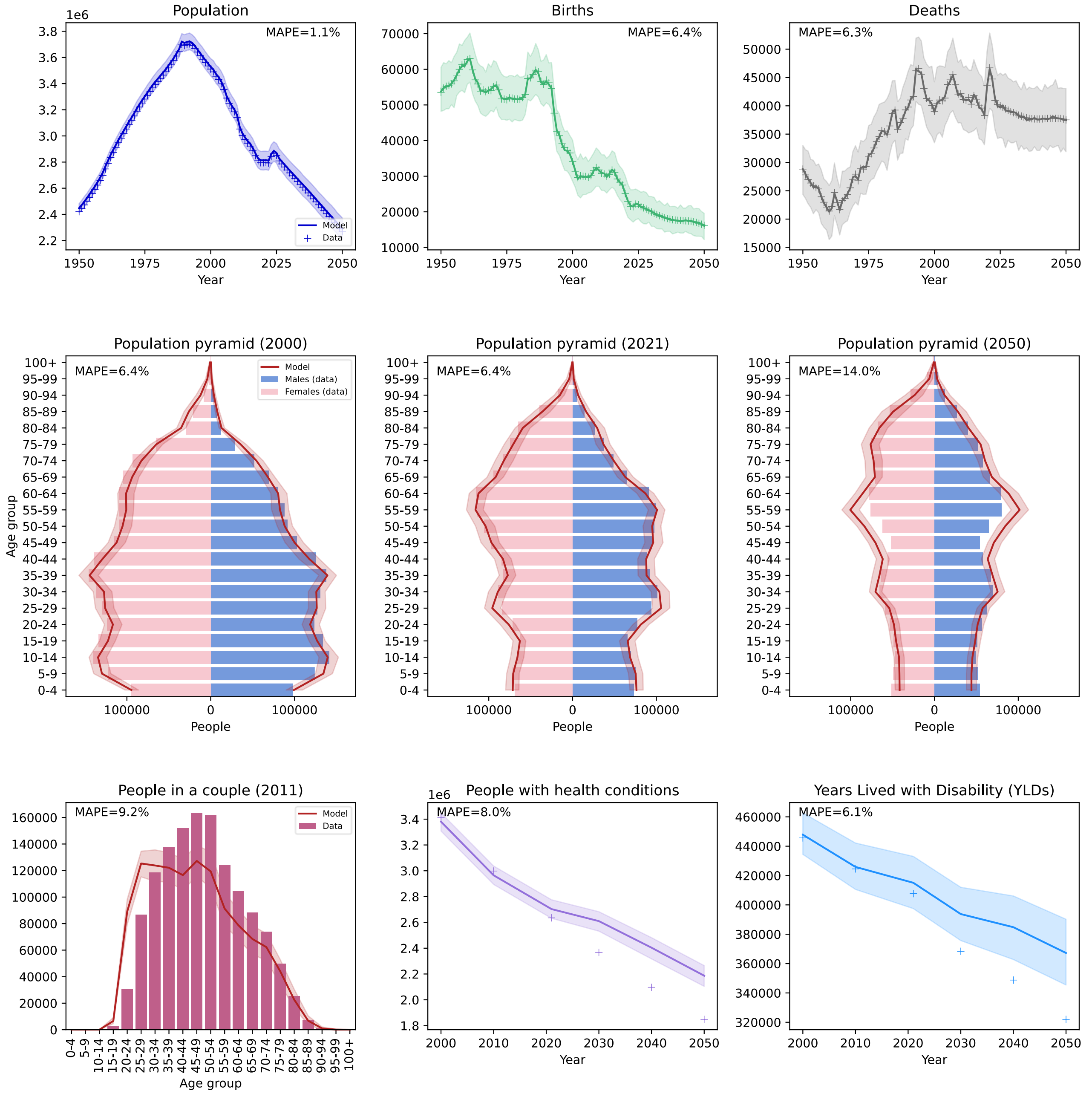

Population
1e6
MAPE=1.1%
Model
Data
Year
Births
MAPE=6.4%
Year
Deaths
MAPE=6.3%
Year
Population pyramid (2000)
MAPE=6.4%
Model
Males (data)
Females (data)
Age group
People
Population pyramid (2021)
MAPE=6.4%
People
Population pyramid (2050)
MAPE=14.0%
People
People in a couple (2011)
MAPE=9.2%
Model
Data
Age group
People with health conditions
1e6
MAPE=8.0%
Year
Years Lived with Disability (YLDs)
MAPE=6.1%
Year

Malta

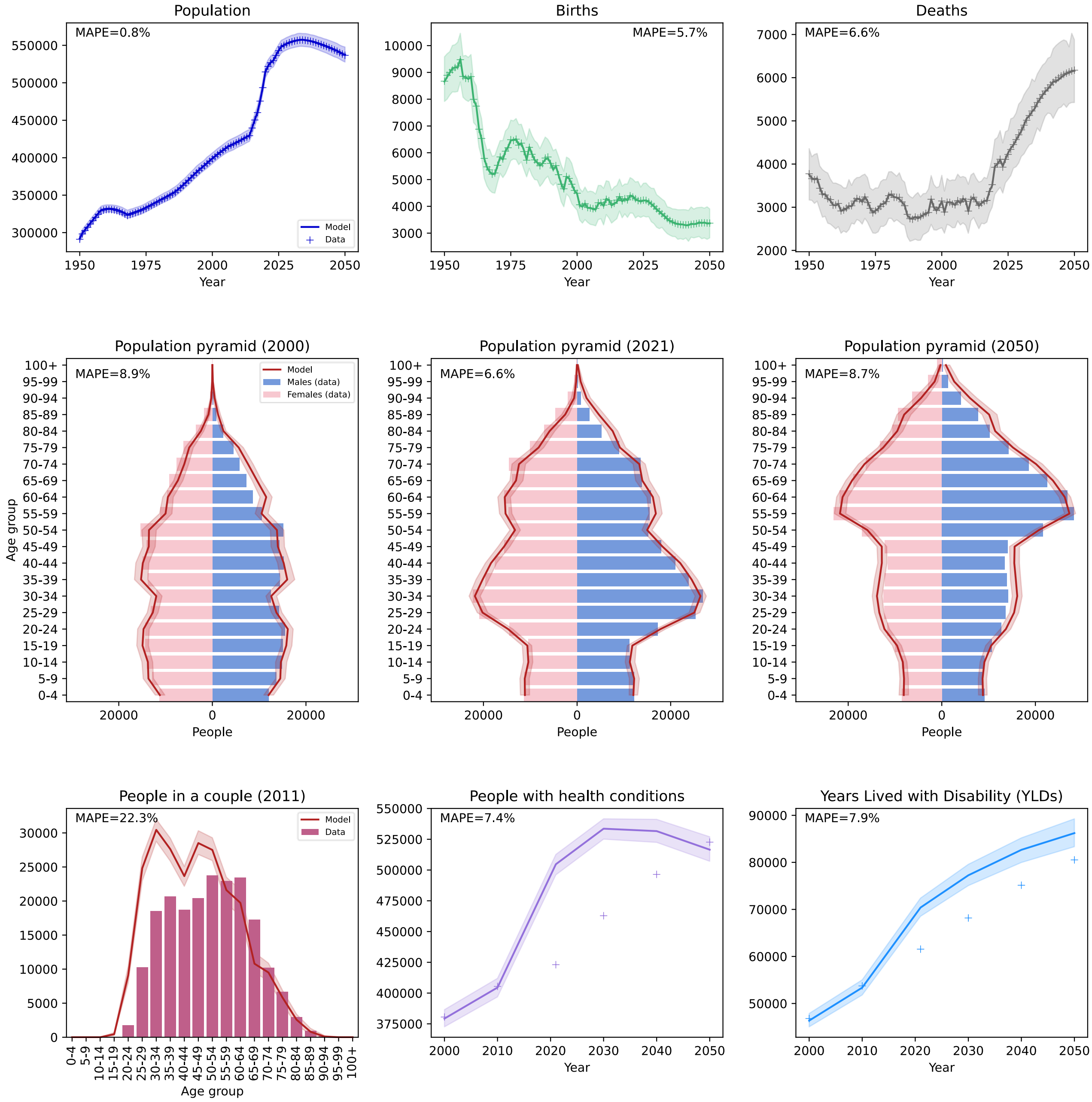

Population
MAPE=0.8%
Model
Data
Year
Births
MAPE=5.7%
Deaths
MAPE=6.6%
Population pyramid (2000)
MAPE=8.9%
Males (data)
Females (data)
Age group
People
Population pyramid (2021)
MAPE=6.6%
Population pyramid (2050)
MAPE=8.7%
People in a couple (2011)
MAPE=22.3%
People with health conditions
MAPE=7.4%
Years Lived with Disability (YLDs)
MAPE=7.9%

Montenegro

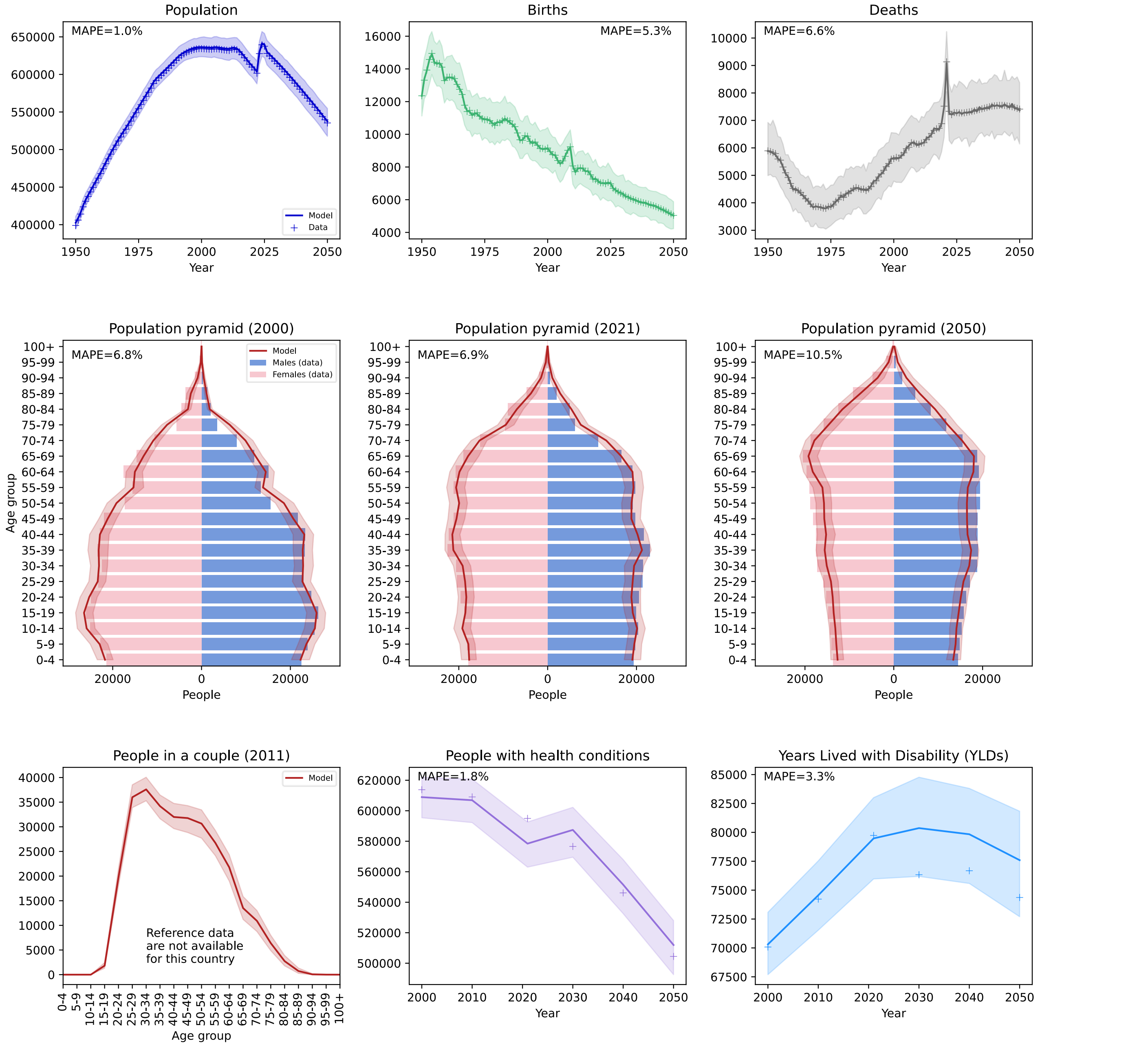

Population
MAPE=1.0%
Model
Data
Year
Births
MAPE=5.3%
Year
Deaths
MAPE=6.6%
Year
Population pyramid (2000)
MAPE=6.8%
Model
Males (data)
Females (data)
Age group
People
Population pyramid (2021)
MAPE=6.9%
People
Population pyramid (2050)
MAPE=10.5%
People
People in a couple (2011)
Model
Reference data
are not available
for this country
Age group
People with health conditions
MAPE=1.8%
Year
Years Lived with Disability (YLDs)
MAPE=3.3%
Year

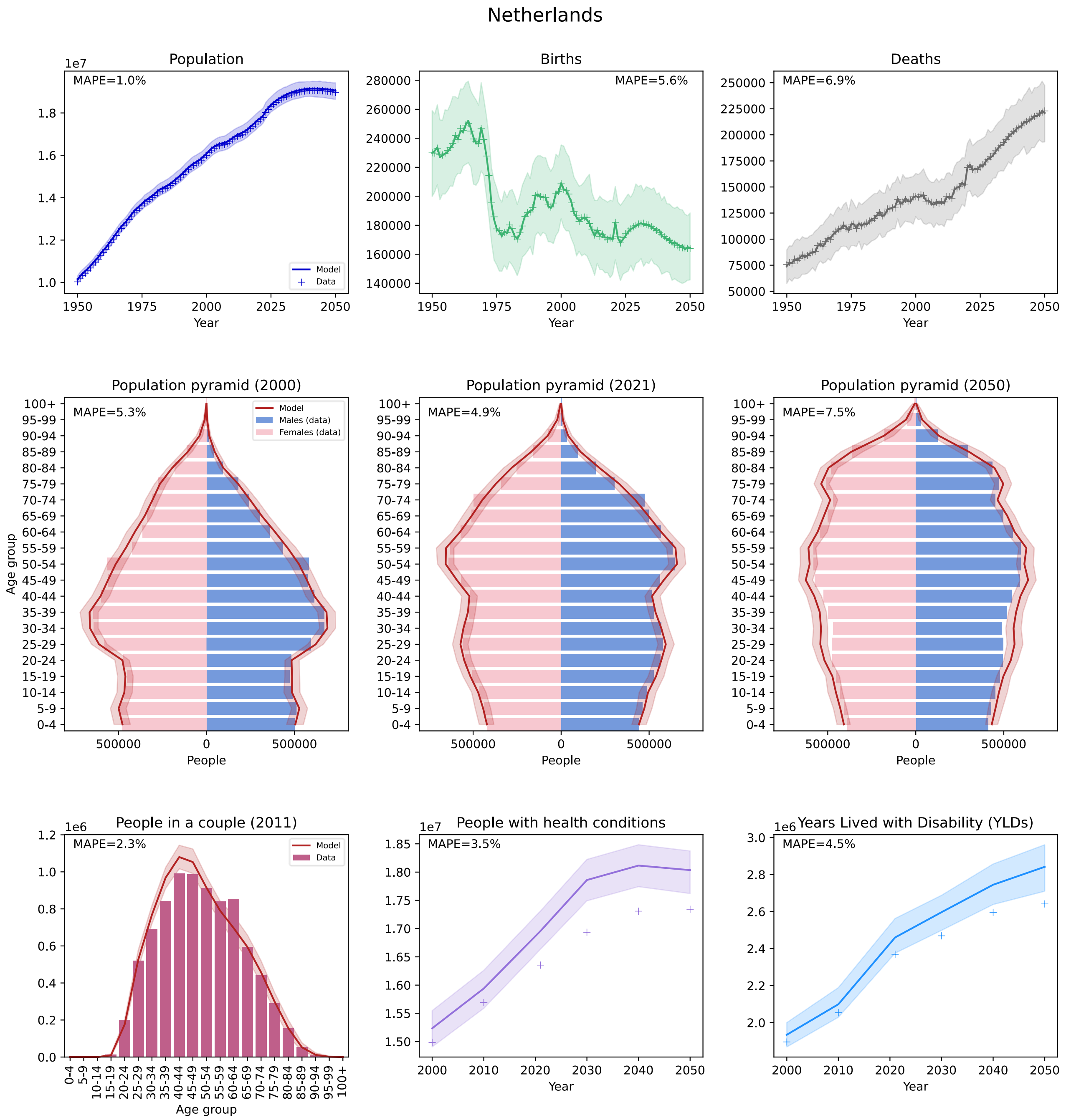
Netherlands
Population
1e7
MAPE=1.0%
Model
Data
Year
Births
MAPE=5.6%
Year
Deaths
MAPE=6.9%
Year
Population pyramid (2000)
MAPE=5.3%
Model
Males (data)
Females (data)
Age group
People
Population pyramid (2021)
MAPE=4.9%
People
Population pyramid (2050)
MAPE=7.5%
People
People in a couple (2011)
1e6
MAPE=2.3%
Model
Data
Age group
People with health conditions
1e7
MAPE=3.5%
Year
Years Lived with Disability (YLDs)
1e6
MAPE=4.5%
Year

North Macedonia

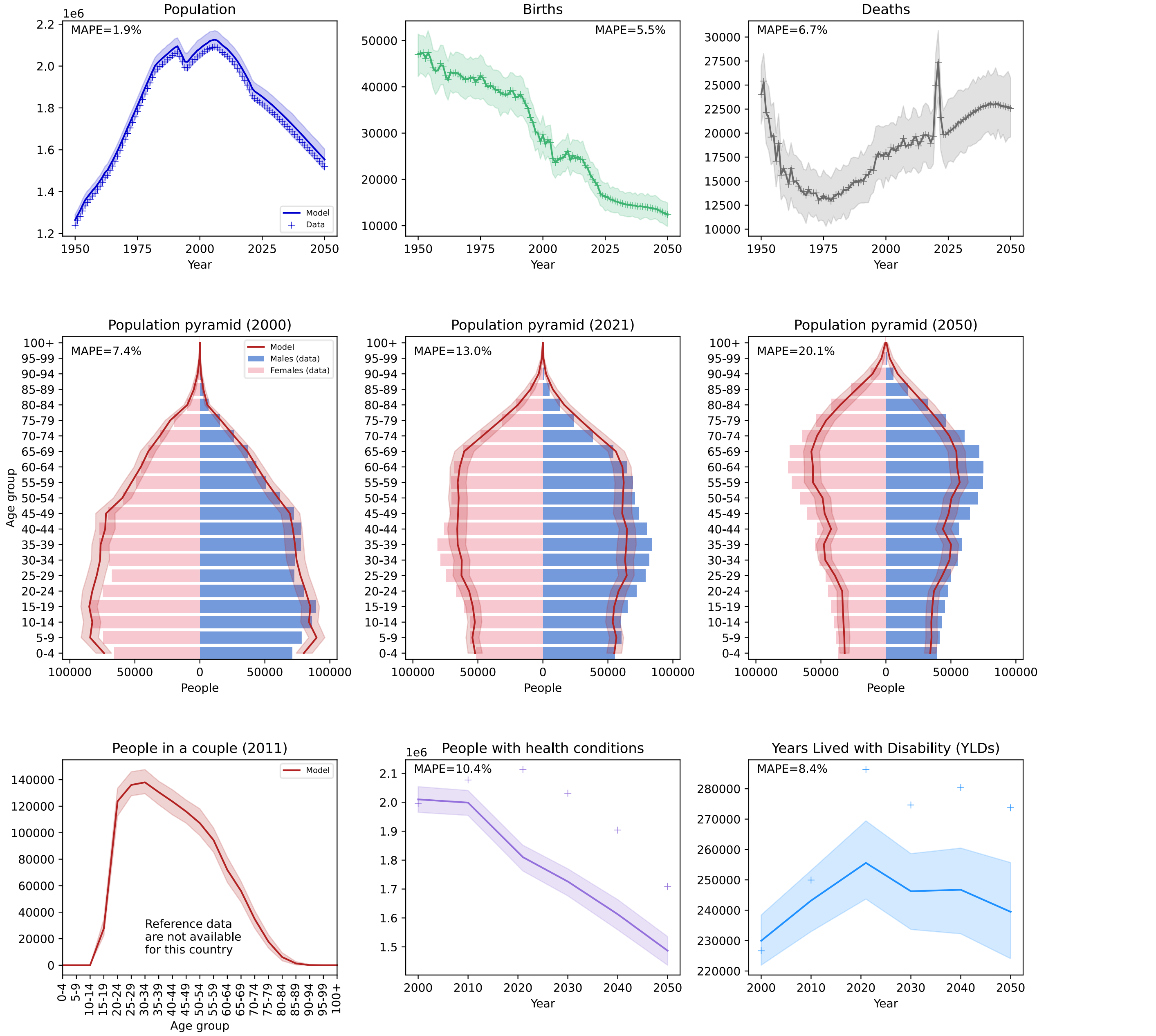

Norway

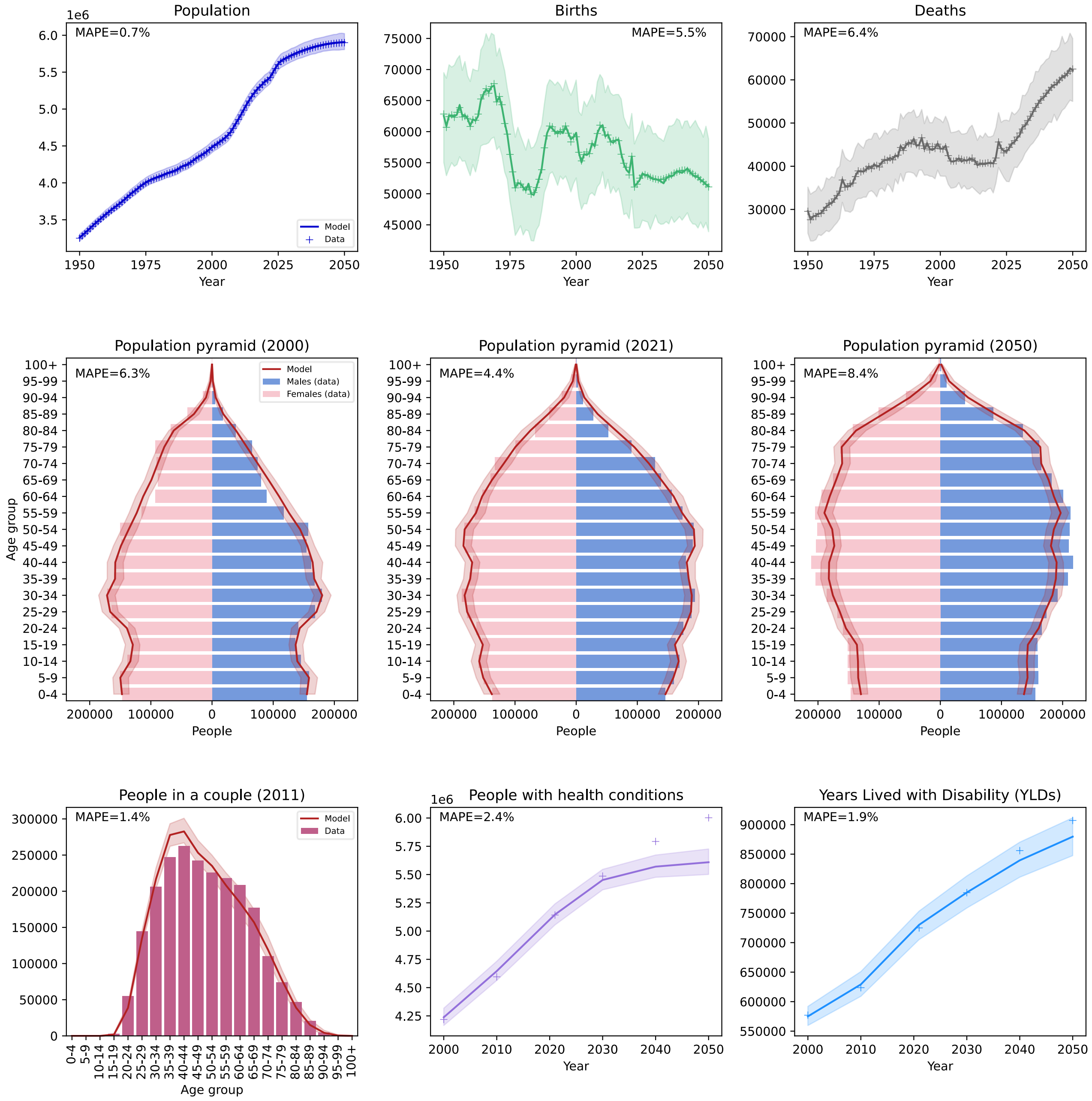
Population
1e6
MAPE=0.7%
Model
Data
Year
Births
MAPE=5.5%
Year
Deaths
MAPE=6.4%
Year
Population pyramid (2000)
MAPE=6.3%
Model
Males (data)
Females (data)
Age group
People
Population pyramid (2021)
MAPE=4.4%
People
Population pyramid (2050)
MAPE=8.4%
People
People in a couple (2011)
MAPE=1.4%
Model
Data
Age group
People with health conditions
1e6
MAPE=2.4%
Year
Years Lived with Disability (YLDs)
MAPE=1.9%
Year

Poland

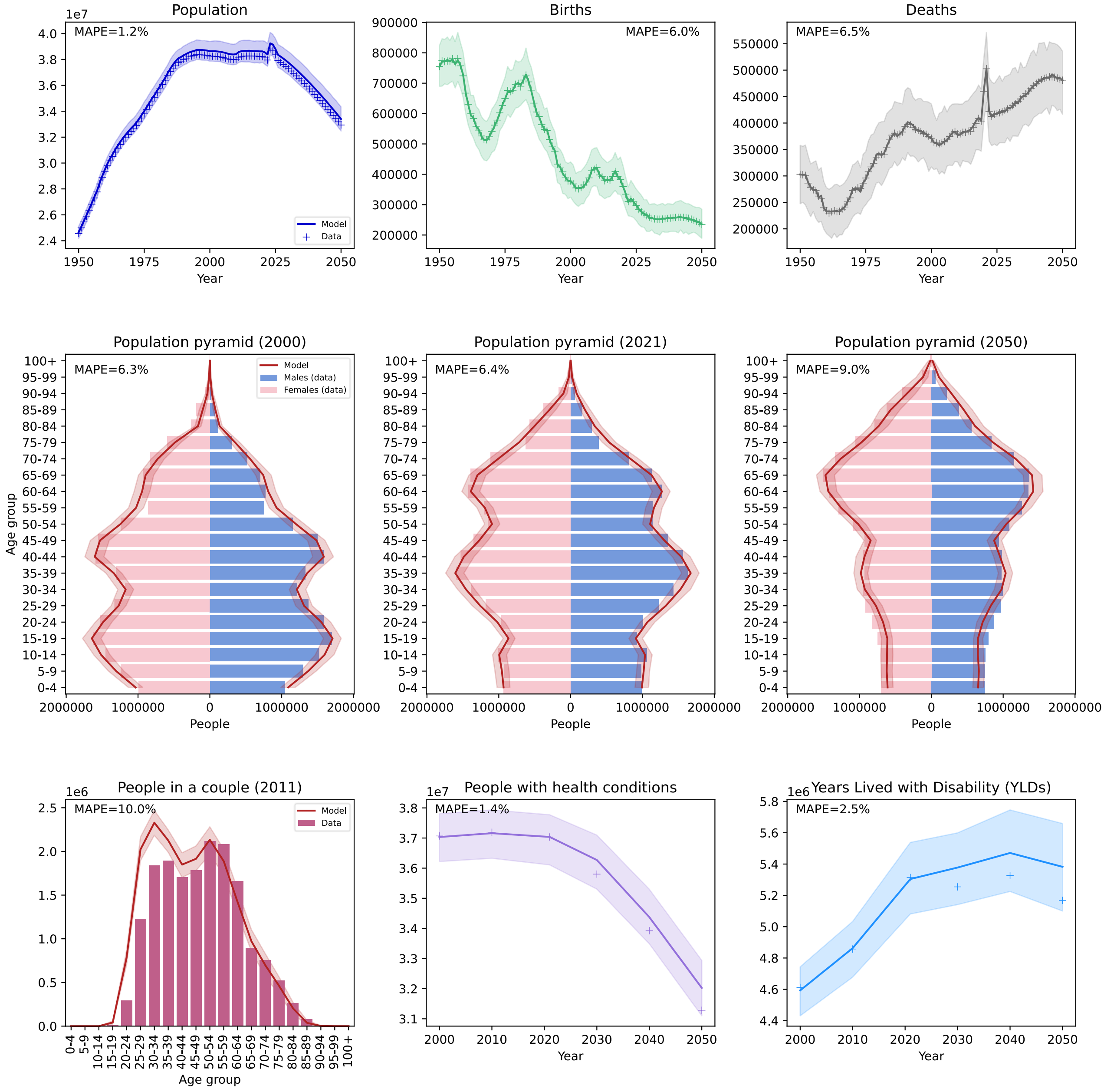
Population
MAPE=1.2%
Model
Data
Year
Births
MAPE=6.0%
Deaths
MAPE=6.5%
Population pyramid (2000)
MAPE=6.3%
Males (data)
Females (data)
Age group
People
Population pyramid (2021)
MAPE=6.4%
Population pyramid (2050)
MAPE=9.0%
People in a couple (2011)
MAPE=10.0%
People with health conditions
MAPE=1.4%
Years Lived with Disability (YLDs)
MAPE=2.5%

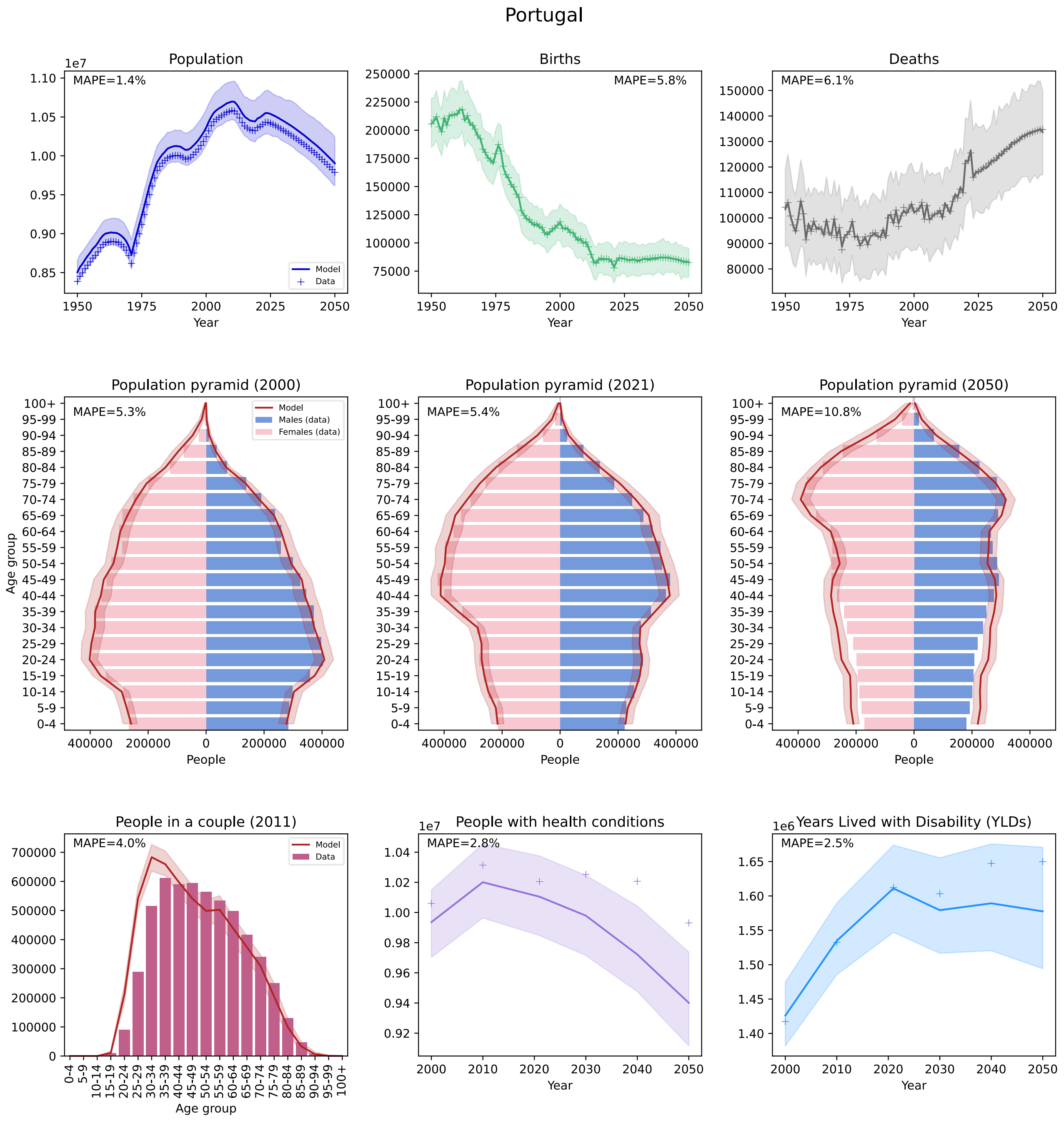
Portugal
Population
MAPE=1.4%
Model
Data
Births
MAPE=5.8%
Deaths
MAPE=6.1%
Year
Population pyramid (2000)
MAPE=5.3%
Males (data)
Females (data)
Population pyramid (2021)
MAPE=5.4%
Population pyramid (2050)
MAPE=10.8%
Age group
People
People in a couple (2011)
MAPE=4.0%
People with health conditions
MAPE=2.8%
Years Lived with Disability (YLDs)
MAPE=2.5%

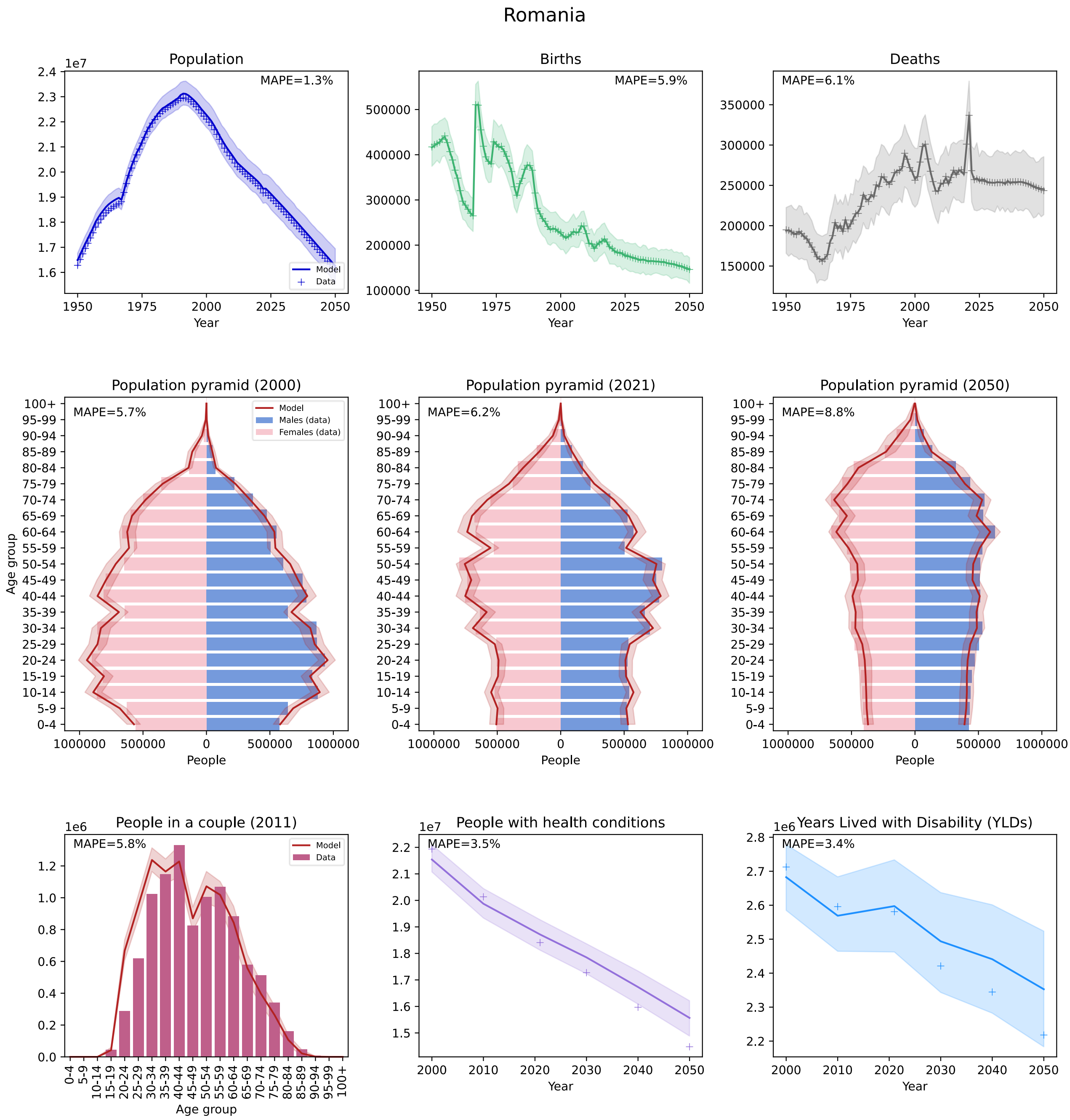
Romania
Population
MAPE=1.3%
1e7
Model
Data
Year
Births
MAPE=5.9%
Year
Deaths
MAPE=6.1%
Year
Population pyramid (2000)
MAPE=5.7%
Model
Males (data)
Females (data)
Age group
People
Population pyramid (2021)
MAPE=6.2%
People
Population pyramid (2050)
MAPE=8.8%
People
People in a couple (2011)
MAPE=5.8%
1e6
Model
Data
Age group
People with health conditions
MAPE=3.5%
1e7
Year
Years Lived with Disability (YLDs)
MAPE=3.4%
1e6
Year

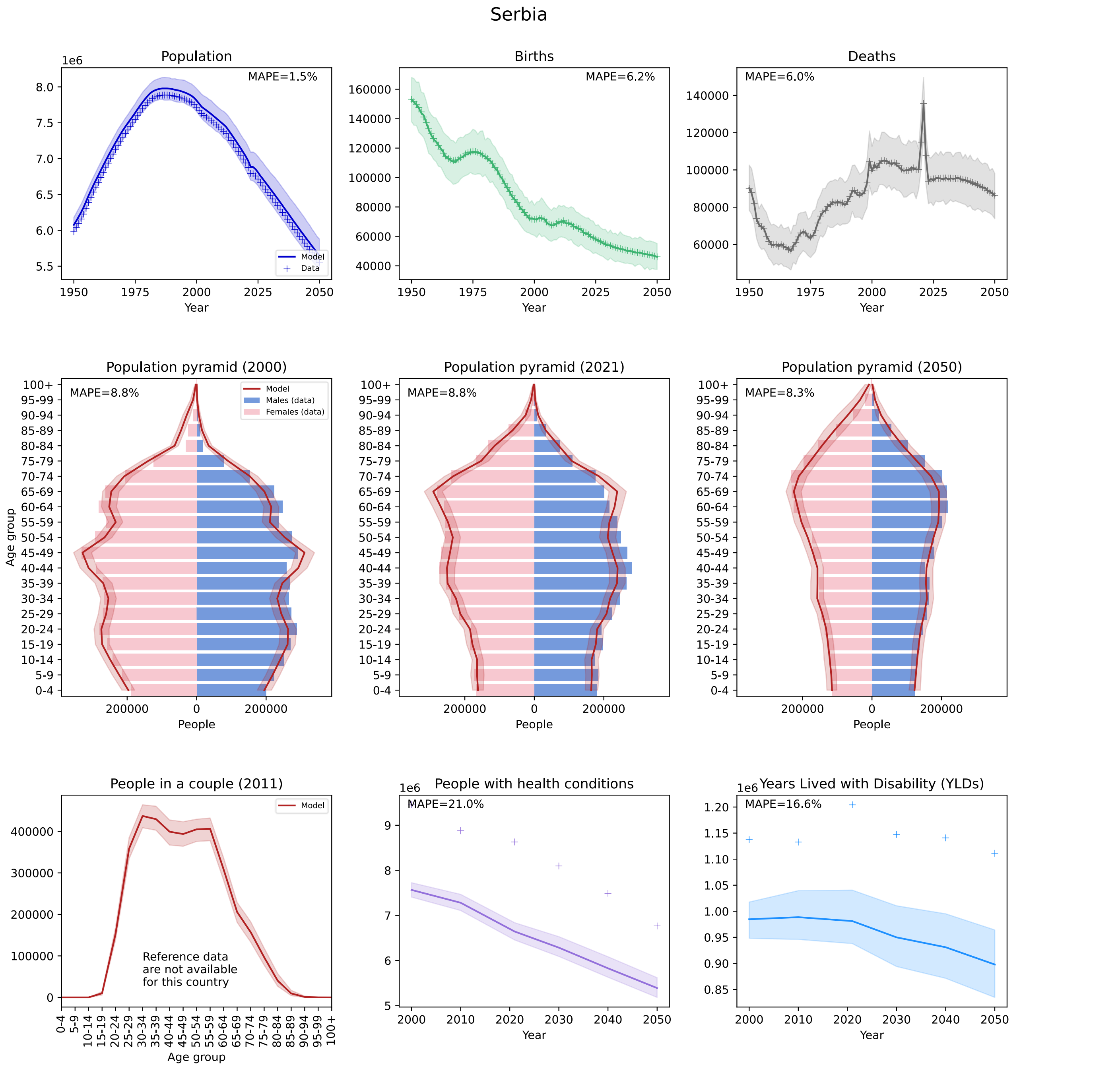
Serbia
Population
MAPE=1.5%
1e6
Model
Data
Year
Births
MAPE=6.2%
Year
Deaths
MAPE=6.0%
Year
Population pyramid (2000)
MAPE=8.8%
Model
Males (data)
Females (data)
Age group
People
Population pyramid (2021)
MAPE=8.8%
People
Population pyramid (2050)
MAPE=8.3%
People
People in a couple (2011)
Model
Reference data
are not available
for this country
Age group
People with health conditions
1e6
MAPE=21.0%
Year
Years Lived with Disability (YLDs)
1e6
MAPE=16.6%
Year

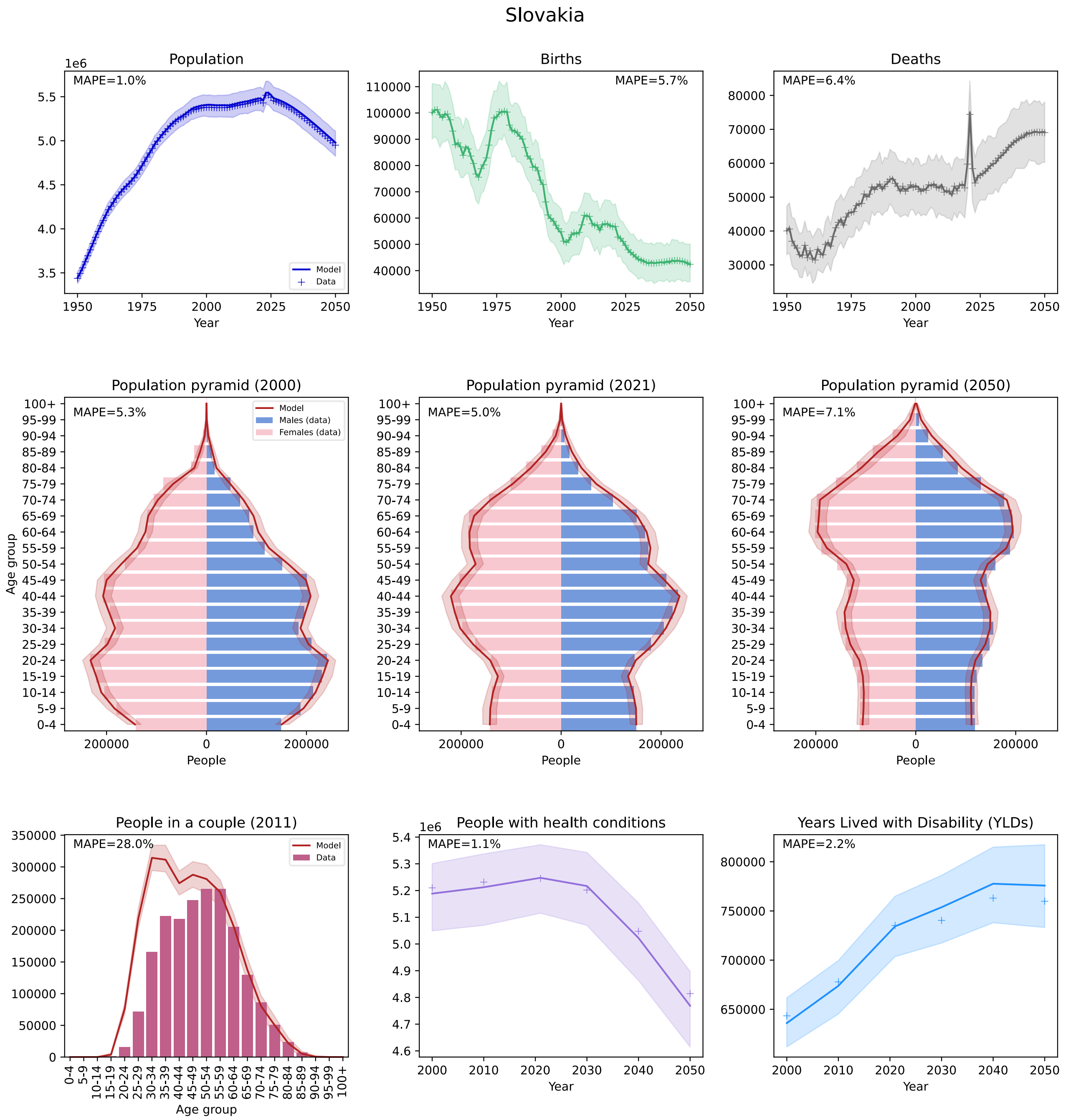
Slovakia
Population
1e6
MAPE=1.0%
Model
Data
Year
Births
MAPE=5.7%
Year
Deaths
MAPE=6.4%
Year
Population pyramid (2000)
MAPE=5.3%
Model
Males (data)
Females (data)
Age group
People
Population pyramid (2021)
MAPE=5.0%
People
Population pyramid (2050)
MAPE=7.1%
People
People in a couple (2011)
MAPE=28.0%
Model
Data
Age group
People with health conditions
1e6
MAPE=1.1%
Year
Years Lived with Disability (YLDs)
MAPE=2.2%
Year

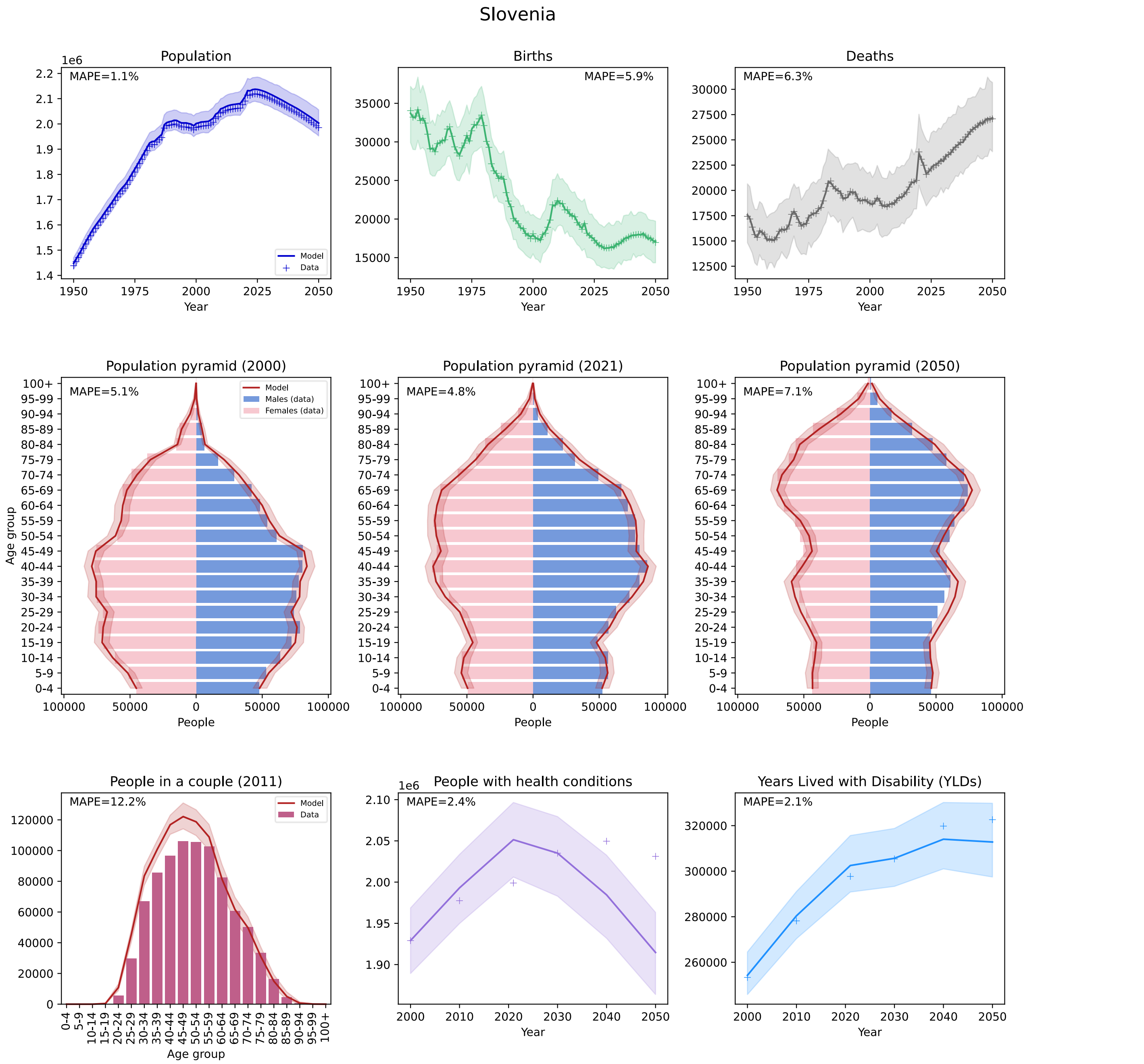

Slovenia
Population
1e6
MAPE=1.1%
Model
Data
Year
Births
MAPE=5.9%
Year
Deaths
MAPE=6.3%
Year
Population pyramid (2000)
MAPE=5.1%
Model
Males (data)
Females (data)
Age group
People
Population pyramid (2021)
MAPE=4.8%
People
Population pyramid (2050)
MAPE=7.1%
People
People in a couple (2011)
MAPE=12.2%
Model
Data
Age group
People with health conditions
1e6
MAPE=2.4%
Year
Years Lived with Disability (YLDs)
MAPE=2.1%
Year

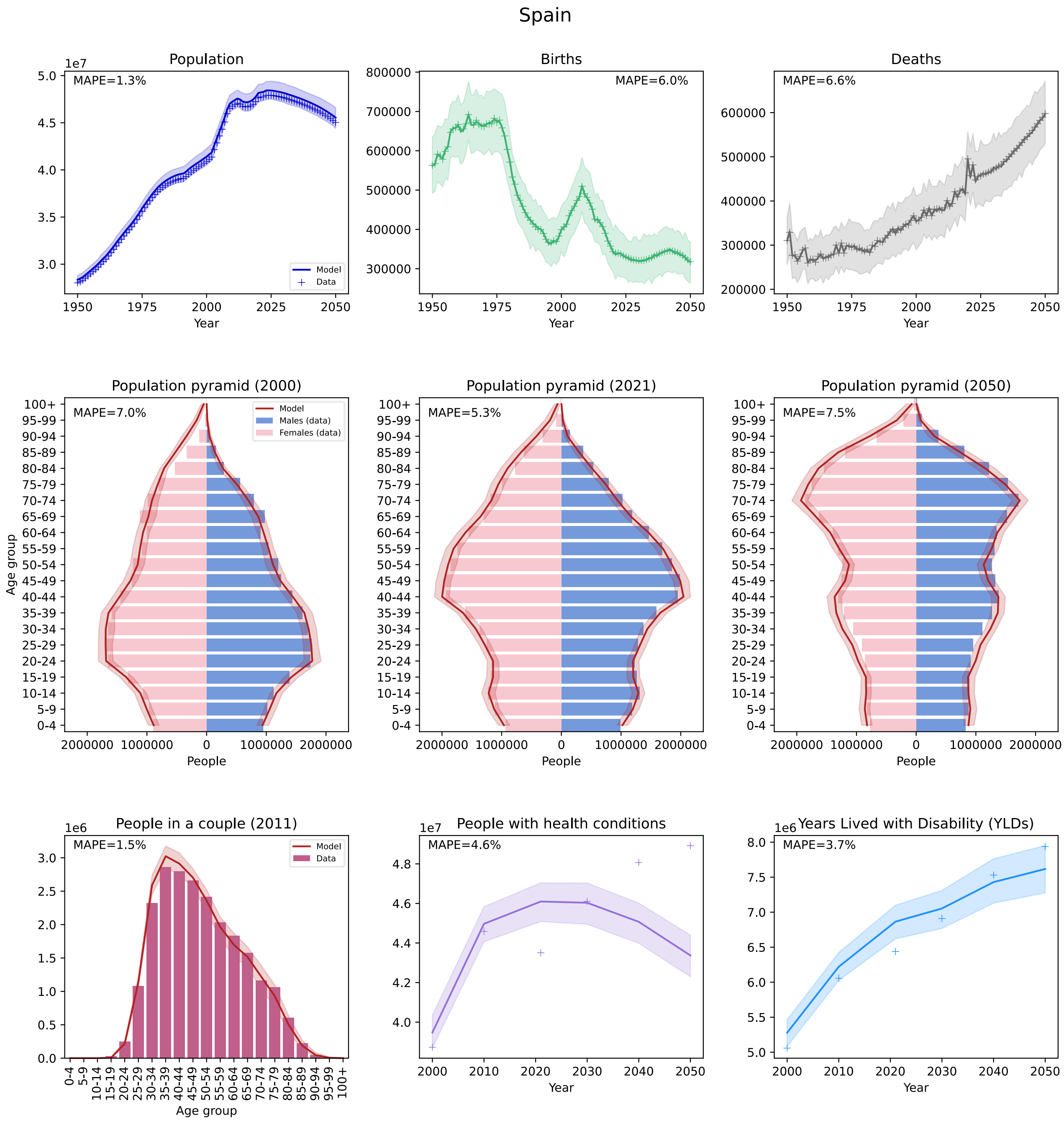
Spain
Population
MAPE=1.3%
Model
Data
Year
Births
MAPE=6.0%
Deaths
MAPE=6.6%
Population pyramid (2000)
MAPE=7.0%
Males (data)
Females (data)
Age group
People
Population pyramid (2021)
MAPE=5.3%
Population pyramid (2050)
MAPE=7.5%
People in a couple (2011)
MAPE=1.5%
People with health conditions
MAPE=4.6%
Years Lived with Disability (YLDs)
MAPE=3.7%

Sweden

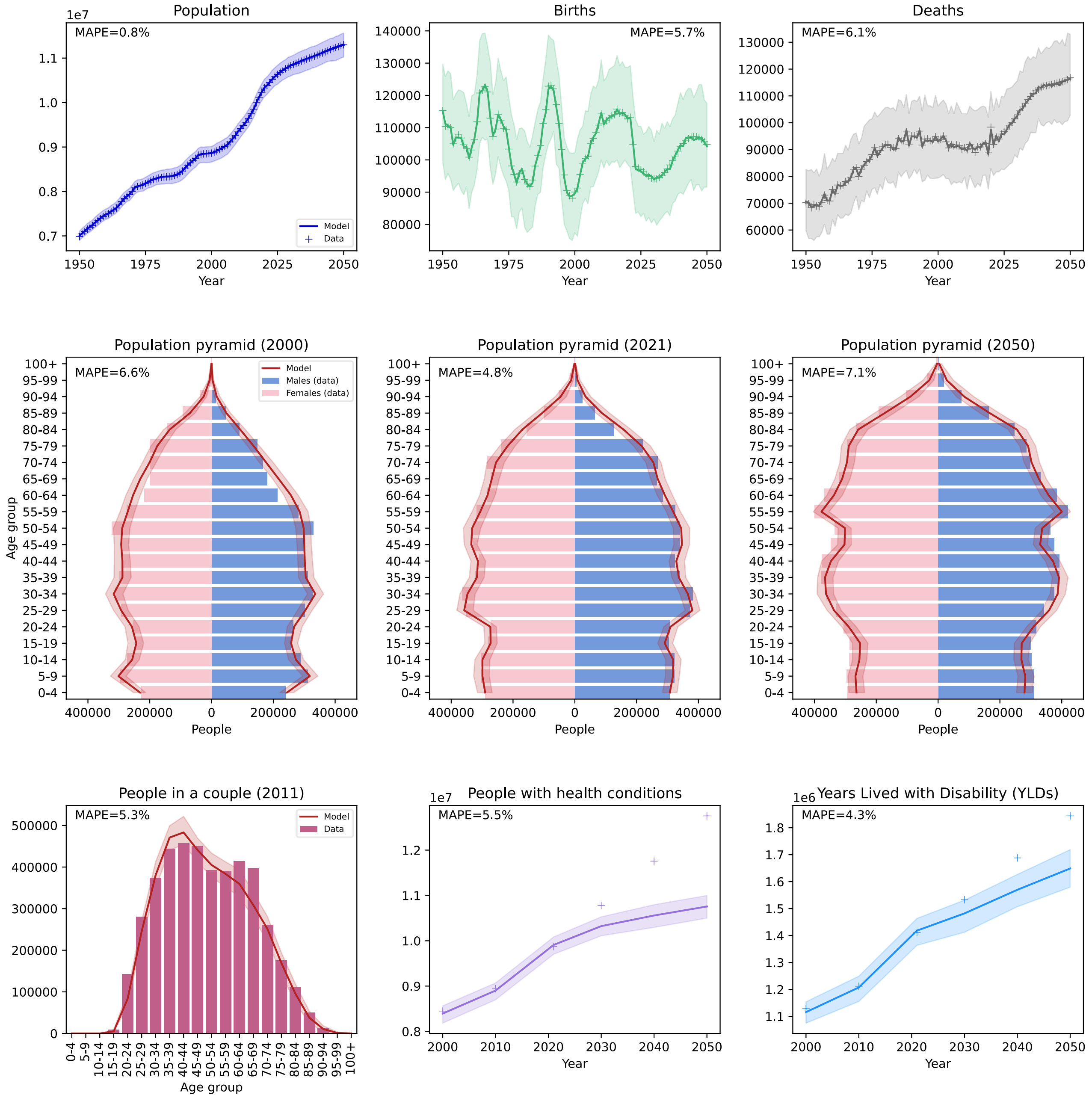

Population
1e7
MAPE=0.8%
Model
Data
Year
Births
MAPE=5.7%
Year
Deaths
MAPE=6.1%
Year
Population pyramid (2000)
MAPE=6.6%
Model
Males (data)
Females (data)
Age group
People
Population pyramid (2021)
MAPE=4.8%
People
Population pyramid (2050)
MAPE=7.1%
People
People in a couple (2011)
MAPE=5.3%
Model
Data
Age group
People with health conditions
1e7
MAPE=5.5%
Year
Years Lived with Disability (YLDs)
1e6
MAPE=4.3%
Year

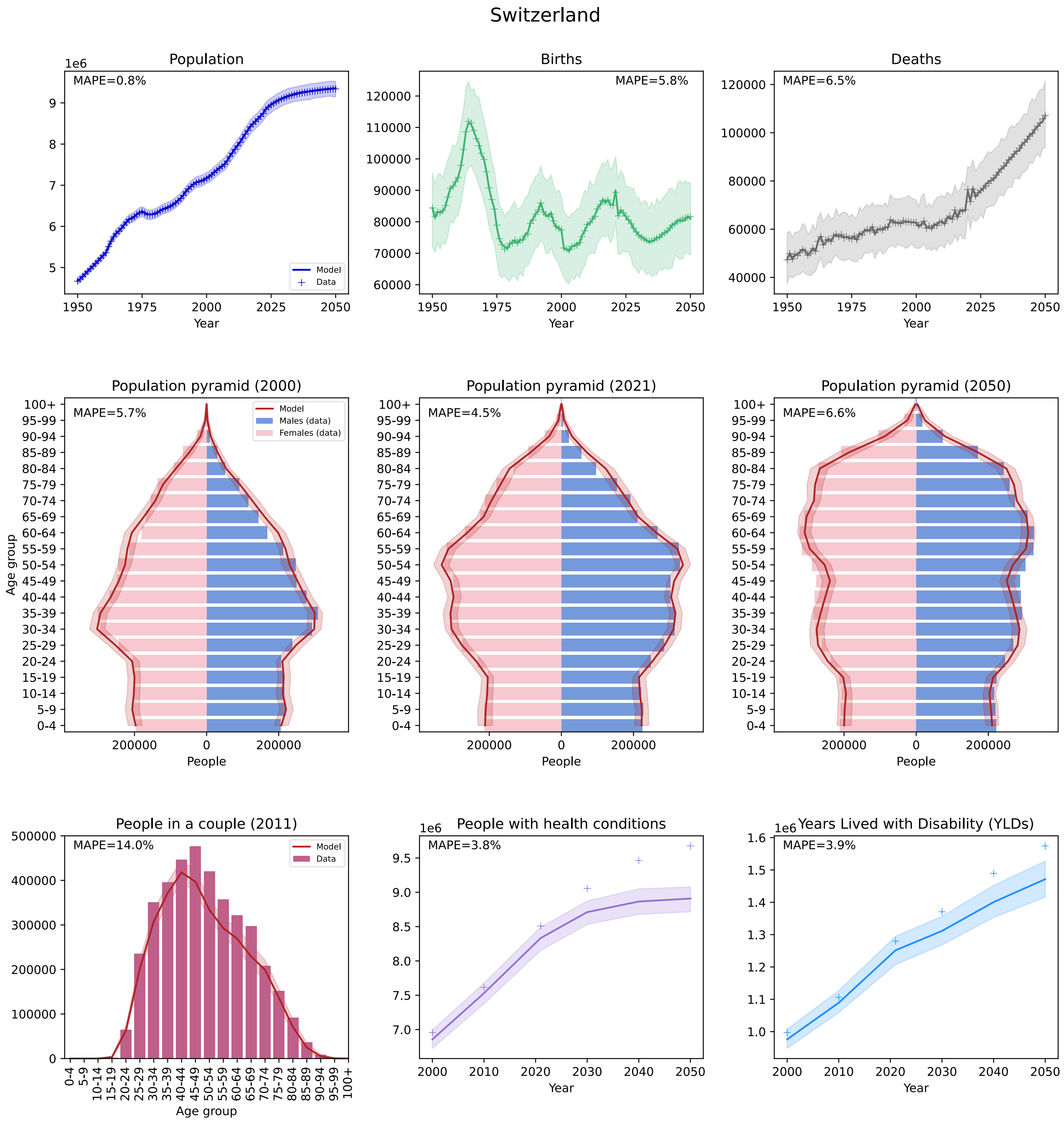
Switzerland
Population
1e6
MAPE=0.8%
Model
Data
Year
Births
MAPE=5.8%
Year
Deaths
MAPE=6.5%
Year
Population pyramid (2000)
MAPE=5.7%
Model
Males (data)
Females (data)
Age group
People
Population pyramid (2021)
MAPE=4.5%
People
Population pyramid (2050)
MAPE=6.6%
People
People in a couple (2011)
MAPE=14.0%
Model
Data
Age group
People with health conditions
1e6
MAPE=3.8%
Year
Years Lived with Disability (YLDs)
1e6
MAPE=3.9%
Year

Sensitivity analyses

Population Growth Dynamics

To assess the impact of demographic uncertainty on YLC rates across Europe, we conducted a sensitivity analysis using alternative fertility and mortality trajectories. We adjusted the UN World Population Prospects (WPP) reference projections by systematically varying births and deaths within a range of -20% to +20%. These changes were applied over the period 2023–2050.

The analysis indicates that mortality rates exert the strongest influence on YLC outcomes (Figure S2). A 20% reduction in mortality leads to a 17.3% increase in YLC rates, while a 20% increase in mortality results in a 16.5% decrease in YLC per person. Conversely, fertility rate changes have a smaller impact: a 20% increase in births reduces YLC rates by 4.1%, while a 20% decrease in births raises the informal care burden by 4.9% per person.

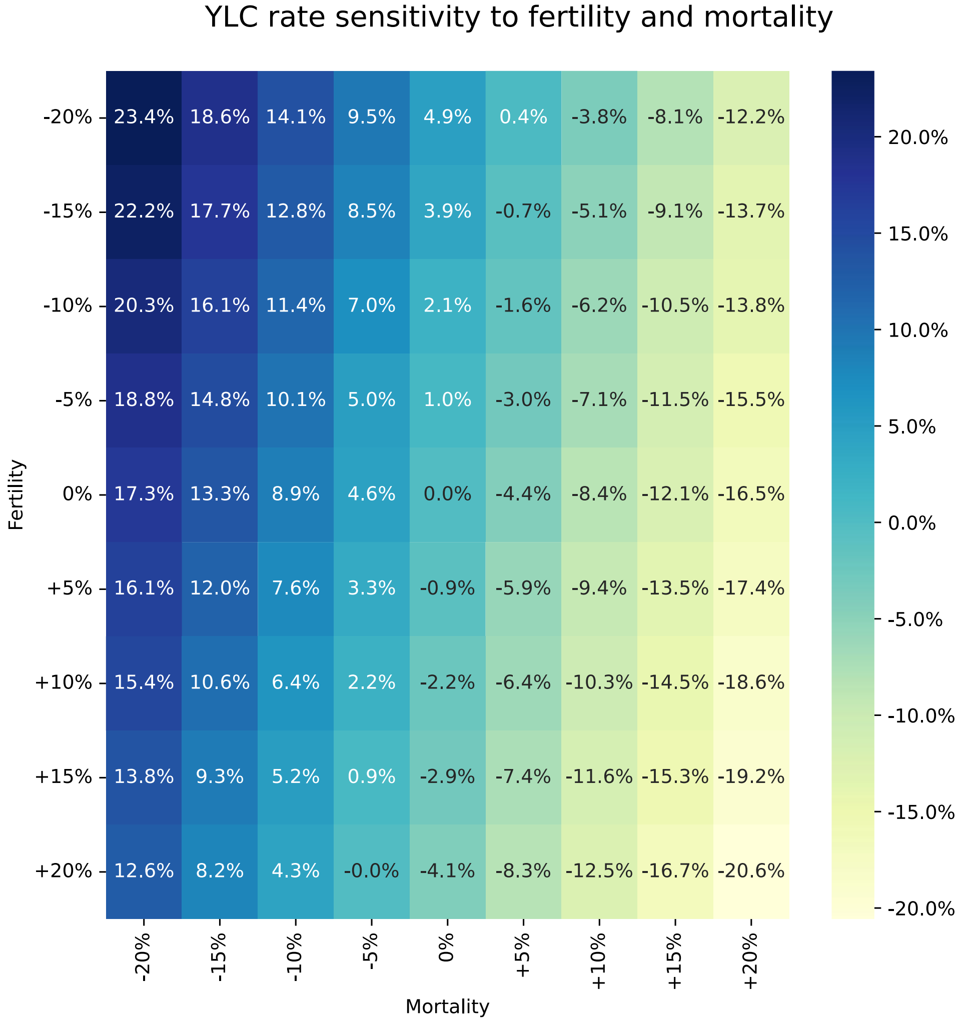

Figure S2. Change in YLC rate as of 2050 under varying fertility and mortality scenarios. Results are expressed as percentage variation with respect to the reference scenario.

Couples formation and dissolution

To assess the impact of the uncertainty on rates of formation and dissolution of romantic couples, we conducted a sensitivity analysis under alternative trajectories. We adjusted the reference rates obtained by projecting Eurostat rates with logistic growth models by systematically varying the number of expected new couples and breakups within a range of -50% to +50%. These changes were applied over the period 2022–2050.

The analysis indicates that uncertainty in rates of couple formation and dissolution trajectories over the period 2022-2050 shows modest impacts on YLC rates, ranging from -2.2% to +1.2% in the most extreme cases. (Figure S3).

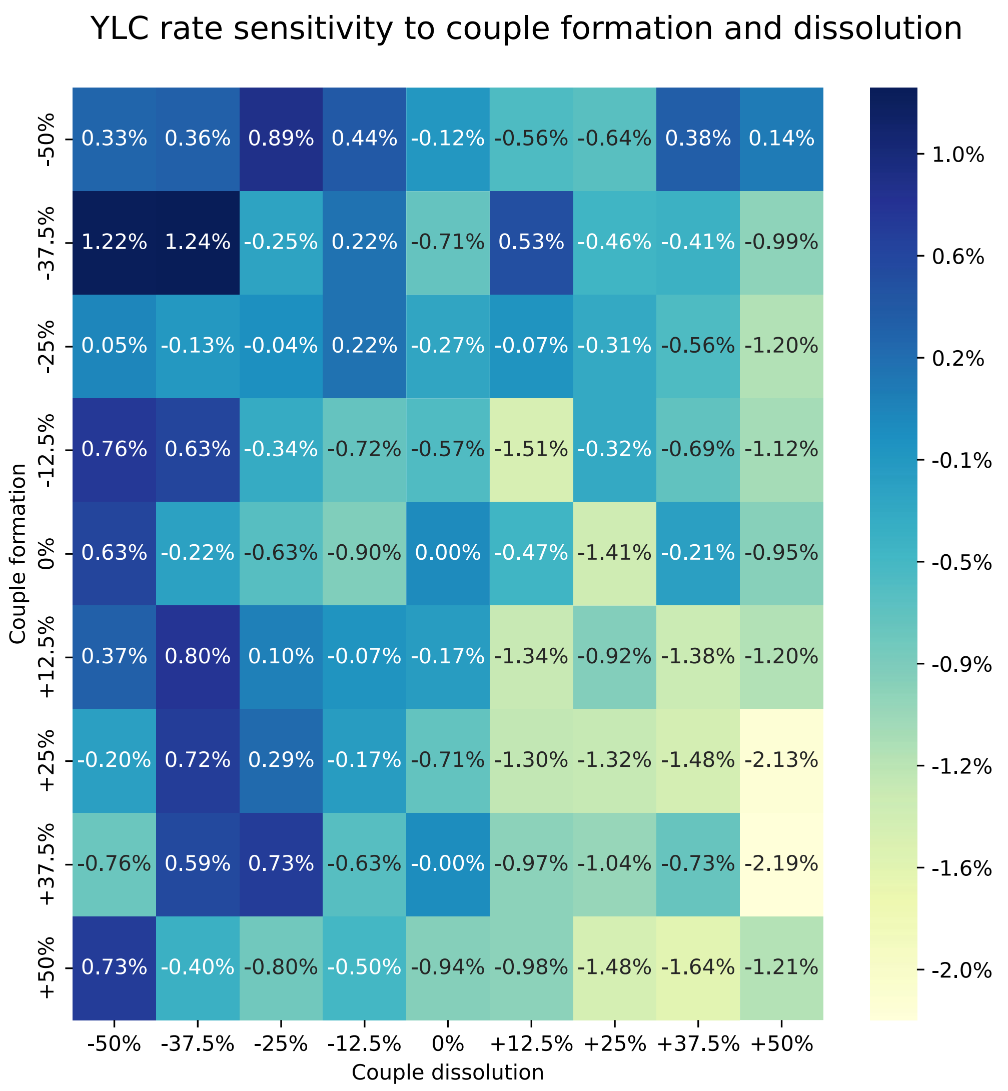

Figure S3. Change in YLC rate as of 2050 assuming varying rates of romantic couples formations and dissolutions. Results are expressed as percentage variation with respect to the reference scenario.

Health trajectories

We checked the influence on YLC rates of alternative health trajectories projected by the GBD study. They were constructed by replacing appropriate reference trajectories for risk factors with hypothetical trajectories of gradual elimination of risk factor exposure from current levels to 2050. The scenarios were constructed from various sets of risk factors: environmental risks (Safer Environment scenario), risks associated with communicable, maternal, neonatal, and nutritional diseases (Improved Childhood Nutrition and Vaccination scenario), risks associated with major non-communicable diseases (Improved Behavioural and Metabolic Risks scenario), and the combined effects of these three scenarios.

We found the best outcome under the “Combined” scenario. Under this trajectory, the YLC rate in Europe is expected to be -12.5% (-13.9 to -10.8) lower than in the “Reference” scenario. Details are reported in Table S5.

Table S5. Projections of Years Lived Caregiving (YLC) rate per 1,000 people under different GBD scenarios of the health trajectories, as of 2050. Numbers in parenthesis are 95% uncertainty intervals.

	GBD Scenario (2050)				
	**Reference**	**Safer environment**	**Improved Behavioral and Metabolic Risks**	**Improved Childhood Nutrition and Vaccination**	**Combined**
Austria	28.7 (26.5-30.8)	28.7 (26.7-30.7)	25.7 (23.7-27.7)	28.7 (26.8-30.9)	25.7 (24.1-27.6)
Belgium	29.1 (26.9-31.5)	29.1 (27-31.4)	25.3 (23.4-27.3)	29.1 (27.1-31.5)	25.3 (23.5-27.5)
Bulgaria	32.4 (29.8-35.1)	32.4 (29.7-34.9)	27.8 (25.2-30.4)	32.3 (29.6-34.8)	27.8 (25.6-30.7)
Croatia	34.1 (31.1-37.2)	34.1 (31.1-36.9)	28.8 (26.3-31.5)	34.1 (31.4-37.3)	28.8 (26.4-31.2)
Czechia	31 (28.5-33.5)	30.9 (28.3-33.5)	26.5 (23.9-28.9)	30.9 (28.4-33.7)	26.5 (24.2-28.8)
Denmark	29.5 (27.6-31.4)	29.5 (27.5-31.4)	26.5 (24.8-28.5)	29.5 (28-31.6)	26.4 (24.6-28.2)
Estonia	30.5 (28.4-32.7)	30.5 (28.6-32.9)	26.7 (24.8-28.5)	30.5 (28.3-32.9)	26.7 (24.8-28.9)
**Europe**	**31 (29.9-32)**	**31 (29.8-32.1)**	**27.1 (26.2-28.2)**	**31 (29.9-32.1)**	**27.1 (26.2-28.1)**
Finland	31.9 (29.5-34)	31.9 (29.6-34.4)	28.1 (26.1-30.2)	31.9 (29.6-34.3)	28.2 (26-30.5)
France	31 (28.7-33.1)	31 (29-33.2)	28 (25.9-29.9)	31 (28.8-33.3)	28 (26-30.1)
Germany	28.3 (26.2-30.3)	28.2 (26.1-30.7)	25.1 (23.1-27.4)	28.2 (26-30.6)	25.1 (23.1-27.1)
Greece	34 (31.7-36.5)	34 (31.3-36.9)	29.4 (27.1-31.8)	34 (31.5-36.6)	29.3 (27.3-32.4)
Hungary	30.1 (27.4-32.3)	30 (27.2-32.6)	25.8 (23.6-28.1)	30.1 (27.3-32.3)	25.8 (23.7-28.2)
Iceland	25.7 (24.1-27.2)	25.8 (24.3-27.3)	22.9 (21.8-24.3)	25.8 (24.1-27.3)	22.9 (21.6-24.2)
Ireland	26.8 (25.4-28.2)	26.8 (25.3-28.3)	23.6 (22.3-25.1)	26.8 (25.3-28.3)	23.6 (22.3-24.8)
Italy	34.3 (32-37.2)	34.3 (31.8-36.9)	30.6 (28.5-33.3)	34.3 (31.9-36.8)	30.5 (28.4-33)
Latvia	29 (26.5-31.3)	29 (26.5-31.7)	25.7 (23.6-27.8)	29 (26.8-31.6)	25.7 (23.4-27.8)
Lithuania	28.2 (25.8-30.5)	28.2 (25.5-30.6)	25.3 (23.2-27.8)	28.3 (26.1-30.7)	25.2 (23.3-27.4)
Malta	25.8 (24.3-27.2)	25.8 (24.4-27.2)	22 (20.8-23.3)	25.7 (24.3-27.2)	22 (20.6-23.5)
Montenegro	28.4 (26.2-30.6)	28.4 (26.1-30.8)	23.6 (21.5-25.5)	28.4 (26.1-30.6)	23.6 (21.6-25.4)
Netherlands	28.5 (26.3-30.7)	28.5 (26.2-30.6)	25.2 (23.3-27.5)	28.5 (26.3-30.3)	25.2 (23-27.2)
North Macedonia	32.3 (29.8-35.1)	32.3 (29.8-35.1)	26.3 (24-28.9)	32.4 (29.8-35.4)	26.4 (24.1-28.7)
Norway	30.9 (28.8-32.8)	30.9 (28.7-32.9)	28.1 (26-30)	30.9 (28.9-32.9)	28.1 (26.4-29.9)
Poland	35.4 (32.8-38.1)	35.4 (32.6-38.3)	30.3 (28.1-32.4)	35.4 (33.1-38.3)	30.2 (27.9-32.3)
Portugal	32.2 (30.1-34.7)	32.2 (30.1-34.5)	27.4 (25.6-29.4)	32.2 (30.1-34.7)	27.3 (25.4-29.1)
Romania	28.4 (25.9-31.3)	28.4 (26-31)	24.9 (22.6-27.5)	28.4 (26-30.9)	24.8 (22.4-27.3)
Serbia	34.2 (31.2-36.9)	34.1 (31-37.1)	28 (25-30.9)	34.1 (31-37.1)	27.8 (24.9-30.3)
Slovakia	33.7 (31.1-36.2)	33.8 (31-36.3)	29.4 (26.6-31.6)	33.7 (30.9-36.3)	29.4 (26.7-31.8)
Slovenia	31.3 (29-33.8)	31.3 (29.1-33.8)	27.1 (25-29.2)	31.4 (29.1-33.7)	27.2 (25.1-29.5)
Spain	31.6 (29.4-33.7)	31.7 (29.4-33.8)	26.4 (24.6-28.1)	31.6 (29.6-33.5)	26.4 (24.7-28.2)
Sweden	28.4 (26-30.4)	28.3 (26.2-30.4)	24.9 (23-26.8)	28.3 (26.2-30.1)	24.8 (22.7-26.7)
Switzerland	27.8 (25.8-29.6)	27.7 (25.9-29.7)	24.3 (22.7-26.1)	27.8 (26-30)	24.3 (22.5-26)

Institutional care capacity

We checked how different trajectories of growth of institutional care capacity (beds in nursing homes, LTC facilities, and LTC beds in hospitals) influence informal care burden. An analysis of the Eurostat databases [19], [20] showed that from 2000 to 2021 bed availability in Europe has risen linearly ($R^2 = 0.971$), at a rate of ~68,000 new beds per year. Hence, in our main analysis, we assumed this rate of growth (scenario 1x). Here we checked for different growth rates: half as fast (0.5x), twice as fast (2x), and three times as fast (3x) (Figure S4).

Under the half-as-fast-growth scenario (0.5x), we found that YLC rates increase by +1.05 % (-0.58 to +2.73) compared to the main scenario (1x), while under the most optimistic scenario (3x) YLC rates are reduced by -4.09 % (-2.57 to 5.65). Details are reported in Table S6.

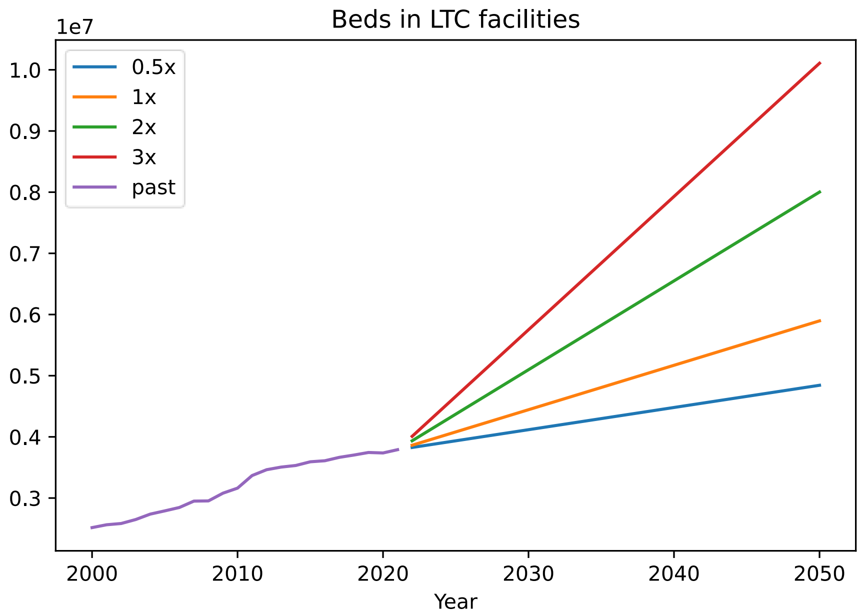

Figure S4.Bed avaiability in LTC facilities in Europe, past trajectory and future growth scenarios.

Table S6. Projections of Years Lived Caregiving (YLC) rate per 1,000 people under different scenarios of growth of institutional care capacity, as of 2050. Numbers in parenthesis are 95% uncertainty intervals.

	**Institutional Care Capacity Growth Scenario (2050)**			
	**0.5x**	**1x**	**2x**	**3x**
Austria	29.1 (27-31.3)	28.7 (26.5-30.8)	28.1 (26-29.9)	27.4 (25.3-29.5)
Belgium	29.6 (27.5-31.9)	29.1 (26.9-31.5)	28.2 (25.9-30.2)	27.3 (25.2-29.9)
Bulgaria	32.3 (29.7-35.2)	32.4 (29.8-35.1)	32.3 (29.9-35.2)	32.3 (29.6-35.1)
Croatia	34.3 (31.6-37.3)	34.1 (31.1-37.2)	33.9 (31-36.7)	33.6 (30.7-37)
Czechia	31.4 (28.5-34.3)	31 (28.5-33.5)	30.2 (27.6-32.9)	29.6 (27-31.9)
Denmark	29.8 (27.9-31.7)	29.5 (27.6-31.4)	29 (27-30.8)	28.4 (26.4-30.5)
Estonia	30.8 (28.7-33)	30.5 (28.4-32.7)	29.9 (27.7-31.9)	29.3 (27.2-31.8)
**Europe**	**31.3 (30.1-32.4)**	**31 (29.9-32)**	**30.4 (29.3-31.5)**	**29.7 (28.7-30.8)**
Finland	32.4 (30.2-34.8)	31.9 (29.5-34)	30.9 (29-33.2)	29.9 (27.9-32.5)
France	31.4 (29.2-33.8)	31 (28.7-33.1)	30.2 (28.1-32.6)	29.3 (26.9-31.5)
Germany	28.7 (26.5-31.2)	28.3 (26.2-30.3)	27.4 (25-29.9)	26.5 (24.4-28.6)
Greece	34 (31.4-36.4)	34 (31.7-36.5)	34 (31.5-36.6)	33.9 (31.5-36.5)
Hungary	30.5 (28-33.4)	30.1 (27.4-32.3)	29.3 (26.8-31.7)	28.5 (26.2-31.1)
Iceland	26 (24.3-27.8)	25.7 (24.1-27.2)	25.4 (23.7-27.2)	25 (23.4-26.7)
Ireland	27 (25.5-28.5)	26.8 (25.4-28.2)	26.4 (25-27.7)	26 (24.5-27.6)
Italy	34.5 (32.2-37.1)	34.3 (32-37.2)	33.9 (31.6-36.7)	33.5 (31.3-36)
Latvia	29.1 (26.9-31.6)	29 (26.5-31.3)	28.7 (26.3-31)	28.6 (26.3-31)
Lithuania	28.6 (26.2-30.9)	28.2 (25.8-30.5)	27.7 (25.3-30.1)	27.1 (24.6-29.3)
Malta	26.2 (24.6-27.7)	25.8 (24.3-27.2)	25 (23.6-26.6)	24.3 (22.8-25.9)
Montenegro	28.5 (26.1-30.6)	28.4 (26.2-30.6)	28.4 (26.1-30.6)	28.4 (26-30.7)
Netherlands	29.1 (26.9-31.5)	28.5 (26.3-30.7)	27.4 (25.1-29.6)	26.3 (24.2-28.2)
North Macedonia	32.5 (30-35.4)	32.3 (29.8-35.1)	32.3 (29.7-35.1)	32.1 (29.4-35.1)
Norway	31.2 (29.2-33.3)	30.9 (28.8-32.8)	30.4 (28.2-32.3)	29.9 (27.9-31.8)
Poland	35.5 (32.5-38.4)	35.4 (32.8-38.1)	35.2 (32.5-38.1)	35.1 (32.5-37.6)

	Institutional Care Capacity Growth Scenario (2050)			
	**0.5x**	**1x**	**2x**	**3x**
Portugal	32.2 (29.8-34.3)	32.2 (30.1-34.7)	32.1 (30-34.4)	32.1 (30.1-34.4)
Romania	28.6 (26.1-31.2)	28.4 (25.9-31.3)	28.3 (25.8-31.1)	27.8 (25.6-29.9)
Serbia	34.4 (31.4-37.6)	34.2 (31.2-36.9)	33.7 (30.4-36.6)	33.4 (30.3-36.7)
Slovakia	34.1 (31.5-36.6)	33.7 (31.1-36.2)	33 (30.5-35.7)	32.3 (29.5-35.1)
Slovenia	31.8 (29.6-34)	31.3 (29-33.8)	30.6 (28-33)	29.7 (27.3-31.9)
Spain	32 (29.9-34.3)	31.6 (29.4-33.7)	31 (29-33.1)	30.4 (28.4-32.5)
Sweden	28.9 (26.5-30.9)	28.4 (26-30.4)	27.4 (25.4-29.6)	26.5 (24.4-28.6)
Switzerland	28.1 (26.3-29.8)	27.8 (25.8-29.6)	27.1 (25.4-29)	26.4 (24.8-28.3

Years Lived Caregiving and median population age

A linear regression analysis was performed to assess the relationship between median age (independent variable) and YLC rate per 1,000 people (dependent variable). The data set includes country-level observations from multiple time points (2000, 2010, 2021, 2030, 2040, 2050. Median population age of selected European countries was obtained from the UN WPP. This approach assumes a linear association between these two variables, which is supported by the visual fit of the data points to the regression line. The 95% uncertainty interval around the regression line was computed to capture the confidence in the model's predictions. Data points are presented with clear year-specific distinctions, facilitating the interpretation of both historical and projected trends.

Figure S5 illustrates a strong positive relationship ($R^2 = 0.711$, p-value < 0.0001) between median age and informal care burden, measured as YLC per 1,000 people, across countries included in this study. As the median age increases, the YLC burden per capita also rises substantially. This trend is consistent across years, though data points from later years (2030–2050) are clustered at higher median ages, reflecting projected demographic changes. The tight clustering along the regression line and high value suggest that median age is a robust predictor of informal care burden.

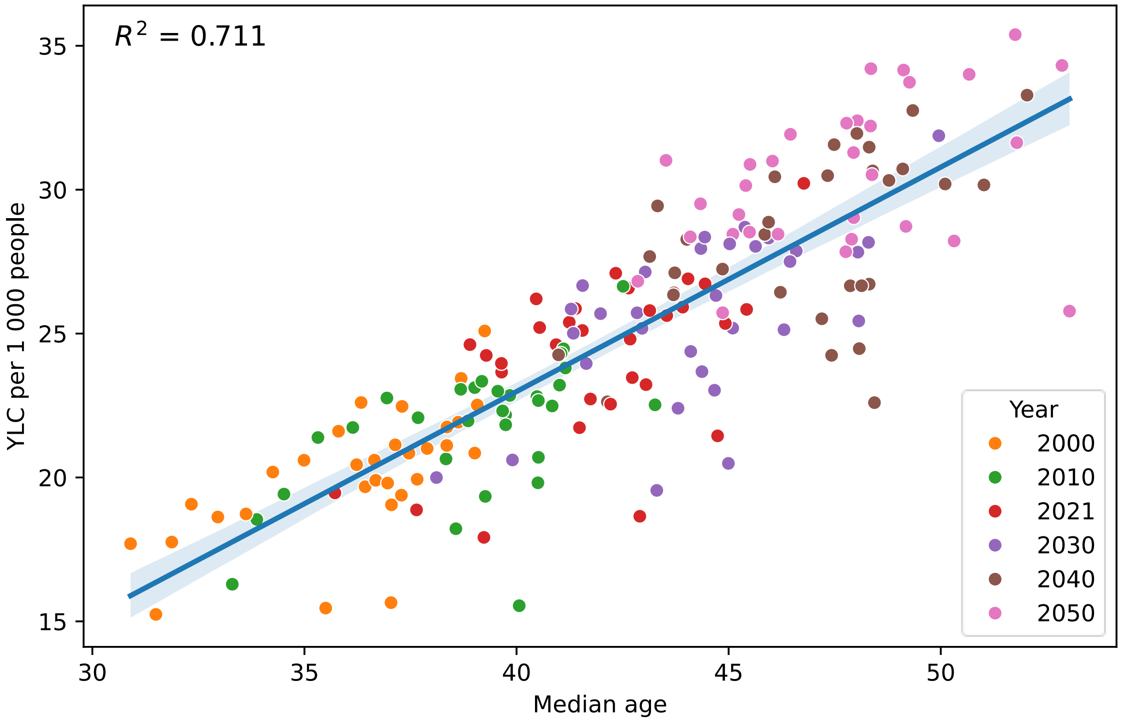

Figure S5. Scatterplot showing the relationship between median age and informal care burden per capita (YLC rate) across countries included in the study. Each dot represents a country, color-coded by year. The shaded region represents the 95% uncertainty interval (UI).

Years Lived Caregiving by GBD cause

Table S7. Years Lived Caregiving for older adults, per 100 000 people. Data are aggregates of selected European countries.

	Years Lived Caregiving (YLC) rate per 100 000		
	2000	2021	2050
All causes	2.13e+03 (2.05e+03 to 2.19e+03)	2.58e+03 (2.49e+03 to 2.66e+03)	3.1e+03 (2.99e+03 to 3.2e+03)
Communicable, maternal, neonatal, and nutritional diseases	36.7 (33.1 to 39.8)	64.9 (60.9 to 69.1)	50.8 (47.4 to 54.5)
HIV/AIDS and sexually transmitted infections	1.46 (0.97 to 2.12)	2.79 (2.15 to 3.61)	0.684 (0.619 to 0.83)
HIV/AIDS	0.858 (0.405 to 1.53)	2.12 (1.48 to 2.98)	0 (0 to 0)
HIV/AIDS - Drug-susceptible Tuberculosis	0.0384 (0 to 0.432)	0.0177 (0 to 0.261)	0 (0 to 0)
HIV/AIDS - Multidrug-resistant Tuberculosis without extensive drug resistance	0.000929 (0 to 0)	0.00074 (0 to 0)	0 (0 to 0)
HIV/AIDS - Extensively drug-resistant Tuberculosis	0 (0 to 0)	0 (0 to 0)	0 (0 to 0)
HIV/AIDS resulting in other diseases	0.819 (0.371 to 1.46)	2.1 (1.4 to 2.95)	0 (0 to 0)
Sexually transmitted infections excluding HIV	0.6 (0.539 to 0.724)	0.672 (0.612 to 0.813)	0.684 (0.619 to 0.83)
Syphilis	0.0413 (0.0149 to 0.127)	0.0404 (0.0174 to 0.0914)	0.0448 (0.00731 to 0.128)
Chlamydial infection	0.0077 (0.00469 to 0.0118)	0.00816 (0.00452 to 0.0134)	0.00647 (0.00344 to 0.0103)
Gonococcal infection	0.00258 (0.00114 to 0.00453)	0.00256 (0.00118 to 0.00449)	0.0013 (0.00039 to 0.00286)
Trichomoniasis	0.154 (0.126 to 0.181)	0.17 (0.145 to 0.2)	0.168 (0.141 to 0.193)
Genital herpes	0.382 (0.362 to 0.402)	0.43 (0.41 to 0.45)	0.45 (0.427 to 0.47)
Other sexually transmitted infections	0.0127 (0 to 0.0966)	0.0208 (0 to 0.145)	0.0141 (0 to 0.126)
Respiratory infections and tuberculosis	6.83 (5.93 to 8.02)	28.1 (25.5 to 30.8)	6.2 (5.52 to 6.88)
Tuberculosis	0.979 (0.376 to 1.77)	0.52 (0.069 to 1.22)	0 (0 to 0)
Latent tuberculosis infection	0 (0 to 0)	0 (0 to 0)	0 (0 to 0)
Drug-susceptible tuberculosis	0.949 (0.375 to 1.77)	0.502 (0.0501 to 1.21)	0 (0 to 0)
Multidrug-resistant tuberculosis without extensive drug resistance	0.0294 (0 to 0.243)	0.0142 (0 to 0.123)	0 (0 to 0)
Extensively drug-resistant tuberculosis	0.0007 (0 to 0)	0.00308 (0 to 0.0193)	0 (0 to 0)
Lower respiratory infections	0.314 (0.123 to 0.58)	0.297 (0.109 to 0.555)	0.476 (0.242 to 0.752)
Upper respiratory infections	4.04 (3.44 to 4.68)	4.56 (4.08 to 5.08)	4.73 (4.19 to 5.32)
Otitis media	1.5 (1.22 to 1.81)	1.53 (1.22 to 1.89)	0.999 (0.745 to 1.24)
COVID-19	0 (0 to 0)	21.2 (18.7 to 23.6)	0 (0 to 0)
Enteric infections	3.56 (2.55 to 4.67)	4.61 (3.61 to 5.94)	12.9 (11 to 15)
Diarrheal diseases	3.56 (2.55 to 4.67)	4.61 (3.61 to 5.94)	12.9 (11 to 15)
Typhoid and paratyphoid	0.00106 (0 to 0.00299)	0.00147 (0 to 0.0114)	0.000767 (0 to 0)
Typhoid fever	0.000834 (0 to 0)	0.00114 (0 to 0)	0.000751 (0 to 0)
Paratyphoid fever	0.000224 (0 to 0)	0.000337 (0 to 0)	1.65e-05 (0 to 0)

	Years Lived Caregiving (YLC) rate per 100 000		
	2000	2021	2050
Invasive Non-typhoidal Salmonella (iNTS)	0.000565 (0 to 0)	6.36e-05 (0 to 0)	0 (0 to 0)
Neglected tropical diseases and malaria	2.69 (1.79 to 3.69)	2.69 (1.73 to 3.58)	3.14 (2.36 to 4.05)
Malaria	0 (0 to 0)	0 (0 to 0)	0 (0 to 0)
Chagas disease	0.00661 (0 to 0.0322)	0.0172 (0 to 0.0432)	0.0183 (0.000223 to 0.0512)
Leishmaniasis	0.0124 (0 to 0.0706)	0.0105 (0 to 0.0738)	0.0142 (0 to 0.0747)
Visceral leishmaniasis	0.000335 (0 to 0)	0.000278 (0 to 0)	4.41e-05 (0 to 0)
Cutaneous and mucocutaneous leishmaniasis	0.0121 (0 to 0.0706)	0.0102 (0 to 0.0738)	0.0141 (0 to 0.0747)
African trypanosomiasis	0 (0 to 0)	0 (0 to 0)	0 (0 to 0)
Schistosomiasis	0 (0 to 0)	0 (0 to 0)	0 (0 to 0)
Cysticercosis	2.12 (1.23 to 3.16)	2.17 (1.26 to 3.06)	2.72 (1.88 to 3.63)
Cystic echinococcosis	0.0617 (0.00296 to 0.191)	0.0589 (0.00169 to 0.165)	0.0609 (0.000783 to 0.205)
Lymphatic filariasis	0 (0 to 0)	0 (0 to 0)	0 (0 to 0)
Onchocerciasis	0 (0 to 0)	0 (0 to 0)	0 (0 to 0)
Trachoma	0 (0 to 0)	0 (0 to 0)	0 (0 to 0)
Dengue	0 (0 to 0)	0 (0 to 0)	0 (0 to 0)
Yellow fever	0 (0 to 0)	0 (0 to 0)	0 (0 to 0)
Rabies	0 (0 to 0)	0 (0 to 0)	0 (0 to 0)
Intestinal nematode infections	0 (0 to 0)	0 (0 to 0)	0 (0 to 0)
Ascariasis	0 (0 to 0)	0 (0 to 0)	0 (0 to 0)
Trichuriasis	0 (0 to 0)	0 (0 to 0)	0 (0 to 0)
Hookworm disease	0 (0 to 0)	0 (0 to 0)	0 (0 to 0)
Food-borne trematodiases	0.0815 (0.0517 to 0.114)	0.0583 (0.0304 to 0.0909)	0.0502 (0.0244 to 0.0846)
Leprosy	0 (0 to 0)	0 (0 to 0)	0 (0 to 0)
Ebola	0 (0 to 0)	0 (0 to 0)	0 (0 to 0)
Zika virus	0 (0 to 0)	0 (0 to 0)	0 (0 to 0)
Guinea worm disease	0 (0 to 0)	0 (0 to 0)	0 (0 to 0)
Other neglected tropical diseases	0.402 (0.294 to 0.531)	0.379 (0.257 to 0.505)	0.273 (0.189 to 0.374)
Other infectious diseases	1.74 (1.31 to 2.25)	1.81 (1.41 to 2.34)	1.74 (1.29 to 2.24)
Meningitis	0.203 (0.0696 to 0.403)	0.114 (0.0229 to 0.257)	0.0587 (0.00535 to 0.178)
Encephalitis	0.139 (0.0239 to 0.381)	0.166 (0.0257 to 0.432)	0.173 (0.0383 to 0.443)
Diphtheria	2.02e-05 (0 to 0)	0 (0 to 0)	0 (0 to 0)
Pertussis	0.00179 (0 to 0.0135)	2.69e-05 (0 to 0)	0.0005 (0 to 0.0042)
Tetanus	0.000271 (0 to 0)	0 (0 to 0)	0 (0 to 0)
Measles	2.64e-05 (0 to 0)	0 (0 to 0)	0 (0 to 0)
Varicella and herpes zoster	0.664 (0.349 to 1.02)	0.793 (0.469 to 1.17)	0.712 (0.432 to 1.06)
Acute hepatitis	0.238 (0.114 to 0.444)	0.245 (0.113 to 0.42)	0.233 (0.0887 to 0.418)
Acute hepatitis A	0.117 (0.0206 to 0.263)	0.127 (0.0285 to 0.277)	0.146 (0.0333 to 0.289)
Acute hepatitis B	0.0797 (0.0257 to 0.16)	0.0706 (0.0167 to 0.151)	0.0442 (0.00726 to 0.105)

	Years Lived Caregiving (YLC) rate per 100 000		
	2000	2021	2050
Acute hepatitis C	0.018 (1.56e-05 to 0.0548)	0.0202 (0.000979 to 0.0584)	0.0109 (0 to 0.0359)
Acute hepatitis E	0.0236 (0 to 0.0807)	0.0271 (0 to 0.086)	0.0321 (0.000448 to 0.0933)
Other unspecified infectious diseases	0.492 (0.354 to 0.664)	0.494 (0.358 to 0.675)	0.565 (0.405 to 0.782)
Maternal and neonatal disorders	9.71 (7.47 to 12)	11.7 (9.64 to 14.1)	12 (10.1 to 14.3)
Maternal disorders	0.000261 (0 to 0.000966)	0.00131 (0 to 0.00747)	0.000516 (0 to 0.00337)
Maternal hemorrhage	0 (0 to 0)	0.000236 (0 to 0)	0.000191 (0 to 0)
Maternal sepsis and other maternal infections	0.00011 (0 to 0.000956)	0.000331 (0 to 0.00325)	0.000235 (0 to 0.00203)
Maternal hypertensive disorders	0.000151 (0 to 0)	6.24e-05 (0 to 0)	0 (0 to 0)
Maternal obstructed labor and uterine rupture	0 (0 to 0)	0 (0 to 0)	0 (0 to 0)
Maternal abortion and miscarriage	0 (0 to 0)	0.000682 (0 to 0)	2.19e-06 (0 to 0)
Ectopic pregnancy	0 (0 to 0)	0 (0 to 0)	8.71e-05 (0 to 0)
Neonatal disorders	9.71 (7.47 to 12)	11.7 (9.64 to 14.1)	12 (10.1 to 14.3)
Neonatal preterm birth	5.4 (4.13 to 6.72)	6.81 (5.37 to 8.17)	6.73 (5.55 to 8.14)
Neonatal encephalopathy due to birth asphyxia and trauma	1.98 (0.989 to 3.04)	2.5 (1.52 to 3.68)	3.08 (1.93 to 4.45)
Neonatal sepsis and other neonatal infections	2.17 (1.22 to 3.5)	2.21 (1.16 to 3.34)	2.02 (1.22 to 3.16)
Hemolytic disease and other neonatal jaundice	0.169 (0 to 0.794)	0.191 (0 to 0.777)	0.222 (0 to 0.803)
Nutritional deficiencies	10.7 (9.84 to 11.5)	13.2 (11.9 to 14.4)	14.1 (13.1 to 15.2)
Protein-energy malnutrition	0.588 (0.379 to 0.848)	3.61 (2.79 to 4.5)	4.01 (3.22 to 4.82)
Iodine deficiency	1.54 (1.31 to 1.8)	1.42 (1.22 to 1.62)	1.21 (1.02 to 1.42)
Vitamin A deficiency	0 (0 to 0)	0 (0 to 0)	0 (0 to 0)
Dietary iron deficiency	8.56 (7.79 to 9.28)	8.21 (7.55 to 8.87)	8.86 (8.16 to 9.76)
Non-communicable diseases	1.85e+03 (1.78e+03 to 1.91e+03)	2.26e+03 (2.18e+03 to 2.33e+03)	2.76e+03 (2.67e+03 to 2.85e+03)
Neoplasms	62.3 (57 to 67.9)	77.4 (72.5 to 82.8)	105 (99.1 to 111)
Lip and oral cavity cancer	0.9 (0.432 to 1.49)	1.16 (0.659 to 1.74)	1.47 (0.856 to 2.2)
Nasopharynx cancer	0.085 (0 to 0.304)	0.0796 (0 to 0.36)	0.104 (0 to 0.475)
Other pharynx cancer	0.295 (0.0179 to 0.817)	0.47 (0.145 to 0.991)	0.621 (0.193 to 1.21)
Esophageal cancer	0.457 (0.0574 to 1.04)	0.544 (0.154 to 0.998)	0.753 (0.3 to 1.44)
Stomach cancer	1.58 (0.803 to 2.54)	1.31 (0.665 to 2.05)	1.44 (0.748 to 2.25)
Colon and rectum cancer	9.91 (8.2 to 11.6)	12.3 (10.6 to 14.1)	17.4 (15.3 to 19.9)
Liver cancer	0.431 (0.0492 to 1.11)	0.625 (0.154 to 1.22)	0.955 (0.292 to 1.71)
Liver cancer due to hepatitis B	0.0448 (0 to 0.252)	0.0683 (0 to 0.285)	0.116 (0 to 0.38)
Liver cancer due to hepatitis C	0.17 (0 to 0.6)	0.26 (0.00321 to 0.689)	0.389 (0.0174 to 1.06)
Liver cancer due to alcohol use	0.159 (0 to 0.621)	0.225 (0.00405 to 0.627)	0.311 (0.027 to 0.741)
Liver cancer due to NASH	0.0406 (0 to 0.294)	0.0466 (0 to 0.305)	0.1 (0 to 0.474)
Hepatoblastoma	0 (0 to 0)	0 (0 to 0)	0 (0 to 0)
Liver cancer due to other causes	0.0167 (0 to 0.214)	0.0255 (0 to 0.215)	0.0394 (0 to 0.25)
Gallbladder and biliary tract cancer	0.456 (0.0351 to 1.11)	0.469 (0.0844 to 0.939)	0.462 (0.0918 to 0.921)

	Years Lived Caregiving (YLC) rate per 100 000		
	2000	2021	2050
Pancreatic cancer	0.802 (0.167 to 1.69)	1.2 (0.486 to 2.08)	2.01 (1.01 to 3.16)
Larynx cancer	0.983 (0.536 to 1.53)	0.933 (0.453 to 1.43)	0.971 (0.49 to 1.54)
Tracheal, bronchus, and lung cancer	3.71 (2.35 to 5.18)	4.48 (3.22 to 5.95)	5.86 (4.18 to 7.72)
Malignant skin melanoma	1.18 (0.743 to 1.7)	2.13 (1.5 to 2.84)	2.5 (1.74 to 3.16)
Non-melanoma skin cancer	0.166 (0.0465 to 0.341)	0.221 (0.0939 to 0.399)	0.36 (0.154 to 0.581)
Non-melanoma skin cancer (squamous-cell carcinoma)	0.156 (0.038 to 0.33)	0.211 (0.0811 to 0.378)	0.346 (0.145 to 0.565)
Non-melanoma skin cancer (basal-cell carcinoma)	0.00982 (0.00163 to 0.0224)	0.0104 (0.00231 to 0.0225)	0.0146 (0.00451 to 0.0288)
Soft tissue and other extraosseous sarcomas	0.274 (0.0447 to 0.672)	0.472 (0.124 to 0.906)	0.711 (0.277 to 1.25)
Malignant neoplasm of bone and articular cartilage	0.223 (0.0264 to 0.566)	0.19 (0.0186 to 0.521)	0.37 (0.0734 to 0.867)
Breast cancer	12.4 (10.7 to 14.2)	14.6 (12.9 to 16.2)	16.9 (15.2 to 18.8)
Cervical cancer	0.606 (0.257 to 1.01)	0.655 (0.27 to 1.14)	0.611 (0.249 to 1.11)
Uterine cancer	1.95 (1.38 to 2.55)	2.83 (2.08 to 3.51)	3.59 (2.81 to 4.32)
Ovarian cancer	1.32 (0.652 to 2.2)	1.2 (0.616 to 1.94)	1.49 (0.7 to 2.31)
Prostate cancer	9.16 (7.49 to 10.7)	12 (10.4 to 13.9)	20.5 (17.9 to 22.8)
Testicular cancer	0.0546 (0 to 0.243)	0.0927 (0.00209 to 0.272)	0.0969 (0.00292 to 0.269)
Kidney cancer	1.36 (0.778 to 1.94)	1.82 (1.23 to 2.65)	2.36 (1.62 to 3.42)
Bladder cancer	3.34 (2.38 to 4.31)	4.1 (3.21 to 5.13)	5.48 (4.3 to 6.73)
Brain and central nervous system cancer	0.669 (0.202 to 1.44)	0.821 (0.32 to 1.49)	1.35 (0.564 to 2.26)
Eye cancer	0.142 (0.0114 to 0.369)	0.223 (0.0449 to 0.532)	0.258 (0.0517 to 0.57)
Retinoblastoma	0 (0 to 0)	0 (0 to 0)	0 (0 to 0)
Other eye cancers	0.142 (0.0114 to 0.369)	0.223 (0.0449 to 0.532)	0.258 (0.0517 to 0.57)
Neuroblastoma and other peripheral nervous cell tumors	0.00469 (0 to 0.0381)	0.00356 (0 to 0.0591)	0.00785 (0 to 0.058)
Thyroid cancer	0.585 (0.273 to 0.97)	0.621 (0.328 to 1.03)	0.791 (0.399 to 1.25)
Mesothelioma	0.138 (0 to 0.571)	0.189 (0 to 0.589)	0.199 (0 to 0.742)
Hodgkin lymphoma	0.176 (0.0101 to 0.519)	0.196 (0.0194 to 0.498)	0.256 (0.0367 to 0.665)
Non-Hodgkin lymphoma	2.84 (1.98 to 3.94)	3.97 (2.71 to 5.37)	5.01 (3.77 to 6.42)
Burkitt lymphoma	0.0553 (0 to 0.244)	0.107 (0 to 0.308)	0.145 (0.00179 to 0.422)
Other non-Hodgkin lymphoma	2.79 (1.9 to 3.84)	3.86 (2.62 to 5.31)	4.87 (3.66 to 6.24)
Multiple myeloma	1.07 (0.281 to 2.14)	1.47 (0.706 to 2.41)	2 (0.983 to 3.18)
Leukemia	2.18 (1.16 to 3.39)	2.61 (1.64 to 3.8)	3.51 (2.16 to 4.97)
Acute lymphoid leukemia	0.0443 (0 to 0.365)	0.0372 (0 to 0.294)	0.05 (0 to 0.314)
Chronic lymphoid leukemia	1.43 (0.623 to 2.37)	1.72 (0.925 to 2.52)	2.27 (1.4 to 3.25)
Acute myeloid leukemia	0.248 (0 to 0.673)	0.401 (0.0261 to 0.993)	0.59 (0.121 to 1.29)
Chronic myeloid leukemia	0.195 (0 to 0.675)	0.112 (0 to 0.458)	0.145 (0 to 0.603)
Other leukemia	0.268 (0.0257 to 0.702)	0.334 (0.0629 to 0.773)	0.452 (0.118 to 0.993)
Other malignant neoplasms	1.05 (0.564 to 1.67)	1.42 (0.834 to 2.1)	2.07 (1.38 to 2.89)
Other neoplasms	1.8 (1.4 to 2.26)	2.05 (1.66 to 2.48)	2.66 (2.16 to 3.29)
Myelodysplastic, myeloproliferative, and other hematopoietic neoplasms	1.8 (1.4 to 2.26)	2.05 (1.66 to 2.48)	2.66 (2.16 to 3.29)

	Years Lived Caregiving (YLC) rate per 100 000		
	2000	2021	2050
Benign and in situ intestinal neoplasms	0 (0 to 0)	0 (0 to 0)	0 (0 to 0)
Benign and in situ cervical and uterine neoplasms	0 (0 to 0)	0 (0 to 0)	0 (0 to 0)
Other benign and in situ neoplasms	0 (0 to 0)	0 (0 to 0)	0 (0 to 0)
Cardiovascular diseases	180 (169 to 189)	213 (203 to 224)	292 (278 to 305)
Rheumatic heart disease	1.76 (1.27 to 2.36)	1.57 (1.08 to 2.16)	1.96 (1.43 to 2.52)
Ischemic heart disease	28.2 (26.7 to 30)	31.1 (29 to 33)	41.8 (39.5 to 44.2)
Stroke	63.3 (57.6 to 68.6)	68.4 (61.9 to 74.4)	91.1 (83.9 to 98.9)
Ischemic stroke	52.9 (48 to 57.9)	57.8 (52.2 to 63.3)	79 (71.9 to 85.2)
Intracerebral hemorrhage	6.43 (4.87 to 8.23)	6.19 (4.65 to 8.33)	7.09 (5.32 to 9)
Subarachnoid hemorrhage	4.01 (2.78 to 5.4)	4.38 (3.14 to 5.78)	5.02 (3.67 to 6.53)
Hypertensive heart disease	4.4 (3.5 to 5.59)	7.56 (6.14 to 8.99)	12.3 (10.3 to 14.4)
Non-rheumatic valvular heart disease	4.42 (3.8 to 4.97)	7.12 (6.43 to 7.83)	13.2 (12.2 to 14.3)
Non-rheumatic calcific aortic valve disease	2.28 (1.87 to 2.63)	4.14 (3.71 to 4.64)	9.03 (8.28 to 9.73)
Non-rheumatic degenerative mitral valve disease	2.14 (1.78 to 2.47)	2.97 (2.5 to 3.45)	4.17 (3.62 to 4.73)
Other non-rheumatic valve diseases	0.00436 (0 to 0.0291)	0.0134 (0 to 0.125)	0.0154 (0 to 0.13)
Cardiomyopathy and myocarditis	2.59 (1.75 to 3.35)	2.99 (2.11 to 3.93)	3.6 (2.78 to 4.58)
Myocarditis	0.11 (0.00542 to 0.274)	0.0936 (0.00454 to 0.24)	0.112 (0.0159 to 0.248)
Alcoholic cardiomyopathy	0.405 (0.109 to 0.839)	0.436 (0.15 to 0.754)	0.391 (0.143 to 0.689)
Other cardiomyopathy	2.07 (1.35 to 2.79)	2.46 (1.68 to 3.28)	3.09 (2.32 to 4)
Pulmonary Arterial Hypertension	0.0836 (0 to 0.242)	0.105 (0.000473 to 0.312)	0.129 (0.0129 to 0.337)
Atrial fibrillation and flutter	33.6 (30.5 to 36.4)	42 (38.9 to 45.4)	61.5 (57.2 to 66)
Lower extremity peripheral arterial disease	4.94 (4.62 to 5.28)	5.11 (4.79 to 5.43)	6.66 (6.27 to 7.07)
Endocarditis	0.151 (0.00182 to 0.378)	0.381 (0.112 to 0.719)	0.509 (0.204 to 0.889)
Other cardiovascular and circulatory diseases	36.4 (33.3 to 39.7)	47.1 (43.3 to 50.7)	58.8 (54.5 to 63.2)
Chronic respiratory diseases	82.7 (78.8 to 86.3)	85.5 (81.1 to 89.8)	99.8 (94.3 to 105)
Chronic obstructive pulmonary disease	46.2 (43.5 to 49.1)	59.5 (55.9 to 63.1)	83.7 (78.9 to 88.3)
Pneumoconiosis	0.255 (0.0165 to 0.703)	0.213 (0.0104 to 0.538)	0.216 (0.0112 to 0.634)
Silicosis	0.152 (0 to 0.593)	0.0891 (0 to 0.39)	0.0824 (0 to 0.372)
Asbestosis	0.0419 (0 to 0.26)	0.0722 (0 to 0.321)	0.0906 (0 to 0.366)
Coal workers pneumoconiosis	0.0372 (0 to 0.192)	0.034 (0 to 0.183)	0.0291 (0 to 0.173)
Other pneumoconiosis	0.0238 (0 to 0.157)	0.0171 (0 to 0.109)	0.0139 (0 to 0.113)
Asthma	34.5 (32.1 to 36.9)	23.4 (21.6 to 24.7)	12.8 (11.5 to 14.3)
Interstitial lung disease and pulmonary sarcoidosis	1.74 (1.15 to 2.47)	2.47 (1.69 to 3.32)	3.06 (2.24 to 3.96)
Digestive diseases	57.2 (54.5 to 60.1)	61.9 (59 to 64.8)	65.7 (61.8 to 69.6)
Cirrhosis and other chronic liver diseases	1.79 (1.48 to 2.16)	1.67 (1.36 to 1.97)	1.83 (1.47 to 2.21)
Chronic hepatitis B including cirrhosis	0.227 (0.192 to 0.263)	0.192 (0.159 to 0.221)	0.143 (0.108 to 0.177)

	Years Lived Caregiving (YLC) rate per 100 000		
	2000	2021	2050
Chronic hepatitis C including cirrhosis	0.394 (0.343 to 0.446)	0.362 (0.314 to 0.414)	0.362 (0.314 to 0.418)
Cirrhosis due to alcohol	0.681 (0.41 to 0.988)	0.624 (0.368 to 0.892)	0.724 (0.458 to 1.03)
Nonalcoholic fatty liver disease including cirrhosis	0.285 (0.269 to 0.303)	0.307 (0.294 to 0.32)	0.372 (0.356 to 0.389)
Cirrhosis due to other causes	0.202 (0.0575 to 0.366)	0.186 (0.0456 to 0.361)	0.229 (0.0846 to 0.471)
Upper digestive system diseases	17.6 (16.6 to 18.6)	20.2 (19.1 to 21.2)	20.1 (19.1 to 21.2)
Peptic ulcer disease	0.857 (0.552 to 1.19)	0.744 (0.437 to 1.07)	0.619 (0.331 to 0.876)
Gastritis and duodenitis	3.86 (3.21 to 4.64)	4.39 (3.75 to 5.02)	3.25 (2.62 to 3.8)
Gastroesophageal reflux disease	12.9 (12.3 to 13.5)	15.1 (14.4 to 15.7)	16.2 (15.5 to 16.9)
Appendicitis	0.12 (0 to 0.578)	0.141 (0 to 0.687)	0.111 (0 to 0.476)
Paralytic ileus and intestinal obstruction	0.584 (0.0482 to 1.44)	0.716 (0.121 to 1.47)	0.998 (0.25 to 1.92)
Inguinal, femoral, and abdominal hernia	2.73 (2.09 to 3.39)	2.15 (1.48 to 2.88)	1.98 (1.43 to 2.6)
Inflammatory bowel disease	3.38 (2.32 to 4.86)	4.29 (2.96 to 6)	4.05 (2.78 to 5.32)
Vascular intestinal disorders	0.539 (0.0581 to 1.34)	0.67 (0.0802 to 1.59)	0.891 (0.21 to 1.84)
Gallbladder and biliary diseases	29.3 (27.7 to 30.8)	30.7 (29.2 to 32.5)	34.3 (32.4 to 36.3)
Pancreatitis	1.24 (0.822 to 1.69)	1.28 (0.91 to 1.73)	1.42 (0.988 to 1.93)
Neurological disorders	173 (162 to 185)	228 (216 to 252)	303 (285 to 326)
Alzheimer's disease and other dementias	82.1 (74.5 to 90.4)	121 (112 to 132)	184 (170 to 201)
Parkinson's disease	9.96 (7.67 to 12)	14.4 (12.2 to 16.8)	20.7 (17.6 to 23.6)
Idiopathic epilepsy	9.75 (7.47 to 12.7)	12.7 (10.1 to 15.6)	19.9 (16.3 to 23.7)
Multiple sclerosis	3.09 (1.63 to 4.67)	4.2 (2.62 to 6.1)	4.23 (2.59 to 6.03)
Motor neuron disease	0.433 (0.0627 to 1.05)	0.616 (0.105 to 1.45)	0.706 (0.154 to 1.51)
Headache disorders	66.1 (62.8 to 69.1)	73.1 (69.7 to 76.3)	71.8 (68.6 to 74.8)
Migraine	56.5 (53.6 to 59.4)	62.4 (59.2 to 65.6)	61 (58 to 63.9)
Tension-type headache	9.62 (9.19 to 9.97)	10.7 (10.3 to 11.1)	10.8 (10.4 to 11.3)
Other neurological disorders	1.04 (0 to 8.12)	1.71 (0 to 22.6)	1.52 (0 to 12.9)
Mental disorders	226 (213 to 239)	275 (260 to 289)	278 (264 to 293)
Schizophrenia	19.4 (14.1 to 25.1)	21.3 (15.6 to 27.5)	21 (15.8 to 27.2)
Depressive disorders	90.6 (83.6 to 98)	110 (103 to 119)	113 (105 to 122)
Major depressive disorder	75.2 (69 to 82.9)	92.3 (84.5 to 101)	94.3 (87 to 102)
Dysthymia	15.4 (13.4 to 17.6)	18 (16.2 to 20.1)	19.1 (16.8 to 21.3)
Bipolar disorder	18.9 (15.6 to 22.7)	21.6 (18.4 to 25.5)	21.9 (18.3 to 25.1)
Anxiety disorders	64.9 (59.3 to 70.4)	85.2 (78.8 to 92.1)	81.6 (75.8 to 87.8)
Eating disorders	0 (0 to 0)	0 (0 to 0)	0 (0 to 0)
Anorexia nervosa	0 (0 to 0)	0 (0 to 0)	0 (0 to 0)
Bulimia nervosa	0 (0 to 0)	0 (0 to 0)	0 (0 to 0)
Autism spectrum disorders	12 (9.42 to 14.4)	13.9 (11.4 to 16.5)	14.3 (11.8 to 16.9)
Attention-deficit/hyperactivity disorder	0.138 (0.0836 to 0.215)	0.157 (0.1 to 0.229)	0.129 (0.0774 to 0.182)
Conduct disorder	0 (0 to 0)	0 (0 to 0)	0 (0 to 0)
Idiopathic developmental intellectual disability	1.31 (0.894 to 1.76)	1.17 (0.796 to 1.6)	1.06 (0.737 to 1.43)

	Years Lived Caregiving (YLC) rate per 100 000		
	2000	2021	2050
Other mental disorders	18.9 (16.9 to 21)	21.9 (20 to 24.2)	24.3 (22.3 to 26.7)
Substance use disorders	27 (23.9 to 30.9)	30.1 (26.8 to 33.7)	32.7 (29.1 to 35.9)
Alcohol use disorders	22.6 (20.3 to 25.3)	24.2 (21.6 to 26.7)	25.4 (23.1 to 28.2)
Drug use disorders	4.33 (2.44 to 6.32)	5.95 (3.81 to 8.24)	7.28 (5.01 to 9.89)
Opioid use disorders	2.91 (1.26 to 4.64)	4.29 (2.31 to 6.39)	5.01 (2.95 to 7.55)
Cocaine use disorders	0.745 (0.266 to 1.37)	0.793 (0.353 to 1.34)	1.05 (0.473 to 1.79)
Amphetamine use disorders	0.337 (0.0658 to 0.736)	0.414 (0.1 to 0.869)	0.752 (0.253 to 1.41)
Cannabis use disorders	0.18 (0.0702 to 0.314)	0.217 (0.105 to 0.366)	0.197 (0.101 to 0.331)
Other drug use disorders	0.159 (0.0116 to 0.441)	0.229 (0.0351 to 0.529)	0.262 (0.0547 to 0.589)
Diabetes and kidney diseases	130 (123 to 136)	203 (195 to 211)	324 (312 to 337)
Diabetes mellitus	101 (95.2 to 106)	162 (155 to 169)	252 (242 to 261)
Diabetes mellitus type 1	4.55 (3.59 to 5.66)	8.36 (7.08 to 9.6)	10.2 (8.81 to 11.8)
Diabetes mellitus type 2	96.2 (90.8 to 101)	154 (147 to 161)	242 (232 to 251)
Chronic kidney disease	29.7 (27.7 to 31.9)	40.8 (38.2 to 43.4)	72.4 (67.2 to 77.2)
Chronic kidney disease due to diabetes mellitus type 1	0.559 (0.225 to 0.98)	0.868 (0.451 to 1.42)	0.914 (0.527 to 1.37)
Chronic kidney disease due to diabetes mellitus type 2	7.15 (6.38 to 7.98)	9.06 (8.11 to 10)	14.4 (13 to 15.7)
Chronic kidney disease due to hypertension	4.87 (4.14 to 5.63)	6.9 (5.97 to 7.77)	13.9 (12.4 to 15.6)
Chronic kidney disease due to glomerulonephritis	2.41 (1.42 to 3.67)	3.22 (2.05 to 4.61)	5.03 (3.36 to 6.86)
Chronic kidney disease due to other and unspecified causes	14.7 (13.7 to 15.8)	20.7 (19.4 to 22.3)	38.2 (35.4 to 40.7)
Acute glomerulonephritis	0.0012 (0 to 0.00922)	0.00153 (0 to 0.0128)	0.000973 (0 to 0.00906)
Skin and subcutaneous diseases	57.7 (54.7 to 60.8)	69.8 (66.8 to 73.5)	73.1 (69.4 to 76.6)
Dermatitis	9.68 (8.69 to 10.7)	11.3 (10.3 to 12.4)	12.6 (11.4 to 13.8)
Atopic dermatitis	6 (5.14 to 6.93)	7.09 (6.15 to 7.94)	8 (7.03 to 9.03)
Contact dermatitis	3.17 (2.71 to 3.7)	3.6 (3.11 to 4.09)	3.91 (3.32 to 4.47)
Seborrhoeic dermatitis	0.511 (0.378 to 0.69)	0.616 (0.442 to 0.794)	0.72 (0.572 to 0.867)
Psoriasis	14.3 (12.2 to 16.3)	16.9 (15.1 to 19)	16.8 (14.6 to 19.1)
Bacterial skin diseases	0.634 (0.374 to 0.909)	0.761 (0.485 to 1.09)	1.3 (0.939 to 1.77)
Cellulitis	0.441 (0.196 to 0.725)	0.521 (0.247 to 0.841)	0.734 (0.403 to 1.18)
Pyoderma	0.194 (0.135 to 0.259)	0.241 (0.18 to 0.302)	0.564 (0.471 to 0.667)
Scabies	0.639 (0.483 to 0.804)	0.733 (0.581 to 0.884)	0.843 (0.657 to 1.03)
Fungal skin diseases	8.98 (8.39 to 9.42)	11.4 (10.9 to 12)	14.9 (14.2 to 15.7)
Viral skin diseases	4.85 (4.19 to 5.56)	5.52 (4.72 to 6.27)	5.66 (4.97 to 6.41)
Acne vulgaris	1.08 (0.832 to 1.36)	1.25 (1 to 1.53)	1.28 (1.03 to 1.57)
Alopecia areata	0.77 (0.529 to 1.03)	0.854 (0.624 to 1.12)	0.866 (0.608 to 1.13)
Pruritus	1.4 (1.22 to 1.62)	1.72 (1.5 to 1.94)	2 (1.77 to 2.23)
Urticaria	3.85 (3.12 to 4.65)	4.36 (3.63 to 5.22)	4.61 (3.8 to 5.53)
Decubitus ulcer	0.361 (0.0877 to 0.834)	0.479 (0.12 to 0.964)	0.567 (0.204 to 1.04)
Other skin and subcutaneous diseases	11.2 (10.7 to 11.7)	14.5 (13.9 to 15.1)	11.6 (11 to 12.2)
Sense organ diseases	210 (201 to 220)	262 (251 to 272)	339 (323 to 352)

	Years Lived Caregiving (YLC) rate per 100 000		
	2000	2021	2050
Blindness and vision loss	64.1 (59.8 to 68.3)	76.9 (72.5 to 81.7)	96.2 (90.3 to 102)
Glaucoma	5.08 (3.86 to 6.67)	5.86 (4.4 to 7.32)	8.21 (6.27 to 9.96)
Cataract	13.8 (12.2 to 15.7)	18 (16.1 to 20)	24.5 (22.5 to 27)
Age-related macular degeneration	5.83 (4.56 to 7.27)	6.62 (5.29 to 8.31)	9.2 (7.44 to 11.2)
Refraction disorders	11.6 (10.3 to 13.1)	13.8 (12.4 to 15.1)	16.8 (15.4 to 18.4)
Near vision loss	20.5 (19.1 to 21.7)	24.3 (22.8 to 25.6)	27.1 (25.6 to 28.6)
Other vision loss	7.39 (6.16 to 8.63)	8.3 (6.96 to 9.49)	10.4 (9.12 to 11.9)
Age-related and other hearing loss	130 (125 to 137)	167 (160 to 173)	220 (212 to 229)
Other sense organ diseases	15.1 (13.9 to 16.6)	18.4 (17.2 to 19.8)	22 (20.3 to 23.7)
Musculoskeletal disorders	483 (466 to 502)	573 (554 to 592)	647 (626 to 666)
Rheumatoid arthritis	9.78 (7.94 to 11.5)	12.1 (9.91 to 14)	15.5 (13.2 to 17.9)
Osteoarthritis	107 (103 to 112)	135 (129 to 140)	161 (155 to 167)
Osteoarthritis hip	9.13 (8.2 to 10.2)	11.8 (10.8 to 12.8)	15.2 (13.9 to 16.5)
Osteoarthritis knee	54.7 (51.9 to 57.7)	66.9 (64 to 70.2)	79.5 (75.9 to 82.7)
Osteoarthritis hand	33.1 (31.1 to 35.1)	43.1 (41.1 to 45.3)	50.4 (47.8 to 53)
Osteoarthritis other	10.2 (9.12 to 11.4)	12.8 (11.8 to 14.1)	15.9 (14.7 to 17.2)
Low back pain	262 (251 to 274)	300 (288 to 312)	326 (313 to 338)
Neck pain	50.8 (46.6 to 55.1)	57.7 (53.1 to 62.3)	61 (56.8 to 65.5)
Gout	5.56 (4.85 to 6.41)	7.43 (6.62 to 8.25)	9.39 (8.43 to 10.4)
Other musculoskeletal disorders	47.9 (43.5 to 51.9)	61.6 (57.5 to 65.4)	74.2 (69.9 to 79.6)
Other non-communicable diseases	160 (154 to 166)	178 (173 to 184)	199 (191 to 206)
Congenital birth defects	3.9 (2.93 to 5.27)	4.51 (3.38 to 5.89)	4.5 (3.28 to 5.74)
Neural tube defects	0.144 (0 to 0.508)	0.216 (0 to 0.772)	0.234 (0.00205 to 0.738)
Congenital heart anomalies	0.869 (0.563 to 1.27)	1.03 (0.716 to 1.45)	1.12 (0.768 to 1.52)
Orofacial clefts	0.156 (0.0341 to 0.379)	0.158 (0.033 to 0.336)	0.164 (0.0387 to 0.362)
Down syndrome	0.128 (0 to 0.424)	0.152 (0 to 0.489)	0.138 (0 to 0.453)
Turner syndrome	0.00606 (0 to 0.0278)	0.00783 (0 to 0.0347)	0.00588 (0 to 0.0246)
Klinefelter syndrome	0.00128 (0 to 0.00577)	0.00138 (0 to 0.00491)	0.00145 (0 to 0.00572)
Other chromosomal abnormalities	0.177 (0 to 0.5)	0.195 (0.00779 to 0.55)	0.177 (0.00119 to 0.538)
Congenital musculoskeletal and limb anomalies	1.73 (0.954 to 2.7)	1.9 (1.17 to 2.82)	1.86 (1.07 to 2.71)
Urogenital congenital anomalies	0.00579 (0 to 0.047)	0.00897 (0 to 0.0541)	0.00688 (0 to 0.0454)
Digestive congenital anomalies	0.0718 (0.00394 to 0.199)	0.0802 (0.00344 to 0.212)	0.1 (0.0145 to 0.256)
Other congenital birth defects	0.614 (0.169 to 1.2)	0.76 (0.313 to 1.53)	0.691 (0.243 to 1.27)
Urinary diseases and male infertility	10.7 (9.77 to 11.8)	13.4 (12.4 to 14.4)	16.2 (15.2 to 17.5)
Urinary tract infections and interstitial nephritis	0.38 (0.193 to 0.588)	0.435 (0.248 to 0.646)	0.594 (0.411 to 0.811)
Urolithiasis	0.77 (0.456 to 1.19)	0.759 (0.437 to 1.15)	0.696 (0.376 to 1.12)
Benign prostatic hyperplasia	9.6 (8.86 to 10.5)	12.2 (11.3 to 13)	14.9 (14.1 to 16)
Male infertility	0 (0 to 0)	0 (0 to 0)	0 (0 to 0)
Gynecological diseases	40 (37.9 to 42.4)	42.8 (40.4 to 45.3)	37.8 (34.7 to 41.3)
Uterine fibroids	0.0914 (0.0819 to 0.102)	0.0943 (0.085 to 0.107)	0.0454 (0.0386 to 0.0535)

	Years Lived Caregiving (YLC) rate per 100 000		
	2000	2021	2050
Polycystic ovarian syndrome	0.064 (0.0266 to 0.109)	0.0705 (0.0314 to 0.127)	0.0582 (0.0228 to 0.0956)
Female infertility	0 (0 to 0)	0 (0 to 0)	0 (0 to 0)
Endometriosis	0.239 (0.0527 to 0.489)	0.263 (0.0553 to 0.539)	0.0682 (0.000481 to 0.241)
Genital prolapse	1.89 (1.75 to 2.09)	2.3 (2.11 to 2.47)	0.741 (0.615 to 0.871)
Premenstrual syndrome	0 (0 to 0)	0 (0 to 0)	0 (0 to 0)
Other gynecological diseases	36.8 (34.6 to 39.2)	39.1 (36.8 to 41.4)	36.2 (33 to 39.8)
Hemoglobinopathies and hemolytic anemias	2.02 (1.79 to 2.28)	2.14 (1.9 to 2.41)	2.6 (2.22 to 2.97)
Thalassemias	0.000725 (0 to 0.012)	0.00127 (0 to 0.0138)	0.000623 (0 to 0.00476)
Thalassemias trait	0.473 (0.409 to 0.547)	0.554 (0.475 to 0.632)	0.703 (0.602 to 0.806)
Sickle cell disorders	0.00641 (0 to 0.0859)	0.011 (0 to 0.093)	0.00759 (0 to 0.0862)
Sickle cell trait	0.152 (0.131 to 0.174)	0.169 (0.144 to 0.195)	0.218 (0.188 to 0.253)
G6PD deficiency	0.00386 (0.00342 to 0.00439)	0.00457 (0.00401 to 0.00515)	0.00627 (0.00557 to 0.00707)
G6PD trait	0.00499 (0.00466 to 0.00534)	0.00548 (0.00518 to 0.00582)	0.00662 (0.00619 to 0.00712)
Other hemoglobinopathies and hemolytic anemias	1.38 (1.15 to 1.65)	1.4 (1.19 to 1.63)	1.66 (1.32 to 1.98)
Endocrine, metabolic, blood, and immune disorders	14.1 (12.9 to 15.2)	15.3 (14.3 to 16.4)	18.5 (17.3 to 19.8)
Oral disorders	89.6 (86.3 to 93)	99.7 (95.9 to 104)	119 (115 to 124)
Caries of deciduous teeth	0 (0 to 0)	0 (0 to 0)	0 (0 to 0)
Caries of permanent teeth	3.93 (3.74 to 4.1)	4.39 (4.22 to 4.57)	4.57 (4.43 to 4.72)
Periodontal diseases	15 (14.3 to 15.7)	16.8 (16.1 to 17.4)	17.9 (17.2 to 18.7)
Edentulism	64.3 (61.5 to 67.2)	71.4 (68 to 74.6)	89.5 (86 to 93.1)
Other oral disorders	6.41 (5.77 to 7.13)	7.2 (6.52 to 7.86)	7.41 (6.67 to 8.25)
Injuries	255 (245 to 263)	271 (261 to 281)	315 (302 to 328)
Transport injuries	37.1 (34.3 to 39.6)	24.2 (22.4 to 26.2)	25.7 (24 to 27.8)
Road injuries	34.3 (31.8 to 36.9)	21.1 (19.4 to 23)	22.3 (20.7 to 24.3)
Pedestrian road injuries	8.5 (7.39 to 9.54)	4.36 (3.53 to 5.12)	4.87 (3.93 to 5.78)
Cyclist road injuries	5.65 (4.68 to 6.53)	4.46 (3.69 to 5.25)	4.6 (3.81 to 5.32)
Motorcyclist road injuries	4.39 (3.69 to 5.18)	3.9 (3.25 to 4.53)	4.21 (3.5 to 5.07)
Motor vehicle road injuries	14.7 (13 to 16.5)	7.55 (6.49 to 8.62)	7.72 (6.8 to 8.83)
Other road injuries	1.13 (0.767 to 1.46)	0.837 (0.554 to 1.14)	0.882 (0.594 to 1.21)
Other transport injuries	2.76 (2.18 to 3.35)	3.1 (2.59 to 3.67)	3.45 (2.85 to 4.04)
Unintentional injuries	211 (203 to 218)	241 (231 to 250)	283 (271 to 295)
Falls	150 (144 to 156)	182 (174 to 191)	227 (215 to 239)
Drowning	0.174 (0.0769 to 0.321)	0.146 (0.05 to 0.268)	0.154 (0.0645 to 0.27)
Fire, heat, and hot substances	9.19 (8.54 to 9.89)	8.36 (7.72 to 8.98)	7.18 (6.56 to 7.85)
Poisonings	1.69 (1.18 to 2.27)	1.58 (1.12 to 2.09)	1.02 (0.591 to 1.39)
Poisoning by carbon monoxide	0.203 (0.0688 to 0.422)	0.194 (0.0746 to 0.34)	0.166 (0.0545 to 0.306)
Poisoning by other means	1.49 (1.03 to 2.06)	1.39 (0.933 to 1.89)	0.85 (0.478 to 1.19)
Exposure to mechanical forces	28.6 (27.1 to 30.1)	28.1 (26.8 to 29.4)	28.8 (27.4 to 30.1)
Unintentional firearm injuries	0.591 (0.35 to 0.811)	0.574 (0.371 to 0.791)	0.52 (0.34 to 0.783)

	Years Lived Caregiving (YLC) rate per 100 000		
	2000	2021	2050
Other exposure to mechanical forces	28.1 (26.6 to 29.6)	27.5 (26.3 to 28.8)	28.3 (26.9 to 29.6)
Adverse effects of medical treatment	0.348 (0.0684 to 0.779)	0.405 (0.124 to 0.826)	0.449 (0.117 to 0.924)
Animal contact	1.06 (0.863 to 1.3)	0.962 (0.79 to 1.21)	1.46 (1.21 to 1.78)
Venomous animal contact	0.324 (0.19 to 0.476)	0.277 (0.168 to 0.429)	0.4 (0.247 to 0.625)
Non-venomous animal contact	0.732 (0.583 to 0.905)	0.685 (0.529 to 0.84)	1.06 (0.886 to 1.28)
Foreign body	2.43 (1.98 to 2.94)	2.41 (1.94 to 2.94)	2.66 (2.1 to 3.24)
Pulmonary aspiration and foreign body in airway	0.256 (0.12 to 0.407)	0.215 (0.108 to 0.378)	0.233 (0.0928 to 0.379)
Foreign body in eyes	1.21 (0.808 to 1.68)	1.15 (0.768 to 1.62)	1.15 (0.752 to 1.58)
Foreign body in other body part	0.96 (0.747 to 1.18)	1.05 (0.839 to 1.27)	1.27 (1.01 to 1.55)
Environmental heat and cold exposure	1.86 (1.47 to 2.27)	2.34 (1.87 to 2.93)	2.8 (2.23 to 3.37)
Exposure to forces of nature	0.0244 (0.000176 to 0.0808)	0.0348 (0.000528 to 0.103)	0.0395 (0.00179 to 0.104)
Other unintentional injuries	15.1 (13.9 to 16.3)	14.1 (13 to 15.2)	11.4 (10.4 to 12.7)
Self-harm and interpersonal violence	6.82 (6.01 to 7.58)	6.07 (5.33 to 6.76)	6.39 (5.69 to 7.21)
Self-harm	1.73 (1.37 to 2.12)	1.55 (1.2 to 1.99)	1.55 (1.19 to 1.92)
Self-harm by firearm	0.0301 (0.00323 to 0.0856)	0.0246 (0.000773 to 0.072)	0.0261 (0.00194 to 0.0802)
Self-harm by other specified means	1.7 (1.34 to 2.08)	1.52 (1.18 to 1.95)	1.52 (1.18 to 1.89)
Interpersonal violence	4.96 (4.35 to 5.54)	4.34 (3.8 to 4.86)	3.76 (3.21 to 4.3)
Physical violence by firearm	0.156 (0.0565 to 0.287)	0.122 (0.0331 to 0.252)	0.0919 (0.0161 to 0.208)
Physical violence by sharp object	0.724 (0.531 to 0.942)	0.583 (0.434 to 0.784)	0.47 (0.335 to 0.652)
Sexual violence	0.574 (0.477 to 0.674)	0.639 (0.539 to 0.757)	0.638 (0.533 to 0.757)
Physical violence by other means	3.51 (2.95 to 4.07)	2.99 (2.48 to 3.5)	2.56 (2.12 to 3.09)
Conflict and terrorism	0.131 (0.0758 to 0.199)	0.18 (0.119 to 0.252)	1.07 (0.767 to 1.44)
Police conflict and executions	0.00381 (0 to 0.0377)	0.00706 (0 to 0.047)	0.0066 (0 to 0.0427)

Years Lived Caregiving by sex assigned at birth, age group, and location

Figure S6. Years Lived Caregiving for older adults, by 5-year age groups, sex, and location.

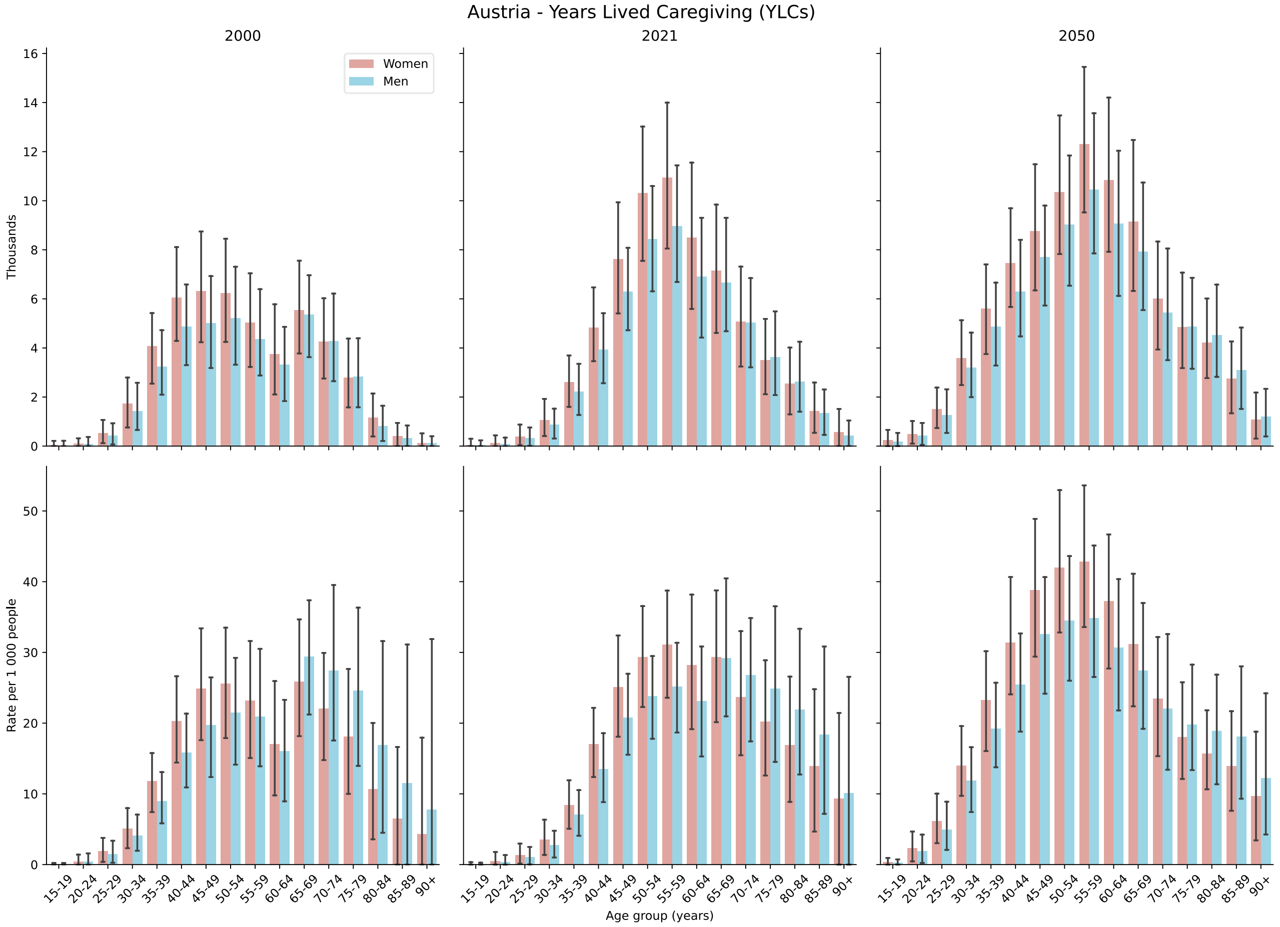

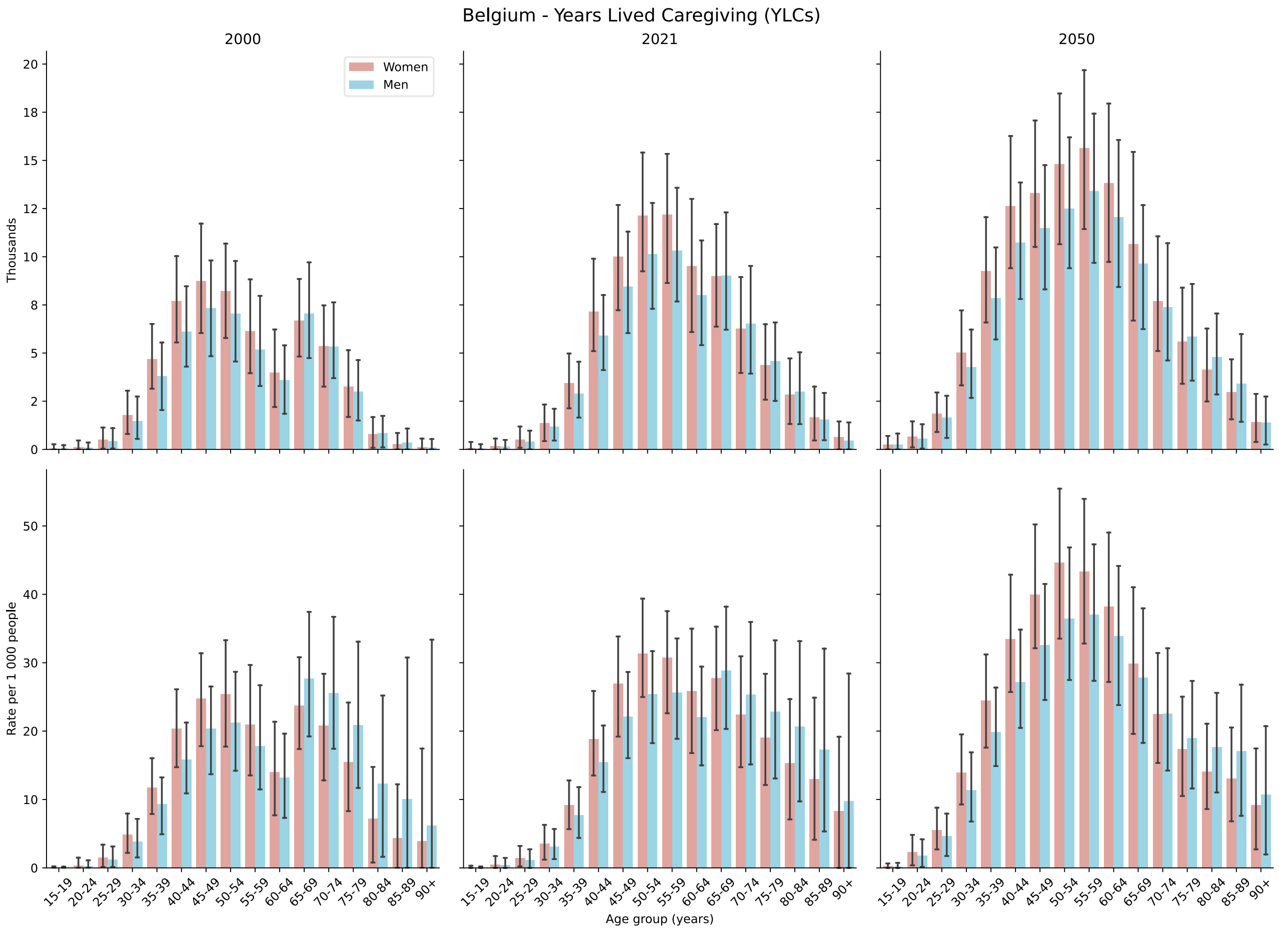
Belgium - Years Lived Caregiving (YLCs)
2000
2021
2050
Women
Men
Thousands
Rate per 1 000 people
0
2
5
8
10
12
15
18
20
30
40
50
15-19
20-24
25-29
30-34
35-39
40-44
45-49
50-54
55-59
60-64
65-69
70-74
75-79
80-84
85-89
90+
Age group (years)

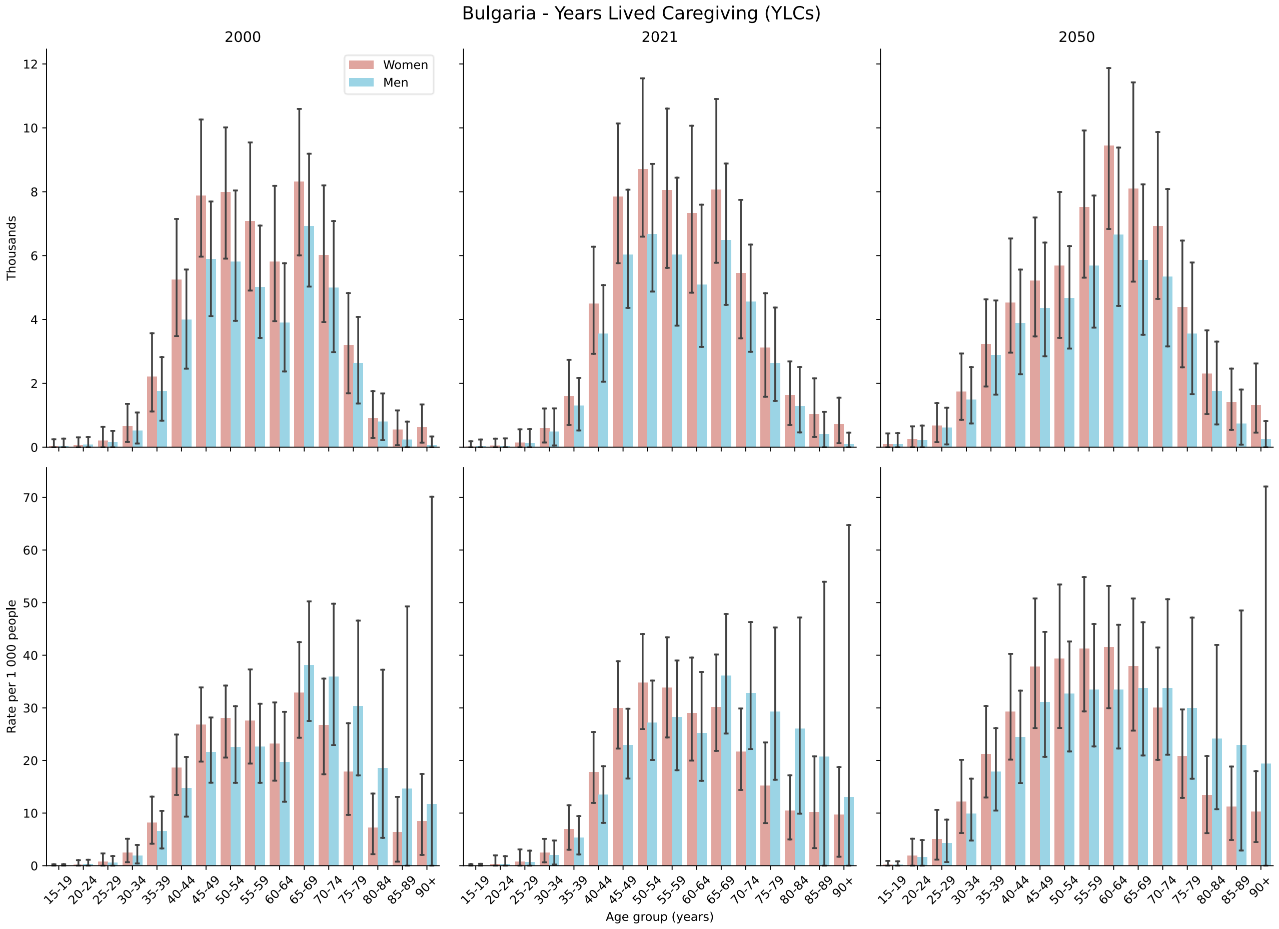
Bulgaria - Years Lived Caregiving (YLCs)
2000
2021
2050
Women
Men
Thousands
Rate per 1 000 people
0
2
4
6
8
10
12
20
30
40
50
60
70
15-19
20-24
25-29
30-34
35-39
40-44
45-49
50-54
55-59
60-64
65-69
70-74
75-79
80-84
85-89
90+
Age group (years)

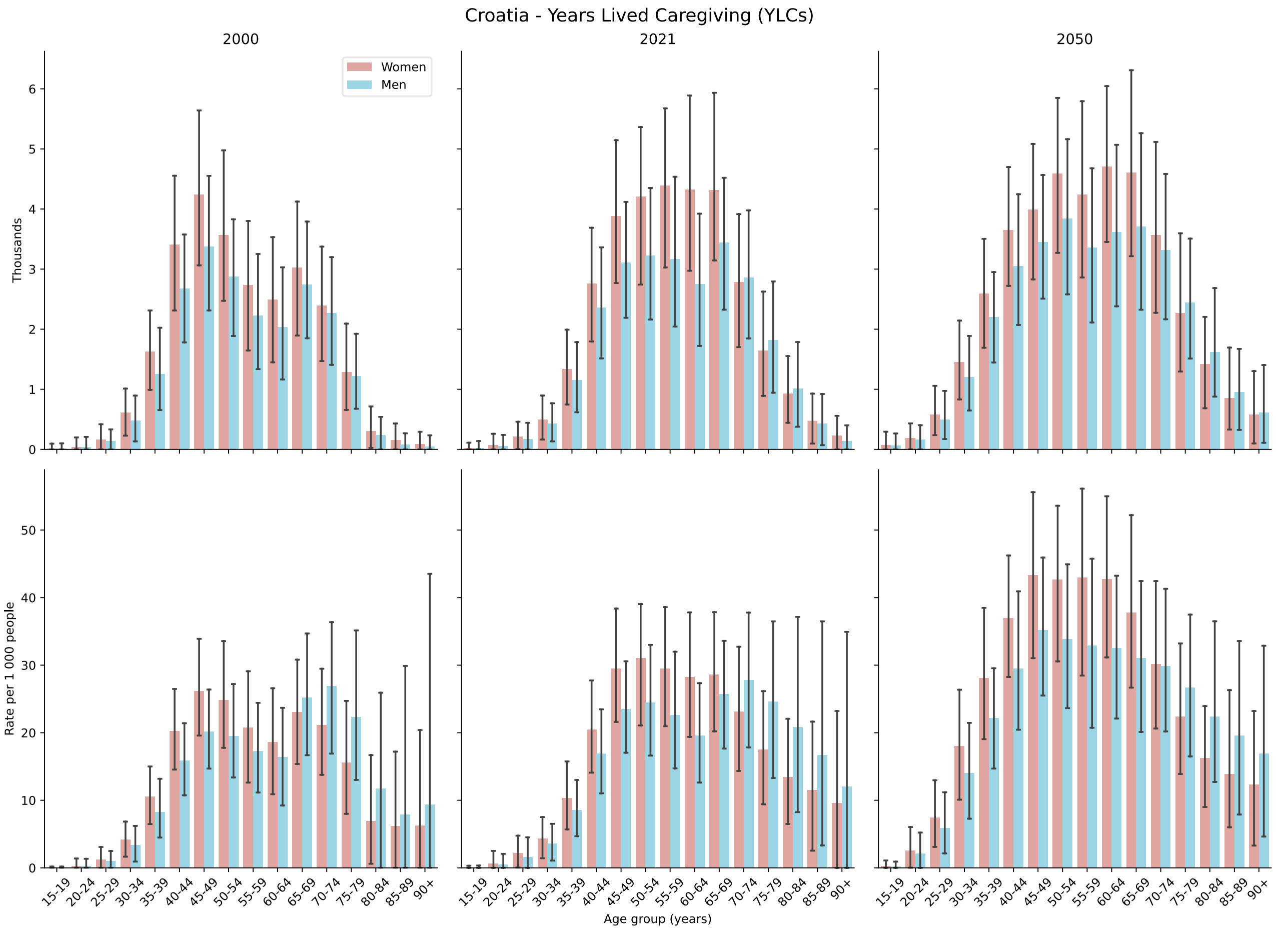
Croatia - Years Lived Caregiving (YLCs)
2000
2021
2050
Women
Men
Thousands
Rate per 1 000 people
15-19 20-24 25-29 30-34 35-39 40-44 45-49 50-54 55-59 60-64 65-69 70-74 75-79 80-84 85-89 90+
Age group (years)

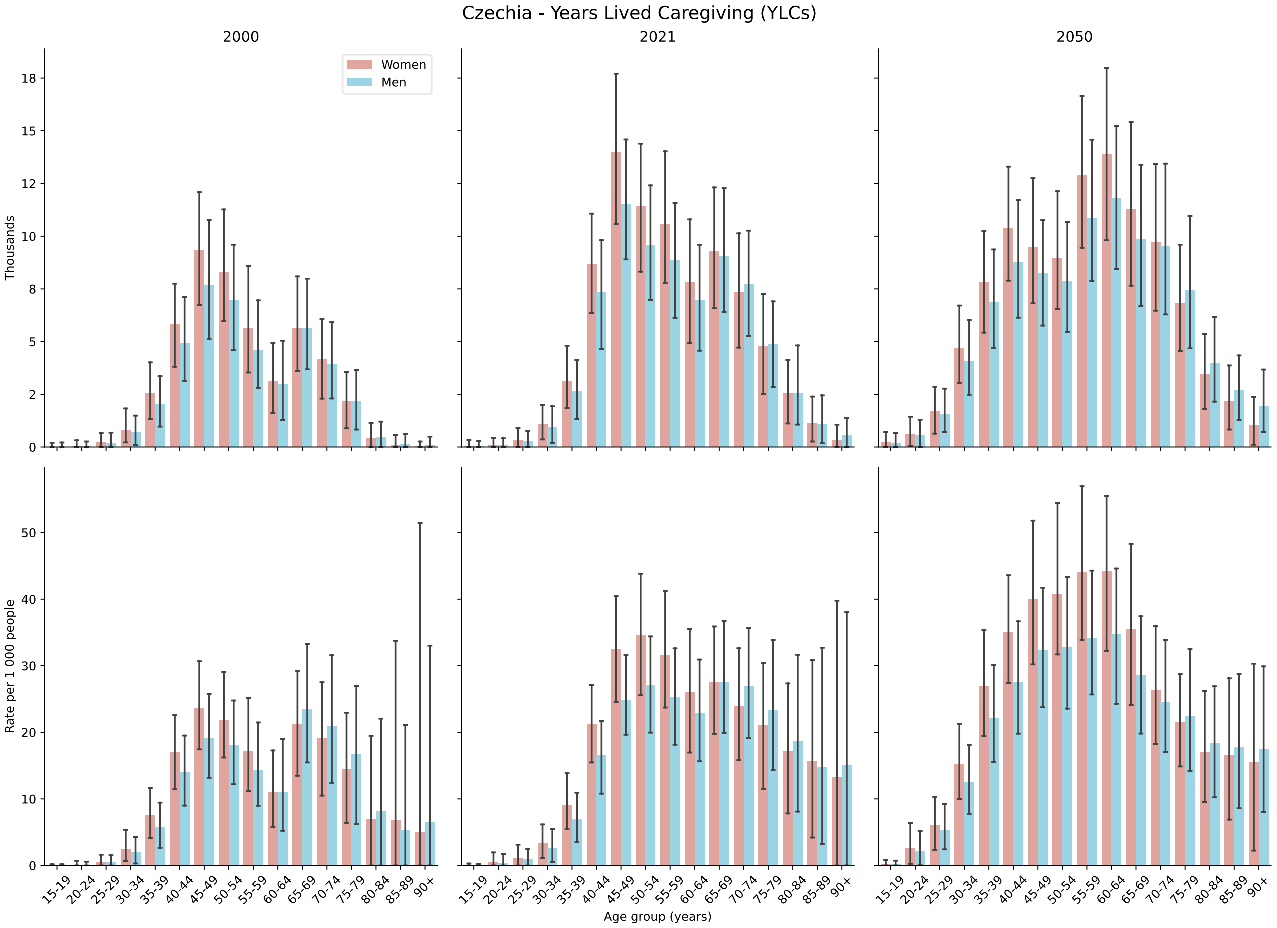
Czechia - Years Lived Caregiving (YLCs)
2000
2021
2050
Women
Men
Thousands
Rate per 1 000 people
15-19 20-24 25-29 30-34 35-39 40-44 45-49 50-54 55-59 60-64 65-69 70-74 75-79 80-84 85-89 90+
Age group (years)

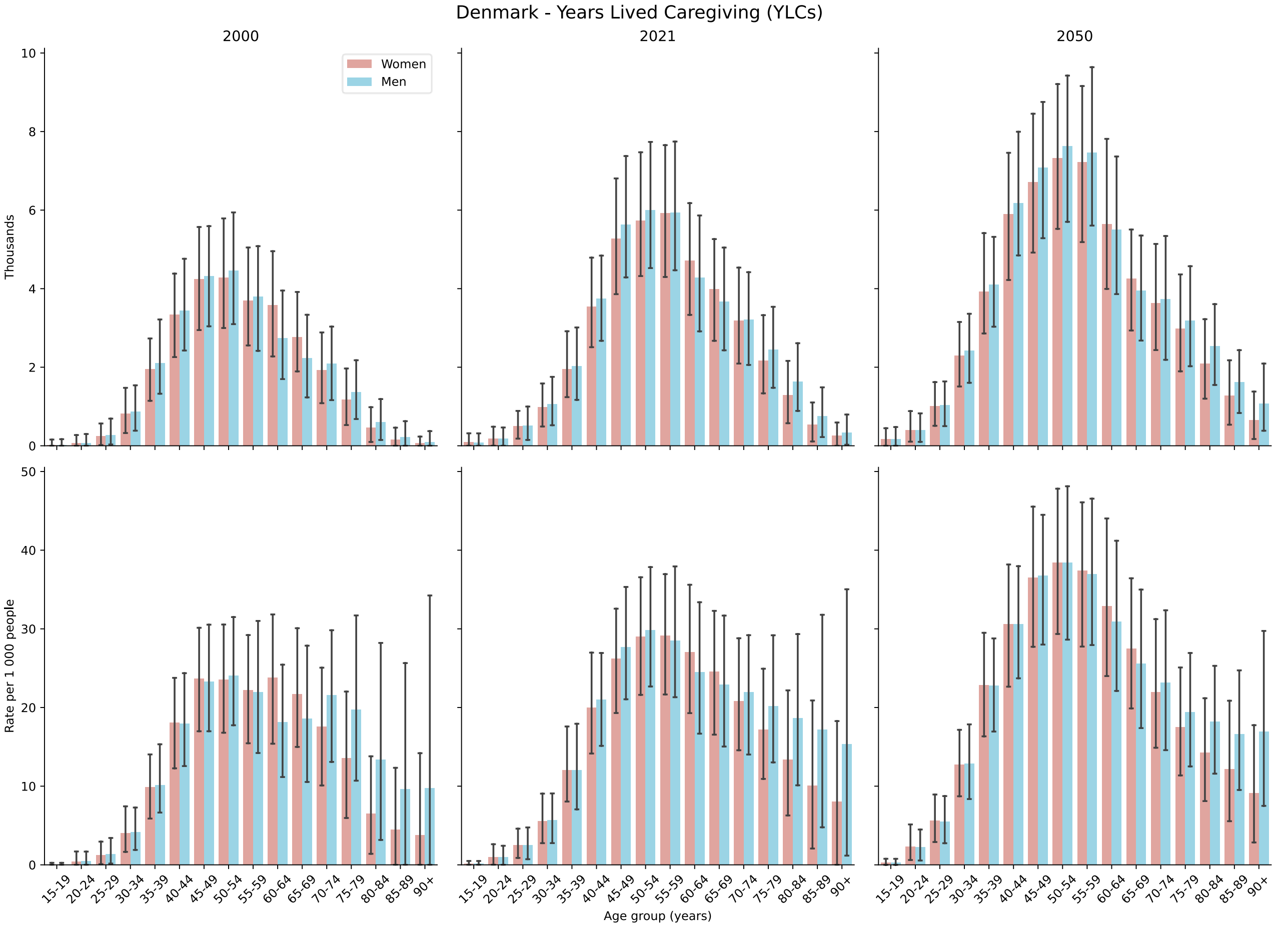
Denmark - Years Lived Caregiving (YLCs)
2000
2021
2050
Women
Men
Thousands
Rate per 1 000 people
15-19 20-24 25-29 30-34 35-39 40-44 45-49 50-54 55-59 60-64 65-69 70-74 75-79 80-84 85-89 90+
Age group (years)

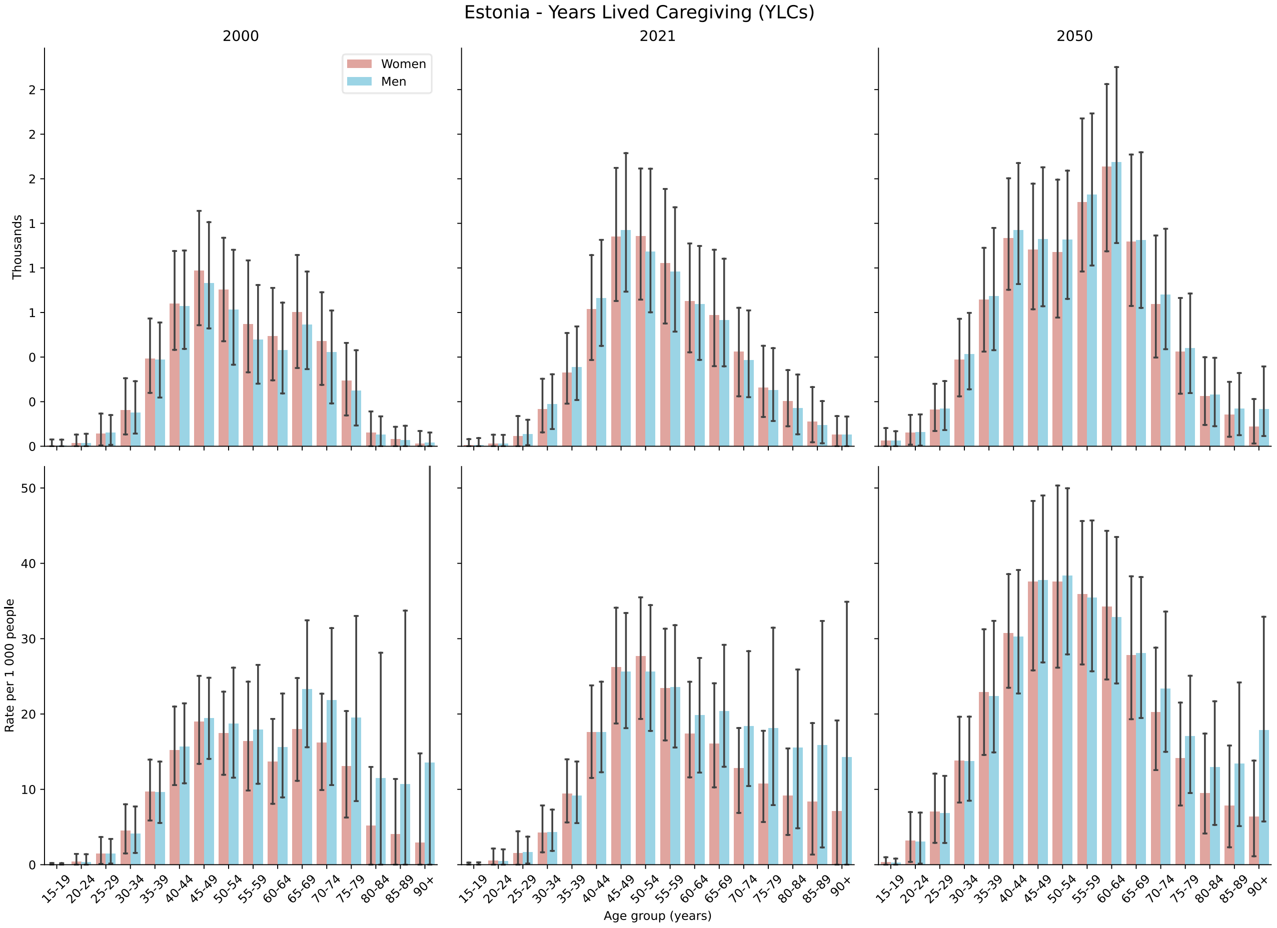
Estonia - Years Lived Caregiving (YLCs)
2000
2021
2050
Women
Men
Thousands
Rate per 1 000 people
15-19 20-24 25-29 30-34 35-39 40-44 45-49 50-54 55-59 60-64 65-69 70-74 75-79 80-84 85-89 90+
Age group (years)

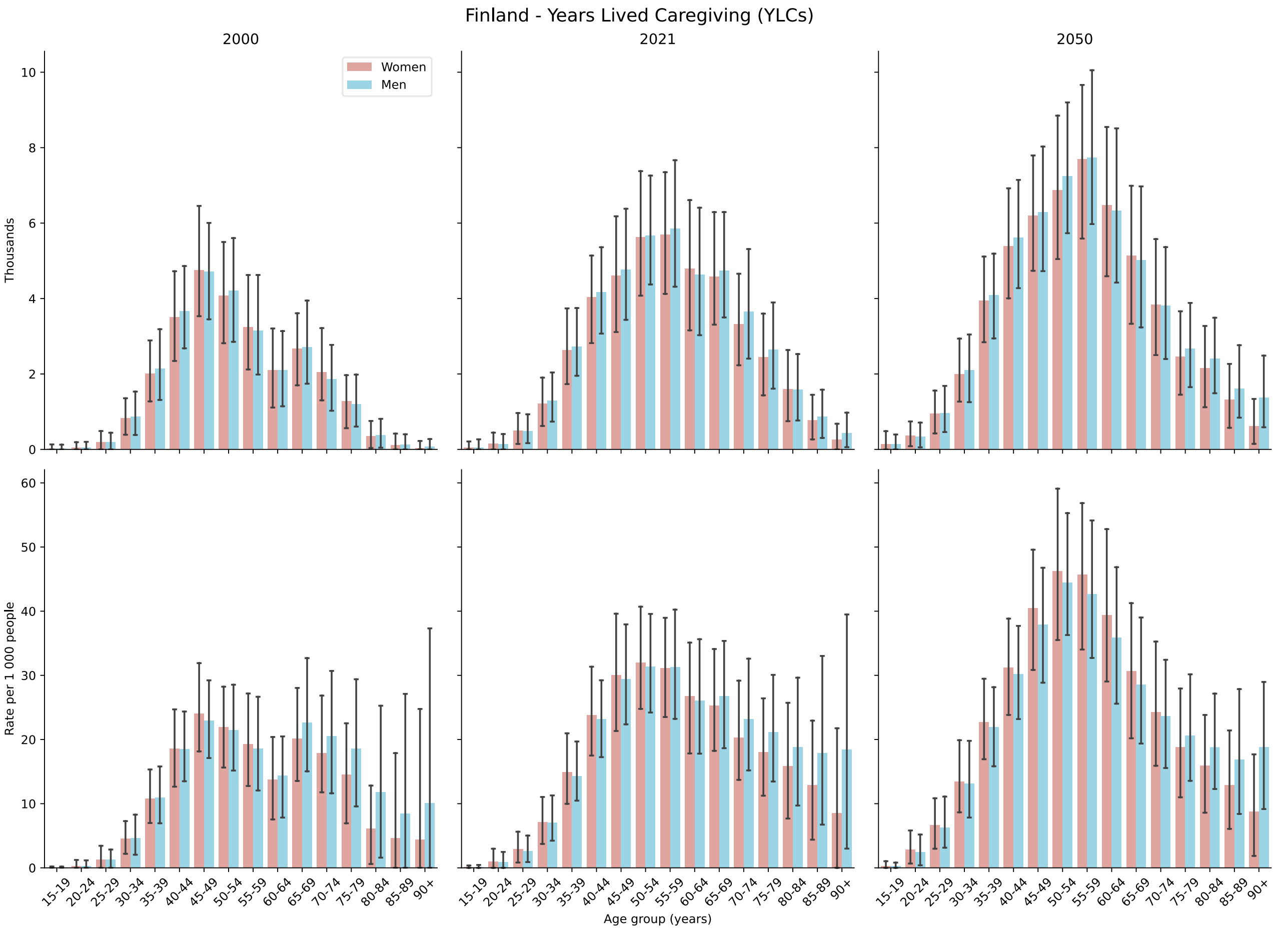
Finland - Years Lived Caregiving (YLCs)
2000
2021
2050
Women
Men
Thousands
Rate per 1 000 people
Age group (years)
15-19
20-24
25-29
30-34
35-39
40-44
45-49
50-54
55-59
60-64
65-69
70-74
75-79
80-84
85-89
90+

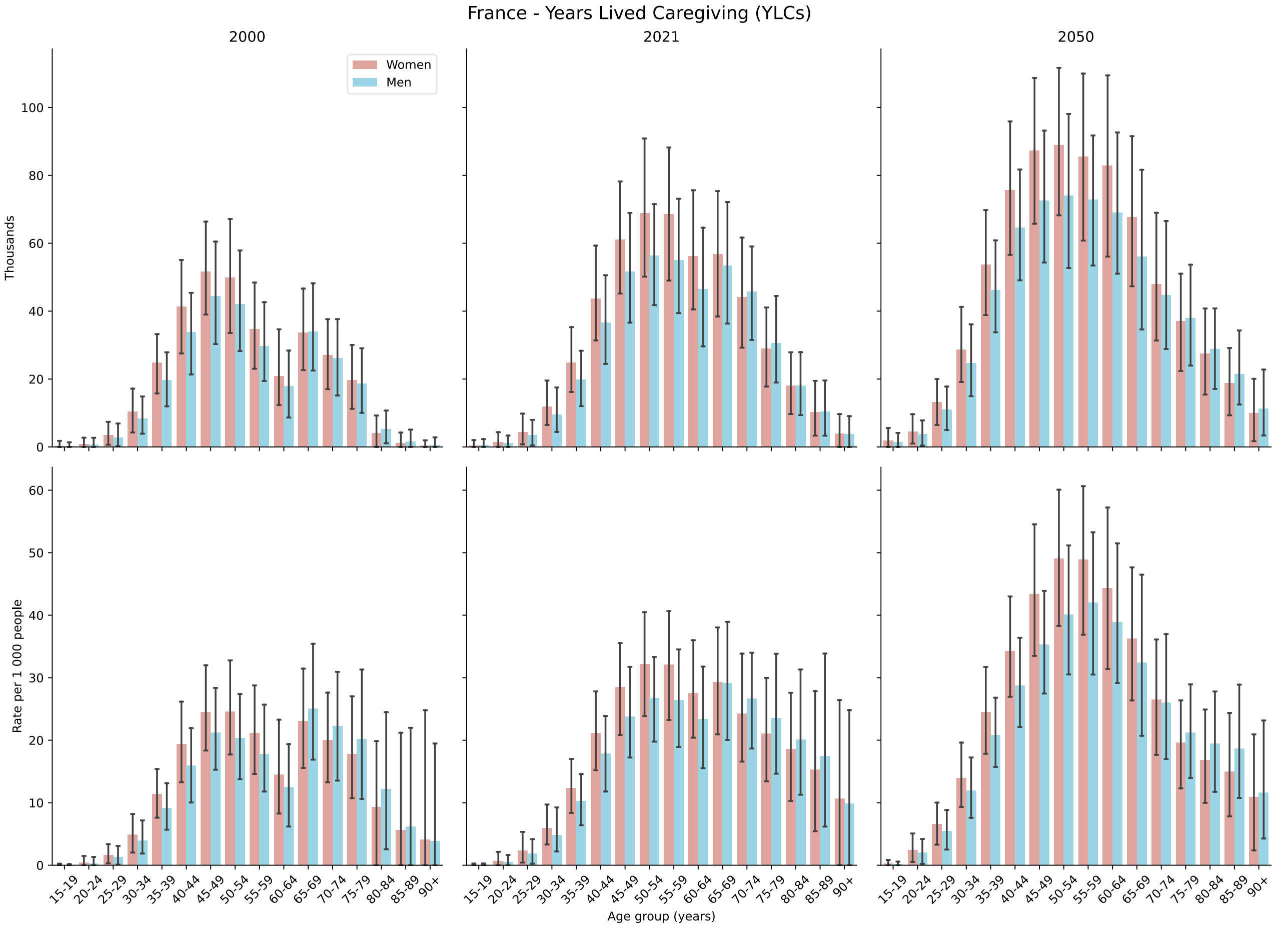
France - Years Lived Caregiving (YLCs)
2000
2021
2050
Women
Men
Thousands
Rate per 1 000 people
Age group (years)
15-19
20-24
25-29
30-34
35-39
40-44
45-49
50-54
55-59
60-64
65-69
70-74
75-79
80-84
85-89
90+

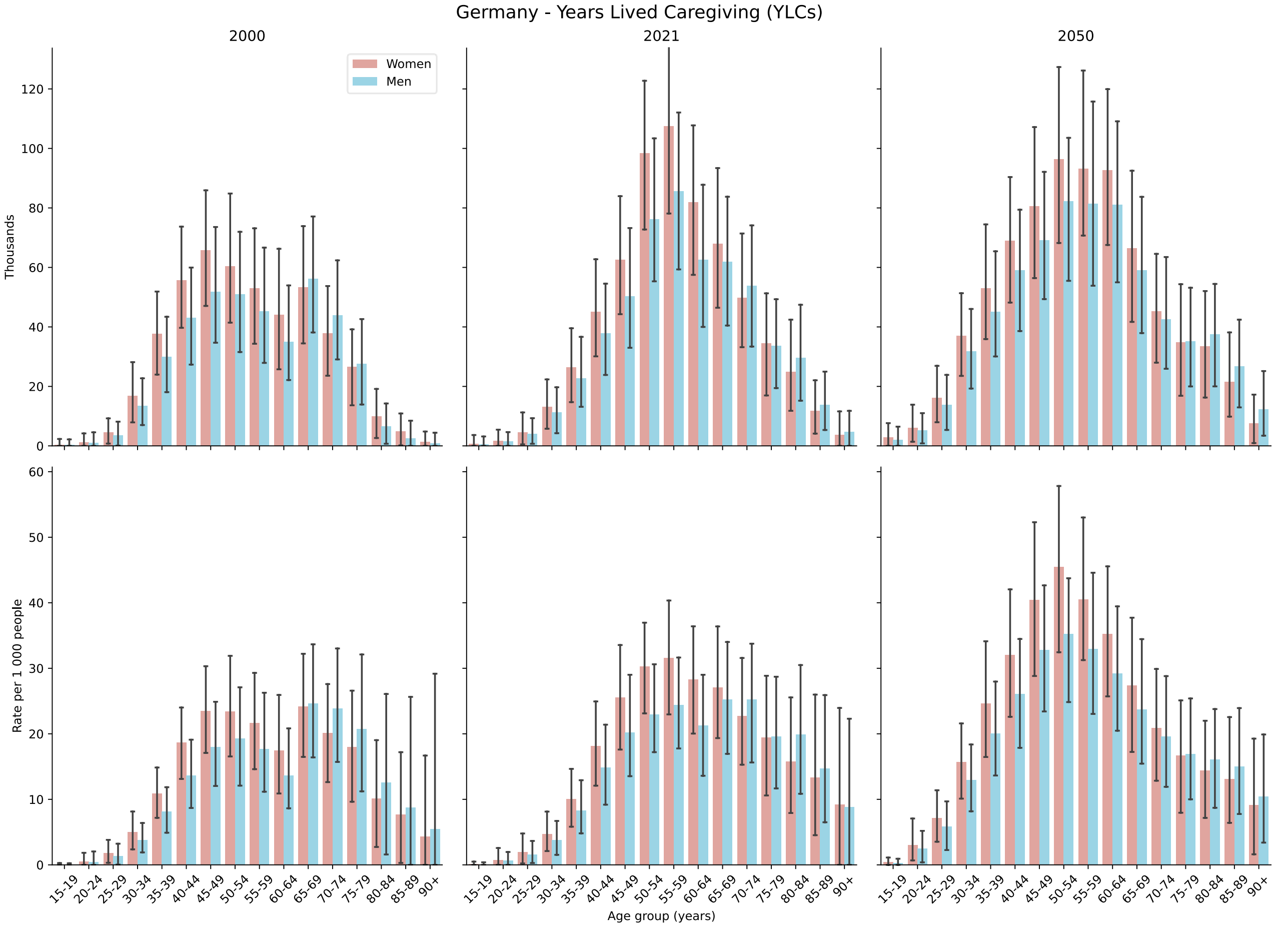

Germany - Years Lived Caregiving (YLCs)
2000
2021
2050
Women
Men
Thousands
Rate per 1 000 people
Age group (years)
15-19
20-24
25-29
30-34
35-39
40-44
45-49
50-54
55-59
60-64
65-69
70-74
75-79
80-84
85-89
90+

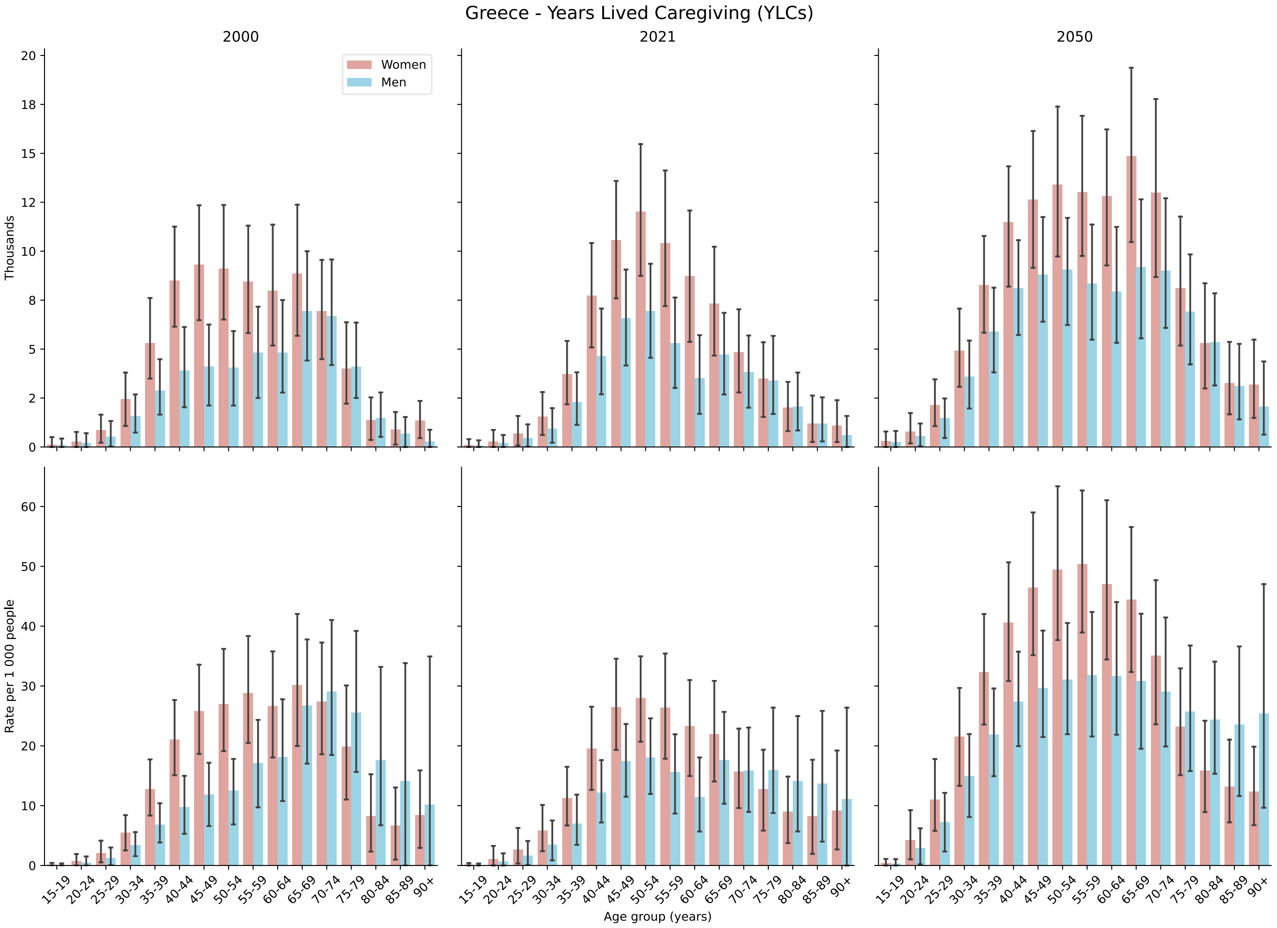

Greece - Years Lived Caregiving (YLCs)
2000
2021
2050
Women
Men
Thousands
Rate per 1 000 people
Age group (years)
15-19
20-24
25-29
30-34
35-39
40-44
45-49
50-54
55-59
60-64
65-69
70-74
75-79
80-84
85-89
90+

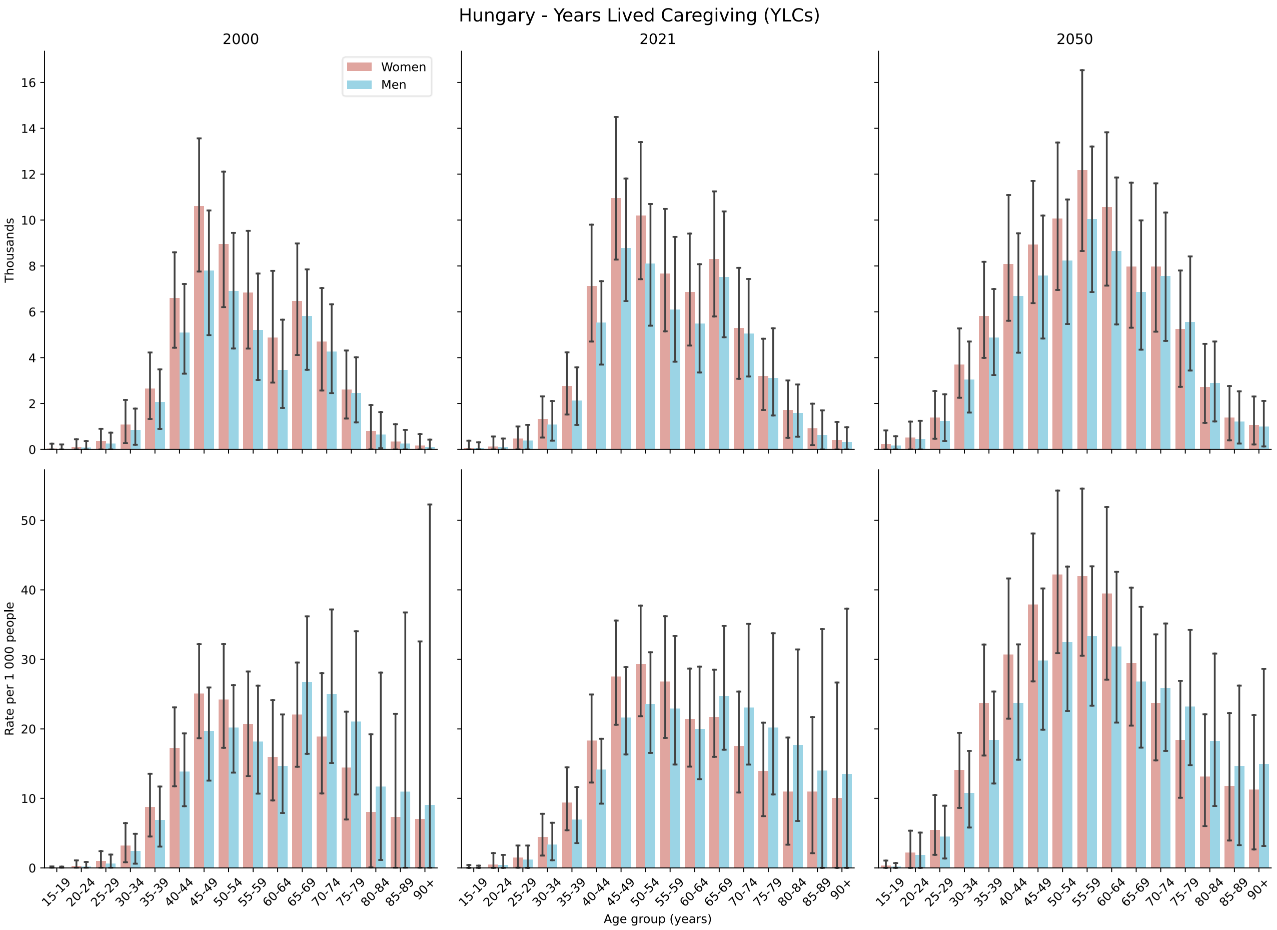
Hungary - Years Lived Caregiving (YLCs)
2000
2021
2050
Women
Men
Thousands
Rate per 1 000 people
Age group (years)
15-19
20-24
25-29
30-34
35-39
40-44
45-49
50-54
55-59
60-64
65-69
70-74
75-79
80-84
85-89
90+

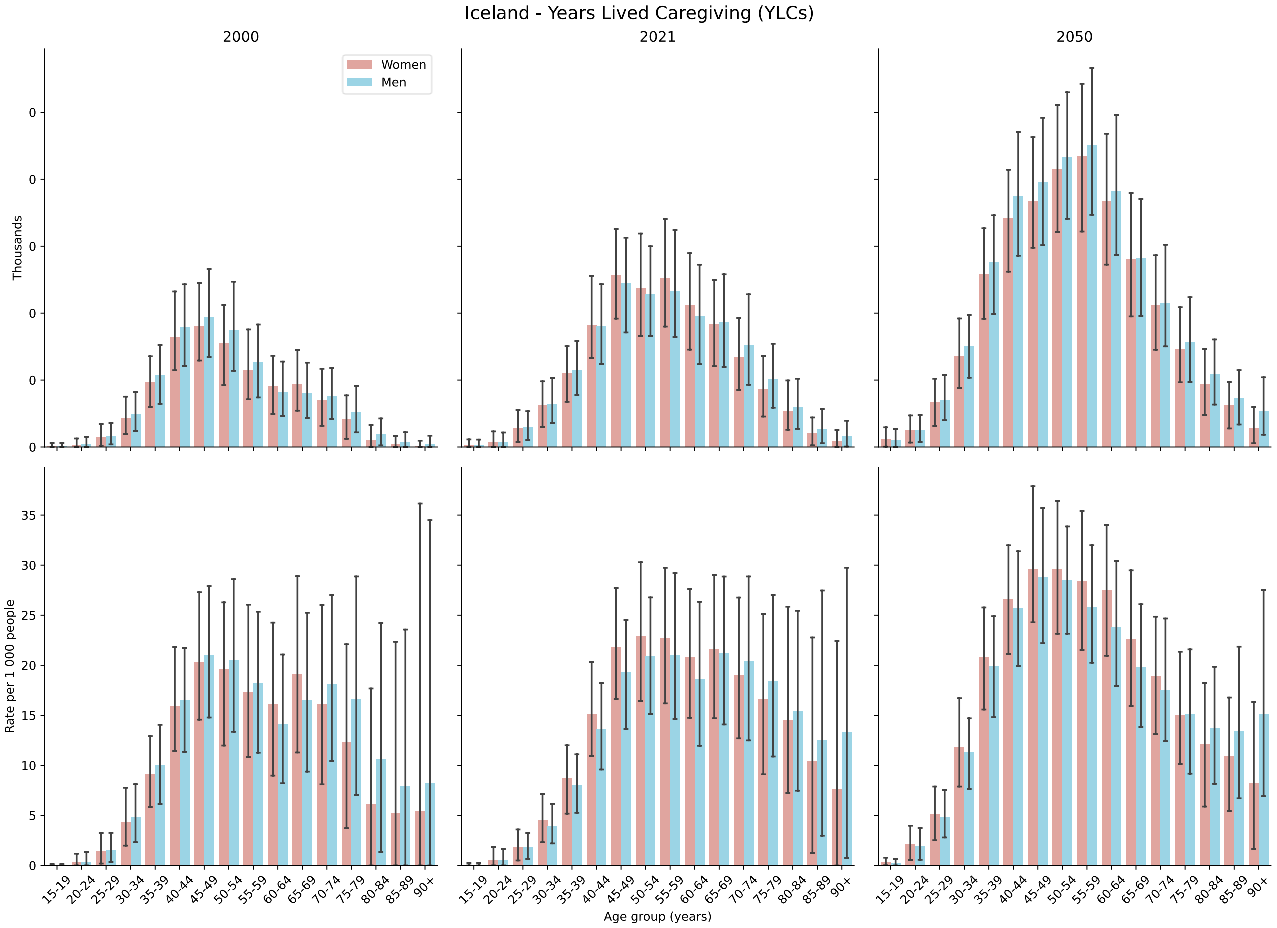
Iceland - Years Lived Caregiving (YLCs)
2000
2021
2050
Women
Men
Thousands
Rate per 1 000 people
Age group (years)
15-19
20-24
25-29
30-34
35-39
40-44
45-49
50-54
55-59
60-64
65-69
70-74
75-79
80-84
85-89
90+

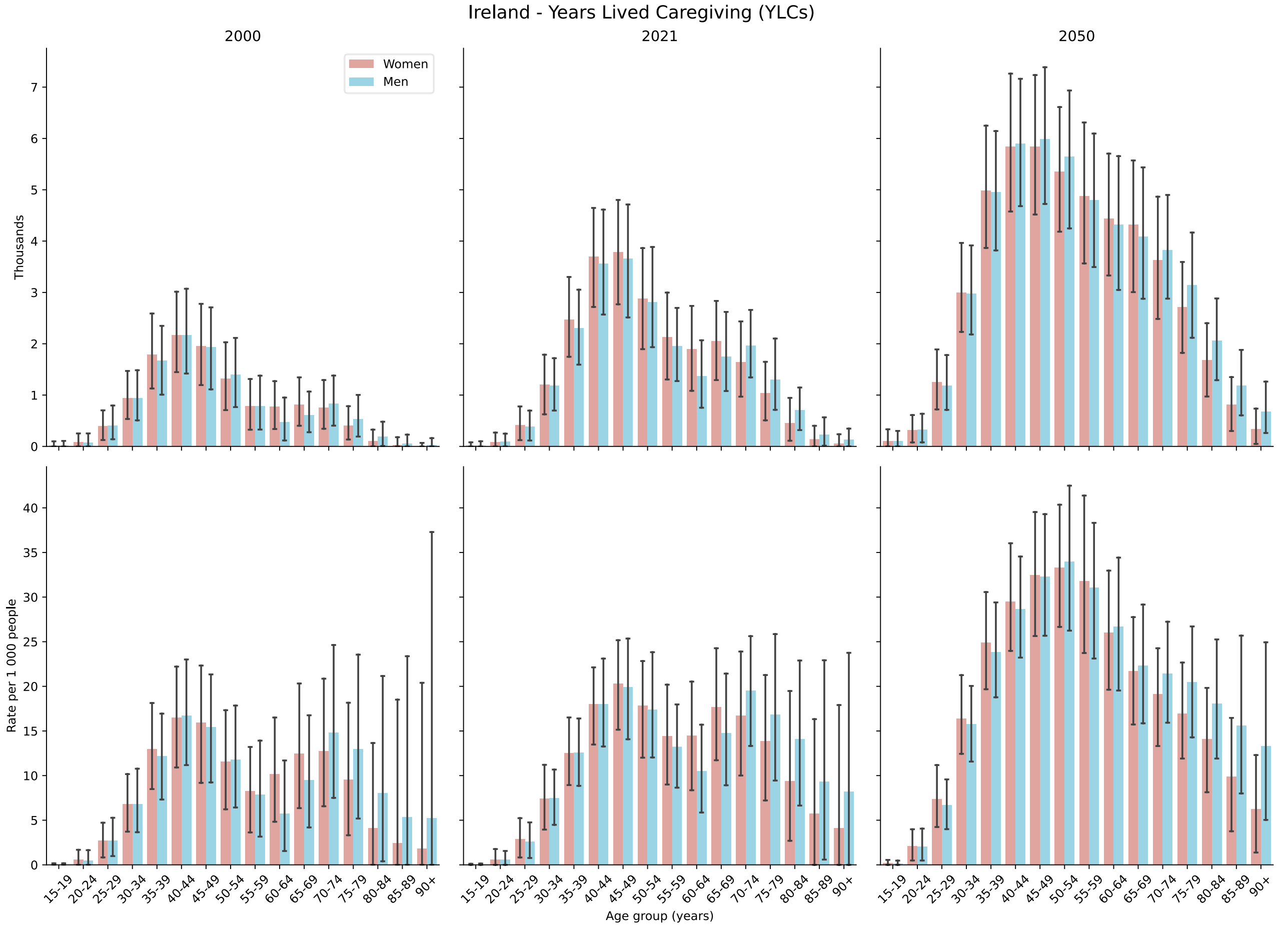
Ireland - Years Lived Caregiving (YLCs)
2000
2021
2050
Women
Men
Thousands
Rate per 1 000 people
Age group (years)
15-19
20-24
25-29
30-34
35-39
40-44
45-49
50-54
55-59
60-64
65-69
70-74
75-79
80-84
85-89
90+

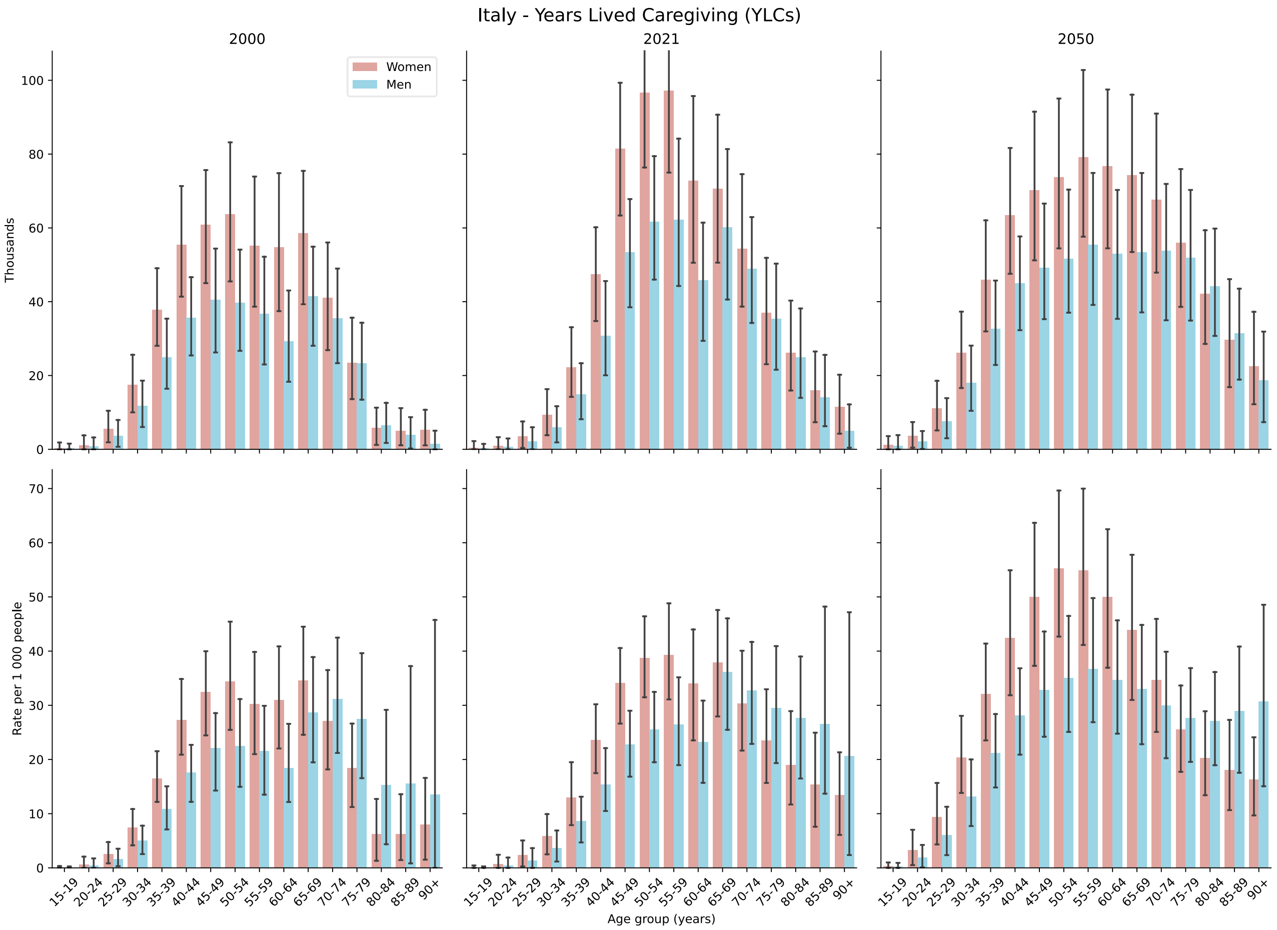
Italy - Years Lived Caregiving (YLCs)
2000
2021
2050
Women
Men
Thousands
Rate per 1 000 people
15-19
20-24
25-29
30-34
35-39
40-44
45-49
50-54
55-59
60-64
65-69
70-74
75-79
80-84
85-89
90+
Age group (years)

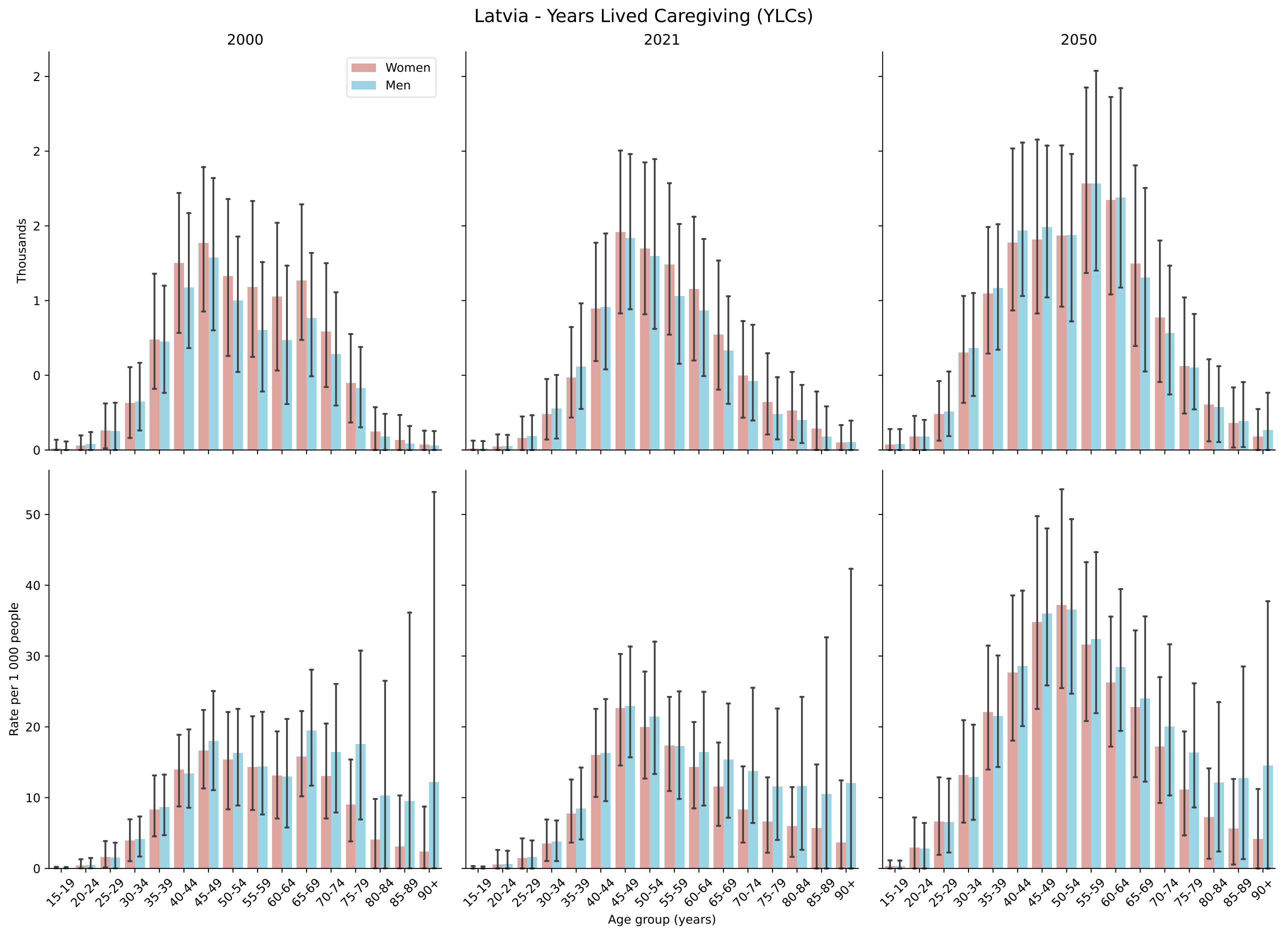
Latvia - Years Lived Caregiving (YLCs)
2000
2021
2050
Women
Men
Thousands
Rate per 1 000 people
15-19
20-24
25-29
30-34
35-39
40-44
45-49
50-54
55-59
60-64
65-69
70-74
75-79
80-84
85-89
90+
Age group (years)

Lithuania - Years Lived Caregiving (YLCs)

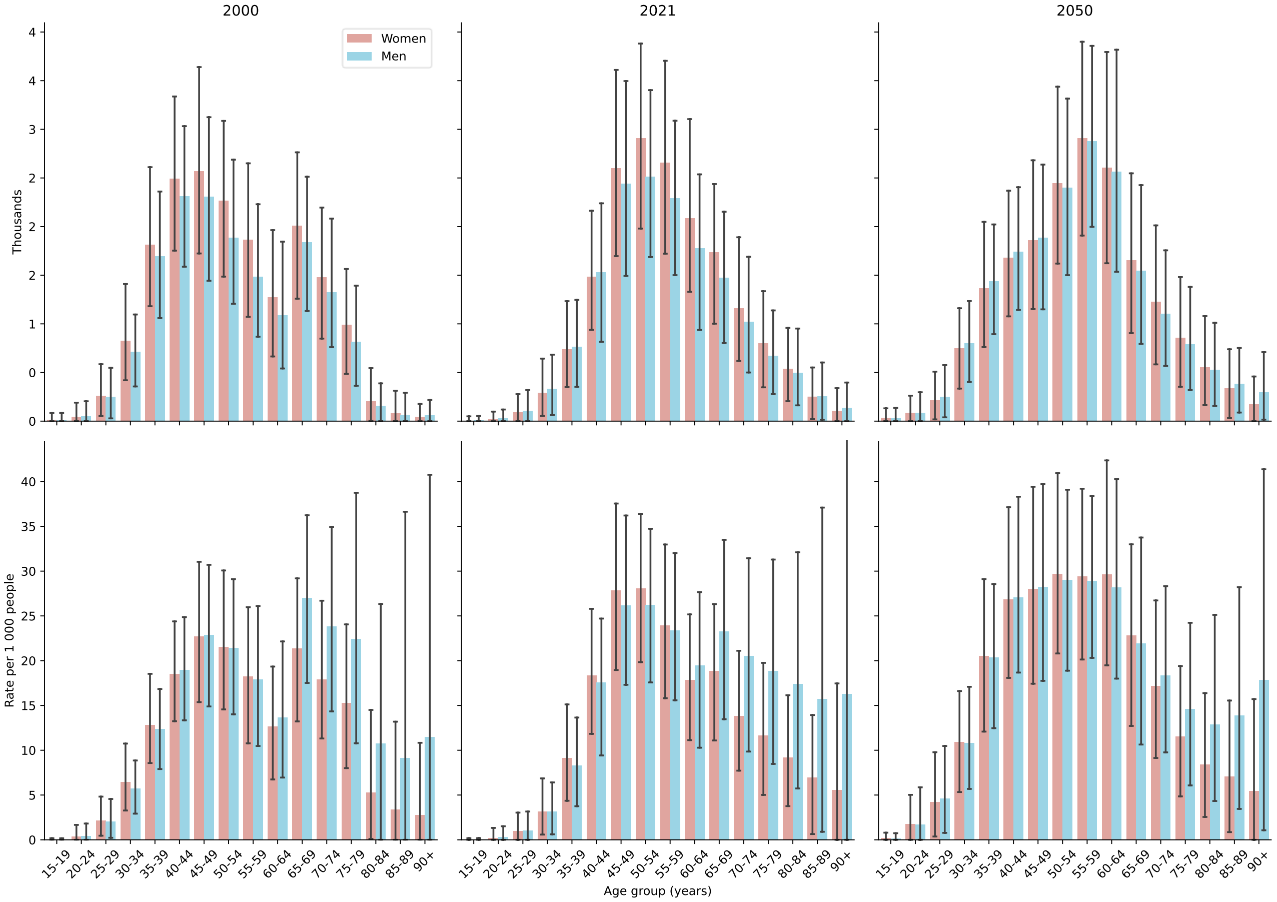

Malta - Years Lived Caregiving (YLCs)

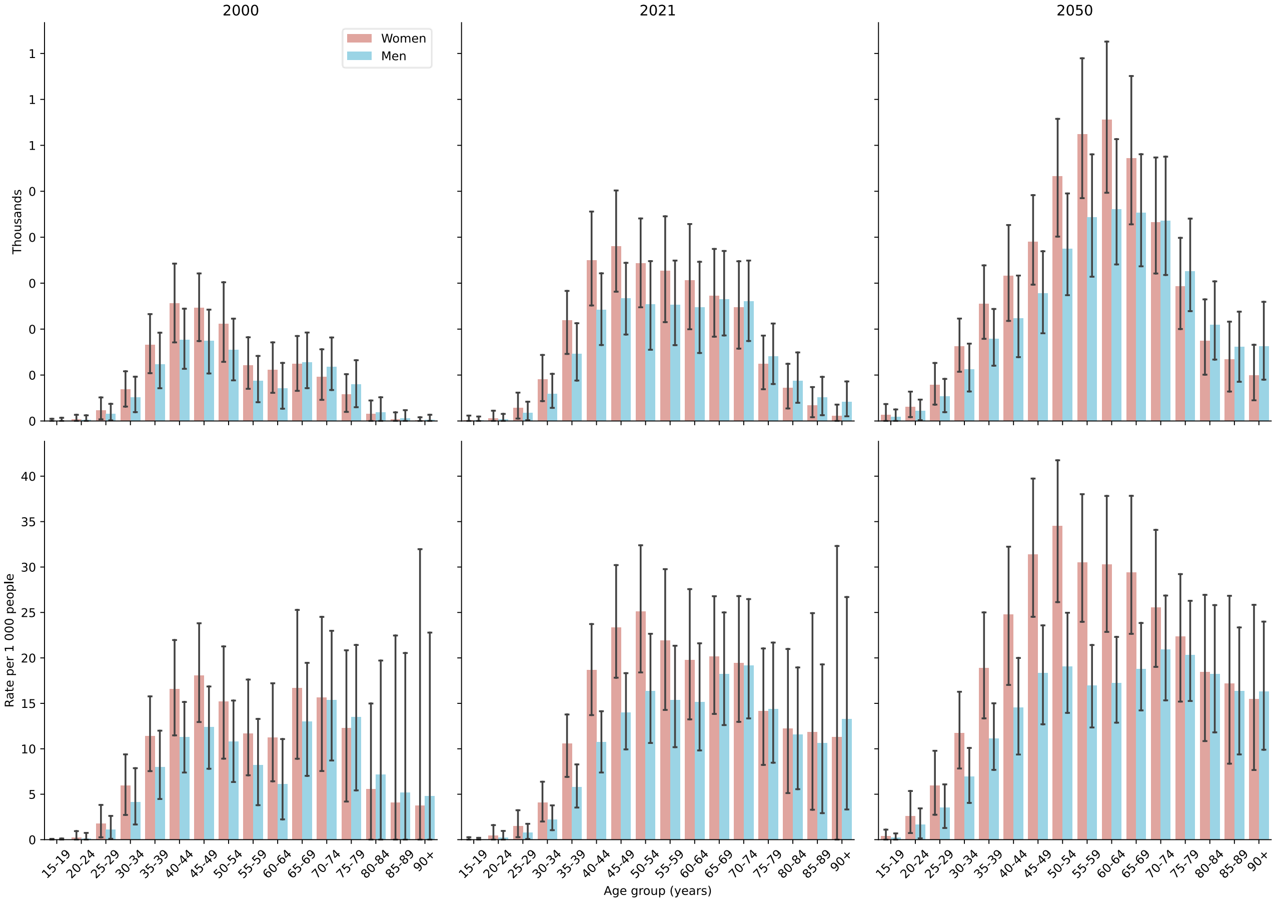

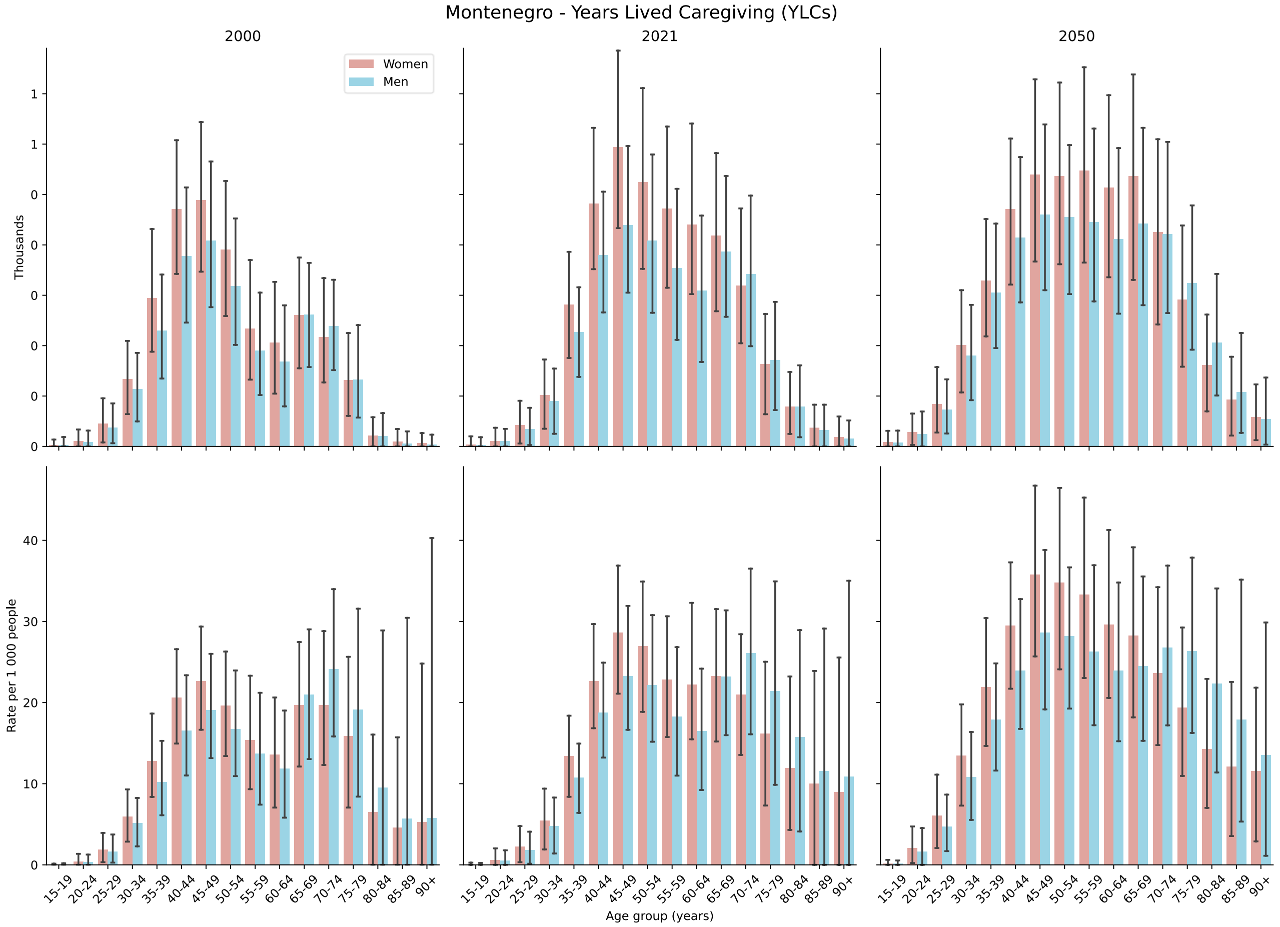
Montenegro - Years Lived Caregiving (YLCs)
2000
2021
2050
Women
Men
Thousands
Rate per 1 000 people
Age group (years)
15-19
20-24
25-29
30-34
35-39
40-44
45-49
50-54
55-59
60-64
65-69
70-74
75-79
80-84
85-89
90+

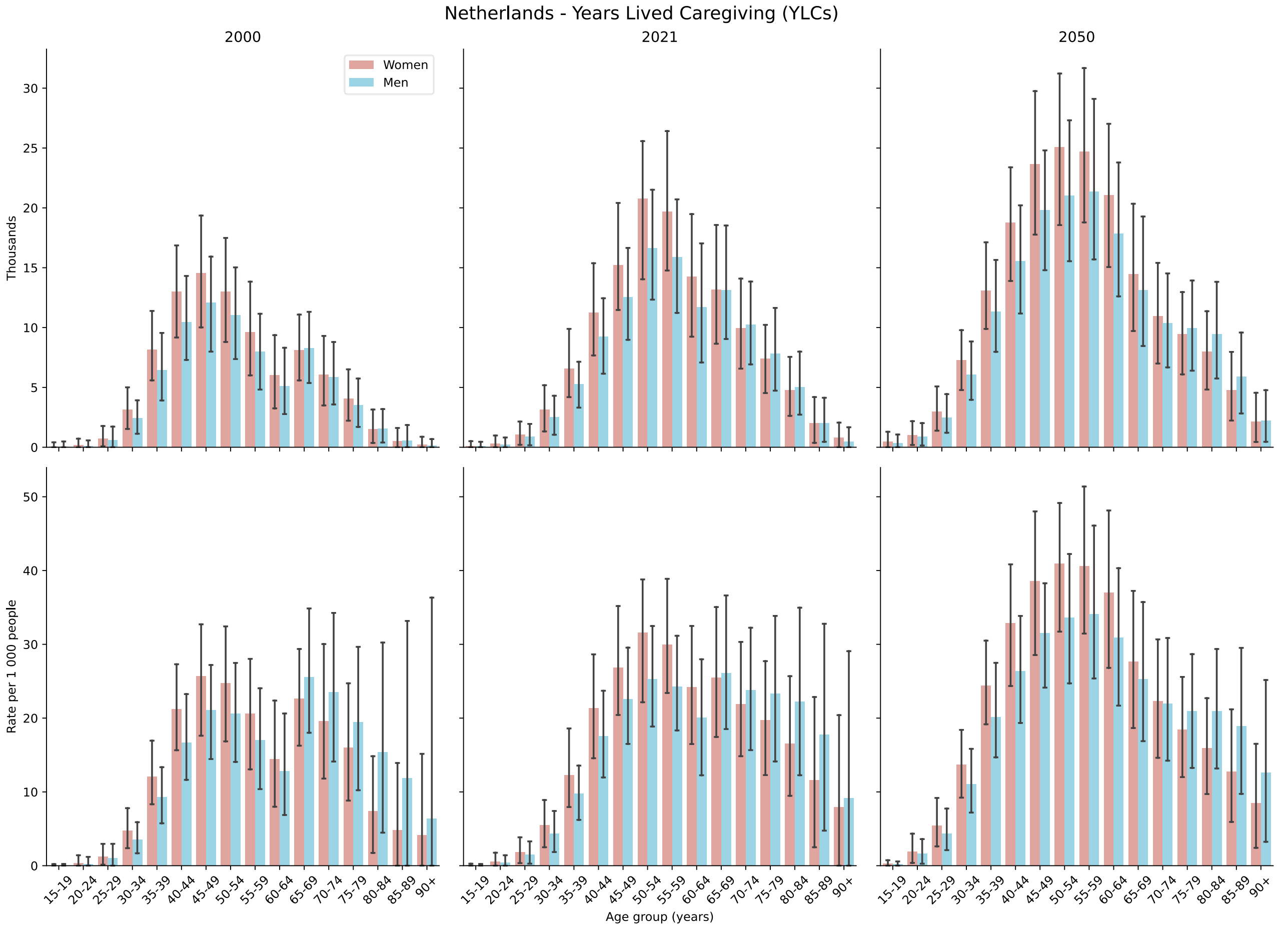
Netherlands - Years Lived Caregiving (YLCs)
2000
2021
2050
Women
Men
Thousands
Rate per 1 000 people
Age group (years)
15-19
20-24
25-29
30-34
35-39
40-44
45-49
50-54
55-59
60-64
65-69
70-74
75-79
80-84
85-89
90+

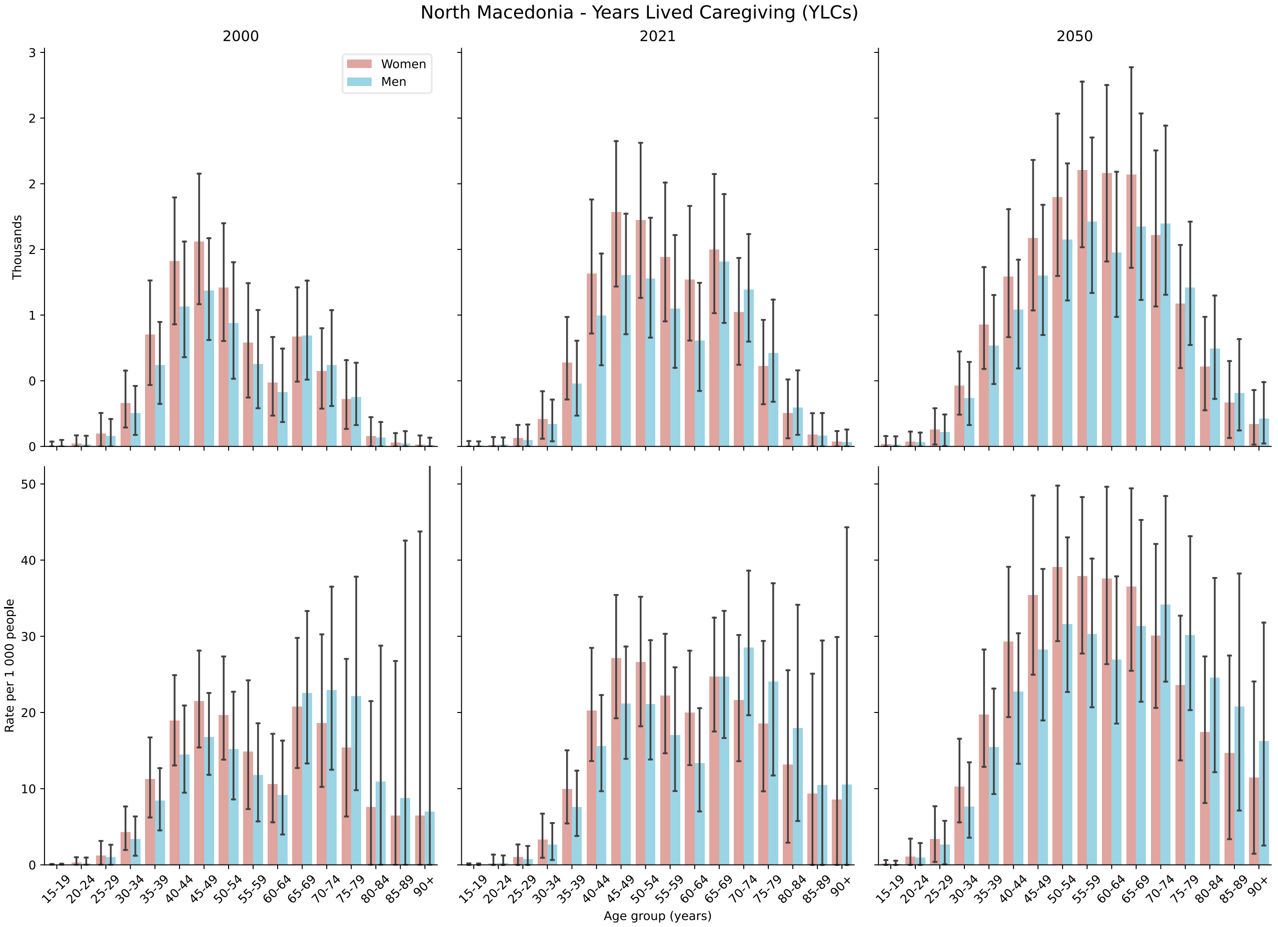
North Macedonia - Years Lived Caregiving (YLCs)
2000
2021
2050
Women
Men
Thousands
Rate per 1 000 people
Age group (years)
15-19
20-24
25-29
30-34
35-39
40-44
45-49
50-54
55-59
60-64
65-69
70-74
75-79
80-84
85-89
90+

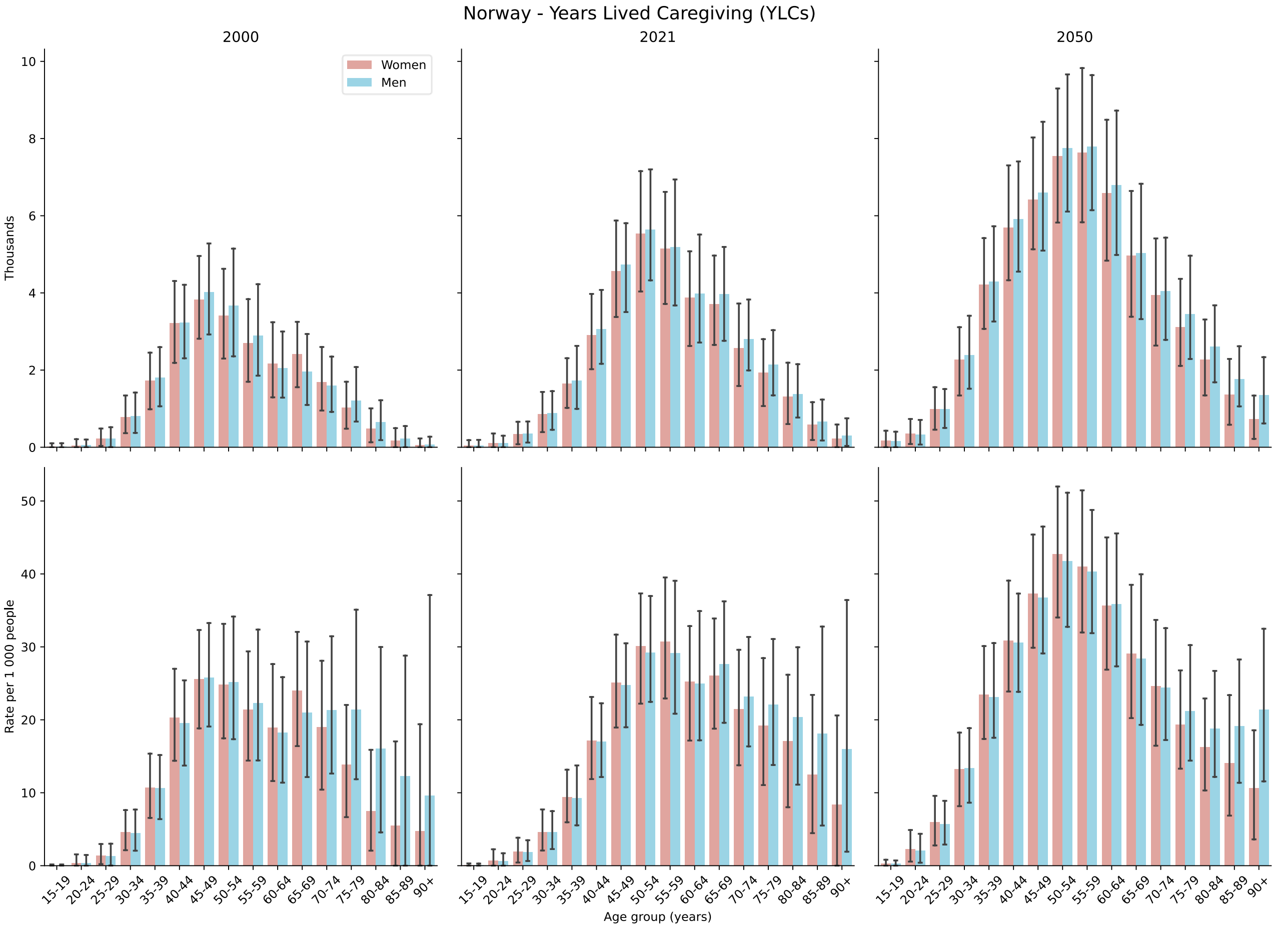
Norway - Years Lived Caregiving (YLCs)
2000
2021
2050
Women
Men
Thousands
Rate per 1 000 people
Age group (years)
15-19
20-24
25-29
30-34
35-39
40-44
45-49
50-54
55-59
60-64
65-69
70-74
75-79
80-84
85-89
90+

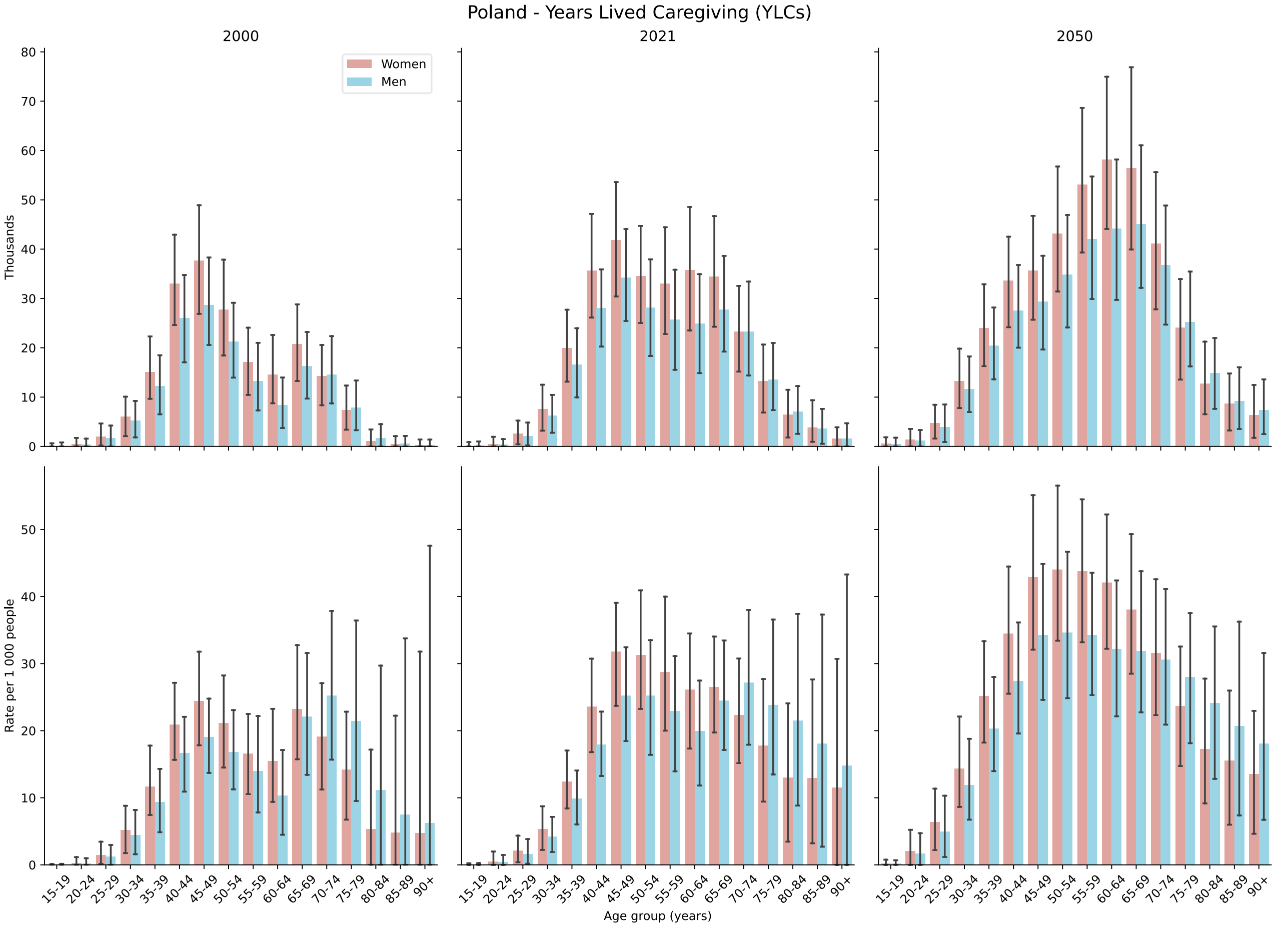
Poland - Years Lived Caregiving (YLCs)
2000
2021
2050
Women
Men
Thousands
Rate per 1 000 people
Age group (years)
15-19
20-24
25-29
30-34
35-39
40-44
45-49
50-54
55-59
60-64
65-69
70-74
75-79
80-84
85-89
90+

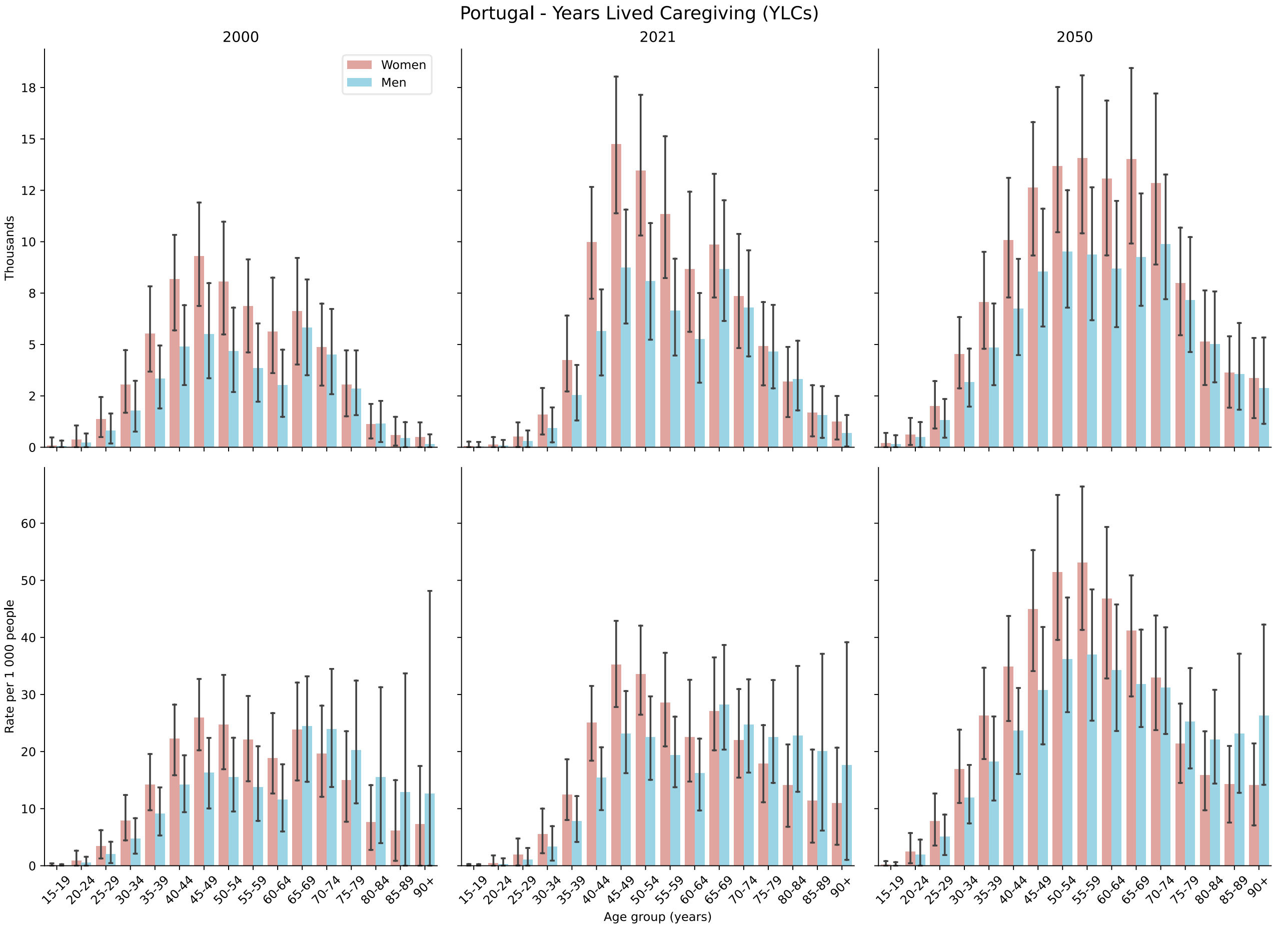
Portugal - Years Lived Caregiving (YLCs)
2000
2021
2050
Women
Men
Thousands
Rate per 1 000 people
Age group (years)
15-19
20-24
25-29
30-34
35-39
40-44
45-49
50-54
55-59
60-64
65-69
70-74
75-79
80-84
85-89
90+

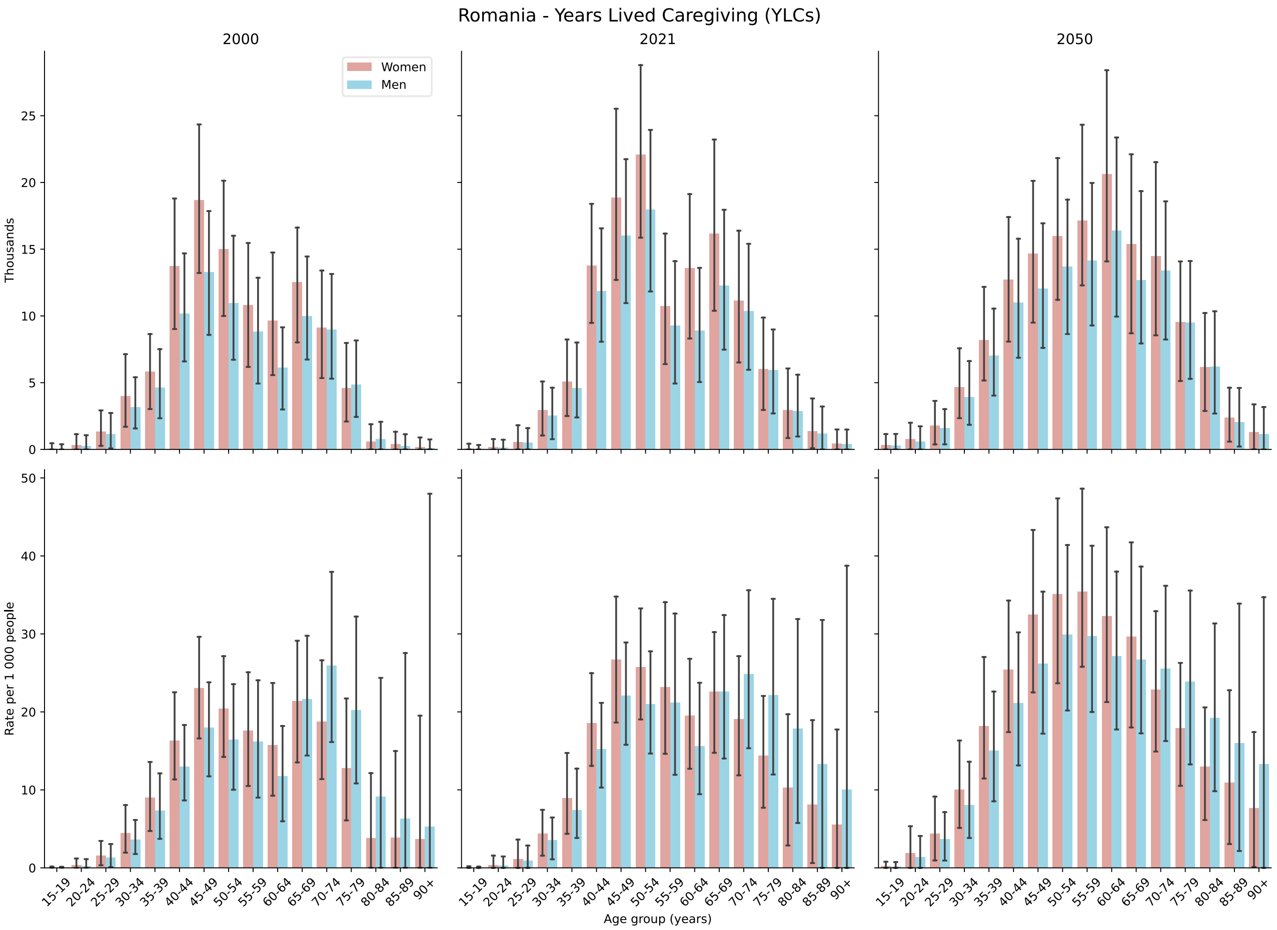
Romania - Years Lived Caregiving (YLCs)
2000
2021
2050
Women
Men
Thousands
Rate per 1 000 people
Age group (years)
15-19
20-24
25-29
30-34
35-39
40-44
45-49
50-54
55-59
60-64
65-69
70-74
75-79
80-84
85-89
90+

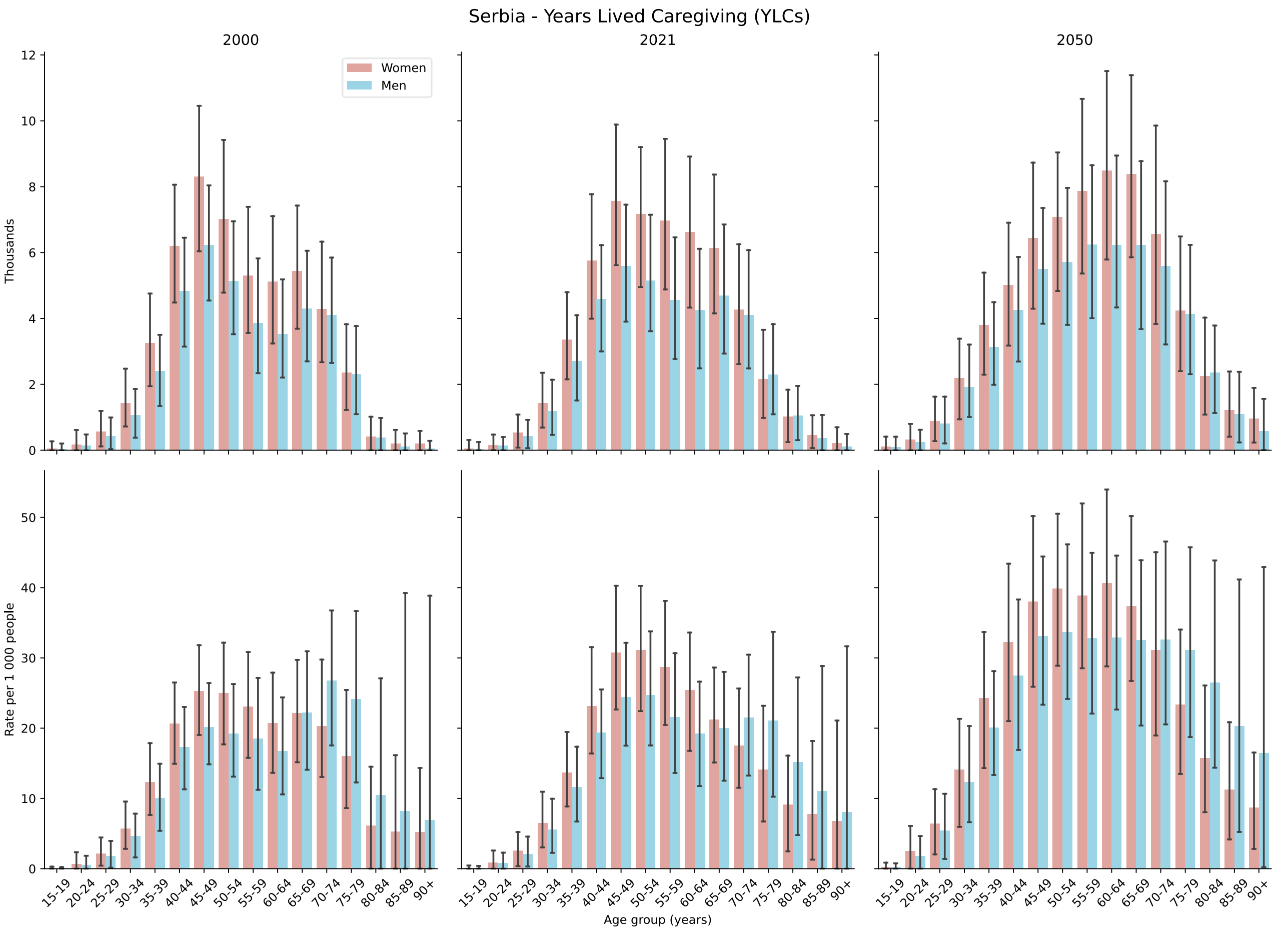
Serbia - Years Lived Caregiving (YLCs)
2000
2021
2050
Women
Men
Thousands
Rate per 1 000 people
Age group (years)
15-19
20-24
25-29
30-34
35-39
40-44
45-49
50-54
55-59
60-64
65-69
70-74
75-79
80-84
85-89
90+

Slovakia - Years Lived Caregiving (YLCs)

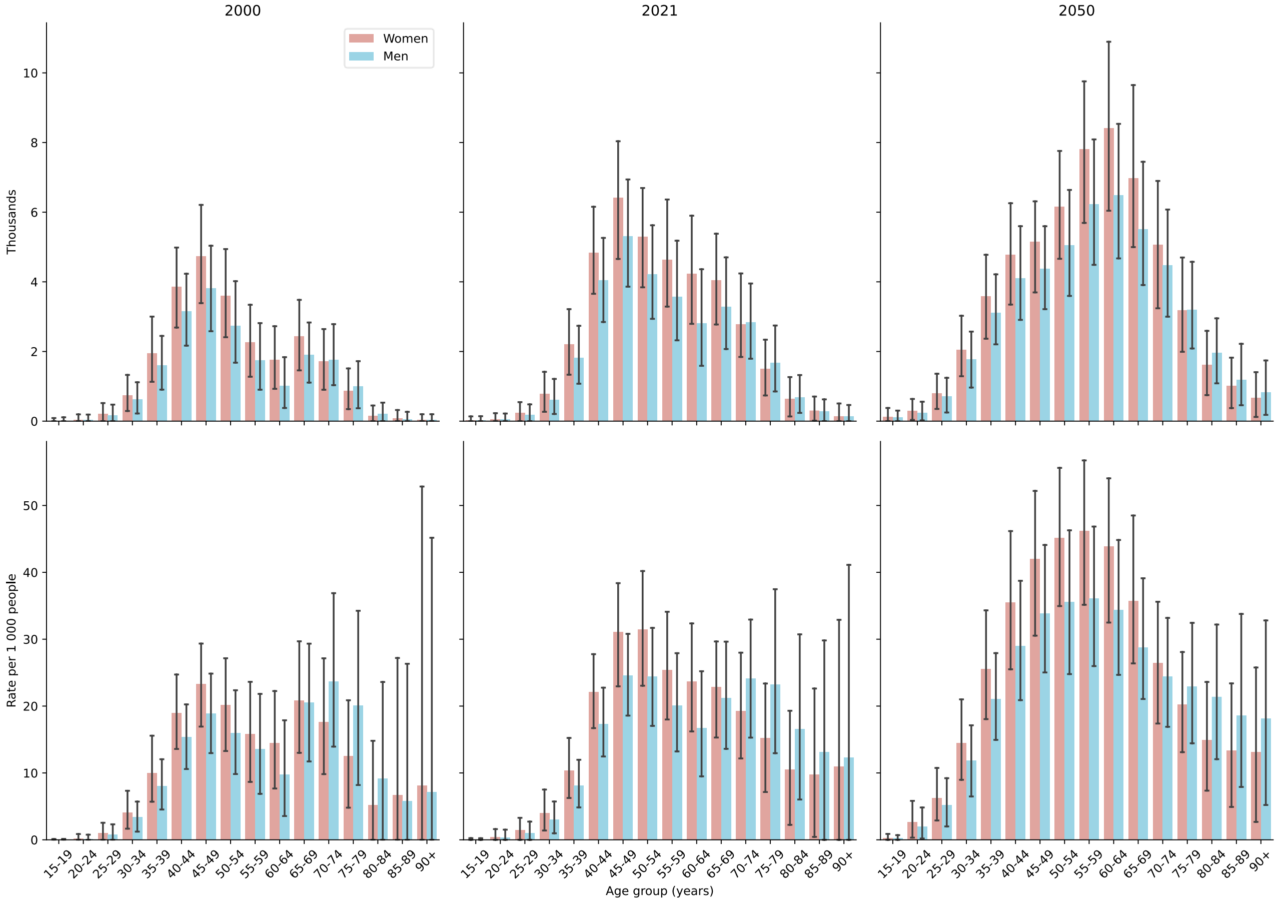

Slovenia - Years Lived Caregiving (YLCs)

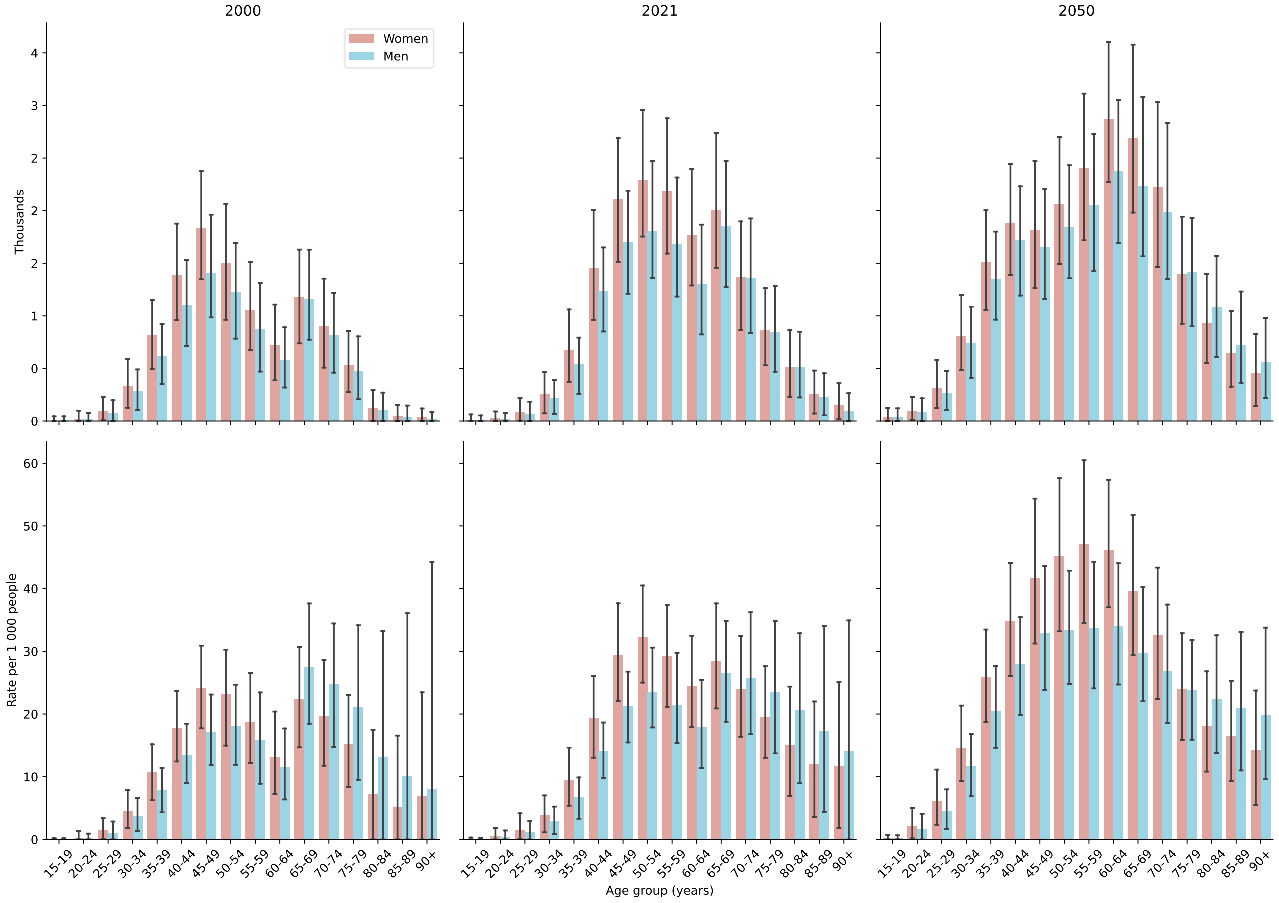

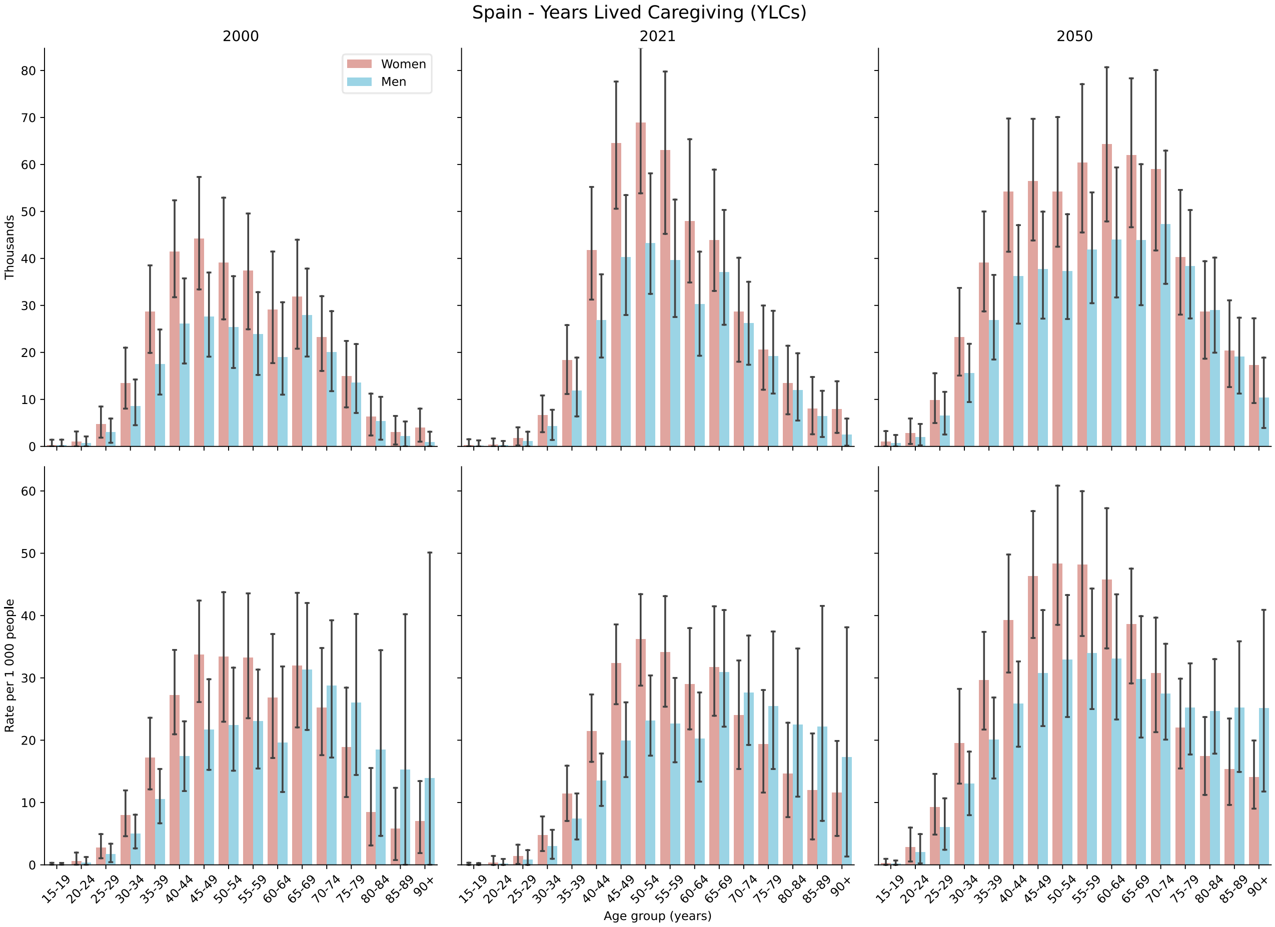
Spain - Years Lived Caregiving (YLCs)
2000
2021
2050
Women
Men
Thousands
Rate per 1 000 people
Age group (years)
15-19
20-24
25-29
30-34
35-39
40-44
45-49
50-54
55-59
60-64
65-69
70-74
75-79
80-84
85-89
90+

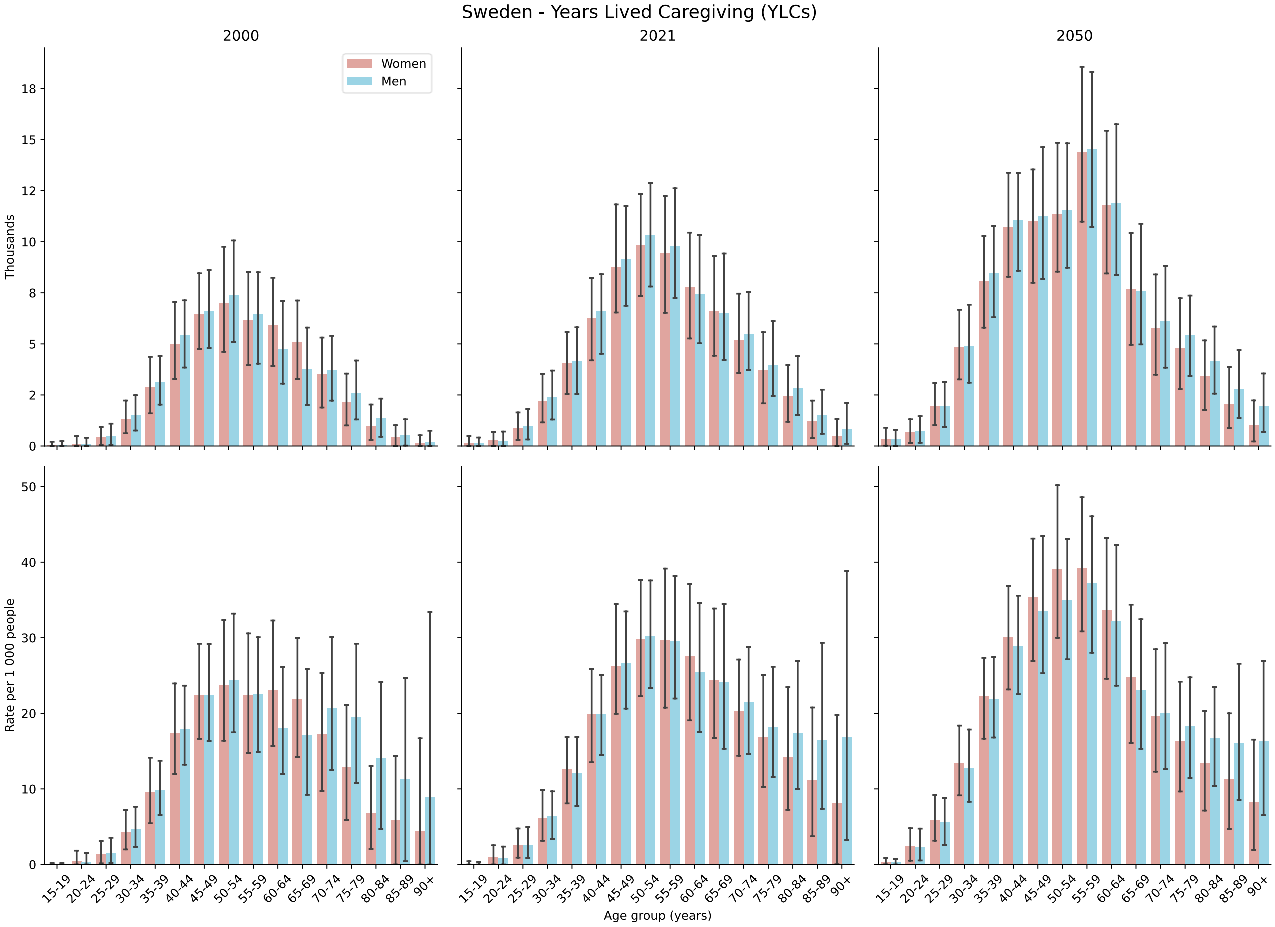
Sweden - Years Lived Caregiving (YLCs)
2000
2021
2050
Women
Men
Thousands
Rate per 1 000 people
Age group (years)
15-19
20-24
25-29
30-34
35-39
40-44
45-49
50-54
55-59
60-64
65-69
70-74
75-79
80-84
85-89
90+

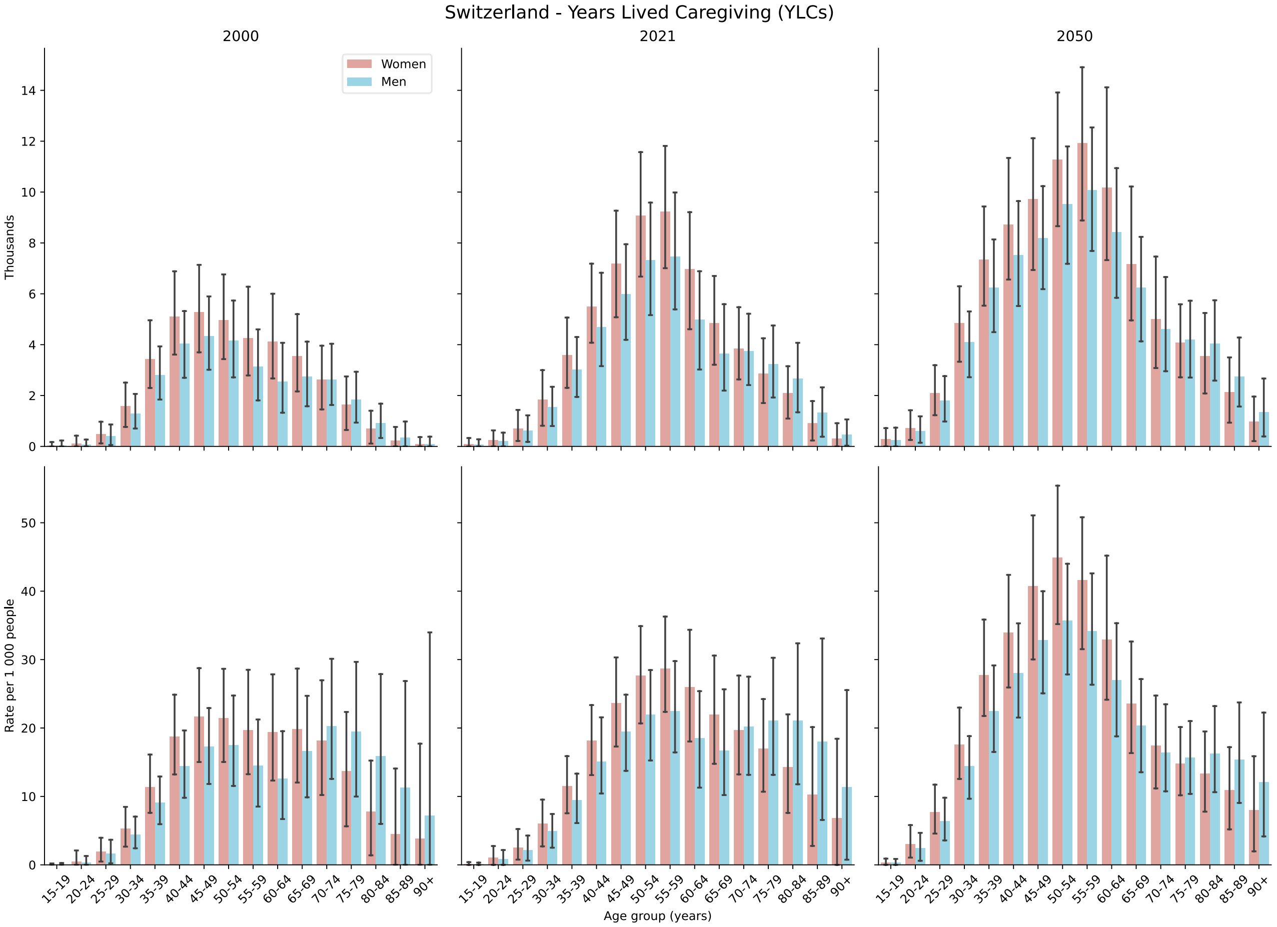

Switzerland - Years Lived Caregiving (YLCs)
2000
2021
2050
Women
Men
Thousands
Rate per 1 000 people
Age group (years)
15-19
20-24
25-29
30-34
35-39
40-44
45-49
50-54
55-59
60-64
65-69
70-74
75-79
80-84
85-89
90+

Data Sources, Limitations, and Imputation Methods

Table S8. Overview of data sources utilized in the study, including their latest update, known limitations, and imputation methods applied

Data source	Description	Latest update	Known limitations	Imputation method used [2]
GBD Foresight	Estimates and projections of prevalence of health conditions and disability weights.	May 2024	GBD reports limited data quality for some regions, although Europe is generally well covered. Includes 81 drivers of health, but other drivers and potential threats were not incorporated.	-
UN World Population Prospects (UN WPP) 2024	Population data (fertility, mortality, and migration).	July 2024	Data sometimes differ from official statistics, as UN WPP seeks to resolve inconsistencies affecting the basic data to establish past population trends.	-
Eurostat Marital and Divorces statistics	Harmonized demographic data on marriages, divorces, and cohabitation rates across Europe.	November 2024	Reporting gaps exist in some smaller member states and for specific years.	Interpolation with logistic growth curves.
OECD Same-Sex Marriages Statistics	Data on same-sex marriages and partnerships.	June 2022	Coverage limited to OECD countries; cultural and legal variations in definitions and reporting.	Modeled estimates based on regional averages.
Eurostat LTC Bed Availability	Data on long-term care bed availability in healthcare and social care facilities in Europe.	September 2024	Gaps for specific countries, particularly in Eastern and Southern Europe. Inconsistent definitions of LTC beds. Data for Portugal not available.	Linear interpolation for missing data. Portugal data imputed from report of the Portughese Public Finance Council.
SHARE Survey Wave 9	Microdata on health, socio-economic status, and social networks of individuals aged 50+ in Europe.	March 2024	Self-reported data may be subject to recall bias; some regional variability in data completeness and representativeness.	-

[2] The column "Imputation method used" refers specifically to the data operations conducted as part of this study. Data providers may have employed additional data cleaning or imputation techniques to produce their respective datasets.

References

[1] V. Grimm *et al.*, “A standard protocol for describing individual-based and agent-based models,” *Ecol Modell*, vol. 198, no. 1–2, pp. 115–126, Sep. 2006, doi: 10.1016/J.ECOLMODEL.2006.04.023.

[2] V. Grimm *et al.*, “The ODD Protocol for Describing Agent-Based and Other Simulation Models: A Second Update to Improve Clarity, Replication, and Structural Realism,” *2019:147:2*, vol. 23, no. 2, Mar. 2020, doi: 10.18564/JASSS.4259.

[3] A. J. Ferrari *et al.*, “Global incidence, prevalence, years lived with disability (YLDs), disability-adjusted life-years (DALYs), and healthy life expectancy (HALE) for 371 diseases and injuries in 204 countries and territories and 811 subnational locations, 1990–2021: a systematic analysis for the Global Burden of Disease Study 2021,” *The Lancet*, vol. 403, no. 10440, pp. 2133–2161, May 2024, doi: 10.1016/S0140-6736(24)00757-8.

[4] A. Cieza, K. Causey, K. Kamenov, S. W. Hanson, S. Chatterji, and T. Vos, “Global estimates of the need for rehabilitation based on the Global Burden of Disease study 2019: a systematic analysis for the Global Burden of Disease Study 2019,” *The Lancet*, vol. 396, no. 10267, pp. 2006–2017, Dec. 2020, doi: 10.1016/S0140-6736(20)32340-0.

[5] C. Mason, “Demographic Models,” in *Contributions to Economic Analysis*, vol. 293, Emerald Group Publishing Ltd., 2014, pp. 345–365. doi: 10.1108/S0573-855520140000293010.

[6] S. E. Vollset *et al.*, “Burden of disease scenarios for 204 countries and territories, 2022–2050: a forecasting analysis for the Global Burden of Disease Study 2021,” *The Lancet*, vol. 403, no. 10440, pp. 2204–2256, May 2024, doi: 10.1016/S0140-6736(24)00685-8.

[7] United Nations - Population Division, “World Population Prospects 2024.” Accessed: Jul. 30, 2024. [Online]. Available: https://population.un.org/wpp/

[8] M. Snyder, D. Alburez-Gutierrez, I. Williams, and E. Zagheni, “Estimates from 31 countries show the significant impact of COVID-19 excess mortality on the incidence of family bereavement,” *Proc Natl Acad Sci U S A*, vol. 119, no. 26, p. e2202686119, Jun. 2022, doi: 10.1073/PNAS.2202686119/SUPPL_FILE/PNAS.2202686119.SAPP.PDF.

[9] OECD, “Demographic module — SPHeP-NCDs documentation.” Accessed: May 20, 2024. [Online]. Available: http://oecdpublichealthexplorer.org/ncd-doc/demography/head.html

[10] Eurostat, “Marriage indicators.” Accessed: May 20, 2024. [Online]. Available: https://ec.europa.eu/eurostat/databrowser/view/demo_nind__custom_8613119/default/table?lang=en

[11] S. J. Taylor and B. Letham, “Forecasting at Scale,” *Am Stat*, vol. 72, no. 1, pp. 37–45, Jan. 2018, doi: 10.1080/00031305.2017.1380080.

[12] OECD, “OECD Family Database.” Accessed: May 20, 2024. [Online]. Available: https://www.oecd.org/els/family/database.htm

[13] Eurostat, “First marriage rates by age and sex.” Accessed: May 20, 2024. [Online]. Available: https://ec.europa.eu/eurostat/databrowser/view/demo_nsinrt__custom_9872965/bookmark/table?lang=en&bookmarkId=e9118d6a-c18c-4256-84b0-353957e12cdd

[14] Eurostat, “Marriage indicators.” Accessed: May 20, 2024. [Online]. Available: https://ec.europa.eu/eurostat/databrowser/view/demo_nind__custom_10630016/bookmark/table?lang=en&bookmarkId=d1cb5580-e970-41a5-8635-1c7f92b5426d

[15] Eurostat, “Divorce indicators.” Accessed: May 20, 2024. [Online]. Available: https://ec.europa.eu/eurostat/databrowser/view/demo_ndivind/default/table?lang=en

[16] Eurostat, “Divorces by duration of marriage (reached during the year).” Accessed: May 20, 2024. [Online]. Available: https://ec.europa.eu/eurostat/databrowser/view/demo_ndivdur__custom_8827001/default/table?lang=en

[17] Institute for Health Metrics and Evaluation (IHME), “WHO Rehabilitation Need Estimator.” Accessed: May 10, 2024. [Online]. Available: https://vizhub.healthdata.org/rehabilitation/

[18] S. E. Vollset *et al.*, “Burden of disease scenarios for 204 countries and territories, 2022-2050: a forecasting analysis for the Global Burden of Disease Study 2021,” *The Lancet*, vol. 403, no. 10440, pp. 2204–2256, May 2024, doi: 10.1016/S0140-6736(24)00685-8.

[19] Eurostat, “Hospital beds by function and type of care.”

[20] Eurostat, “Beds in nursing and other residential long-term care facilities.”

[21] “NHS performance in 2021.” Accessed: Nov. 19, 2024. [Online]. Available: https://www.cfp.pt/en/publications/general-government-sectors/nhs-performance-in-2021

[22] SHARE-ERIC, “Survey of Health, Ageing and Retirement in Europe (SHARE) Wave 9. Release version: 9.0.0,” 2024.

[23] A. Börsch-Supan *et al.*, “Data Resource Profile: The Survey of Health, Ageing and Retirement in Europe (SHARE),” *Int J Epidemiol*, vol. 42, no. 4, pp. 992–1001, Aug. 2013, doi: 10.1093/IJE/DYT088.

[24] C. Mason, “Socsim Oversimplified,” 2016.

[25] J. A. Salomon *et al.*, “Disability weights for the Global Burden of Disease 2013 study,” *Lancet Glob Health*, vol. 3, no. 11, pp. e712–e723, Nov. 2015, doi: 10.1016/S2214-109X(15)00069-8.

[26] World Health Organization, “WHO methods and data sources for global burden of disease estimates 2000-2019.” Accessed: May 24, 2024. [Online]. Available: https://cdn.who.int/media/docs/default-source/gho-documents/global-health-estimates/ghe2019_daly-methods.pdf

[27] MathWorks, “Computational Advantages of Sparse Matrices.” Accessed: May 30, 2024. [Online]. Available: https://it.mathworks.com/help/matlab/math/computational-advantages-of-sparse-matrices.html

[28] M. I. Broese van Groenou and A. De Boer, “Providing informal care in a changing society,” *Eur J Ageing*, vol. 13, no. 3, pp. 271–279, Sep. 2016, doi: 10.1007/S10433-016-0370-7/TABLES/1.

[29] S. K. Smith, J. Tayman, and D. A. Swanson, “Overview of the Cohort-Component Method,” *Springer Series on Demographic Methods and Population Analysis*, vol. 37, pp. 45–50, 2013, doi: 10.1007/978-94-007-7551-0_3/FIGURES/2.

[30] T. K. Burch, “The Cohort-Component Population Projection: A Strange Attractor for Demographers,” *Demographic Research Monographs*, pp. 135–151, 2018, doi: 10.1007/978-3-319-65433-1_10.

[31] P. Virtanen *et al.*, “SciPy 1.0: fundamental algorithms for scientific computing in Python,” *Nature Methods 2020 17:3*, vol. 17, no. 3, pp. 261–272, Feb. 2020, doi: 10.1038/s41592-019-0686-2.

[32] Eurostat, “Population by family status and NUTS 3 region.” Accessed: May 10, 2024. [Online]. Available: https://ec.europa.eu/eurostat/databrowser/view/cens_11fs_r3__custom_9857335/bookmark/table?lang=en&bookmarkId=02272ff3-ca11-47ea-9ec8-ca369df90ba3

[33] Eurostat, “Glossary: Consensual union.” Accessed: May 20, 2024. [Online]. Available: https://ec.europa.eu/eurostat/statistics-explained/index.php?title=Glossary:Consensual_union
